# Azine Activation via Silylium Catalysis

**DOI:** 10.1021/jacs.1c03257

**Published:** 2021-04-28

**Authors:** Carla Obradors, Benjamin List

**Affiliations:** Max-Planck-Institut für Kohlenforschung, Kaiser-Wilhelm-Platz 1, Mülheim an der Ruhr, 45470, Germany

## Abstract

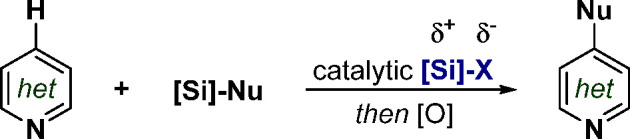

Practical, efficient, and general methods for the diversification of *N*-heterocycles have been a recurrent goal in chemical synthesis due to the ubiquitous influence of these motifs within bioactive frameworks. Here, we describe a direct, catalytic, and selective functionalization of azines via silylium activation. Our catalyst design enables mild conditions and a remarkable functional group tolerance in a one-pot setup.

Nitrogen-based heterocycles constitute cardinal pharmacophores in a myriad of biologically active products spanning from synthetic drugs to agrochemicals.^1^ Still, retrosynthetic analysis of representative targets relies largely on engineered ring condensations and manipulation of prefunctionalized building blocks.^2^ An array of alternative methods toward late-stage diversification of complex *N*-heterocycles has consequently arisen,^3^ capitalizing on Minisci-type reactions, transition-metal-mediated C–H activation processes, or photoredox transformations.^4^ While significant progress has been achieved, limited selectivity, harsh conditions, or a restricted scope is rather common and preactivation of the substrate remains the prevailing approach to date (Figure 1A).^5^ Thus, complementing *N*-acylation and alkylation approaches,^6^ perhaps the most prominent strategy involves the formation of an *N*-oxide motif to enable a nucleophilic addition to the aromatic ring.^7^ Despite its vast utility, this classical route requires prior preparation—if not isolation—of sensitive intermediates, followed by appendage of the desired scaffold. A tedious step to remove the activating group is also frequently necessary, leading to stoichiometric waste generation. In addition, the required reagents often limit the functional group tolerance of the overall transformation. Therefore, the design of novel methodologies allowing for milder conditions and direct disconnections is a recurrent challenge for chemical synthesis. The preparation of phosphonium salts reported by McNally et al. and a novel bifunctional reagent described by Fier stand out as the latest annexes to the toolkit.^8,9^ Furthermore, the Buchwald group recently reported an asymmetric copper-catalyzed addition of styrenes to pyridines, in which turnover is achieved upon reaction of the organometallic species with an external reductant.^10^

**Figure 1:**
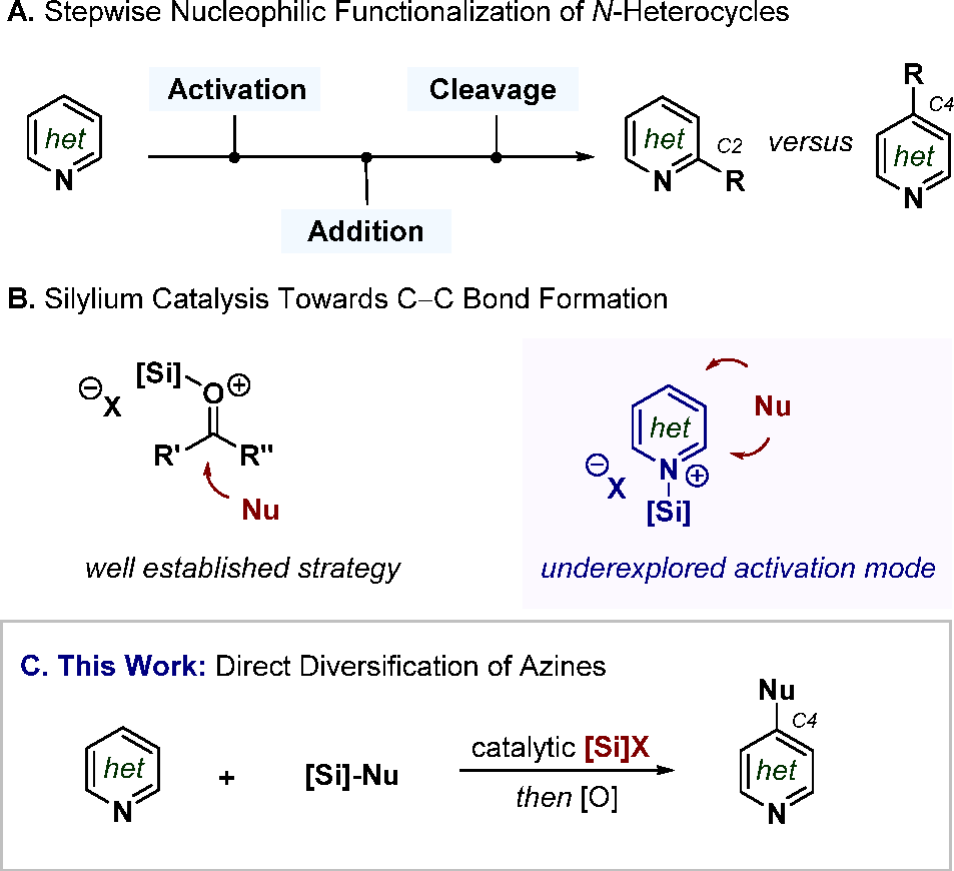
(A) Traditional approach for the functionalization of *N*-heterocycles with nucleophiles. (B) Silylium-based Lewis acid catalysis. (C) This work: direct, efficient and general diversification of azines by means of silylium catalyst design.

Silylium-based Lewis acid catalysis is a vastly useful and powerful approach to the activation of oxygeneous compounds, and our group has contributed several enantioselective examples of this type of organocatalysis (Figure 1B).^11^ By means of catalyst design, long Si–X bonds in an ion pair offer little stabilization, leading to highly electrophilic and extremely reactive silylium activated cations.^12,13^ Such structural features can be achieved with decreasing basicity of the counteranion along with steric constraints. However, while both carbonyl compounds and pyridines can bind to the silylium ion,^14^ even to the extent that pyridines can inhibit catalysis in carbonyl transformations, to our knowledge, catalytic silylium activation of azines toward the formation of carbon–carbon bonds has remained unprecedented. We hypothesized that we could potentially turn this conventional setback into a solution for the longstanding challenge of *N*-heterocycle functionalization (Figure 1C). Moreover, the use of organosilicon compounds would turn the addition step concomitant to the regeneration of the catalyst.^15,16^ In this manner, the sole presence of a catalyst would permit a highly practical assembly between substrate and nucleophile.

Here, we report the addition of silyl ketene acetals (SKA) to azines via silylium ion catalysis. Our new transformation proceeds without preactivation of the substrate and with complete C4-regioselectivity.^17^ The active species is generated *in situ* from a Brønsted acid precatalyst (**HX**) upon protodesilylation of the SKA.^18^ The design based on high electrophilicity allows mild conditions to obtain high yields with a broad, divergent palette of scaffolds. Straightforward rearomatization via oxidation furnishes the functionalized product in a one-pot fashion.

Initial proof of concept was established when alkylative dearomatization of 3-nitropyridine (**1a**) was observed by ^1^H NMR using 1 mol % of triflimide (Tf_2_NH) in the presence of SKA **2a** (Figure 2A). Effective isolation of the resulting *N*-silylated dihydropyridine (**3a**) proved to be rather challenging but structural assignment was confirmed via single-crystal X-ray diffraction, uncovering planarization of the endocyclic nitrogen due to conjugation. In parallel, the more electron-deficient substrate **1b**—with the potential activation site sterically impeded—showed no reactivity and questioned the role of the nitro moiety as a directing group as well as a potential noncatalyzed reaction. Moreover, sequentially increasing the electron density of the aromatic ring led to a drop of the addition yield (**1c** > **1d** > **1e**). ^19^F NMR analysis of the triflimide catalyst speciation revealed efficient silylation of all three substrates.^19^ Pyridine itself is also silylated as established by a peak at 41.48 ppm in ^29^Si HMBC, even though no nucleophilic addition occurred in this case. These results corroborate C–C bond formation as the most challenging step of the transformation, which in the case of electron-rich pyridines is more arduous to occur.

**Figure 2:**
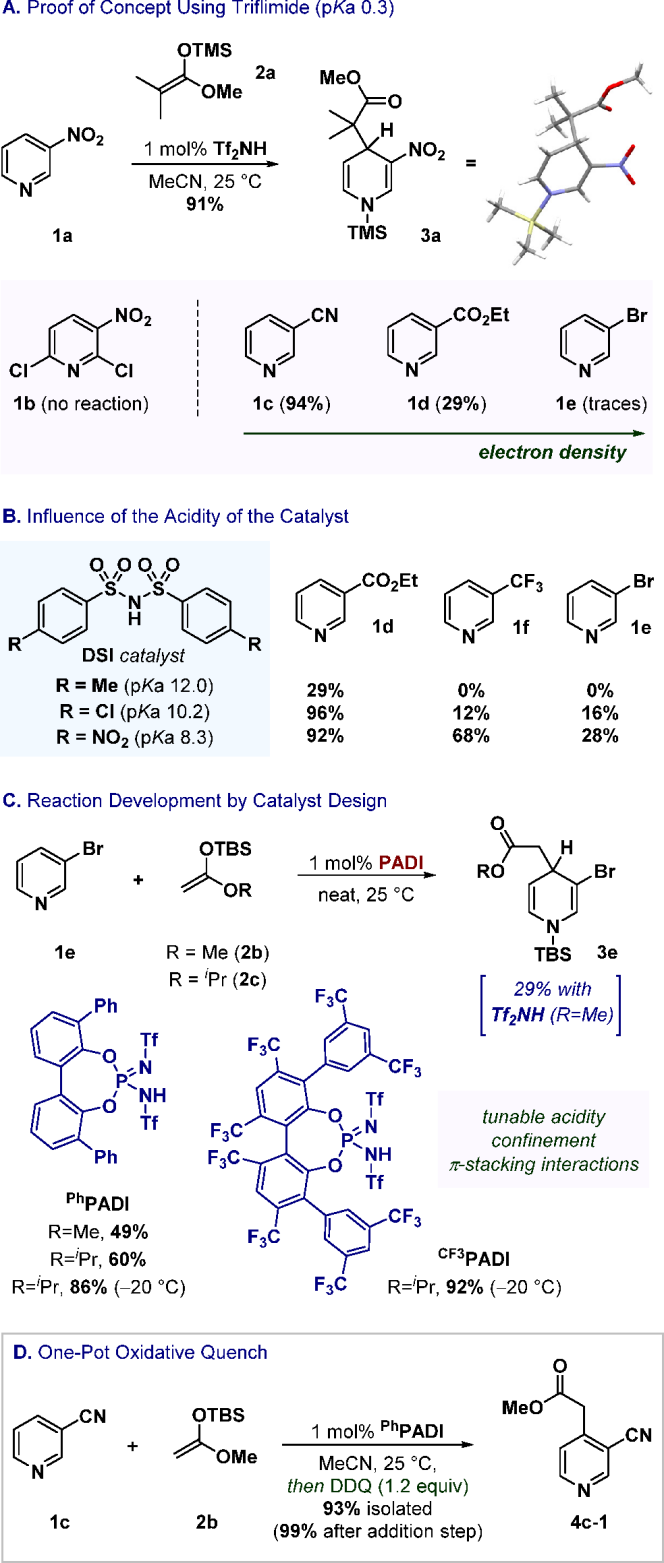
(A) Proof of concept and effects of the pyridine substitution. (B) Assessment of the catalyst acidity (p*K*_a_’s determined in MeCN).^20^ (C) Developing highly acidic and chemoselective **PADI** catalysts. (D) Practical and direct oxidation toward the functionalized product.

This scenario results from either an increase of the activation energy and/or the thermodynamic stability of the aforementioned intermediate. Examination of the reaction conditions as well as the use of α-unsubstituted SKAs showed a maximum yield of 16% of the addition to 3-bromopyridine (**1e**; see the Supporting Information (SI) for details). In this case, the SKA decomposed to a complex mixture due to a slower reaction with the *N*-heterocycle accompanied by self-condensation/polymerization. Ultimately, we investigated a fundamental pillar of this transformation: the acidity of the catalyst. A systematic analysis was performed contrasting its p*K*_a_—using comparable disulfonimides (**DSIs**)—with electronically different pyridines (Figure 2B). First, ethyl nicotinate (**1d**) rapidly delivered an excellent yield (from 29% with **DSI** p*K*_a_ 12.0 to 96% with **DSI** p*K*_a_ 10.2). The strong σ-electronegative trifluoromethyl group in pyridine **1f** required **DSI** p*K*_a_ of 8.3 for moderate efficiency (68%), suggesting that a slight complementary directing effect is in fact possible. Finally, nucleophilic addition to 3-bromopyridine (**1e**) analogously increased to 28% with the most acidic **DSI**. In spite of the clear trend of behavior within each example, less acidic catalysts proved more competent than Tf_2_NH (p*K*_a_ 0.3 in MeCN), indicating that additional structural considerations were required.

Based on the insights gathered until this point, we designed a novel scaffold anchoring in two main intertwined principles: enhanced acidity along with a more defined catalyst microenvironment, offering confinement and/or a source of noncovalent interactions (Figure 2C).^21^ Considering the work of Koppel, Yagupolskii, and Taft describing superacid parameters,^22^ we focused on the phosphoramidimidate moiety (**PADI**) to spur the increase in electrophilicity. The biphenol backbone provides the ideal platform to insert electron-withdrawing groups and further tune the reactivity. Introduction of modular 3,3′-substituents affords then the steric constraints to control the chemoselectivity. We hypothesized that this could prevent the decomposition of the SKA and selectively activate the planar *N*-heterocycle instead, potentially accelerated by additional π–π stacking interactions.^23^ Synthesis of ^**Ph**^**PADI** consists of three steps from 2-phenylphenol in 56% overall yield (see SI). This scaffold indeed catalyzed the addition of SKA **2b** to pyridine **1e** (49% versus 29% using triflimide) together with competing *N*-methylation of the substrate. Use of SKA **2c** exclusively led to the formation of dihydropyridine **3e** and was further optimized to an 86% yield (neat conditions at −20 °C, 14 h). The oligotrifluoromethylated analog further increased the yield to 92% (four steps in total to ^**CF3**^**PADI**). We ascribe this result to a higher acidity, which we exploited when more challenging *N*-heterocycles were to be activated (*vide infra*).

The ultimately devolved protocol is practical as well as mild and selective (Figure 2D). The functionalized aromatic *N*-heterocycle is obtained upon direct *in situ* oxidation of the dihydropyridine intermediate **3**. Thus, reaction of commercially available SKA **2b** with pyridine **1c** using 1 mol % of ^**Ph**^**PADI** gives a 93% yield of the isolated pyridine **4c-1** after treatment with 1.2 equiv of DDQ at 25 °C. The presence of the nitrile group suggests the orthogonal reactivity with analogous metal enolates.

The novel ^**Ph**^**PADI**, the triethylammonium salt of which an X-ray structure could be obtained, turned out to be a remarkably general catalyst that is highly efficient in the presence of a wide variety of functional groups and *N*-heterocyclic scaffolds (Figure 3). Similarly to **4c-1**, products **4a**—with the nitro group—and **4c-2** are obtained in good yields (72% and 86%, respectively), forging a challenging quaternary center α to C4. Formation of product **4d** including the ester functionality (82%) and **4f** bearing the trifluoromethyl group (78%) are optimal at lower temperatures. Product **4e** containing the bromine is isolated in 89% yield.

**Figure 3:**
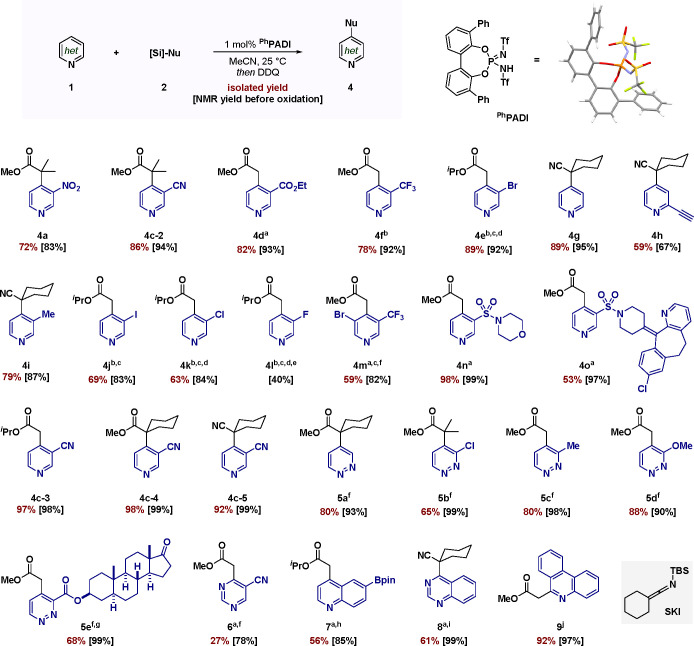
Application to a vast variety of *N*-heterocycles (isolated yields after *in situ* oxidation and addition step determined by ^1^H NMR in brackets). General conditions: reaction of 1 equiv of substrate with 2 equiv of SKA in MeCN using 1 mol % of ^**Ph**^**PADI** at 25 °C followed by DDQ (see SI for all the details). ^a^Reaction at 0 °C. ^b^Reaction at −20 °C. ^c^Use of ^**CF3**^**PADI**. ^d^Neat conditions. ^e^Addition before oxidation after 7 days. ^f^Oxidation with PIFA. ^g^Reaction in DCM. ^h^Oxidation with DIAD. ^i^Oxidation with KMnO_4_. ^j^Oxidation with Pd/C 10 mol %.

Perhaps even more impressively, the ^**Ph**^**PADI**-catalyzed synthesis of product **4g** has been accomplished in 89% yield, by direct installation of a new C–C bond onto unsubstituted pyridine itself. Thus, the use of a silyl ketene imine nucleophile (SKI) can leverage its lower stability to functionalize even less reactive substrates.^24^ It is also possible to furnish the sterically demanding *ortho*-substituted product **4h** bearing a geometrically linear group such as an alkyne in satisfactory yield (59%). Substrate **4i** with an alkyl group at the 3-position is formed in good yield (79%).

Unlike transition-metal catalyzed processes, this method tolerates sensitive halogen functionalities such as iodine or chlorine. Here,^**CF3**^**PADI** outperformed ^**Ph**^**PADI** with products **4j** (83% of addition instead of 68%) and **4k** (84% versus 67%). In contrast, fluorine-substituted product **4l** was obtained in only 40% yield after 7 days. In spite of its electronegativity, fluorine is known to engage in π-backdonation due to a shorter C–X bond, which increases the electron density of the aromatic ring and therefore decreases the reactivity.^25^ The catalyst also succeeds with more congested substitution patterns; trisubstituted product **4m** is formed in 59% yield. In this case, the challenging oxidation occurs more efficiently when using PIFA.

The sulfonamide moiety of substrate **1n** remains intact upon treatment with SKA **2b** and then with DDQ (98%). Remarkably, highly functionalized product **4o**—which contains the antihistaminic desloratadine—illustrates an outstanding selectivity between electronically distinct pyridines (97% of addition, 53% isolated after oxidation), which suggests that our method is even suitable for late stage diversification of complex bioactive molecules. Product **4c-3** is obtained when using **2c** (97%), **4c-4** when using a cyclic SKA (98%), and **4c-5** with the silyl ketene imine (92%).

The new method is also highly effective when applied to diverse azines. For instance, pyridazine **5a** is formed with excellent yield and regioselectivity (80%). Remarkably, functionalization toward product **5b**—which contains a good leaving group at an activated position, comparable to the Vilsmeier–Haack intermediate—also occurs very efficiently (99% of addition, 65% isolated). Substrates containing electron-rich groups perform greatly as well (**5c**, 80% and **5d**, 88%). Product **5e**—with neurosteroid epiandrosterone—displays the impressive orthogonal selectivity of the new catalyst in the presence of a ketone moiety (68%). We hypothesized that in these cases the regioselectivity is determined by the catalyst coordination to the less sterically hindered nitrogen atom. Besides, pyrimidines such as **6** can also be functionalized (78% of addition, 27% isolated after oxidation). Fused rings such as quinoline **7** bearing a labile boronic ester (56%) or quinazoline **8** (61%) are tolerated substrates as well. Alternative oxidants were required for these targets.^26^ The reaction can also occur with C2-selectivity in a highly reactive α-position when the C4-position is blocked and phenanthridine **9** is directly functionalized in an identical manner (92%). Otherwise, addition yields to *para*-substituted substrates are still rather low.

We have demonstrated the catalytic nucleophilic addition to azines via silylium activation (Figure 4A). Generation of the silylated catalyst precedes coordination of the substrate, which can then react with SKA **2** rapidly closing the catalytic cycle. We finally envisioned further diversification of **3** toward more elaborated scaffolds in a versatile approach. For instance, we conceived the direct assembly of dihydropyridine derivatives such as **10** upon subsequent reaction with an electrophile (Figure 4B). Combination of an acyl chloride with TBAF indeed forms the desired product quantitatively in a one-pot fashion.

**Figure 4:**
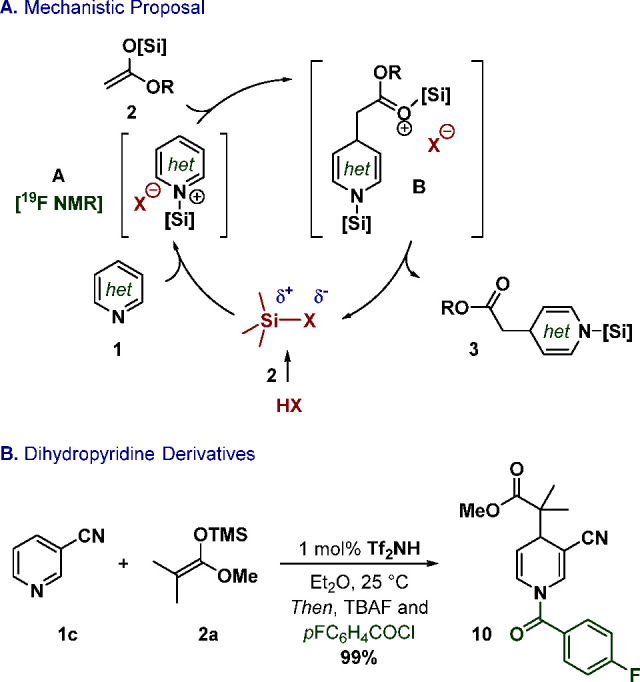
(A) Mechanistic proposal. (B) Synthesis of dihydropyridine derivatives.

In summary, we report an unprecedented silylium-catalyzed, one-pot functionalization of azines with complete C4-regioselectivity that requires no preactivation of the substrate. Thorough examination of the novel reactivity revealed a crucial dependence on the acidity of the catalyst alongside confinement to increase the chemoselectivity. The design presented here features exceptional electrophilicity, allowing the method to proceed efficiently for a great variety of scaffolds and orthogonally to numerous functional groups. Facile access to dihydropyridine derivatives is unlocked when our process is combined with an *in situ* reaction with an electrophile.

## Supporting Information Available

Experimental procedures and analytical data for all new compounds (PDF)

## Accession Codes

CCDC 2055772 and 2055774 contain the supplementary crystallographic data for this paper. These data can be obtained free of charge via www.ccdc.cam.ac.uk/data_request/cif, or by emailing data_request@ccdc.cam.ac.uk, or by contacting The Cambridge Crystallographic Data Centre, 12 Union Road, Cambridge CB2 1EZ, UK; fax: +44 1223 336033.
