# Supplementary material for: Azine Activation via Silylium Catalysis
Source: J Am Chem Soc. 2021 Apr 28;143(18):6817–22. doi: 10.1021/jacs.1c03257 (PMC8154516; doi:10.1021/jacs.1c03257)
Supplement: Supplementary file 1 — ja1c03257_si_001.pdf [file ja1c03257_si_001.pdf]

Supplementary Information

Azine Activation *via* Silylium Catalysis

Carla Obradors and Benjamin List*

Table of Contents

1. Materials and Methods 2
2. General Procedures 4
3. Dihydropyridine Derivatives 12
4. Synthesis of Catalysts 13
5. Synthesis of Substrates 20
6. Optimization Studies 22
7. Mechanistic Insights 26
8. References 29
9. Spectroscopic Data 30
10. HPLC Data 77
11. Crystallographic Data 79

1. Materials and Methods

Unless otherwise stated, all reactions were magnetically stirred and conducted in oven-dried (90 °C) or flame-dried glassware in anhydrous solvents under Ar, applying standard Schlenk techniques. Solvents and liquid reagents, as well as solutions of solid or liquid reagents were added via syringes, stainless steel or polyethylene cannulas through rubber septa or through a weak Ar counter-flow. Solid reagents were added through a weak Ar counter-flow. Cooling baths were prepared in Dewar vessels, filled with ice/water (0 °C), cooled acetone (> –78 °C) or dry ice/acetone (–78 °C). Heated oil baths were used for reactions requiring elevated temperatures. Solvents were removed under reduced pressure at 40 °C using a rotary evaporator, and unless otherwise stated, the remaining compound was dried in high vacuum (10^{-3} mbar) at ambient temperature. All given yields are isolated yields of chromatographically and NMR spectroscopically pure materials (95%), unless otherwise stated.

Chemicals
Chemicals were purchased from commercial suppliers (including abcr, Acros, Alfa Aesar, Fluorochem, Merck, and TCI) and used without further purification unless otherwise stated. Et_3N was distilled from $LiAlH_4$ and stored under argon prior to use. Pyridine was dried and stored over molecular sieves. *N*-Trifluoromethylsulfonyl-*P*,*P*,*P*-trichlorophosphazene and DSI catalysts were prepared according to literature procedures.[1,2]

Solvents
Solvents (CH_2Cl_2, Et_2O, THF, toluene) were dried by distillation from an appropriate drying agent in the technical department of the Max-Planck-Institut für Kohlenforschung and received in Schlenk flasks under argon.[3] Other anhydrous solvents (including acetone, benzene, $CHCl_3$, chlorobenzene, cyclohexane, 1,4-dioxane, DME, DMF, DMSO, EtOAc, EtOH, MeCN, MeOH, MTBE, NMP, *n*-hexane and *n*-pentane) were purchased from commercial suppliers and used as received.

Inert Gas
Dry argon was purchased from Air Liquide with >99.5% purity.

Thin Layer Chromatography
Thin-layer chromatography (TLC) was performed using silica gel pre-coated glass plates (SIL G-25, with fluorescent indicator UV_{254}; Macherey-Nagel) and aluminium oxide pre-coated plastic sheets (Polygram AlOx N, 0.2 mm, with fluorescent indicator UV_{254}; Macherey-Nagel), which were visualized by irradiation with UV light (λ = 254 or 366 nm), *p*-anisaldehyde, basic $KMnO_4$, and/or phosphomolybdic acid (PMA). PMA stain: PMA (20 g) in EtOH (200 mL), $KMnO_4$ stain: aq NaOH (10 wt%, 1.25 mL), $KMnO_4$ (1.5 g), K_2CO_3(10 g) in H_2O (200 mL), *p*-anisaldehyde stain: *p*-anisaldehyde (1 mL) in HOAc (20 mL), MeOH (170 mL), and concentrated H_2SO_4 (10 mL). Preparative thin-layer chromatography was performed on silica gel pre-coated glass plates SIL G-100, with fluorescent indicator UV_{254} (Macherey-Nagel).

Column Chromatography
Column chromatography was carried out using Merck silica gel (60 Å, 230−400 mesh, particle size 0.040−0.063 mm) or aluminum oxide (neutral, activated, Brockmann I, Sigma-Aldrich; activity adjustment individually specified) using technical grade solvents. Elution was accelerated using compressed air. Automated column chromatography was conducted on a Biotage® Isolera™ ISO-4SW instrument, using SNAP Ultra HP-Sphere™ 25 μm chromatography cartridges. All fractions containing a desired substance were combined and concentrated in vacuo, then dissolved in an appropriate solvent and filtered through cotton to remove silica residues.

Nomenclature
Nomenclature follows the suggestions proposed by the computer program ChemDraw Professional 15.0 of PerkinElmer®.

Melting Points
Melting points (m.p.) were measured on a Büchi 540 melting point apparatus in open glass capillaries and are uncorrected.

Nuclear Magnetic Resonance Spectroscopy
^{1}H, ^{13}C, ^{11}B, ^{19}F, ^{31}P nuclear magnetic resonance (NMR) spectra were recorded on a Bruker AV-500, AV-400 or DPX-300 spectrometer in a suitable deuterated solvent. The solvent employed and respective measuring frequencies are indicated for each experiment. Chemical shifts are reported with Me_4Si serving as a universal reference of all nuclides and with two or one digits after the comma. The resonance multiplicity is described as s (singlet), d (doublet), t (triplet), q (quadruplet), p (pentet), hept (heptet), m (multiplet) and br (broad). All spectra were recorded at 298 K unless otherwise noted, processed with the program MestReNova 11.0, and coupling constants are reported as observed. The residual deuterated solvent signal relative to Me_4Si was used as the internal reference in ^{1}H NMR spectra (e.g. $CDCl_3$ = 7.26 ppm)[4,5] and are reported as follows: chemical shift in ppm (multiplicity, coupling constant *J* in Hz, number of protons). ^{11}B, ^{13}C, ^{19}F, ^{31}P NMR spectra were referenced according to Ξ-values (IUPAC recommendations 2008)[6] relative to the internal references set in ^{1}H NMR spectra (e.g. ^{13}C: Me_4Si, ^{19}F: CCl_3F, ^{31}P: H_3PO_4 each 0.00 ppm). All spectra are broadband decoupled unless otherwise noted.

Mass Spectrometry
Electron impact (EI) mass spectrometry (MS) was performed on a Finnigan MAT 8200 (70 eV) or MAT 8400 (70 eV) spectrometer. Electrospray ionization (ESI) mass spectrometry was conducted on a Bruker ESQ 3000 spectrometer. High resolution mass spectrometry (HRMS) was performed on a Finnigan MAT 95 (EI) or Bruker APEX III FTMS (7T magnet, ESI). The ionization method and mode of detection employed is indicated for the respective experiment and all masses are reported in atomic units per elementary charge (m/z) with an intensity normalized to the most intense peak.

Specific Rotations
Specific rotations $[\alpha]_D$ were measured with a Rudolph RA Autopol IV Automatic Polarimeter at the indicated temperature (T) with a sodium lamp (sodium D line, λ = 589 nm). Measurements were performed in an acid resistant 1 mL cell (50 mm length) with concentrations (g/100 mL) reported in the corresponding solvent.

Liquid Chromatography-Mass Spectrometry
Liquid chromatography-mass spectrometry (LC-MS) was performed on Shimadzu LC-MS 2020 liquid chromatograph. All solvents used were HPLC-grade solvents purchased from Sigma-Aldrich. The column employed, the respective solvent mixture, and the MS parameters are indicated for each experiment.

High Performance Liquid Chromatography
High-performance liquid chromatography (HPLC) was performed on Shimadzu LC-20AD liquid chromatograph (SIL-20AC auto sampler, CMB-20A communication bus module, DGU-20A5 degasser, CTO-20AC column oven, SPD-M20A diode array detector), Shimadzu LC-20AB liquid chromatograph (SIL-20ACHT auto sampler, DGU-20A5 degasser, CTO-20AC column oven, SPD-M20A diode array detector), or Shimadzu LC-20AB liquid chromatograph (reversed phase, SIL-20ACHT auto sampler, CTO-20AC column oven, SPD-M20A diode array detector) using Daicel columns with chiral stationary phases. All solvents used were HPLC-grade solvents, purchased from Merck. The column employed and respective solvent mixture are indicated for each experiment.

2. General Procedures

Oxidative quenching using 2,3-dichloro-5,6-dicyano-1,4-benzoquinone (DDQ)

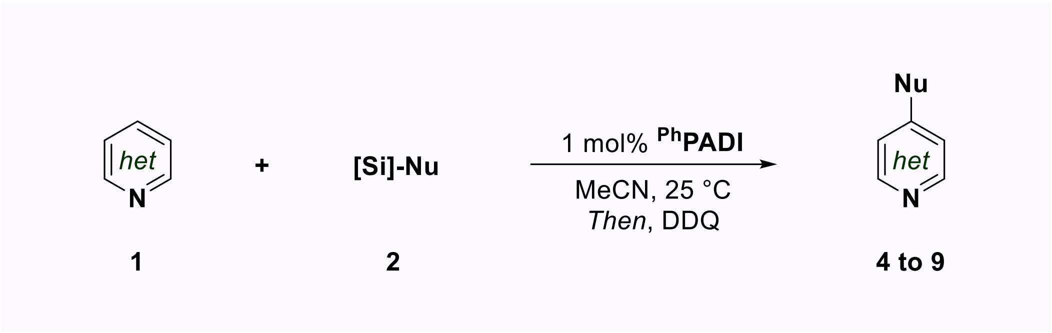

Weigh the catalyst (1 mol %) and the substrate if solid (0.2 mmol) in a vial charged with a magnetic stirring bar and place it under argon atmosphere. Unless stated otherwise, add the corresponding silyl ketene acetal (2 equiv) *via* Hamilton syringe followed by MeCN (5 M) and the substrate if liquid. Then, seal with Teflon/parafilm and stir the mixture overnight at the specified temperature. Check the crude by ^{1}H NMR in order to determine the conversion of the addition step (shown in parenthesis). Alternatively, quench the reaction dropwise with dry DDQ (1 M in MeCN) and stir 3 h at room temperature. Add triethylamine (1 equiv) and filter the crude through a long pad of silica eluting with DCM/MeOH (1%). Purify the desired product using chromatography techniques.

Methyl 2-methyl-2-(3-nitropyridin-4-yl)propanoate (**4a**)

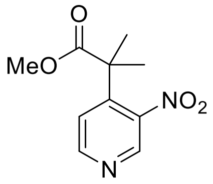

General procedure with **1a** and **2a** performed at room temperature (83%). Quench with 2 equiv DDQ (*Note: 20 h*). Purify by prep-TLC using MTBE as eluent (Rf = 0.65). The desired product **4a** was obtained as a pale yellow powder (32.1 mg, **72% yield**). ^{1}H NMR (500 MHz, CD_2Cl_2) δ 9.06 (s, 1H), 8.78 (d, *J* = 5.3 Hz, 1H), 7.53 (d, *J* = 5.3 Hz, 1H), 3.60 (s, 3H), 1.64 (s, 6H). ^{13}C NMR (126 MHz, CD_2Cl_2) δ 174.8, 154.4, 148.4, 146.7, 145.4, 122.7, 52.6, 46.6, 27.0. HRMS (ESI$^+$) calculated for $C_{10}H_{12}N_2O_4Na^+$ [M+Na$^+$] 247.0689, found 247.0690. Mp = 72.8 – 75.1 °C.

Methyl 2-(3-cyanopyridin-4-yl)acetate (**4c-1**)

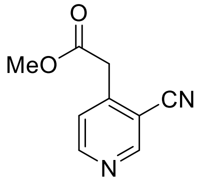

General procedure with **1c** and **2b** performed at room temperature (99%). Quench with 1.2 equiv DDQ. Purify by prep-TLC using MTBE as eluent (Rf = 0.43). The desired product **4c-1** was obtained as a dark yellow oil (32.6 mg, **93% yield**). ^{1}H NMR (500 MHz, CD_2Cl_2) δ 8.85 (s, 1H), 8.83 (d, *J* = 5.2 Hz, 1H), 7.41 (d, *J* = 5.2, 1H), 3.88 (s, 2H), 3.74 (s, 3H). ^{13}C NMR (126 MHz, CD_2Cl_2) δ 169.3, 153.5, 153.4, 146.7, 125.5, 116.0, 111.7, 53.1, 39.4. HRMS (CI$^+$) calculated for $C_9H_9N_2O_2^+$ [M+H$^+$] 177.0660, found 177.0659.

Methyl 2-(3-cyanopyridin-4-yl)-2-methylpropanoate (**4c-2**)

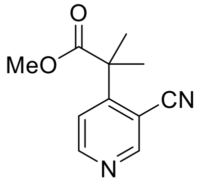

General procedure with **1c** and **2a** performed at room temperature (94%). Quench with 2 equiv DDQ. *Note: extraction with MTBE/sat. K_2CO_3 instead of silica filtration and dry the combined organic layers with Na_2SO_4.* Purify by column chromatography using MTBE/hexanes 1:1 as eluent (Rf (MTBE) = 0.68). The desired product **4c-2** was obtained as a pale yellow powder (35 mg, **86% yield**). ^{1}H NMR (500 MHz, $CDCl_3$) δ 8.81 (s, 1H), 8.74 (d, *J* = 5.4 Hz, 1H), 7.38 (d, *J* = 5.4 Hz, 1H), 3.75 (s, 3H), 1.66 (s, 6H). ^{13}C NMR (126 MHz, $CDCl_3$) δ 174.8, 156.5, 153.9, 153.3, 120.6, 115.9, 109.5, 52.8, 47.2, 25.8. HRMS (ESI^+) calculated for $C_{11}H_{12}N_2O_2Na^+$ [$M+Na^+$] 227.0791, found 227.0792. Mp = 53.4 – 55.8 °C.

Isopropyl 2-(3-cyanopyridin-4-yl)acetate (**4c-3**)

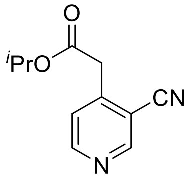

General procedure with **1c** and **2c** performed at room temperature (98%). Quench with 1.2 equiv DDQ. Purify by prep-TLC using MTBE as eluent (Rf = 0.64). The desired product **4c-3** was obtained as a yellow powder (39.5 mg, **97% yield**). ^{1}H NMR (500 MHz, CD_2Cl_2) δ 8.85 (s, 1H), 8.72 (d, *J* = 5.2 Hz, 1H), 7.39 (d, *J* = 5.5 Hz, 1H), 5.04 (hept, *J* = 6.2 Hz, 1H), 3.83 (s, 2H), 1.24 (d, *J* = 6.3 Hz, 6H). ^{13}C NMR (126 MHz, CD_2Cl_2) δ 168.3, 153.5, 153.4, 147.1, 125.4, 116.1, 111.7, 70.1, 40.0, 21.9. HRMS (EI^-) calculated for $C_{11}H_{12}N_2O_2^-$ [M^-] 204.0893, found 204.0893. Mp = 75.1 – 76.8 °C.

Methyl 1-(3-cyanopyridin-4-yl)cyclohexane-1-carboxylate (**4c-4**)

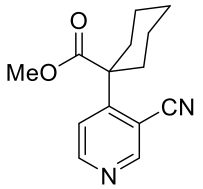

General procedure with **1c** and (cyclohexylidene(methoxy)methoxy)trimethylsilane (**S-1**) performed at room temperature (99%).[7] Quench with 2 equiv DDQ. *Note: extraction with MTBE/sat. K_2CO_3 instead of silica filtration and dry the combined organic layers with Na_2SO_4.* Purify by column chromatography using MTBE/hexanes 1:1 as eluent (Rf (MTBE) = 0.71). The desired product **4c-4** was obtained as a yellow oil (48.1 mg, **98% yield**). ^{1}H NMR (500 Mz, $CDCl_3$) δ 8.81 (s, 1H), 8.71 (d, *J* = 5.5 Hz, 1H), 7.39 (d, *J* = 5.5 Hz, 1H), 3.73 (s, 3H), 2.45 – 2.41 (m, 2H), 2.05 – 1.99 (m, 2H), 1.68 – 1.58 (m, 5H), 1.41 – 1.35 (m, 1H). ^{13}C NMR (126 MHz, $CDCl_3$) δ 173.5, 155.6, 155.1, 153.1, 122.1, 116.5, 109.1, 52.6, 52.0, 33.5, 25.1, 22.7. HRMS (ESI^+) calculated for $C_{14}H_{16}N_2O_2Na^+$ [$M+Na^+$] 267.1104, found 267.1105.

4-(1-Cyanocyclohexyl)nicotinonitrile (**4c-5**)

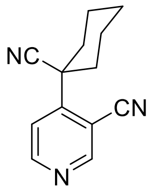

General procedure with **1c** and *N*-(*tert*-butyldimethylsilyl)-1-cyclohexylidenemethanimine (**S-2**) performed at room temperature (99%).[8] Quench with 2 equiv DDQ. *Note: extraction with MTBE/sat. K_2CO_3 instead of silica filtration and dry the combined organic layers with Na_2SO_4.* Purify by column using MTBE/hexanes 1:1 as eluent (Rf (MTBE) = 0.57). The desired product **4c-5** was obtained as a yellow solid (38.8 mg, **92% yield**). ^{1}H NMR (500 MHz, $CDCl_3$) δ 8.92 (s, 1H), 8.82 (d, *J* = 5.4 Hz, 1H), 7.71 (d, *J* = 5.4 Hz, 1H), 2.27 – 2.19 (m, 4H), 1.98 – 1.95 (m, 2H), 1.92 – 1.82 (m, 3H), 1.43 – 1.34 (m, 1H). ^{13}C NMR (126 MHz, $CDCl_3$) δ 155.4, 154.0, 151.7, 121.7, 119.6, 116.2, 107.9, 45.3, 34.5, 24.3, 23.3. HRMS (ESI^+) calculated for $C_{13}H_{13}N_3Na^+$ [$M+Na^+$] 234.1002, found 234.1003. Mp = 118.7 – 120.4 °C.

Ethyl 4-(2-methoxy-2-oxoethyl)nicotinate (**4d**)

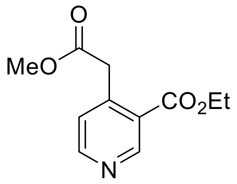

General procedure with **1d** and **2b** performed at 0 °C (93%). Quench with 1.2 equiv DDQ. Purify by prep-TLC using MTBE as eluent (Rf = 0.45). The desired product **4d** was obtained as a yellow oil (36.2 mg, **82% yield**). ^{1}H NMR (500 MHz, $CDCl_3$) δ 9.17 (s, 1H), 8.65 (d, *J* = 5.0 Hz, 1H), 7.19 (d, *J* = 5.0 Hz, 1H), 4.36 (q, *J* = 7.1 Hz, 2H), 4.02 (s, 2H), 3.70 (s, 3H), 1.38 (t, *J* = 7.1 Hz, 3H). ^{13}C NMR (126 MHz, $CDCl_3$) δ 170.5, 165.8, 152.8, 152.0, 144.6, 126.7, 126.0, 61.6, 52.3, 39.8, 14.2. HRMS (ESI$^+$) calculated for $C_{11}H_{14}N_1O_4^+$ [M+H$^+$] 224.0917, found 224.0918.

Isopropyl 2-(3-bromopyridin-4-yl)acetate (**4e**)

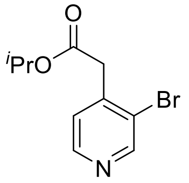

General procedure with **1e** and **2c** performed at –20 °C (92%). *Note: ^{CF3}PADI in neat conditions.* Quench with 1.2 equiv DDQ. Purify by prep-TLC using MTBE as eluent (Rf = 0.81). The desired product **4e** was obtained as a brown oil (46.1 mg, **89% yield**). ^{1}H NMR (500 MHz, CD_2Cl_2) δ 8.69 (s, 1H), 8.46 (d, *J* = 5.0 Hz, 1H), 7.26 (d, *J* = 4.9 Hz, 1H), 5.02 (hept, *J* = 6.2 Hz, 1H), 3.74 (s, 3H), 1.23 (d, *J* = 6.1 Hz, 6H). ^{13}C NMR (126 MHz, CD_2Cl_2) δ 168.7, 152.2, 148.7, 143.7, 126.6, 69.3, 41.6, 21.8. HRMS (ESI$^+$) calculated for $C_{10}H_{13}N_1O_2Br_1^+$ [M+H$^+$] 258.0124, found 258.0124.

Methyl 2-(3-(trifluoromethyl)pyridin-4-yl)acetate (**4f**)

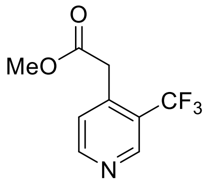

General procedure with **1f** and **2b** performed at –20 °C (92%). Quench with 1.2 equiv DDQ. Purify by prep-TLC using MTBE as eluent (Rf = 0.73). The desired product **4f** was obtained as a dark yellow oil (34.2 mg, **78% yield**). ^{1}H NMR (500 MHz, CD_2Cl_2) δ 8.86 (s, 1H), 8.75 (d, *J* = 5.0 Hz, 1H), 7.37 (d, *J* = 5.0 Hz, 1H), 3.84 (s, 2H), 3.70 (s, 3H). ^{13}C NMR (126 MHz, CD_2Cl_2) δ 169.8, 153.6, 147.5 (q, *J* = 6.0 Hz), 127.1, 127.0, 125.4, 125.3, 125.1, 123.2, 121.0, 52.7, 37.8. *Note: overlapping of signals observed.* ^{19}F NMR (471 MHz) δ – 60.32. HRMS (EI$^-$) calculated for $C_9H_8N_1O_2F_3^-$ [M$^-$] 219.0502, found 219.0504.

1-(Pyridin-4-yl)cyclohexane-1-carbonitrile (**4g**)

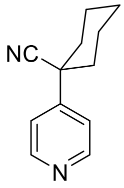

General procedure with **1g** and **S-2** performed at room temperature (95%). *Note: 10 M used.* Quench with 1 equiv DDQ. Purify by prep-TLC using MTBE as eluent (Rf = 0.46). The desired product **4g** was obtained as a yellow solid (33 mg, **89% yield**). ^{1}H NMR (500 MHz, CD_2Cl_2) δ 8.61 (br d, *J* = 4.8 Hz, 2H), 7.41 – 7.40 (m, 2H), 2.13 – 2.09 (m, 2H), 1.91 – 1.82 (m, 4H), 1.80 – 1.74 (m, 3H), 1.33 – 1.26 (m, 1H). ^{13}C NMR (126 MHz, CD_2Cl_2) δ 150.8, 150.5, 121.8, 121.0, 44.6, 37.0, 25.1, 23.7. HRMS (EI$^-$) calculated for $C_{12}H_{14}N^-$ [M$^-$] 186.1151, found 186.1151. Mp = 74.8 – 76.3 °C.

1-(2-Ethynylpyridin-4-yl)cyclohexane-1-carbonitrile (**4h**)

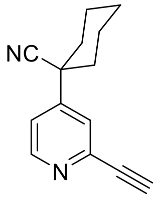

General procedure with **1h** and **S-2** performed at room temperature (67%). Quench with 1 equiv DDQ. Purify by prep-TLC using MTBE/hexanes 3:1 as eluent (Rf (MTBE) = 0.72). The desired product **4h** was obtained as a brown oil (24.7 mg, **59% yield**). ^{1}H NMR (500 MHz, CD_2Cl_2) δ 8.58 (d, *J* = 5.3 Hz, 1H), 7.60 (d, *J* = 2.1 Hz, 1H), 7.40 (dd, *J* = 5.3, 2.0 Hz, 1H), 3.23 (s, 1H), 2.17 – 2.05 (m, 2H), 1.93 – 1.81 (m, 4H), 1.80 – 1.70 (m, 3H), 1.37 – 1.23 (m, 1H). ^{13}C NMR (126 MHz, CD_2Cl_2) δ 151.1, 151.0, 143.3, 125.0, 121.4, 121.0, 82.9, 77.6, 44.6, 36.9, 25.0, 23.7. HRMS (ESI$^+$) calculated for $C_{14}H_{14}N_2^+$ [M+H$^+$] 211.1230, found 211.1231.

1-(3-Methylpyridin-4-yl)cyclohexane-1-carbonitrile (**4i**)

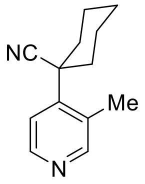

General procedure with **1i** and **S-2** performed at room temperature (87%). Quench with 1 equiv DDQ. Purify by prep-TLC using MTBE as eluent (Rf (MTBE) = 0.46). The desired product **4i** was obtained as a yellow oil (31.5 mg, **79% yield**). ^{1}H NMR (500 MHz, CD_2Cl_2) δ 8.43 (d, *J* = 5.4 Hz, 1H), 8.41 (s, 1H), 7.20 (d, *J* = 5.3 Hz, 1H), 2.60 (s, 3H), 2.41 – 2.26 (m, 2H), 1.97 – 1.80 (m, 5H), 1.76 – 1.65 (m, 2H), 1.35 – 1.22 (m, 1H). ^{13}C NMR (126 MHz, CD_2Cl_2) δ 153.29, 148.31, 146.78, 131.68, 121.04, 119.35, 41.64, 35.04, 24.94, 23.18, 17.84. HRMS (EI^-) calculated for $C_{13}H_{16}N_2^-$ [M^-] 200.1308, found 200.1309.

Isopropyl 2-(3-iodopyridin-4-yl)acetate (**4j**)

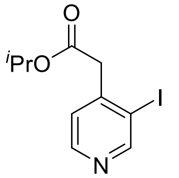

General procedure with **1j** and **2c** performed at −20 °C (83%). *Note: ^{CF3}PADI used.* Quench with 1.2 equiv DDQ. Purify by prep-TLC using MTBE/hexanes 1:2 as eluent (Rf = 0.35). The desired product **4j** was obtained as a yellow oil (42 mg, **69% yield**). ^{1}H NMR (500 MHz, CD_2Cl_2) δ 8.91 (s, 1H), 8.45 (d, *J* = 4.9 Hz, 1H), 7.26 (d, *J* = 4.9 Hz, 1H), 5.03 (hept, *J* = 6.2 Hz, 1H), 3.72 (s, 2H), 1.24 (d, *J* = 6.3 Hz, 6H). ^{13}C NMR (126 MHz, CD_2Cl_2) δ 168.7, 157.9, 149.4, 147.1, 126.3, 100.8, 69.3, 46.0, 21.8. HRMS (GC-CI) calculated for $C_{10}H_{13}N_1O_2I_1^+$ [$M+H^+$] 305.9986, found 305.9989.

Isopropyl 2-(3-chloropyridin-4-yl)acetate (**4k**)

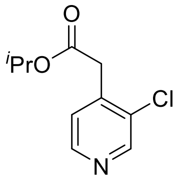

General procedure with **1k** and **2c** performed at –20 °C (84%). *Note: ^{CF3}PADI in neat conditions.* Quench with 1.2 equiv DDQ. Purify by prep-TLC using MTBE/hexanes 1:3 as eluent (Rf (MTBE) = 0.79). The desired product **4k** was obtained as a yellow oil (27 mg, **63% yield**). ^{1}H NMR (500 MHz, CD_2Cl_2) δ 8.56 (s, 1H), 8.42 (d, *J* = 4.9 Hz, 1H), 7.25 (d, *J* = 4.8 Hz, 1H), 5.02 (hept, *J* = 6.3 Hz, 1H), 3.73 (s, 2H), 1.23 (d, *J* = 6.2 Hz, 6H). ^{13}C NMR (126 MHz, CD_2Cl_2) δ 168.8, 149.7, 148.2, 141.7, 126.2, 69.3, 39.0, 21.8. HRMS (ESI^+) calculated for $C_{10}H_{13}N_1O_2Cl_1^+$ [$M+H^+$] 214.0629, found 214.0631.

Methyl 2-(3-(morpholinosulfonyl)pyridin-4-yl)acetate (**4n**)

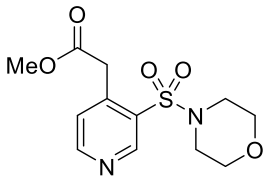

General procedure with 4-(pyridin-3-ylsulfonyl)morpholine and **2b** performed at 0 °C (99%).[9] *Note: 2.5 M used.* Quench with 1.2 equiv DDQ. Purify by prep-TLC using MTBE as eluent (Rf = 0.31). The desired product **4n** was obtained as a yellow paste (59 mg, **98% yield**). ^{1}H NMR (500 MHz, $CDCl_3$) δ 9.02 (s, 1H), 8.72 (d, *J* = 5.0 Hz, 1H), 7.34 (d, *J* = 5.2 Hz, 1H), 4.05 (s, 2H), 3.70 (s, 3H), 3.69 – 3.67 (m, 4H), 3.15 – 3.13 (m, 4H). ^{13}C NMR (126 MHz, $CDCl_3$) δ 169.9, 153.6, 150.6, 143.0, 132.5, 127.6, 66.1, 52.5, 45.2, 37.9. HRMS (ESI^+) calculated for $C_{12}H_{16}N_2O_5S_1Na_1^+$ [$M+Na^+$] 323.0672, found 323.0673.

Methyl 2-(3-((4-(8-chloro-5,6-dihydro-11*H*-benzo[5,6]cyclohepta[1,2-*b*]pyridin-11-ylidene)piperidin-1-yl)sulfonyl)pyridin-4-yl)acetate (**4o**)

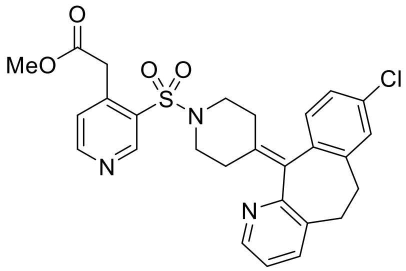

General procedure with **1o** and **2b** performed at 0 °C (97%). *Note: 5 equiv of SKA and 1 M used at 0.05 mmol scale.* Quench with 2.4 equiv DDQ (Rf (MTBE) = 0.19). The desired product **4o** was obtained as a yellow foam after filtration (13.4 mg, **53% yield**). ^1H NMR (500 MHz, $CDCl_3$) δ 9.03 (s, 1H), 8.73 (d, J = 5.0 Hz, 1H), 8.37 (dd, J = 4.7, 1.7 Hz, 1H), 7.43 (dd, J = 7.7, 1.7 Hz, 1H), 7.34 (d, J = 5.1 Hz, 1H), 7.16 (d, J = 2.2 Hz, 1H), 7.14 – 7.08 (m, 2H), 7.05 (d, J = 8.2 Hz, 1H), 4.10 (d, J = 16.9 Hz, 1H), 4.05 (d, J = 16.9 Hz, 1H), 3.73 (s, 3H), 3.51 – 3.39 (m, 2H), 3.32 (dddd, J = 19.4, 11.3, 7.7, 3.6 Hz, 2H), 3.06 (ddt, J = 12.4, 8.6, 4.0 Hz, 2H), 2.90 – 2.73 (m, 2H), 2.58 (ddd, J = 13.7, 8.9, 4.3 Hz, 1H), 2.51 – 2.36 (m, 3H). ^{13}C NMR (126 MHz, $CDCl_3$) δ 170.0, 156.5, 153.5, 150.4, 146.8, 142.8, 139.6, 137.8, 137.4, 135.5, 135.4, 133.6, 133.5, 133.3, 130.4, 129.1, 127.5, 126.3, 122.6, 52.6, 46.7, 38.1, 31.7, 31.6, 30.5, 30.3. HRMS (ESI$^+$) calculated for $C_{27}H_{27}N_3O_4S_1Cl_1^+$ [M+H$^+$] 524.1405, found 524.1408. *Note: melting point measurements inconclusive, potential polymorphism.*

Oxidative quenching using (bis(trifluoroacetoxy)iodo)benzene (PIFA)

The addition step is independent on the oxidant used for the rearomatization of **3**. Quench the reaction *via* dilution of the crude with DCM followed by slow addition of PIFA. Keep stirring at room temperature and add triethylamine (1 equiv). Purify the desired product using chromatography techniques.

Methyl 2-(3-bromo-5-(trifluoromethyl)pyridin-4-yl)acetate (**4m**)

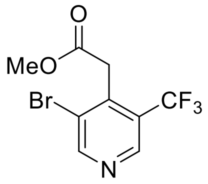

General procedure with **1m** and **2b** performed at 0 °C (82%). *Note: ^{CF3}PADI and 2.5 M used at 0.1 mmol scale.* Quench for 2 h with 2 equiv PIFA (0.2 M). In this case, filter the crude through a long pad of silica eluting with DCM/MeOH (1%). Then, purify by prep-TLC using MTBE/hexanes 1:4 as eluent (Rf = 0.63). The desired product **4m** was obtained as a yellow oil (17.5 mg, **59% yield**). ^1H NMR (600 MHz, CD_2Cl_2) δ 8.93 (s, 1H), 8.80 (s, 1H), 4.03 (s, 2H), 3.71 (s, 3H). ^{13}C NMR (151 MHz, CD_2Cl_2) δ 168.4, 156.0 (q, J = 1.3 Hz), 146.1 (q, J = 6.3 Hz), 142.0 (q, J = 1.5 Hz), 126.9 (q, J = 30.3 Hz), 126.2, 123.5 (q, J = 274.6 Hz), 52.9, 38.1 (q, J = 1.9 Hz). ^{19}F NMR (565 MHz, CD_2Cl_2) δ – 60.32. HRMS (ESI$^+$) calculated for $C_9H_8N_1O_2Br_1F_3^+$ [M+H$^+$] 297.9685, found 297.9686.

Methyl 1-(pyridazin-4-yl)cyclohexane-1-carboxylate (**5a**)

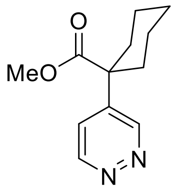

General procedure with pyridazine and **S-1** performed at room temperature (93%). Quench overnight with 2 equiv PIFA (0.2 M). In this case, extract with DCM/sat. $NaHCO_3$ and dry the combined organic layers with Na_2SO_4. Purify by prep-TLC using MTBE as eluent (Rf = 0.33). The desired product **5a** was obtained as a pale yellow solid (35 mg, **80% yield**). ^1H NMR (500 MHz, CD_2Cl_2) δ 9.21 (d, J = 1.4 Hz, 1H), 9.08 (d, J = 5.5 Hz, 1H), 7.42 (dd, J = 5.5, 2.6 Hz, 1H), 3.65 (s, 3H), 2.45 – 2.42 (m, 2H), 1.80 – 1.75 (m, 2H), 1.66 – 1.60 (m, 3H), 1.54 – 1.46 (m, 2H), 1.36 – 1.28 (m, 1H). ^{13}C NMR (126 MHz, CD_2Cl_2) δ 174.0, 151.6, 150.8, 143.2, 124.3, 52.9, 50.0, 34.0, 25.7, 23.6. HRMS (ESI$^+$) calculated for $C_{12}H_{16}N_2O_2Na^+$ [M+Na$^+$] 243.1104, found 243.1105. Mp = 57.5 – 59.8 °C.

Methyl 2-(3-chloropyridazin-4-yl)-2-methylpropanoate (**5b**)

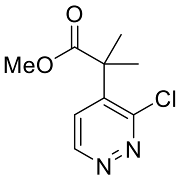

General procedure with 3-chloropyridazine and **2a** performed at rt (99%). Quench for 1 h with 1.5 equiv PIFA (0.1 M). Purify by prep-TLC using MTBE as eluent (Rf = 0.47). The desired product **5b** was obtained as a dark yellow oil (28 mg, **65% yield**). ^{1}H NMR (500 MHz, CD_2Cl_2) δ 9.08 (d, *J* = 5.1 Hz, 1H), 7.50 (d, *J* = 5.2 Hz, 1H), 3.67 (s, 3H), 1.61 (s, 6H). ^{13}C NMR (151 MHz, CD_2Cl_2) δ 175.0, 157.4, 151.3, 144.5, 125.8, 53.0, 46.3, 24.9. HRMS (EI$^-$) calculated for $C_9H_{11}N_2O_2Cl_1^-$ [M$^-$] 214.0504, found 214.0504.

Methyl 2-(3-methylpyridazin-4-yl)acetate (**5c**)

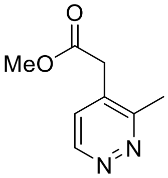

General procedure with 3-methylpyridazine and **2b** performed at rt (98%). Quench for 1 h with 1.2 equiv PIFA (0.05 M). Purify by prep-TLC using DCM with 3% MeOH as eluent (Rf = 0.21). The desired product **5c** was obtained as an orange oil (26.7 mg, **80% yield**). ^{1}H NMR (500 MHz, CD_2Cl_2) δ 8.96 (d, *J* = 5.1 Hz, 1H), 7.28 (d, *J* = 5.0 Hz, 1H), 3.70 (s, 3H), 3.65 (s, 2H), 2.66 (s, 3H). ^{13}C NMR (151 MHz, CD_2Cl_2) δ 169.8, 160.3, 150.2, 133.2, 127.4, 52.7, 37.8, 20.4. HRMS (EI$^-$) calculated for $C_8H_{10}N_2O_2^-$ [M$^-$] 166.0737, found 166.0739.

Methyl 2-(3-methoxypyridazin-4-yl)acetate (**5d**)

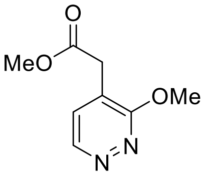

General procedure with 3-methoxypyridazine and **2b** performed at rt (90%). Quench for 1 h with 1.2 equiv PIFA (0.05 M). Purify by prep-TLC using DCM with 3% MeOH as eluent (Rf = 0.26). The desired product **5d** was obtained as an orange oil (32.3 mg, **88% yield**). ^{1}H NMR (500 MHz, CD_2Cl_2) δ 8.76 (d, *J* = 4.6 Hz, 1H), 7.33 (d, *J* = 4.7 Hz, 1H), 4.11 (s, 3H), 3.69 (s, 3H), 3.62 (s, 2H). ^{13}C NMR (151 MHz, CD_2Cl_2) δ 169.8, 160.3, 150.2, 133.2, 127.4, 52.7, 37.8, 20.4. HRMS (GC-EI) calculated for $C_8H_{10}N_2O_3^-$ [M$^-$] 182.0689, found 182.0686.

(3*S*,5*S*,8*R*,9*S*,10*S*,13*S*,14*S*)-10,13-Dimethyl-17-oxohexadecahydro-1*H*-cyclopenta[*a*]phenanthren-3-yl 4-(2-methoxy-2-oxoethyl)pyridazine-3-carboxylate (**5e**)

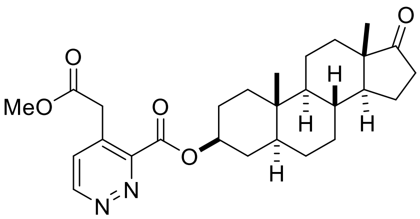

General procedure with **S-3** and **2b** performed at rt (99%). *Note: DCM used at 0.05 mmol scale.* Quench for 1 h with 1.2 equiv PIFA (0.05 M). Purify by prep-TLC using hexanes/ethyl acetate 1:3 as eluent (Rf = 0.30). The desired product **5e** was obtained as a pale yellow powder (15 mg, **68% yield**). ^{1}H NMR (500 MHz, CD_2Cl_2) δ 9.19 (d, *J* = 5.2 Hz, 1H), 7.44 (d, *J* = 5.3 Hz, 1H), 5.03 (tt, *J* = 11.4, 5.0 Hz, 1H), 3.96 (s, 2H), 3.69 (s, 3H), 2.42 – 2.37 (m, 1H), 2.06 – 1.98 (m, 2H), 1.95 – 1.90 (m, 1H), 1.85 – 1.79 (m, 2H), 1.77 – 1.65 (m, 3H), 1.62 – 1.55 (m, 3H), 1.51 (ddd, *J* = 12.4, 9.0, 3.4 Hz, 1H), 1.39 – 1.36 (m, 2H), 1.34 – 1.26 (m, 3H), 1.25 – 1.20 (m, 1H), 1.13 (td, *J* = 13.5, 3.9 Hz, 1H), 1.06 – 1.00 (m, 1H), 0.91 (s, 3H), 0.85 (s, 3H), 0.77 (ddd, *J* = 12.3, 10.5, 4.2 Hz, 1H). ^{13}C NMR (151 MHz, CD_2Cl_2) 220.9, 169.8, 164.8, 153.1, 152.6, 135.0, 129.6, 76.4, 54.7, 52.7, 51.8, 48.0, 45.1, 38.5, 37.1, 36.1, 36.1, 35.4, 34.1, 32.0, 31.2, 28.7, 27.6, 22.1, 20.9, 14.0, 12.4. HRMS (DIP-EI) calculated for $C_{27}H_{36}N_2O_5^-$ [M$^-$] 468.2619, found 468.2615. Mp = 168.1 – 172.9 °C.

Methyl 2-(5-cyanopyrimidin-4-yl)acetate (**6**)

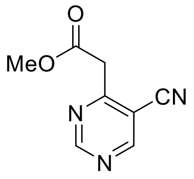

General procedure with pyrimidine-5-carbonitrile and **2b** performed at 0 °C (78%). *Note: ^{CF3}PADI and 1.25 M used.* Quench for 3 h with 1.2 equiv PIFA (0.05 M). Purify by prep-TLC using hexanes/MTBE 2:1 as eluent (Rf = 0.18). The desired product **6** was obtained as a bright yellow paste (9.7 mg, **27% yield**). ^{1}H NMR (500 MHz, CD_2Cl_2) δ 9.30 (s, 1H), 8.99 (s, 1H), 4.07 (s, 2H), 3.75 (s, 3H). ^{13}C NMR (151 MHz, CD_2Cl_2) δ 168.2, 166.1, 160.5, 160.5, 114.4, 110.6, 53.0, 42.6. HRMS (ESI$^+$) calculated for $C_8H_7N_3O_2Na_1^+$ [M+Na$^+$] 200.0430, found 200.0433.

Oxidative quenching using diisopropyl azodicarboxylate (DIAD)

The addition step is independent on the oxidant used for the rearomatization of **3**. Quench the reaction *via* dilution of the crude with DCM followed by addition of DIAD and keep stirring at room temperature. Purify the desired product using chromatography techniques.

Isopropyl 2-(6-(4,4,5,5-tetramethyl-1,3,2-dioxaborolan-2-yl)quinolin-4-yl)acetate (**7**)

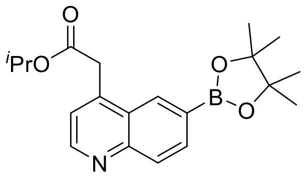

General procedure with 6-(4,4,5,5-tetramethyl-1,3,2-dioxaborolan-2-yl)quinoline and **2c** performed at 0 °C (85%). *Note: 0.1 mmol scale.* Quench for 4 h with 1.5 equiv DIAD (0.5 M). Purify by column chromatography using hexanes/MTBE 3:1 as eluent (Rf (MTBE) = 0.77). The desired product **7** was obtained as a pale yellow powder (20.2 mg, **56% yield**). ^{1}H NMR (500 MHz, CD_2Cl_2) δ 8.86 (d, *J* = 4.4 Hz, 1H), 8.47 (s, 1H), 8.07 (d, *J* = 8.3 Hz, 1H), 8.03 (dd, *J* = 8.4, 1.3 Hz, 1H), 7.34 (d, *J* = 4.4 Hz, 1H), 5.03 (hept, *J* = 6.3 Hz, 1H), 4.09 (s, 2H), 1.38 (s, 12H), 1.24 (d, *J* = 6.3 Hz, 6H). ^{13}C NMR (151 MHz, CD_2Cl_2) δ 170.1, 151.5, 150.2, 141.6, 134.2, 132.1, 129.5, 127.2, 123.0, 84.6, 69.2, 39.0, 25.1, 21.8. *Note: C6 not resolved due to coupling with the boron atom.* ^{11}B NMR (161 MHz, CD_2Cl_2) δ 31.12. HRMS (EI$^-$) calculated for $C_{20}H_{26}N_1O_4B_1^-$ [M$^-$] 355.1949, found 355.1952. Mp = 89.2 – 93.1 °C.

Oxidative quenching using potassium permanganate ($KMnO_4$)

The addition step is independent on the oxidant used for the rearomatization of **3**. Quench the reaction with AcOH (1 equiv) and dilute the crude with THF/MeCN 5:1. Add $KMnO_4$ and keep stirring at room temperature. Extract with EtOAc/sat. $NaHCO_3$ followed by brine and dry the combined organic layers with Na_2SO_4. Purify the desired product using chromatography techniques.

1-(Quinazolin-4-yl)cyclohexane-1-carbonitrile (**8**)

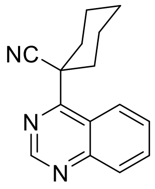

General procedure with quinazoline and **S-2** performed at rt (99%). Quench for 8 h with 5 equiv $KMnO_4$ (0.17 M). Purify by prep-TLC using hexanes/EtOAc 3:2 as eluent (Rf = 0.56). The desired product **8** was obtained as a white crystalline solid (29.1 mg, **61% yield**). ^{1}H NMR (500 MHz, $CDCl_3$) δ 9.28 (s, 1H), 8.69 (dd, *J* = 8.7, 1.2 Hz, 1H), 8.12 (dd, *J* = 8.4, 1.2 Hz, 1H), 7.92 (ddd, *J* = 8.4, 6.8, 1.3 Hz, 1H), 7.71 (ddd, *J* = 8.4, 6.9, 1.3 Hz, 1H), 2.53 – 2.49 (m, 2H), 2.15 – 2.09 (m, 2H), 2.01 – 1.95 (m, 4H), 1.91 – 1.87 (m, 1H), 1.36 – 1.29 (m, 1H). ^{13}C NMR (126 MHz, $CDCl_3$) δ 166.6, 153.9, 151.2, 133.8, 130.2, 127.9, 124.8, 122.4, 122.0, 44.5, 35.7, 25.1, 23.0. The analytical data matches those reported in the literature.[10]

Oxidative quenching using palladium on carbon (Pd/C 10 mol%)

The addition step is independent on the oxidant used for the rearomatization of **3**. Quench the reaction *via* dilution of the crude with AcOH followed by addition of Pd/C 10 mol% and keep stirring at 70 °C. Filter through Celite, extract with EtOAc/sat. $NaHCO_3$ and dry the combined organic layers with Na_2SO_4. Purify the desired product using chromatography techniques.

Methyl 2-(phenanthridin-6-yl)acetate (**9**)

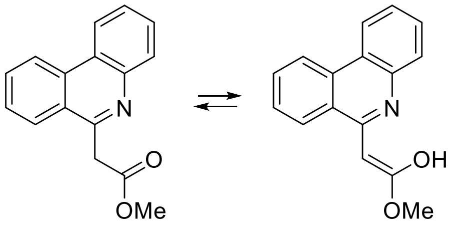

General procedure with phenanthridine and **2b** performed at rt (97%). Quench for 2.5 h with 7 mol% of Pd/C 10 mol% (0.2 M). Purify by column chromatography using MTBE/hexanes 1:9 as eluent (Rf (1:2) = 0.48). The desired product **9** was obtained as a yellow powder (46 mg, **92% yield, 5:1**). *Note: the ratio between tautomers is solvent dependent.* ^{1}H NMR keto tautomer (500 MHz, CD_3CN) δ 8.72 (d, *J* = 8.4 Hz, 1H), 8.64 (dd, *J* = 8.1, 1.4 Hz, 1H), 8.18 (d, *J* = 8.3 Hz, 1H), 8.03 (dd, *J* = 8.0, 1.4 Hz, 1H), 7.89 (ddd, *J* = 8.3, 7.0, 1.3 Hz, 1H), 7.76 – 7.73 (m,, 2H), 7.70 – 7.66 (m, 1H), 4.39 (s, 2H), 3.69 (s, 3H). Enol tautomer (30%): δ 12.34 (br s, 1H), 8.26 (dd, *J* = 8.2, 1.2 Hz, 1H), 8.13 (dd, *J* = 8.1, 1.2 Hz, 1H), 8.03 (dd, *J* = 8.0, 1.4 Hz, 1H), 7.70 – 7.66 (m, 1H), 7.49 (ddd, *J* = 8.3, 7.1, 1.2 Hz, 1H), 7.40 (ddd, *J* = 8.3, 7.1, 1.3 Hz, 1H), 7.21 (dd, *J* = 8.2, 1.2 Hz, 1H), 7.16 (ddd, *J* = 8.3, 7.2, 1.2 Hz, 1H), 5.53 (s, 1H), 3.71 (s, 3H). ^{13}C NMR (126 MHz, CD_3CN) δ 172.3, 171.8, 156.4, 151.9, 144.3, 136.3, 133.6, 132.6, 132.2, 131.8, 130.7, 130.4, 129.8, 129.1, 128.7, 128.0, 127.3, 126.2, 125.8, 125.4, 124.7, 123.7, 123.6, 123.5, 123.3, 123.2, 119.5, 117.3, 76.9, 52.6, 50.9, 43.1. *Note: overlapping of signals observed.* HRMS (GC-CI) calculated for $C_{16}H_{14}N_1O_1^+$ [M+H$^+$] 252.1019, found 252.1018. Mp = 91.9 – 93.6 °C.

3. Dihydropyridine Derivatives

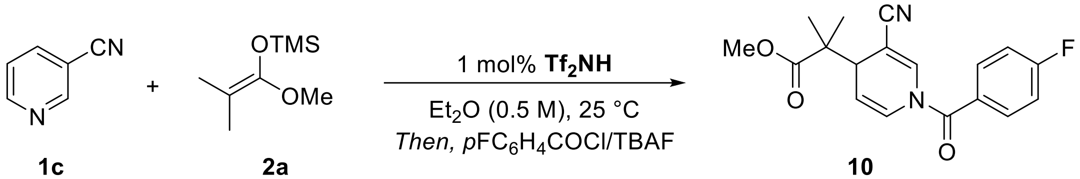

Weigh substrate **1c** (1 equiv, 20.8 mg, 0.2 mmol) in a vial charged with a magnetic stirring bar and place it under inert atmosphere. Dissolve it in Et_2O (0.4 ml) and add silyl ketene acetal **2a** (2 equiv, 80 µl, 0.4 mmol) *via* Hamilton syringe followed by Tf_2NH 0.1 M in DCM (1 mol%, 20 µl, 2 µmol). Then, seal with Teflon/parafilm and stir the mixture overnight at room temperature. Quench the reaction with 10 µl of triethylamine and add 4-fluorobenzoyl chloride (2 equiv, 48 µl, 0.4 mmol) followed by TBAF 1 M in THF (2 equiv, 0.4 ml, 0.4 mmol). Keep stirring for another hour. Dilute the mixture with ethyl acetate and wash the solution with saturated aqueous $NaHCO_3$ and brine. Then, dry the combined organic layers with Na_2SO_4 and concentrate the crude under reduced pressure. Purify by prep-TLC using hexanes/ethyl acetate 2:1 as eluent (R_f = 0.57). The desired product **10** was obtained as a colorless thick oil (66 mg, **99% yield**). ^1H NMR (500 MHz, CD_2Cl_2) δ 7.64 (s, 1H), 7.62 – 7.53 (m, 2H), 7.28 – 7.16 (m, 2H), 7.02 (d, *J* = 7.9 Hz, 1H), 5.12 (dd, *J* = 8.3, 5.2 Hz, 1H), 3.71 (s, 3H), 3.61 (d, *J* = 5.1 Hz, 1H), 1.29 (s, 3H), 1.24 (s, 3H). ^{13}C NMR (126 MHz, CD_2Cl_2) δ 176.2, 166.2, 165.3 (d, *J* = 252.9 Hz), 139.4, 131.8 (d, *J* = 9.3 Hz), 128.4 (d, *J* = 3.5 Hz), 125.4, 119.1, 116.5 (d, *J* = 22.3 Hz), 108.0, 91.8, 52.4, 48.6, 42.8, 21.8, 21.3. ^{19}F NMR (471 MHz, CD_2Cl_2) δ – 106.37. HRMS (ESI^+) calculated for $C_{18}H_{17}N_2O_3F_1Na_1^+$ [M+Na^+] 351.1115, found 351.1115.

4. Synthesis of Catalysts

^{Ph}PADI

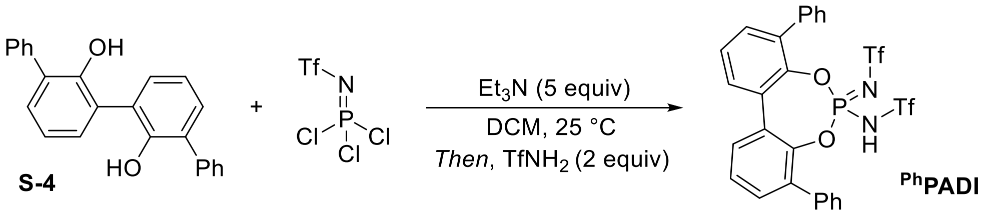

In a flame-dried Schlenk, dissolve compound **S-4** (1 equiv, 50 mg, 0.15 mmol) in DCM (5 ml) under inert conditions. Add *N*-trifluoromethylsulfonyl-*P*,*P*,*P*-trichlorophosphazene (1 equiv, 30 µl, 0.15 mmol) and triethylamine (5.7 equiv, 120 µl, 0.86 mmol). Stir for 1.5 h at room temperature and add trifluoromethanesulfonamide in one portion (2 equiv, 50 mg, 0.3 mmol). Stir for 3 h more and concentrate the crude. Purify by column chromatography using hexanes/ethyl acetate 4:1 as eluent (R_f (pentane/acetone 1:1) = 0.28). Acidify with DOWEX 50Wx8 in MeOH, dry under high vacuum for 24 h and store under argon at – 20°C. **^{Ph}PADI** was obtained as a beige powder (79 mg, **81% yield**). ^{1}H NMR (500 MHz, CD_3OD) δ 7.63 – 7.62 (m, 4H), 7.58 (dd, *J* = 7.6, 1.9 Hz, 2H), 7.52 – 7.50 (m, 2H), 7.46 (td, *J* = 7.9, 1.9 Hz, 2H), 7.39 – 7.36 (m, 4H), 7.33 – 7.30 (m, 2H). ^{13}C NMR (126 MHz, CD_3OD) δ 145.9, 145.8, 137.9, 136.5, 136.4, 132.5, 132.5, 131.2, 131.1, 130.9, 130.8, 130.8, 128.9, 128.4, 127.4, 127.4, 121.2 (qd, *J* = 2.5, 0.1 Hz). ^{31}P NMR (203 MHz, CD_3OD) δ – 5.24. ^{19}F NMR (471 MHz, CD_3OD) δ – 80.86. HRMS (ESI$^-$) calculated for $C_{26}H_{17}N_2O_6P_1S_2F_6^-$ [M-H$^-$] 661.0097, found 661.0103. LCMS purity 99% (50 mm Eclipse Plus C18 1.8 ml, 4.6 mm. Acetonitrile/aqueous TFA 0.1% (gradient 70% – 5′ – 90%), 1 ml/min, 308 K, 254 nm/ES–API$^+$). Structure analysis *via* single-crystal X-ray diffraction of the triethylammonium salt. *Note: attempts to scale up may lead to challenges in the purification due to the formation of the catalyst–base adduct.*

[1,1':3',1":3",1'"-Quaterphenyl]-2',2"-diol (**S-4**)

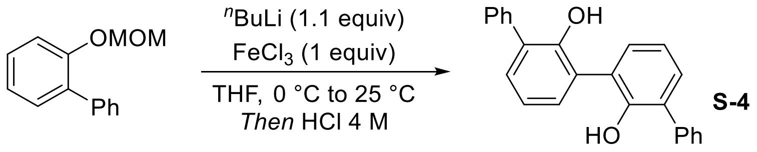

To a solution of 2-(methoxymethoxy)-1,1'-biphenyl (1 equiv, 180 µl, 0.9 mmol)[11] in THF (6 ml) under inert conditions cooled down to 0 °C, add dropwise *n*-butyllithium 2.5 M in hexanes (1.1 equiv, 0.4 ml, 1.0 mmol) and stir for 30 min. Then, add this solution in one portion over anhydrous $FeCl_3$ (1 equiv, 150 mg, 0.9 mmol) previously dissolved in THF (2 ml) under inert conditions and cooled down to 0 °C. Stir vigorously overnight while warming up to room temperature. Dilute the mixture with Et_2O and wash it with brine. Extract the aqueous phase and then dry the combined organic layers with Na_2SO_4. Concentrate the crude under reduced pressure and – without further purification – dissolve it in 2 ml HCl 4 M in dioxane. Stir at room temperature overnight. Dilute the mixture with Et_2O and wash it with water. Extract the aqueous phase and then dry the combined organic layers with Na_2SO_4. Concentrate and purify by column chromatography using hexanes/ethyl acetate 9:1 as eluent (R_f = 0.31). Dry the sample further as an azeotropic mixture with toluene. The desired product **S-4** was obtained as a white crystalline solid (106 mg, **70 % yield**). ^{1}H NMR (500 MHz, $CDCl_3$) δ 7.57 – 7.55 (m, 4H), 7.49 – 7.46 (m, 4H), 7.41 – 7.34 (m, 6H), 7.13 (t, *J* = 7.5 Hz, 2H), 5.77 (s, 2H). ^{13}C NMR (126 MHz, $CDCl_3$) δ 149.8, 137.5, 131.1, 130.8, 129.6, 129.5, 128.9, 127.8, 125.0, 121.5. The analytical data matches those reported in the literature.[12]

N-(4,8-Bis(3,5-bis(trifluoromethyl)phenyl)-6-(((trifluoromethyl)sulfonyl)imino)-6λ5-dibenzo[*d,f*][1,3,2]dioxaphosphepin-6-yl)-1,1,1-trifluoromethanesulfonamide (**S-5**)

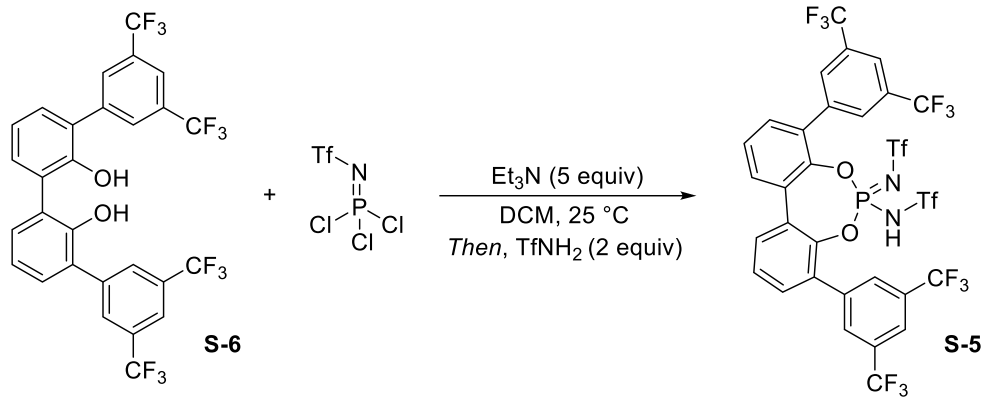

In a flame-dried Schlenk, dissolve compound **S-6** (1 equiv, 18 mg, 0.03 mmol) in DCM (0.8 ml) under inert conditions. Add *N*-trifluoromethylsulfonyl-*P*,*P*,*P*-trichlorophosphazene (1 equiv, 5 µl, 0.03 mmol) and triethylamine (5 equiv, 20 µl, 0.14 mmol). Stir for 30 min at room temperature and add trifluoromethanesulfonamide in one portion (2 equiv, 10 mg, 0.06 mmol). Stir for 2 h more and purify by preparative TLC using pentane/acetone 1:1 as eluent (R_f = 0.47) and then wash with EtOAc. Acidify with DOWEX 50Wx8 in MeOH, dry under high vacuum for 24 h and store under argon at –20°C. Product **S-5** was obtained as a beige powder (23 mg, **82% yield**). ^{1}H NMR (500 MHz, CD_3OD) δ 8.12 (s, 4H), 7.93 (s, 2H), 7.79 (dd, *J* = 7.3, 2.0 Hz, 2H), 7.64 – 7.58 (m, 4H). ^{13}C NMR (126 MHz, CD_3OD) δ 145.9, 145.8, 140.3, 133.7, 133.7, 132.9, 132.7, 132.6, 132.6, 132.4, 132.3, 132.3, 132.2, 131.5, 131.4, 131.0, 131.0, 128.1, 128.1, 128.1, 125.9, 123.7, 122.4, 122.4, 122.3, 122.3, 122.3. *Note: overlapping of signals observed.* ^{31}P NMR (203 MHz) δ – 4.51. ^{19}F NMR (471 MHz, CD_3OD) δ – 64.22, – 81.27. HRMS (ESI$^-$) calculated for $C_{30}H_{13}N_2O_6P_1S_2F_{18}^-$ [M-H$^-$] 932.9593, found 932.9593. LCMS purity 99% (50 mm Eclipse Plus C18 1.8 ml, 4.6 mm. Methanol/aqueous TFA 0.1% (gradient 70% – 5´ – 95%), 1 ml/min, 308 K, 254 nm/ES–API$^+$).

3,3''',5,5'''-Tetrakis(trifluoromethyl)-[1,1':3',1'':3'',1'''-quaterphenyl]-2',2''-diol (**S-6**)

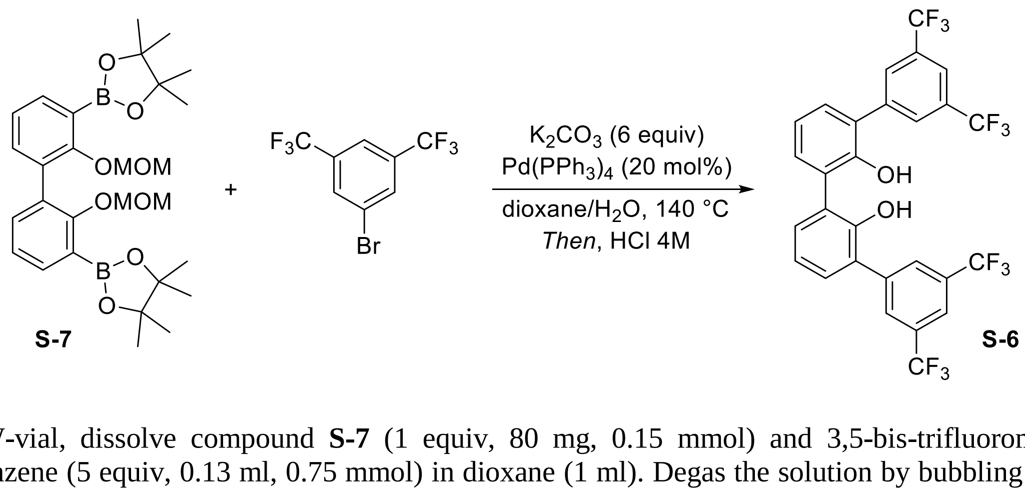

In a MW-vial, dissolve compound **S-7** (1 equiv, 80 mg, 0.15 mmol) and 3,5-bis-trifluoromethyl)-5-bromobenzene (5 equiv, 0.13 ml, 0.75 mmol) in dioxane (1 ml). Degas the solution by bubbling argon for 20 min and add K_2CO_3 (6 equiv, 120 mg, 0.9 mmol) in water (0.5 ml) followed by $Pd(PPh_3)_4$ (10 mol%, 16 mg, 0.01 mmol). *Note: aqueous solution also previously degassed by bubbling argon for 30 min.* Seal the vial and stir under MW irradiation at 140 °C for 1.5 h. Dilute the mixture in Et_2O and quench with sat. NH_4Cl. Extract, wash with brine and dry the combined organic layers with Na_2SO_4. Concentrate the crude under reduced pressure and – without further purification – dissolve it in 2 ml HCl 0.4 M in dioxane. Stir at

room temperature overnight. Dilute the mixture with Et_2O and wash it with water. Extract the aqueous phase and then dry the combined organic layers with Na_2SO_4. Concentrate the crude under reduced pressure and purify by column chromatography using hexanes/ethyl acetate 20:1 as eluent (R_f (9:1) = 0.43). Dry the sample further as an azeotropic mixture with toluene. The desired product **S-6** was obtained as a white crystalline solid (49 mg, **53% yield**). ^{1}H NMR (500 MHz, $CDCl_3$) δ 8.09 (s, 4H), 7.88 (s, 2H), 7.48 (dd, *J* = 7.6, 1.7 Hz, 2H), 7.39 (dd, *J* = 7.6, 1.7 Hz, 2H), 7.23 (t, *J* = 7.6, 2H), 5.42 (s, 2H). ^{13}C NMR (126 MHz, $CDCl_3$) δ 150.5, 139.6, 132.2, 131.9, 131.8, 131.8, 131.6, 131.4, 129.7, 129.7, 127.0, 126.7, 124.6, 123.0, 122.4, 122.3, 121.4, 121.3, 121.3, 121.3, 121.3, 120.2. *Note: overlapping of signals observed.* ^{19}F NMR (471 MHz, $CDCl_3$) δ – 62.81. HRMS (ESI^-) calculated for $C_{28}H_{13}F_{12}O_2^-$ [M-H^-] 609.0729, found 609.0741. Mp 152.6 – 153.9 °C.

2,2'-(2,2'-Bis(methoxymethoxy)-[1,1'-biphenyl]-3,3'-diyl)bis(4,4,5,5-tetramethyl-1,3,2-dioxaborolane) (**S-7**)

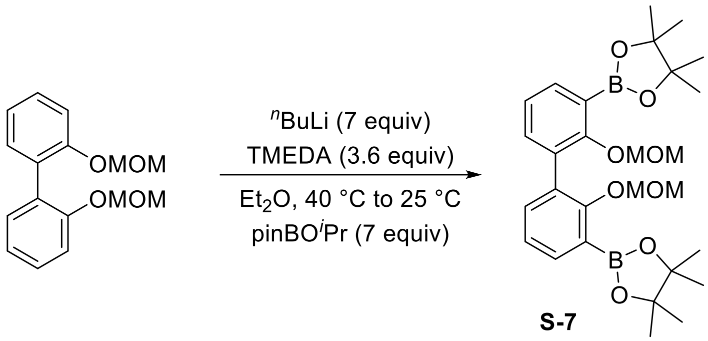

S-7

In a flame-dried Schlenk, dissolve TMEDA (3.6 equiv, 0.27 ml, 1.8 mmol) in Et_2O (8 ml) under inert conditions, add *n*-butyllithium 2.5 M in hexanes (7 equiv, 1.4 ml, 3.5 mmol) and stir for 30 min at room temperature. Add 2,2'-bis(methoxymethoxy)-1,1'-biphenyl (1 equiv, 125 ml, 0.5 mmol)[13] and reflux the mixture at 40 °C for 3 h. Cool down and slowly add 2-isopropoxy-4,4,5,5-tetramethyl-1,3,2-dioxaborolane (7 equiv, 0.7 ml, 3.5 mmol). Keep stirring at room temperature for 3 h more, dilute the suspension with Et_2O and quench with sat. NH_4Cl. Extract, wash with brine and dry the combined organic layers with Na_2SO_4. Concentrate the crude under reduced pressure and purify by column chromatography using hexanes/ethyl acetate 9:1 as eluent (R_f (2:1) = 0.58). The desired product **S-7** was obtained as a white crystalline solid (116 mg, **44% yield**). ^{1}H NMR (500 MHz, CD_2Cl_2) δ 7.71 (dd, *J* = 7.4, 1.9 Hz, 2H), 7.47 (dd, *J* = 7.5, 1.9 Hz, 2H), 7.17 (t, *J* = 7.4 Hz, 2H), 4.80 (s, 4H), 2.80 (s, 6H), 1.35 (s, 24H). ^{13}C NMR (126 MHz, CD_2Cl_2) δ 161.2, 136.8, 135.5, 133.6, 123.7, 101.3, 84.2, 56.6, 25.2. *Note: C2 and C2' not resolved due to coupling with the boron atom.* ^{11}B NMR (161 MHz, CD_2Cl_2) δ 30.41. HRMS (ESI^+) calculated for $C_{28}H_{40}O_8B_2Na_1^+$ [M+Na^+] 549.2801, found 549.2801. Mp 110.9 – 117.2 °C.

N-(4,8-Diphenyl-1,3,9,11-tetrakis(trifluoromethyl)-6-(((trifluoromethyl)sulfonyl)imino)-6λ⁵-dibenzo[*d*,*f*][1,3,2]dioxaphosphepin-6-yl)-1,1,1-trifluoromethanesulfonamide (**S-8**)

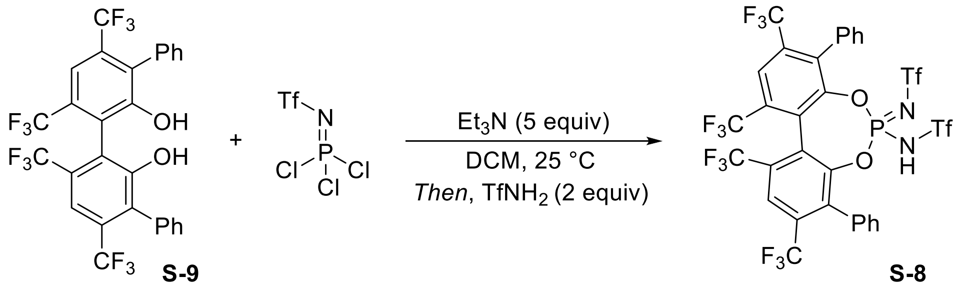

In a flame-dried Schlenk, dissolve compound **S-9** (1 equiv, 18 mg, 0.03 mmol) in DCM (0.8 ml) under inert conditions. Add *N*-trifluoromethylsulfonyl-*P*,*P*,*P*-trichlorophosphazene (1 equiv, 5 µl, 0.03 mmol) and triethylamine (5 equiv, 20 µl, 0.14 mmol). Stir for 30 min at room temperature and add trifluoromethanesulfonamide in one portion (2 equiv, 10 mg, 0.06 mmol). Stir for 2 h more and purify by preparative TLC using pentane/acetone 1:1 as eluent (R_f = 0.39) and then wash with EtOAc. Acidify with DOWEX 50Wx8 in MeOH, dry under high vacuum for 24 h and store under argon at – 20°C. Product **S-8** was obtained as a brownish powder (25 mg, **89% yield**). ^{1}H NMR (500 MHz, CD_3OD) δ 8.11 (s, 2H), 7.65 (br s, 2H), 7.43 – 7.37 (m, 6H), 7.15 (d, *J* = 6.3 Hz, 2H). ^{13}C NMR (126 MHz, CD_3OD) δ 147.0, 147.0, 138.7, 132.3, 132.3, 132.1, 132.1, 130.6, 130.4, 129.3, 128.4, 127.5, 127.0, 126.8, 125.8, 123.6, 123.6, 121.7, 121.7, 121.5, 121.4, 120.8, 120.8, 119.3, 118.3, 118.2. *Note: overlapping of signals observed.* ^{31}P NMR (203 MHz, CD_3OD) δ – 10.01. ^{19}F NMR (471 MHz, CD_3OD) δ – 58.72, – 59.17, – 80.86. HRMS (ESI^-) calculated for $C_{30}H_{12}F_{18}N_2O_2P_1S_2^-$ [M-H$^-$] 932.9593, found 932.9591. LCMS purity 97% (50 mm Eclipse Plus C18 1.8 ml, 4.6 mm. Acetonitrile/aqueous TFA 0.1% (gradient 30% – 5´ – 60%), 1 ml/min, 308 K, 254 nm/ES–API$^+$).

4',4'',6',6''-Tetrakis(trifluoromethyl)-[1,1':3',1'':3'',1'''-quaterphenyl]-2',2''-diol (**S-9**)

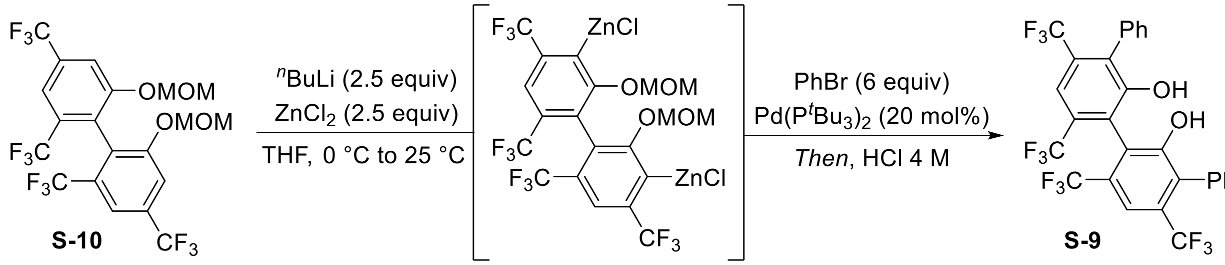

To a solution of compound **S-10** (1 equiv, 110 mg, 0.2 mmol) in THF (2 ml) under inert conditions cooled down to 0 °C, add dropwise *n*-butyllithium 2.5 M in hexanes (2.5 equiv, 0.2 ml, 0.5 mmol) and stir for 90 min. Then, cool down to – 78 °C and add slowly $ZnCl_2$ 1.9 M in 2-Me-THF (2.5 equiv, 0.27 ml, 0.5 mmol). Immediately warm up the solution to room temperature and keep stirring for 5 h. In parallel, prepare a solution of bis(tri-tert-butylphosphine)palladium (20 mol%, 20 mg, 0.04 mmol) and bromobenzene (6 equiv, 0.12 ml, 1.2 mmol) in THF (2 ml) under inert conditions in a flame-dried Schlenk. *Note: solvent previously degassed by bubbling argon for 15 min.* Add the zincate solution, seal and reflux the mixture at 70 °C overnight. Dilute the mixture with Et_2O and quench with sat. NH_4Cl. Extract, wash the combined organic layers with brine and dry with Na_2SO_4. Concentrate the crude under reduced pressure and – without further purification – dissolve it in 2 ml HCl 0.4 M in dioxane. Stir at room temperature overnight. Dilute the mixture with Et_2O and wash it with water. Extract the aqueous phase and then dry the combined organic layers with Na_2SO_4. Concentrate the crude under reduced pressure and purify by column chromatography

using hexanes/ethyl acetate 20:1 as eluent (R_f (3:1) = 0.50). Dry the sample further as an azeotropic mixture with toluene. The desired product **S-9** was obtained as a yellowish crystalline solid (79 mg, **65% yield**). ^{1}H NMR (500 MHz, $CDCl_3$) δ 7.75 (s, 2H), 7.59 – 7.51 (m, 6H), 7.34 (d, *J* = 7.9 Hz, 4H), 5.16 (s, 2H). ^{13}C NMR (126 MHz, $CDCl_3$) δ 152.6, 130.6, 130.4, 130.4, 130.1, 130.0, 129.7, 129.6, 126.3, 126.3, 124.1, 124.1, 122.9, 121.9, 121.9, 119.8, 119.7, 115.8. *Note: overlapping of signals observed.* ^{19}F NMR (471 MHz, $CDCl_3$) δ – 58.39, – 60.54. HRMS (ESI$^-$) calculated for $C_{28}H_{14}F_{12}O_2^-$ [M-H$^-$] 609.0729, found 609.0734. Mp 156.0 – 128.8 °C.

2,2'-Bis(methoxymethoxy)-4,4',6,6'-tetrakis(trifluoromethyl)-1,1'-biphenyl (**S-10**)

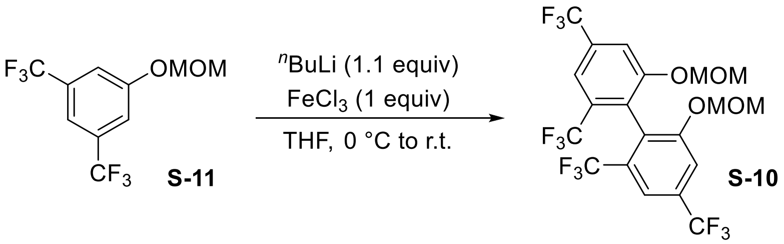

To a solution of compound **S-11** (1 equiv, 366 µl, 1.8 mmol) in THF (15 ml) under inert conditions cooled down to 0 °C, add dropwise *n*-butyllithium 2.5 M in hexanes (1.1 equiv, 0.8 ml, 2.0 mmol) and stir for 30 min. Then, add this solution in one portion over anhydrous $FeCl_3$ (1 equiv, 310 mg, 1.9 mmol) previously dissolved in THF (4 ml) under inert conditions and cooled down to 0 °C. Stir vigorously overnight while warming up to room temperature. Dilute the mixture with Et_2O and wash it with brine. Extract the aqueous phase and then dry the combined organic layers with Na_2SO_4. Concentrate the crude under reduced pressure and purify by column chromatography using hexanes/ethyl acetate 20:1 as eluent (R_f (9:1) = 0.41). The desired product **S-10** was obtained as a pale yellow solid (412 mg, **84% yield**). ^{1}H NMR (500 MHz, $CDCl_3$) δ 7.70 (s, 2H), 7.67 (s, 2H), 5.12 (d, *J* = 7.2 Hz, 2H), 5.09 (d, *J* = 7.2 Hz, 2H), 3.36 (s, 6H). ^{13}C NMR (126 MHz, $CDCl_3$) δ 156.1, 132.6 (q, *J* = 33.2 Hz), 131.4 (q, *J* = 32.4 Hz), 126.0, 123.3 (q, *J* = 271.5 Hz), 122.9 (q, *J* = 276.6 Hz), 116.4 (br s), 114.3 (q, *J* = 3.1 Hz), 95.1, 56.3. ^{19}F NMR (471 MHz, $CDCl_3$) δ – 60.36, – 62.93. HRMS (ESI$^+$) calculated for $C_{20}H_{14}F_{12}O_4Na^+$ [M+Na$^+$] 569.0593, found 569.0594. Mp 87.6 – 88.98 °C.

1-(Methoxymethoxy)-3,5-bis(trifluoromethyl)benzene (**S-11**)

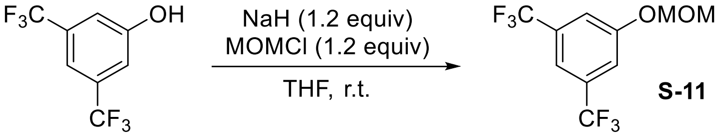

To a suspension of sodium hydride 60% (1.2 equiv, 630 mg, 15.8 mmol) in THF (10 ml) under inert conditions, add 3,5-bis(trifluoromethyl)phenol (1 equiv, 2 ml, 13.2 mmol) carefully and stir for 30 min at room temperature. Then, add chloromethyl methyl ether (1.2 equiv, 1.2 ml, 15.8 mmol) and keep stirring the mixture overnight. Quench the reaction with water and extract the solution with DCM. Then, wash the combined organic layers with saturated aqueous $NaHCO_3$, brine and dry with Na_2SO_4. Concentrate the crude under reduced pressure and purify by column chromatography using hexanes as eluent (R_f = 0.33). The desired product **S-11** was obtained as colorless oil (3.29 g, **91% yield**). ^{1}H NMR (500 MHz, $CDCl_3$), δ 7.51 (s, 1H), 7.47 (s, 2H), 5.25 (s, 2H), 3.50 (s, 3H). ^{13}C NMR (126 MHz, $CDCl_3$) δ 158.0, 132.9 (q, *J* = 33.6 Hz), 123.2 (q, *J* = 274.8 Hz), 116.7 (br q, *J* = 3.8 Hz), 115.5 (hept, *J* = 3.8 Hz), 94.8, 56.5. ^{19}F NMR (471 MHz, $CDCl_3$) δ – 63.02. HRMS (GC-CI) calculated for $C_{10}H_8O_2F_6^{\cdot+}$ [M$^{\cdot+}$] 274.0423, found 274.0424.

^{CF3}PADI

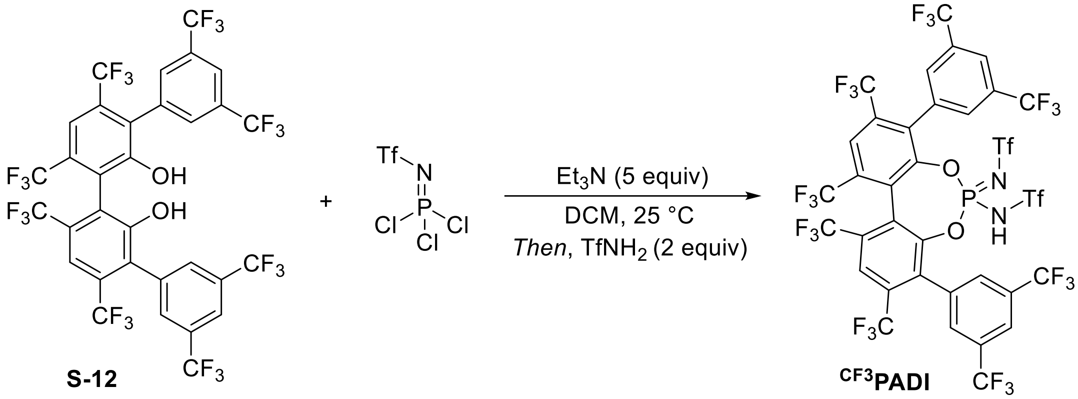

In a flame-dried Schlenk, dissolve compound **S-12** (1 equiv, 26 mg, 0.03 mmol) in DCM (0.8 ml) under inert conditions. Add *N*-trifluoromethylsulfonyl-*P*,*P*,*P*-trichlorophosphazene (1 equiv, 5 µl, 0.03 mmol) and triethylamine (5 equiv, 20 µl, 0.14 mmol). Stir for 30 min at room temperature and add trifluoromethanesulfonamide in one portion (2 equiv, 10 mg, 0.06 mmol). Stir for 2 h more and purify by preparative TLC using pentane/acetone 1:1 as eluent (R_f = 0.73) and then wash with EtOAc. Acidify with DOWEX 50Wx8 in MeOH, dry under high vacuum for 24 h and store under argon at – 20°C. **^{CF3}PADI** was obtained as a brownish powder (31 mg, **86% yield**). ^{1}H NMR (500 MHz, CD_3OD) δ 8.27 (s, 2H), 8.20 (s, 2H), 8.05 (s, 2H), 7.85 (s, 2H). ^{13}C NMR (126 MHz, CD_3OD) δ 146.8, 146.7, 135.2, 135.1, 132.8, 132.6, 132.6, 132.4, 132.3, 131.5, 131.4, 131.2, 131.2, 130.9, 130.9, 130.6, 130.3, 130.3, 127.0, 126.3, 126.3, 125.6, 125.5, 124.2, 124.1, 123.4, 123.4, 122.8, 122.8, 122.7, 122.7, 122.7, 122.0, 122.0, 121.2, 121.2, 120.7, 120.7, 118.2, 118.1. *Note: overlapping of signals observed.* ^{31}P NMR (203 MHz, CD_3OD) δ – 9.44. ^{19}F NMR (471 MHz, CD_3OD) δ – 58.66, – 58.95, – 64.35, – 64.44, – 81.12. HRMS (ESI$^-$) calculated for $C_{34}H_8F_{30}O_6N_2P_1S_2^-$ [M-H$^-$] 1204.9088, found 1204.9093. LCMS purity 99% (50 mm Eclipse Plus C18 1.8 ml, 4.6 mm. Acetonitrile/aqueous TFA 0.1% (gradient 70% – 5´ – 90%), 1 ml/min, 308 K, 254 nm/ES–API$^+$).

3,3''',4',4'',5,5''',6',6''-Octakis(trifluoromethyl)-[1,1':3',1'':3'',1'''-quaterphenyl]-2',2''-diol (**S-12**)

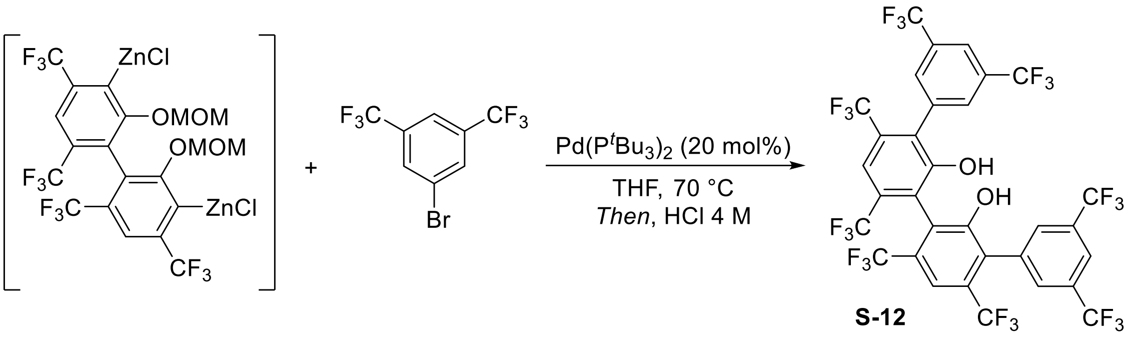

Prepare a solution of bis(tri-tert-butylphosphine)palladium (20 mol%, 20 mg, 0.04 mmol) and 3,5-bis-trifluoromethyl)-5-bromobenzene (10 equiv, 0.35 ml, 2 mmol) in THF (2 ml) under inert conditions in a flame-dried Schlenk. *Note: solvent previously degassed by bubbling argon for 15 min.* Add the zincate solution from **S-10** (0.2 mmol, see synthesis of **S-9**), seal and reflux the mixture at 70 °C overnight. Dilute the mixture with Et_2O and quench with sat. NH_4Cl. Extract, wash the combined organic layers with brine and dry with Na_2SO_4. Concentrate the crude under reduced pressure and – without further purification – dissolve it in 2 ml HCl 0.4 M in dioxane. Stir at room temperature overnight. Dilute the mixture with Et_2O

and wash it with water. Extract the aqueous phase and then dry the combined organic layers with Na_2SO_4. Concentrate the crude under reduced pressure and purify by column chromatography using hexanes/ethyl acetate 30:1 as eluent (R_f (9:1) = 0.59). Dry the sample further as an azeotropic mixture with toluene. The desired product **S-12** was obtained as a yellowish crystalline solid (88 mg, **50% yield**). ^1H NMR (500 MHz, $CDCl_3$) δ 8.05 (s, 2H), 7.85 (s, 2H), 7.82 (s, 2H), 7.79 (s, 2H), 4.96 (s, 2H). ^{13}C NMR (126 MHz, $CDCl_3$) δ 152.7, 133.4, 133.2, 133.1, 132.9, 132.8, 132.7, 132.6, 132.4, 132.3, 132.1, 132.0, 131.9, 131.6, 130.5, 129.2, 128.3, 128.1, 126.2, 126.1, 125.8, 125.8, 124.0, 124.0, 123.9, 123.9, 123.9, 123.8, 123.8, 123.8, 123.6, 123.6, 122.0, 121.8, 121.8, 121.4, 121.4, 119.6, 119.6, 119.2, 119.2, 116.8, 116.8, 116.8. *Note: overlapping of signals observed.* ^{19}F NMR (471 MHz, $CDCl_3$) δ – 58.25, – 60.82, – 63.05, – 63.07. HRMS (ESI^-) calculated for $C_{32}H_{10}F_{24}O_2^-$ [$M-H^-$] 881.0225, found 881.0229. Mp 185.6 – 189.9 °C.

5. Synthesis of Substrates

8-Chloro-11-(1-(pyridin-3-ylsulfonyl)piperidin-4-ylidene)-6,11-dihydro-5*H*-benzo[5,6]cyclohepta[1,2-*b*]pyridine (**1o**)

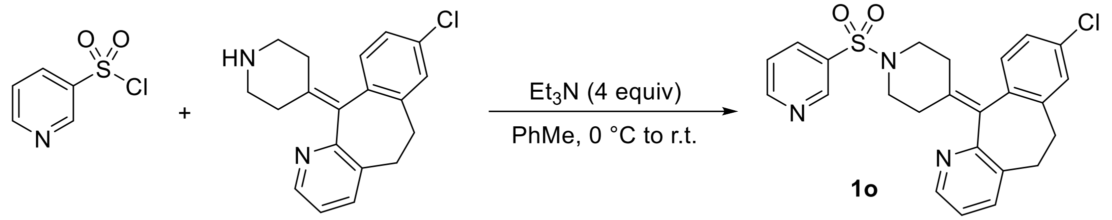

To a solution of 8-chloro-11-(piperidin-4-ylidene)-6,11-dihydro-5H-benzo[5,6]cyclohepta[1,2-*b*]pyridine or desloratadine (1 equiv, 50 mg, 0.16 mmol) in toluene (0.9 ml) under inert conditions, add triethylamine (4 equiv, 90 μl, 0.65 mmol). Cool down the solution to 0 °C and add slowly pyridine-3-sulfonyl chloride (1.3 equiv, 25 μl, 0.2 mmol). Stir overnight while warming up to room temperature. Concentrate the crude and purify by column chromatography using MTBE as eluent (R_f = 0.21). Product **1o** was obtained as a white foam (60 mg, **83% yield**). ^{1}H NMR (500 MHz, $CDCl_3$) δ 8.96 (d, *J* = 2.3 Hz, 1H), 8.81 (dd, *J* = 4.8, 1.6 Hz, 1H), 8.33 (dd, *J* = 4.9, 1.7 Hz, 1H), 8.02 (dt, *J* = 8.0, 2.0 Hz, 1H), 7.46 (dd, *J* = 8.0, 4.8 Hz, 1H), 7.39 (dd, *J* = 7.7, 1.7 Hz, 1H), 7.12 (d, *J* = 2.1 Hz, 1H), 7.08 (ddd, *J* = 9.1, 7.3, 3.5 Hz, 2H), 6.99 (d, *J* = 8.1 Hz, 1H), 3.32 (ddd, *J* = 11.3, 7.2, 4.0 LHz, 1H), 3.24 (ddt, *J* = 19.4, 12.0, 4.1 Hz, 3H), 2.99 (tdd, *J* = 10.8, 6.0, 4.0 Hz, 2H), 2.82 – 2.74 (m, 1H), 2.71 (ddd, *J* = 16.0, 7.9, 4.6 Hz, 1H), 2.62 (ddd, *J* = 14.1, 8.2, 4.2 Hz, 1H), 2.49 (ddd, *J* = 14.0, 8.1, 4.2 Hz, 1H), 2.35 (dtd, *J* = 13.6, 6.4, 4.0 Hz, 2H). ^{13}C NMR (126 MHz, $CDCl_3$) δ 156.3, 153.4, 148.4, 146.5, 139.5, 137.9, 137.2, 135.3, 135.2, 135.1, 133.4, 133.3, 133.2, 130.3, 129.0, 126.3, 123.8, 122.5, 47.3, 47.3, 31.5, 31.4, 30.2, 29.9. HRMS (ESI$^+$) calculated for $C_{24}H_{23}Cl_1N_3O_2S_1^+$ [M+H$^+$] 452.1194, found 452.1194. *Note: melting point measurements inconclusive, potential polymorphism.*

(3*S*,5*S*,8*R*,9*S*,10*S*,13*S*,14*S*)-10,13-Dimethyl-17-oxohexadecahydro-1*H*-cyclopenta[*a*]phenanthren-3-yl pyridazine-3-carboxylate (**S-3**)

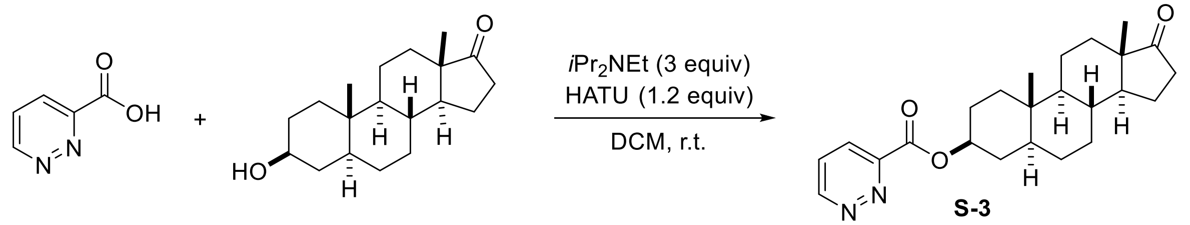

To a solution of pyridazine-3-carboxylic acid (1.2 equiv, 150 mg, 1.2 mmol) and HATU (1.2 equiv, 455 mg, 1.2 mmol) in DCM (4 ml) under inert conditions, add *N,N*-diisopropylethylamine (3 equiv, 0.5 ml, 2.9 mmol). Stir at room temperature for 1.5 hours and add epiandrosterone (1 equiv, 290 mg, 1 mmol). Keep stirring overnight and dilute with ethyl acetate. Wash with water and dry with Na_2SO_4. Concentrate the crude and purify by column chromatography using hexanes/ethyl acetate 1:2 as eluent (R_f (EtOAc) = 0.48). Product **S-3** was obtained as a white crystalline solid (172 mg, **43% yield**). ^{1}H NMR (500 MHz, CD_2Cl_2) δ 9.29 (dd, *J* = 5.0, 1.7 Hz, 1H), 8.14 (dd, *J* = 8.5, 1.8 Hz, 1H), 7.65 (dd, *J* = 8.5, 5.1 Hz, 1H), 5.04 (tt, *J* = 11.3, 5.0 Hz, 1H), 2.37 (dd, *J* = 19.0, 8.8 Hz, 1H), 2.04 – 1.96 (m, 2H), 1.90 (ddd, *J* = 11.9, 8.7, 5.8 Hz, 1H), 1.83 – 1.69 (m, 5H), 1.67 – 1.53 (m, 3H), 1.51 – 1.44 (m, 1H), 1.37 – 1.17 (m, 6H), 1.12 (td, *J* = 13.5, 4.0 Hz, 1H), 1.00 (qd, *J* = 11.9, 5.4 Hz, 1H), 0.90 (s, 3H), 0.82 (s, 3H), 0.75 (ddd, *J* = 12.3, 10.4, 4.0 Hz, 1H). ^{13}C NMR (126 MHz, CD_2Cl_2) δ 220.6, 163.8, 153.3, 152.5, 127.7, 127.3, 76.2, 54.6, 51.6, 47.9, 45.0,

37.0, 36.0, 35.3, 34.1, 31.9, 31.1, 28.6, 27.6, 22.0, 20.8, 13.9, 12.3. *Note: overlapping of signals observed.* HRMS (ESI$^+$) calculated for $C_{24}H_{32}N_2O_3Na_1^+$ [M+Na$^+$] 419.2305, found 419.2304. Mp 273.1 – 275.2 °C.

Isopropyl acetate tert-butyldimethylsilyl enol ether (**2c**)

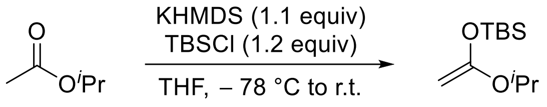

2c

To a solution of potassium bis(trimethylsilyl)amide (1.1 equiv, 5 g, 25 mmol) in THF/toluene (40 ml, 7:1) under inert conditions and cooled down to – 78 °C, add isopropyl acetate (1 equiv, 2.6 ml, 22 mmol) carefully and stir for 3 h. Then, add *tert*-butyldimethylsilyl choride (1.1 equiv, 3.6 g, 24 mmol) and keep stirring the mixture overnight while warming up to room temperature. Quench the reaction with cold saturated aqueous $NaHCO_3$ and extract the solution with Et_2O. Then, wash the combined organic layers with saturated $CuSO_4$, saturated $NaHCO_3$, brine and dry with Na_2SO_4. Concentrate the crude under reduced pressure and purify *via* distillation (T_b = 66 °C, 2.8 mbar). The desired product **2c** was obtained as colorless oil (1.92 g, **91% yield**). ^{1}H NMR (500 MHz, C_6D_6) δ 3.98 (hept, *J* = 6.1, 1H), 3.60 (d, *J* = 2.2, 1H), 3.21 (d, *J* = 2.4, 1H), 1.05 (d, *J* = 6.1, 6H), 0.99 (s, 9H), 0.22 (s, 6H). ^{13}C NMR (126 MHz, C_6D_6) δ 160.1, 69.9, 62.1, 25.9, 21.6, 18.3, – 4.2. The analytical data matches those reported in the literature.[14]

6. Optimization studies

To a solution of triflimide in MeCN-d_3 in a vial under argon atmosphere was added SKA **2a** or **2b** and the resulting mixture was stirred for 30 min at room temperature. Then, the substrate was added at the specified temperature (0.05 mmol) and the stirring continued overnight. The reaction was quenched with 5 µl of triethylamine and the crude was analyzed by ^{1}H NMR. *Notes: yield equals conversion (CHPh$_3$ as internal standard). Measurements under non-anhydrous conditions eventually show traces of dihydropyridine derivative. Retro Mukaiyama-Michael reaction observed if heated to 60 °C.*

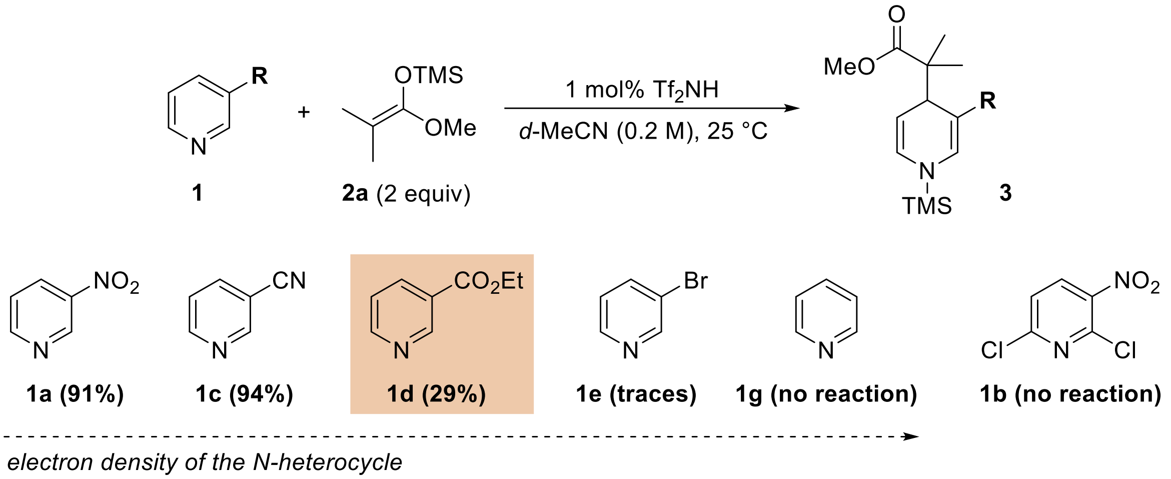

Modification	Conversion
3 days	29%
10 mol%	12%
40 °C	16%
**0 °C**	**54%**
**–10 °C**	**40%**
–20 °C	0%
**0.5 M**	**45%**
**1 M**	**54%**
neat	23%
**0 °C/1 M**	**63%**
TBS-	34%
d-DCM	28%
d-PhMe	17%
Et_2O	14%

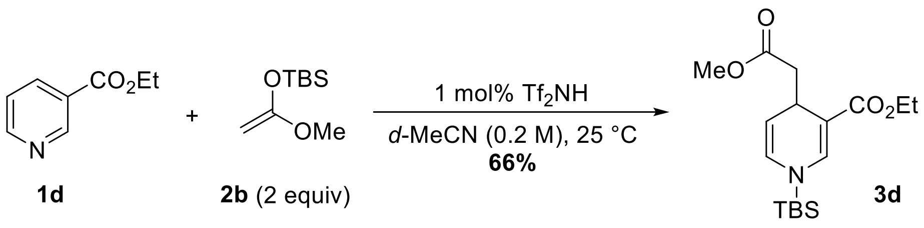

Modification	Yield
**0 °C**	**88%**
**1 M**	**85%**
**0 °C/1 M**	**97%**

This conditions showed a maximum of 16% addition for 3-bromopyridine (**1e**). In this case, competing decomposition of the SKA towards a complex mixture was observed.

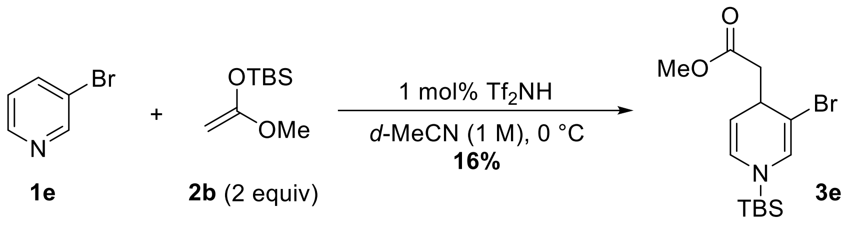

Modification	Yield
25 °C	traces
–20 °C	traces

In order to expand further the chemoselectivity, we explored the reactivity of less acidic catalysts such as bis-arylsulfonimides or diphenyl phosphate.

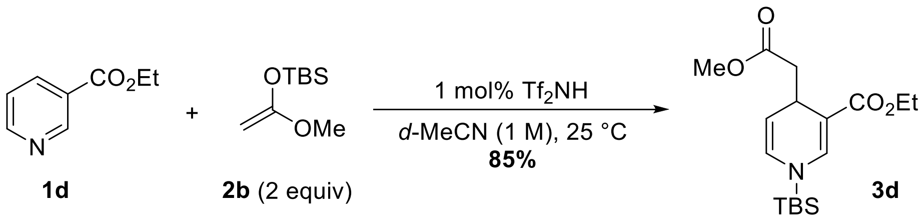

Modification	Yield
$(p\text{-}ClC_6H_4SO_2)_2NH$	79%
$(PhO)_2PO_2H$ or *p*-TsOH	0%
**$(p\text{-}ClC_6H_4SO_2)_2NH$/neat**	**96%**

To a vial charged with the disulfonimide under argon atmosphere was added silyl ketene acetal **2b** followed by the substrate (0.05 mmol). Then, the mixture was stirred overnight at room temperature. The reaction was quenched with 5 µl of triethylamine, dissolved in $CDCl_3$ and analyzed by ^1H NMR. Based on these results, we then designed the novel scaffold.

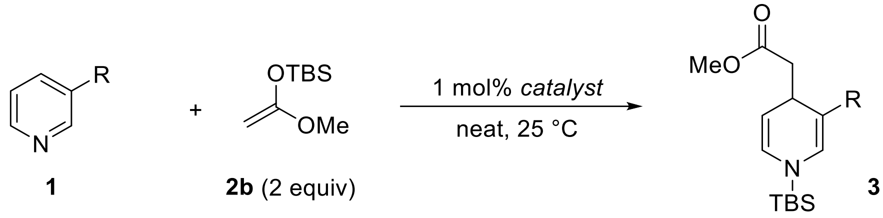

1 + **2b** (2 equiv) → **3**

catalyst:

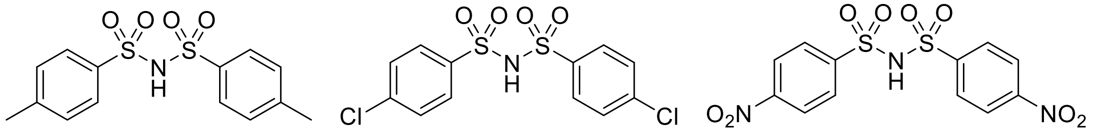

	(p*K*a 12.0)	(p*K*a 10.2)	(p*K*a 8.2)
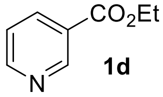 **1d**	29%	96%	92%
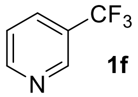 **1f**	0%	12%	68%
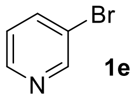 **1e**	0%	16%	28%

To a vial charged with **PhPADI** under argon atmosphere was added silyl ketene acetal **2b** or **2c** followed by the substrate (0.05 mmol). Then, the mixture was stirred overnight at the specified temperature. The reaction was quenched with 5 µl of triethylamine, dissolved in $CDCl_3$ and analyzed by ^{1}H NMR. *Note: several unidentified by-products observed in the case of triflimide. Catalyst analog without 3,3´-substituents proved to be rather unstable.*

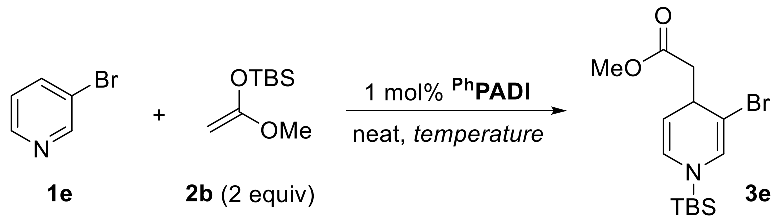

1e + **2b** (2 equiv) → **3e**

49% (25 °C)
29% (25 °C, Tf_2NH)
52% (0 °C)
47% (–20 °C)

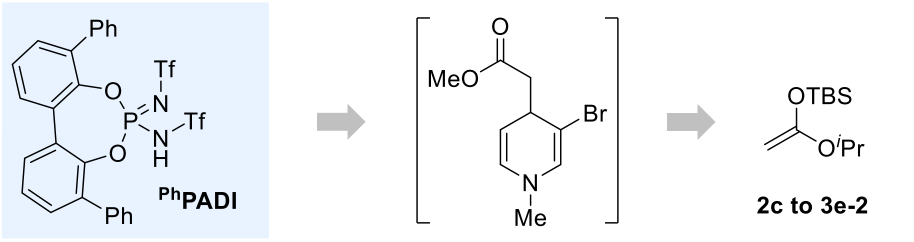

detection by MS

2c to 3e-2

60% (25 °C)
74% (0 °C)
86% (–20 °C)

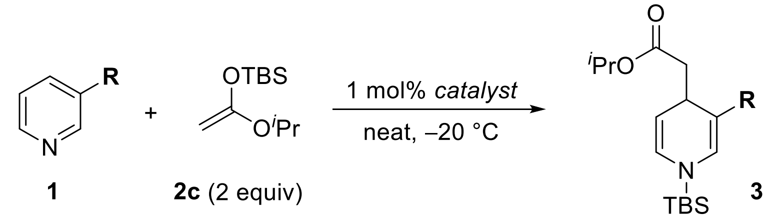

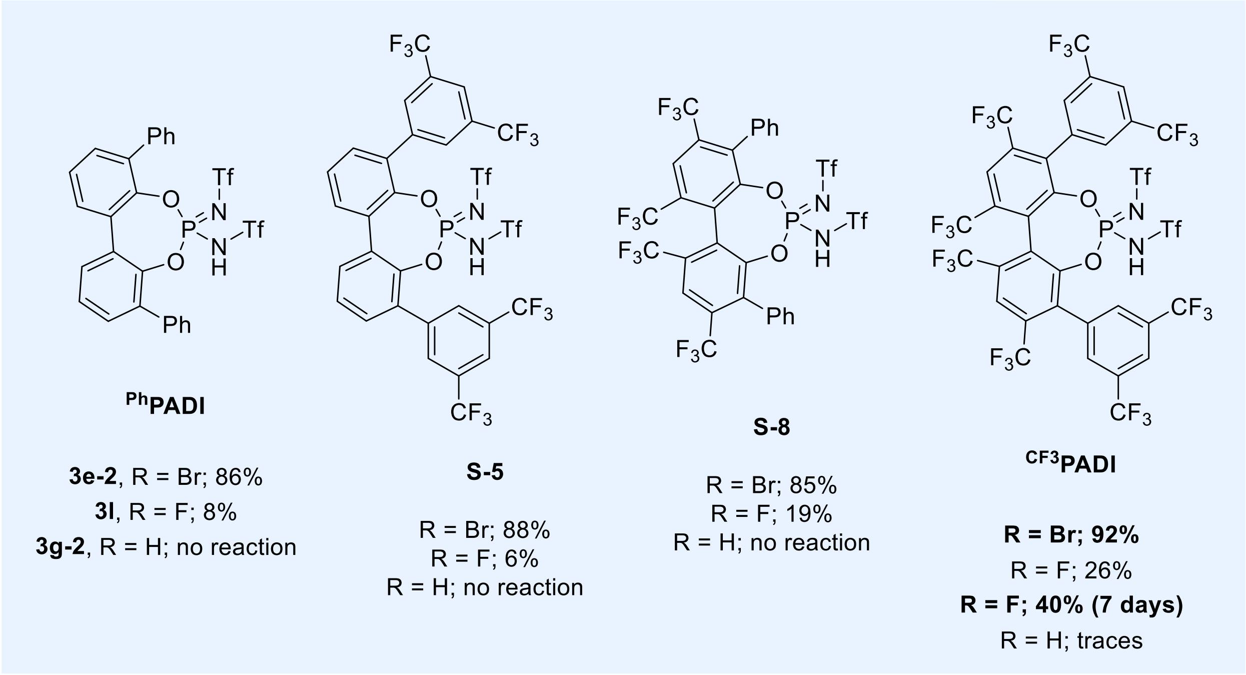

Substrates with *ortho*-substituton are still rather challenging due to arduous coordination of the silylated catalyst in the sterically hindered nitrogen atom. Similarly, C2 addition to *para*-substituted substrates shows little reactivity.

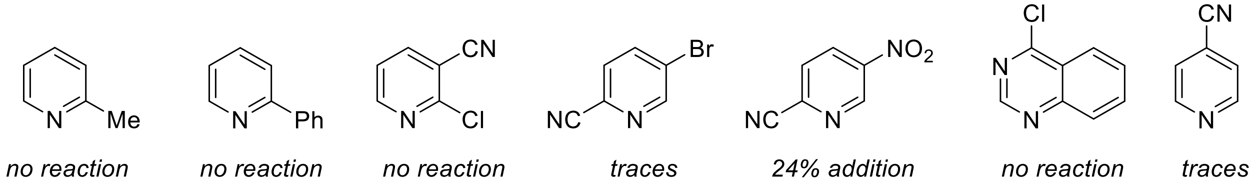

7. Mechanistic Insights

In the optimization studies, no addition of **2a** to **1c** was observed in the absence of catalyst. Otherwise, catalytic Tf_2NH led to complete dearomatization of the substrate towards **3c-2**. Analogous results were obtained with **1a** towards **3a**. *Note: color change is slower without presilylation.*

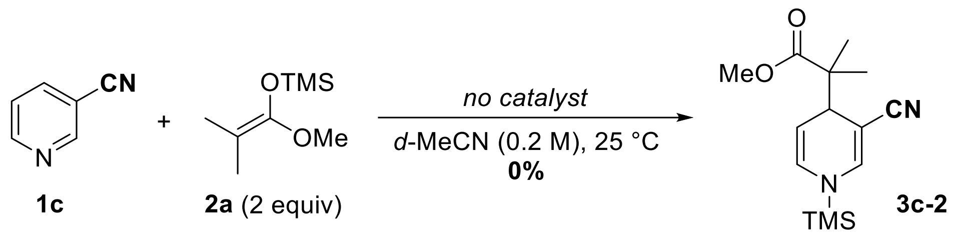

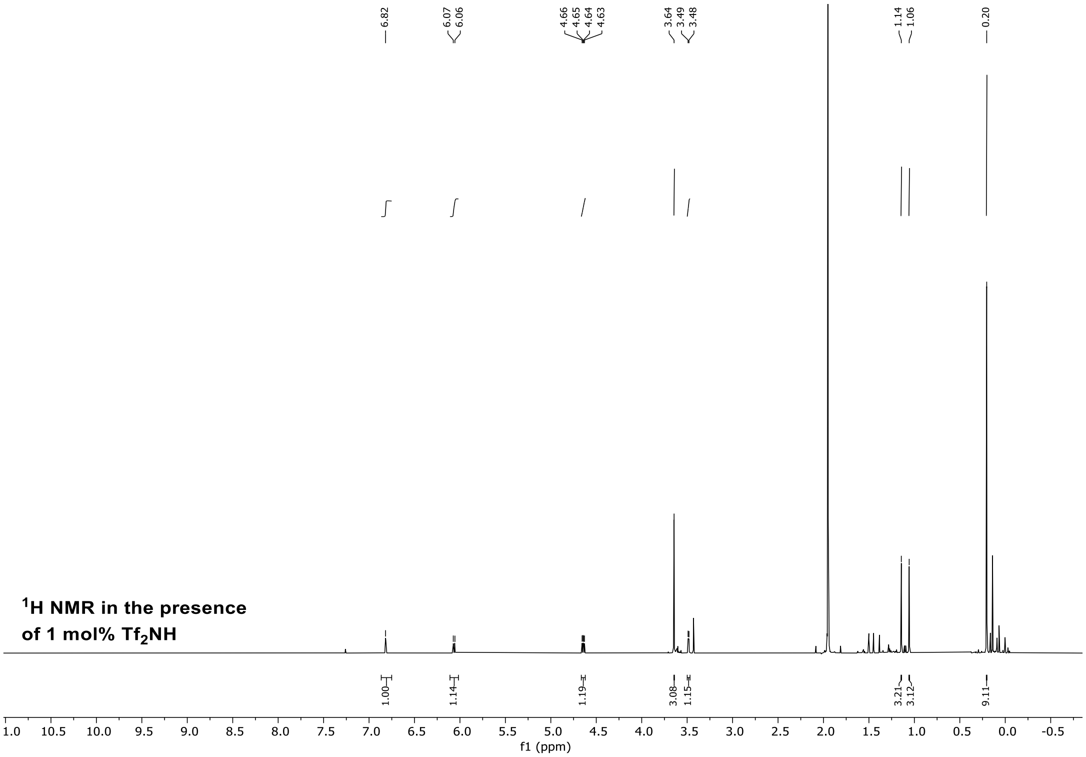

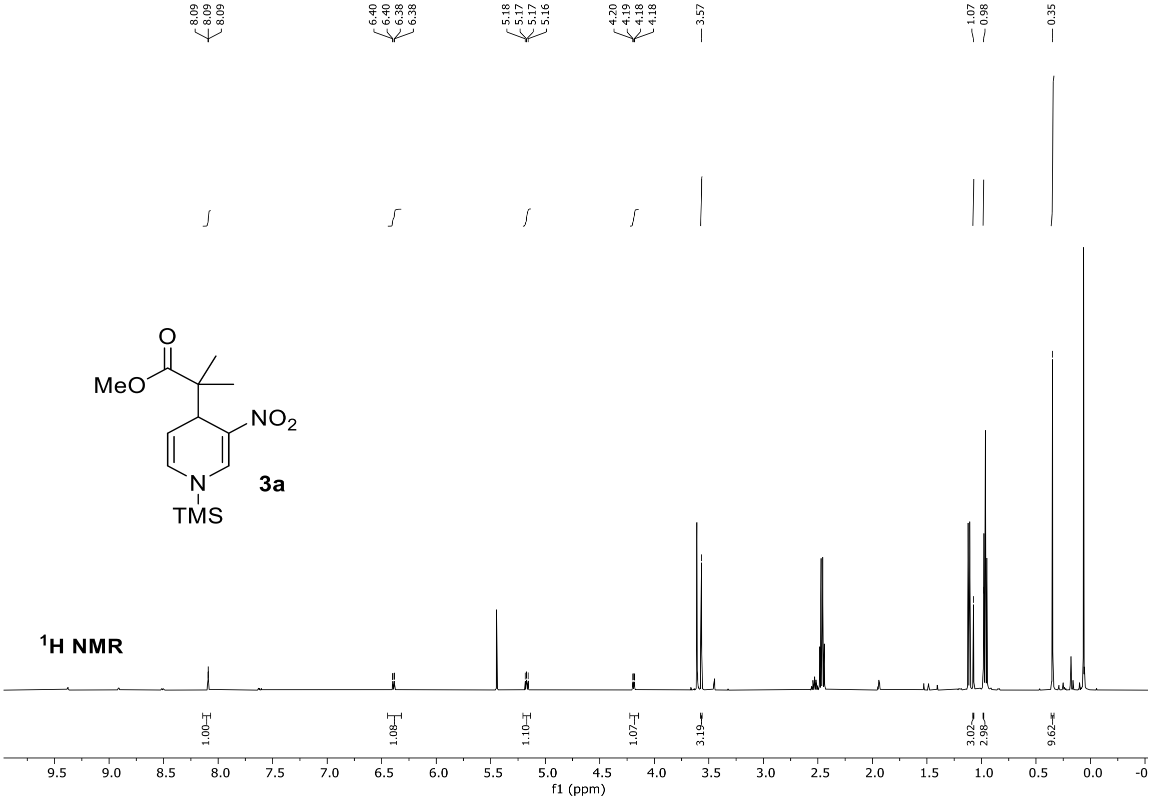

In the case of reaction between **1e** and **2c**, direct analysis of the crude reaction mixture by MS (ESI$^+$) revealed traces of the functionalized *N*–methylated 3-bromodihydropyridine.

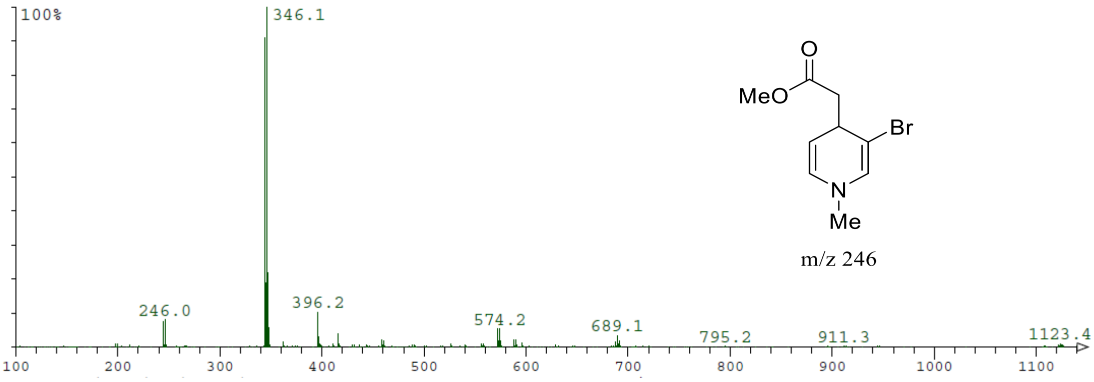

Initial ^{19}F NMR studies suggest that electronically different pyridines interact with the silylated catalyst; Tf_2NH – shown in spectra *(a)* – reacts both with pyridine *via* protonation *(b)* or with trimethylphenylsilane towards Tf_2NTMS *(c)*. In the case of silyl ketene acetal **2a**, the resulting ester partially stabilizes the silicon *(d)* and slowly forms Tf_2NMe.[15] Then, addition of 3-cyanopyridine *(e)*, ethyl nicotinate *(f)* or pyridine *(g)* implied also a slight variation of the chemical shift (**1c**‹**1d**‹**1g**). Furthermore, a well-defined cross-peak in ^{29}Si HMBC was observed in the last example (41.48 ppm in *d*-PhMe), which reasonably corresponds to the substrate coordinated to the activated catalyst. Generation of the proposed intermediate occurs in parallel with the formation of TMS-siloxane, rearranged silyl ketene acetal and TMS-isobutyric acid; but does not lead to the desired addition product **3g-3**.

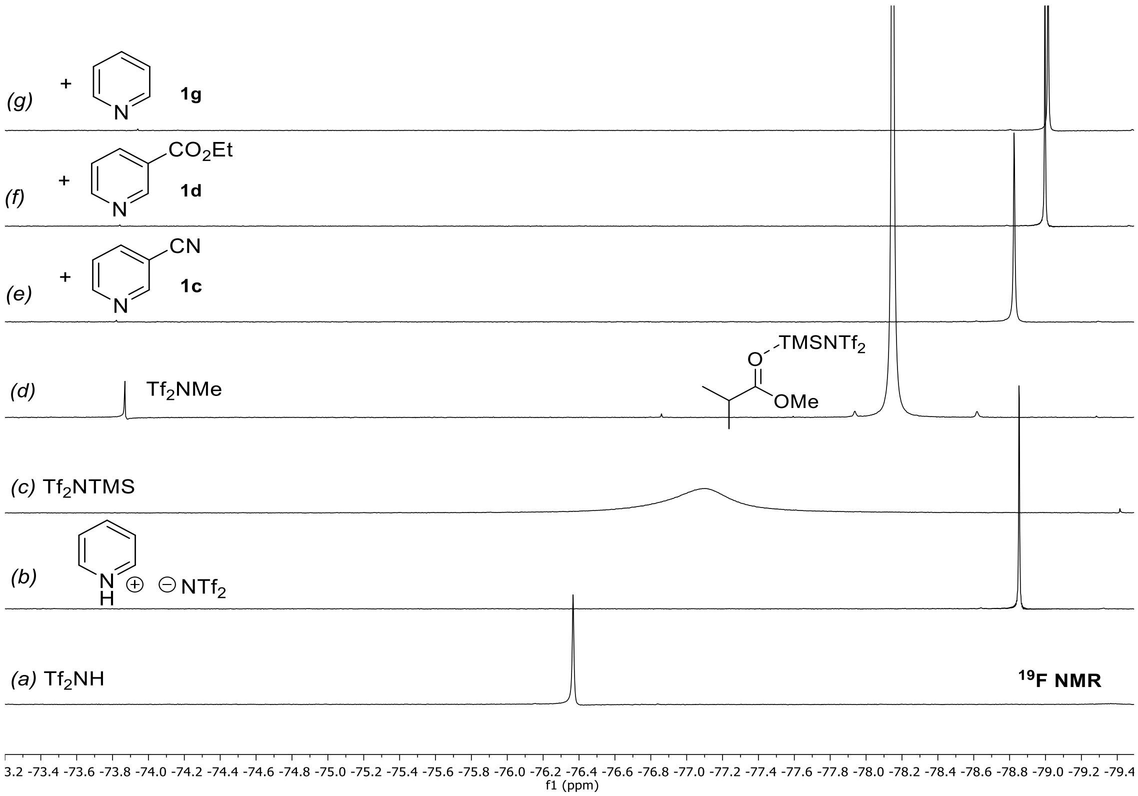

(g) + 1g
(f) + CO2Et 1d
(e) + CN 1c
(d) Tf2NMe
TMSNTf2
OMe
(c) Tf2NTMS
(b) NTf2
(a) Tf2NH
19F NMR
f1 (ppm)

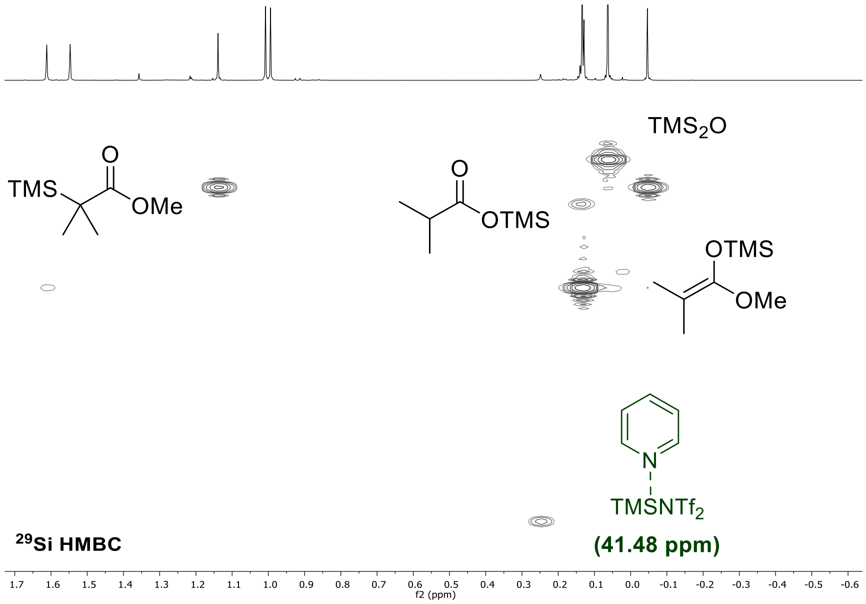

TMS2O
TMS
OMe
OTMS
OTMS
OMe
TMSNTf2
29Si HMBC
(41.48 ppm)
f2 (ppm)

8. References

[1] S. Lee, P. S. J. Kaib, B. List, *Synlett* **2017**, *28*, 1478–1480.
[2] Y. Jeong, J. Ban, M. Lim, H. Rhee, *Synthesis* **2018**, *50*, 1867–1874.
[3] W. L. F. Armarego, C. L. L. Chai, Purification of Laboratory Chemicals, Oxford (2003).
[4] G. R. Fulmer, A. J. M. Miller, N. H. Sherden, H. E. Gottlieb, A. Nudelman, B. M. Stoltz, J. E. Bercaw, K. I. Goldberg, *Organometallics* **2010**, *29*, 2176–2179.
[5] H. E. Gottlieb, V. Kotlyar, A. Nudelman, *J. Org. Chem.* **1997**, *62*, 7512–7515.
[6] R. K. Harris, E. D. Becker, S. M. Cabral de Menezes, P. Granger, R. E. Hoffman, K. W. Zilm, *Pure Appl. Chem.* **2008**, *80*, 59–84.
[7] T. Gatzenmeier, P. S. J. Kaib, J. B. Lingnau, R. Goddard, B. List, *Angew. Chem. Int. Ed.* **2018**, *57*, 2464–2468.
[8] N. Frueh, A. Togni. *Angew. Chem. Int. Ed.* **2014**, *53*, 10813–10816.
[9] P. Breant, F. Marsais, G. Queguiner. *Synthesis* **1983**, 822–824.
[10] C. Anderson, J. Moreno, S. Hadida. *Synlett* **2014**, *25*, 677–680.
[11] I. E. Nifant'ev, P. V. Ivchenko, V. V. Bagrov, S. M. Nagy, S. Mihan, L. N. Winslow, A. V. Churakov, *Organometallics* **2013**, *32*, 2685–2692.
[12] X. Xiong, Y. Y. Yeung, *ACS Catal.* **2018**, *8*, 4033–4043.
[13] D. J. Frank, A. Franzke, A. Pfaltz, *Chem. Eur. J.* **2013**, *19*, 2405–2415.
[14] M. Peifer, R. Berger, V. W. Shurtleff, J. C. Conrad, D. W. C. MacMillan. *J. Am. Chem. Soc.* **2014**, *136*, 5900–5903.
[15] Z. Zhang, H. Y. Bae, J. Guin, C. Rabalakos, M. van Gemmeren, M. Klussmann, B. List, B. *Nat. Commun.* **7**, 12478 (2016).

9. Spectroscopic Data

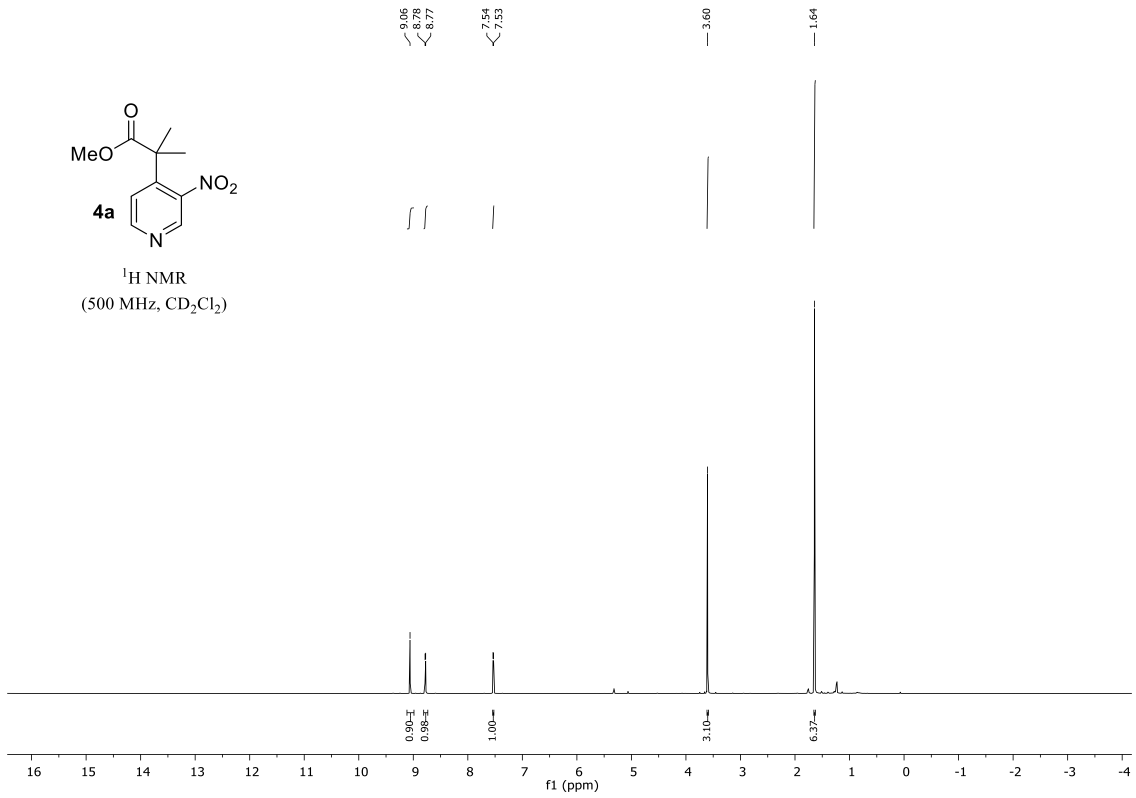

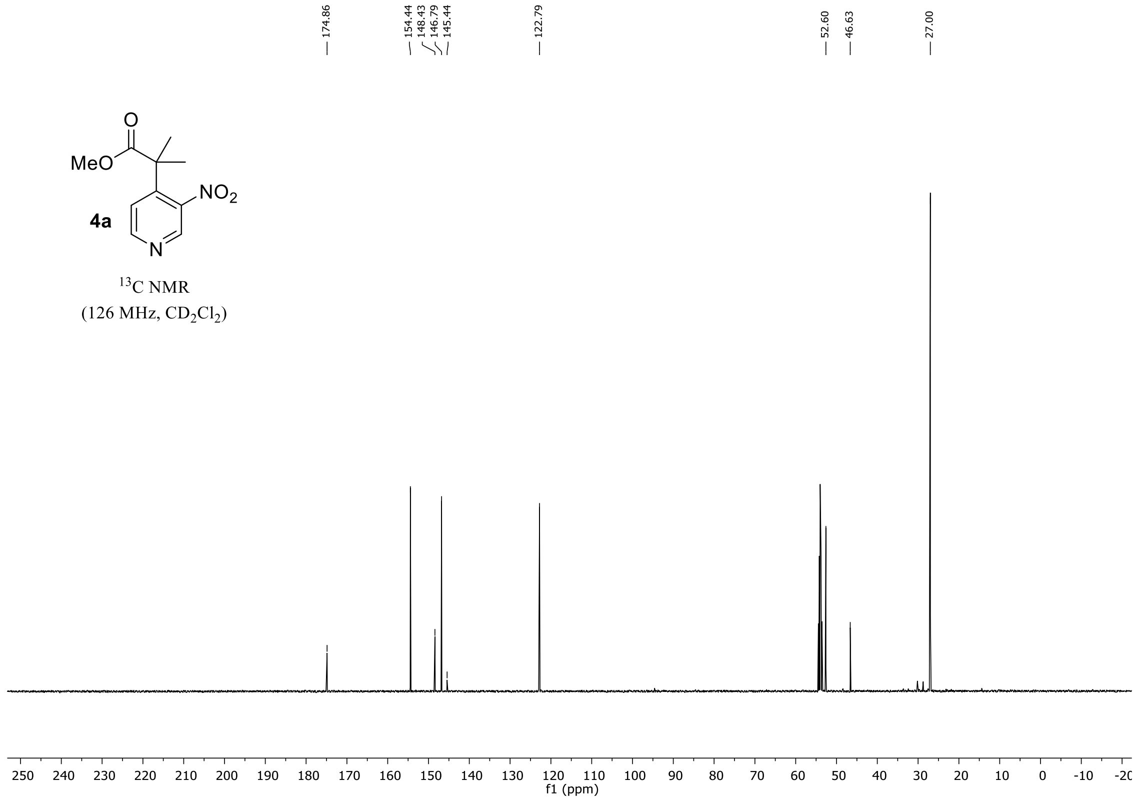

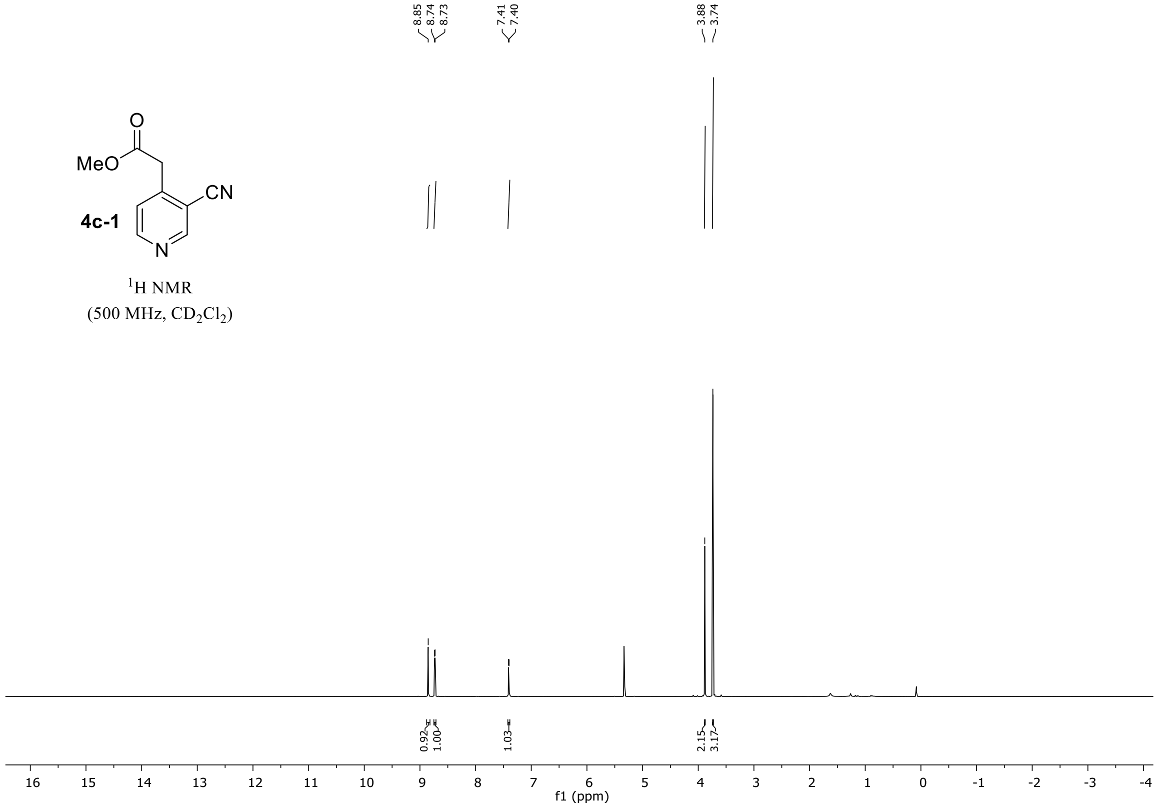
8.85
8.74
8.73
7.41
7.40
3.88
3.74
MeO
O
CN
N
4c-1
1H NMR
(500 MHz, CD2Cl2)
0.92
1.00
1.03
2.15
3.17
16 15 14 13 12 11 10 9 8 7 6 5 4 3 2 1 0 -1 -2 -3 -4
f1 (ppm)

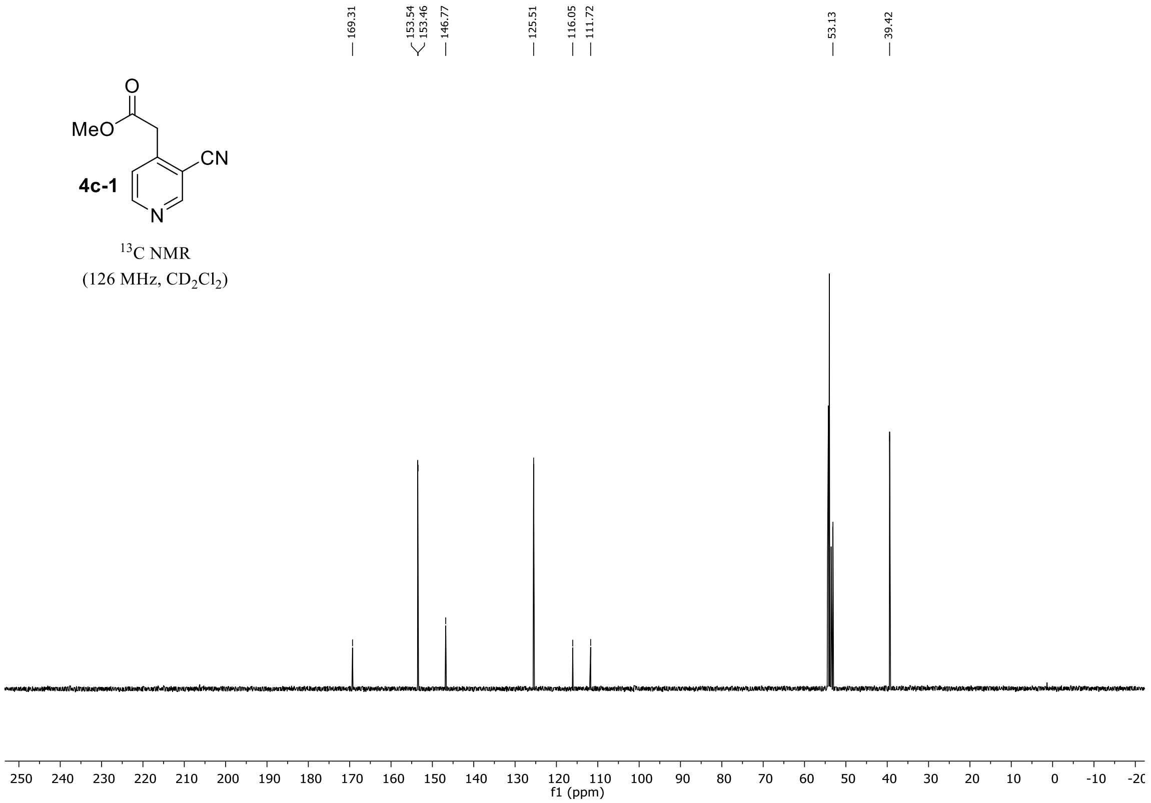
169.31
153.54
153.46
146.77
125.51
116.05
111.72
53.13
39.42
MeO
O
CN
N
4c-1
13C NMR
(126 MHz, CD2Cl2)
250 240 230 220 210 200 190 180 170 160 150 140 130 120 110 100 90 80 70 60 50 40 30 20 10 0 -10 -20
f1 (ppm)

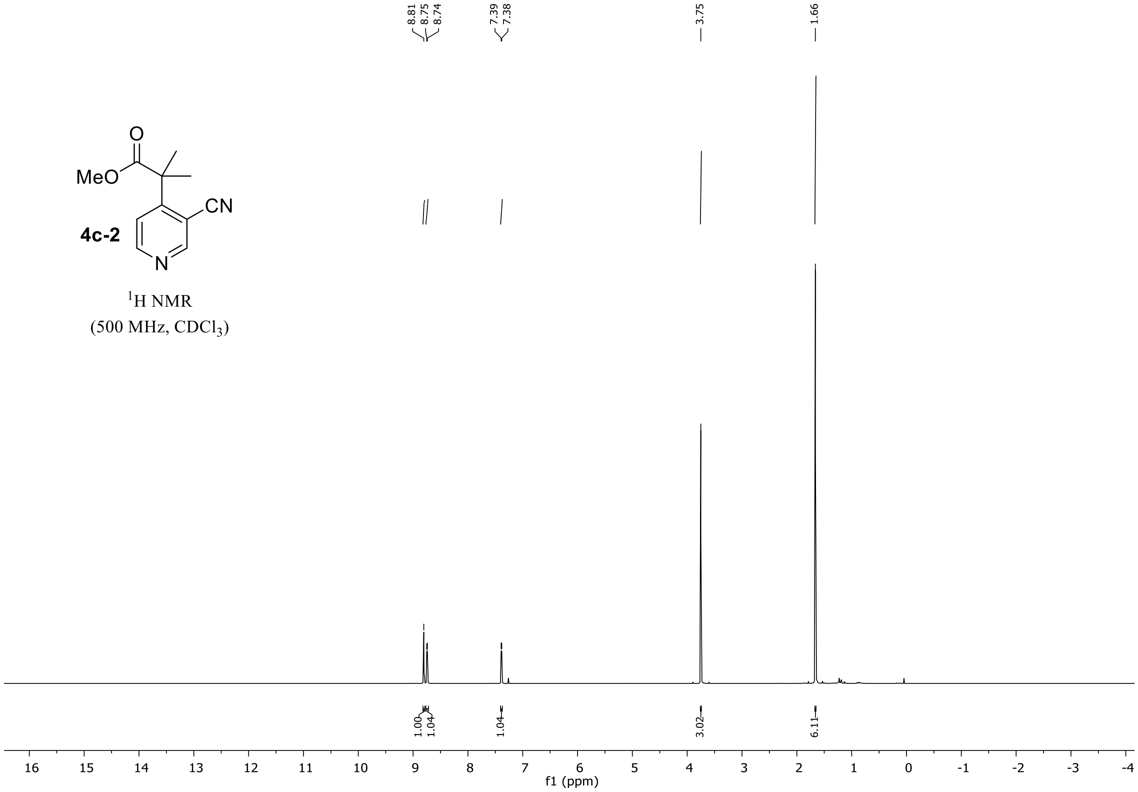
MeO
O
CN
N
4c-2
^{1}H NMR
(500 MHz, $CDCl_3$)
8.81
8.75
8.74
7.39
7.38
3.75
1.66
1.00
1.04
1.04
3.02
6.11
f1 (ppm)

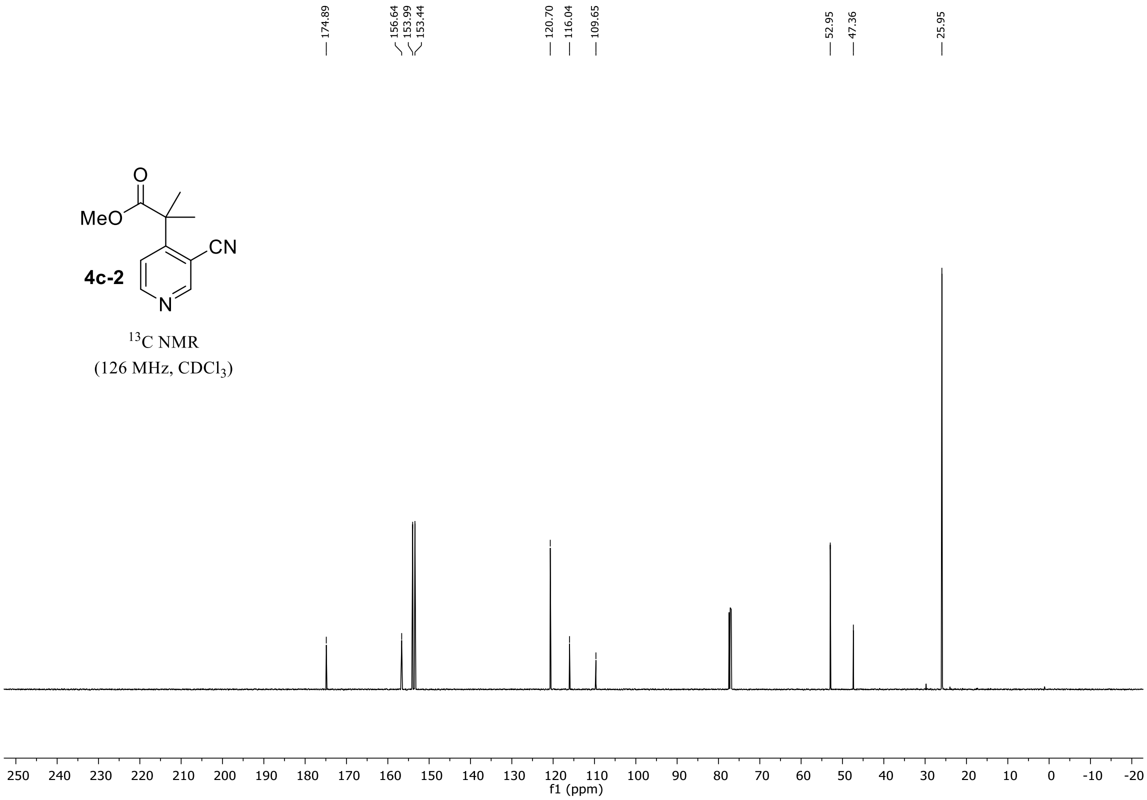
MeO
O
CN
N
4c-2
^{13}C NMR
(126 MHz, $CDCl_3$)
174.89
156.64
153.99
153.44
120.70
116.04
109.65
52.95
47.36
25.95
f1 (ppm)

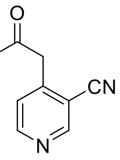

4c-3

^{1}H NMR
(500 MHz, CD_2Cl_2)

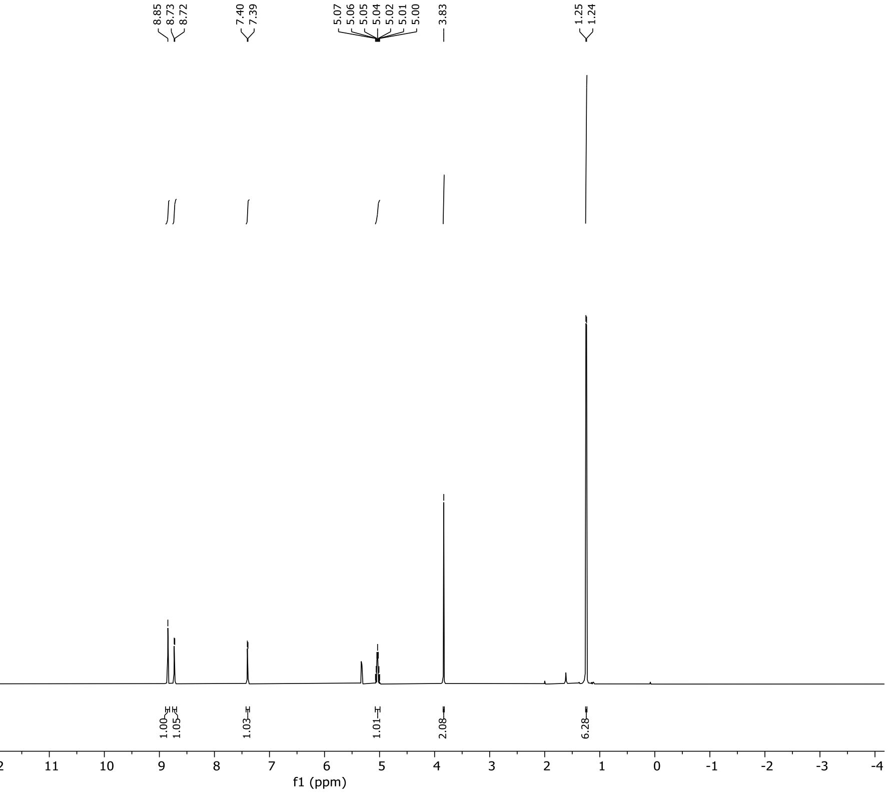

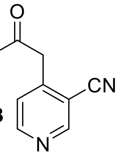

4c-3

^{13}C NMR
(126 MHz, CD_2Cl_2)

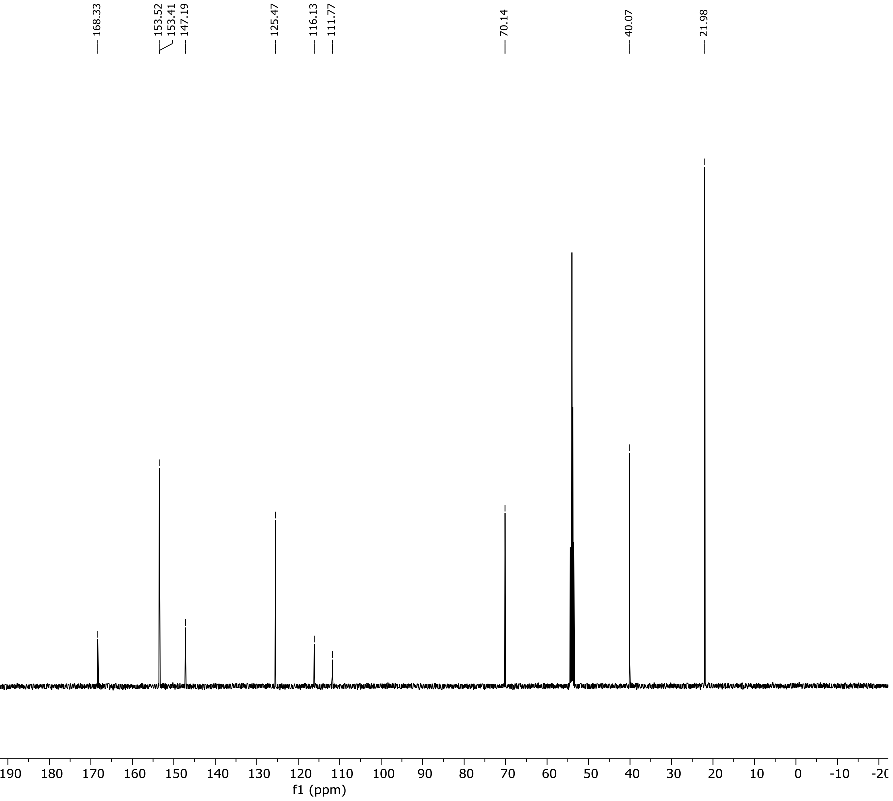

4c-4

^{1}H NMR
(500 MHz, $CDCl_3$)

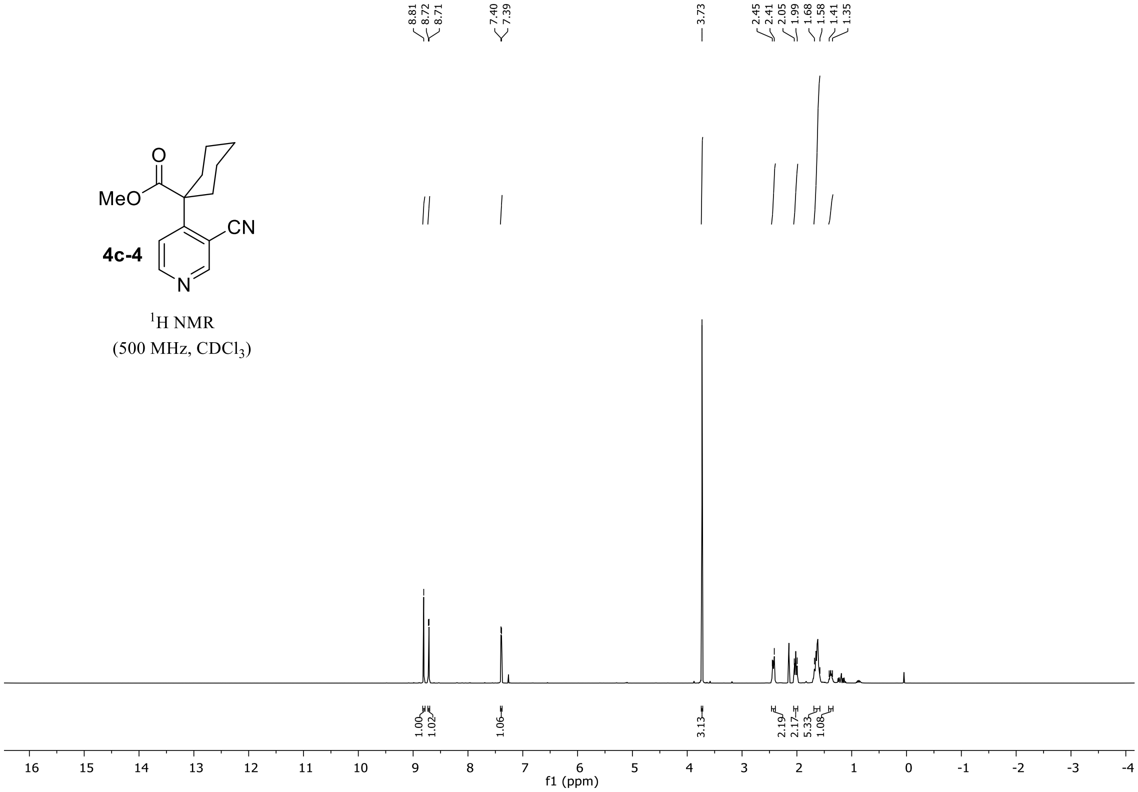

4c-4

^{13}C NMR
(126 MHz, $CDCl_3$)

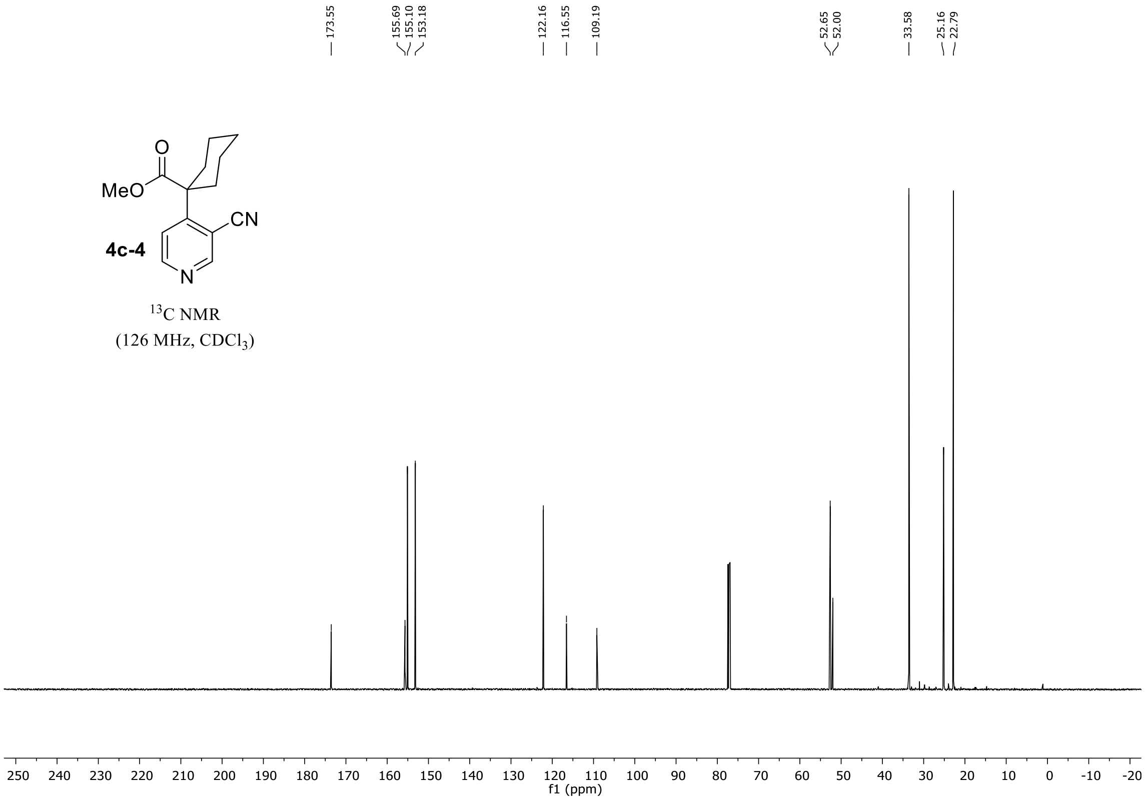

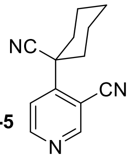

4c-5

^{1}H NMR
(500 MHz, $CDCl_3$)

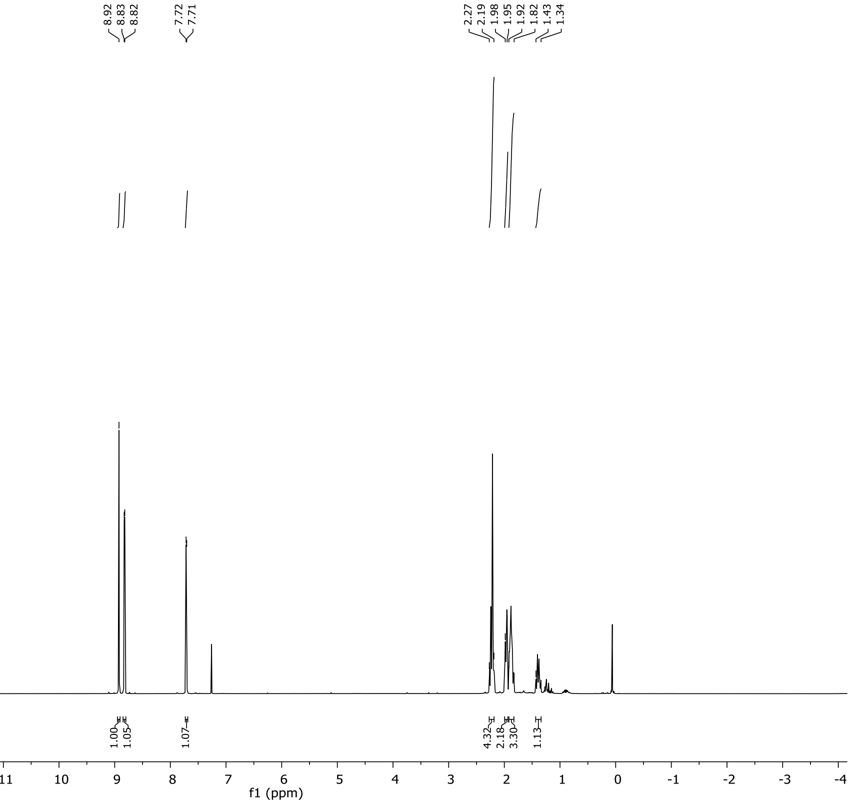

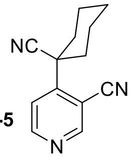

4c-5

^{13}C NMR
(126 MHz, $CDCl_3$)

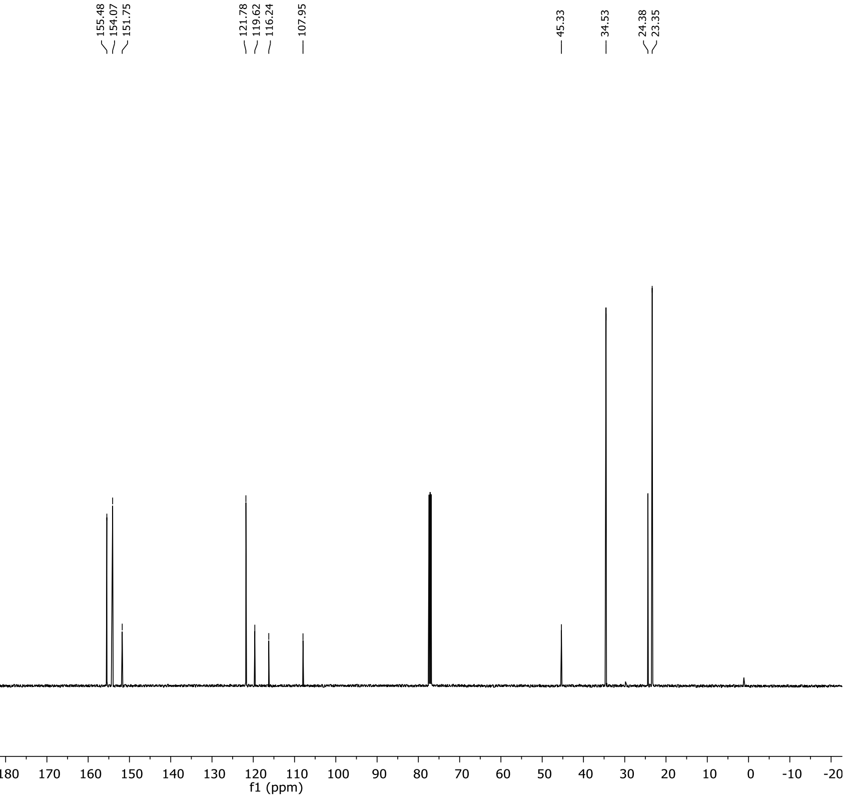

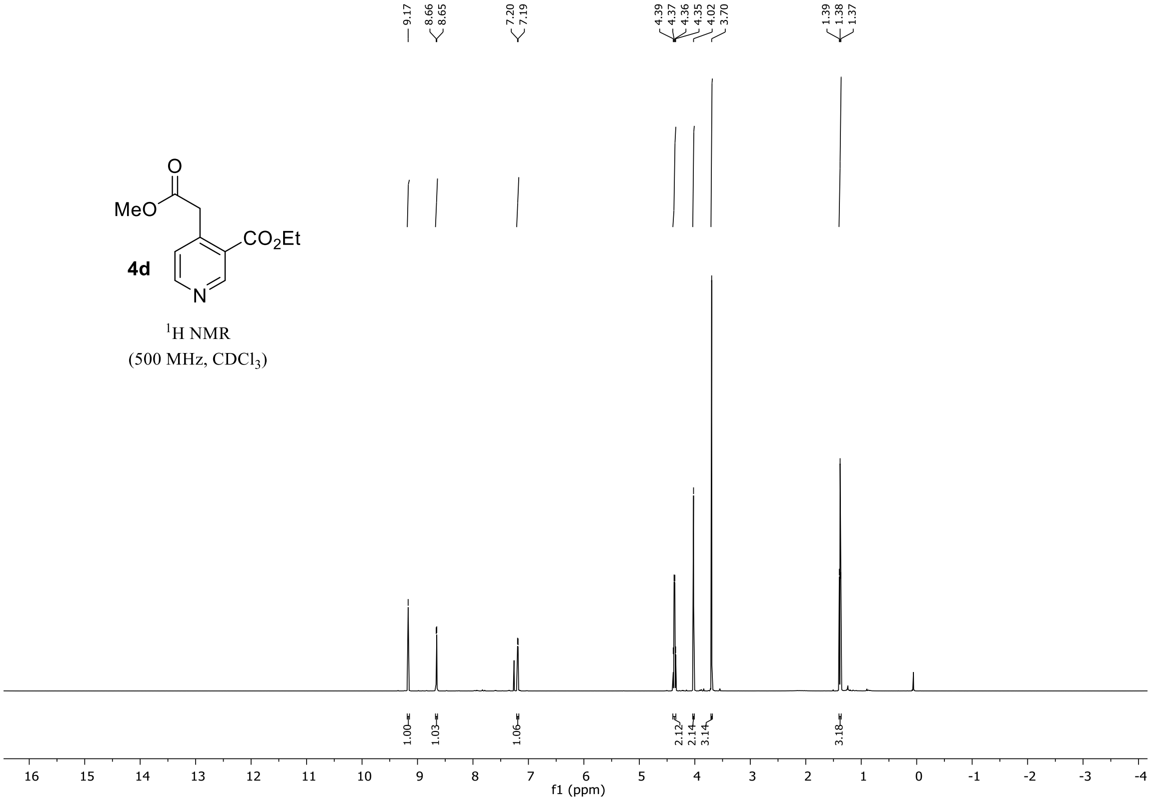
MeO
O
CO_2Et
N
4d
^{1}H NMR
(500 MHz, $CDCl_3$)
9.17 8.66 8.65 7.20 7.19 4.39 4.37 4.36 4.35 4.02 3.70 1.39 1.38 1.37
1.00 1.03 1.06 2.12 2.14 3.14 3.18
f1 (ppm)

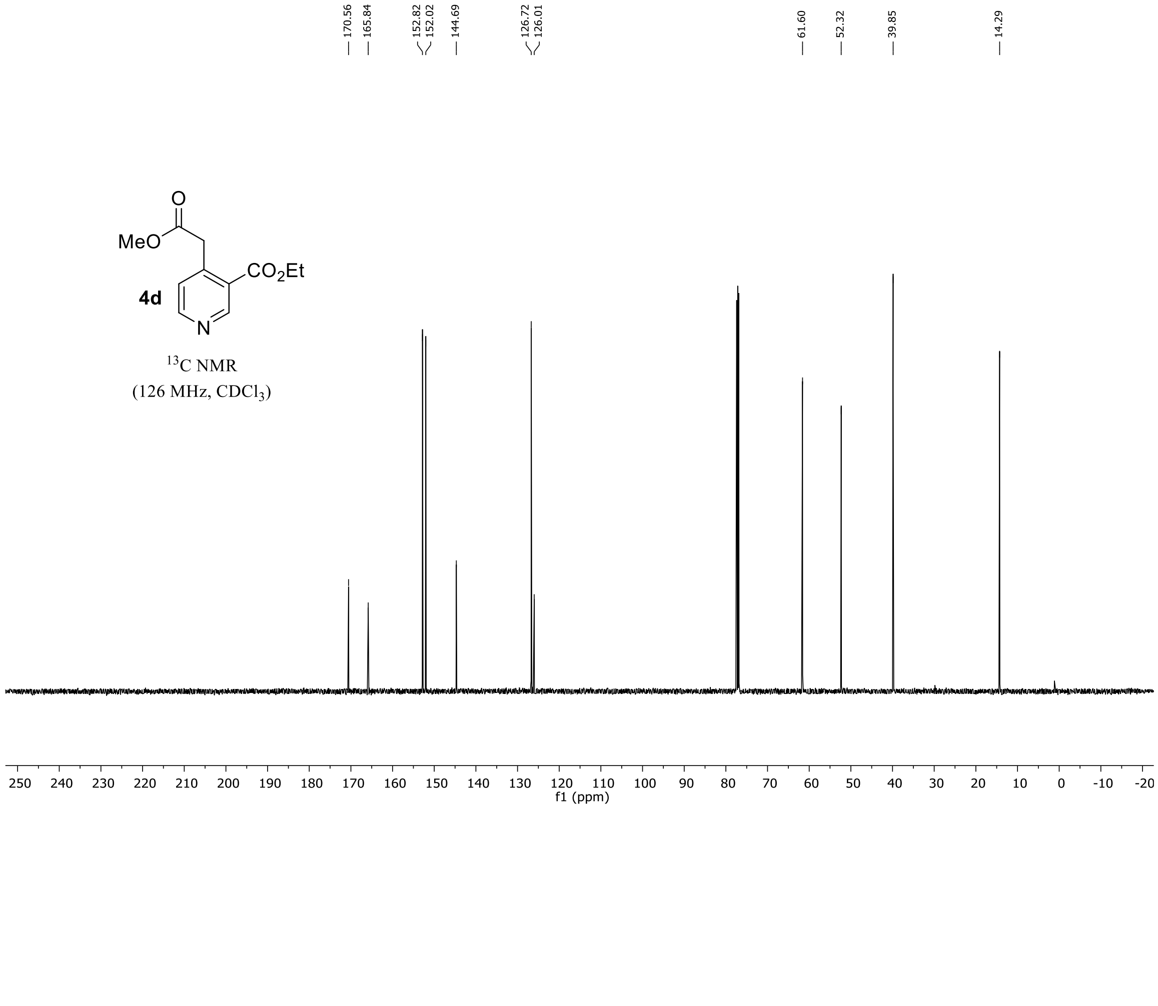
MeO
O
CO_2Et
N
4d
^{13}C NMR
(126 MHz, $CDCl_3$)
170.56 165.84 152.82 152.02 144.69 126.72 126.01 61.60 52.32 39.85 14.29
f1 (ppm)

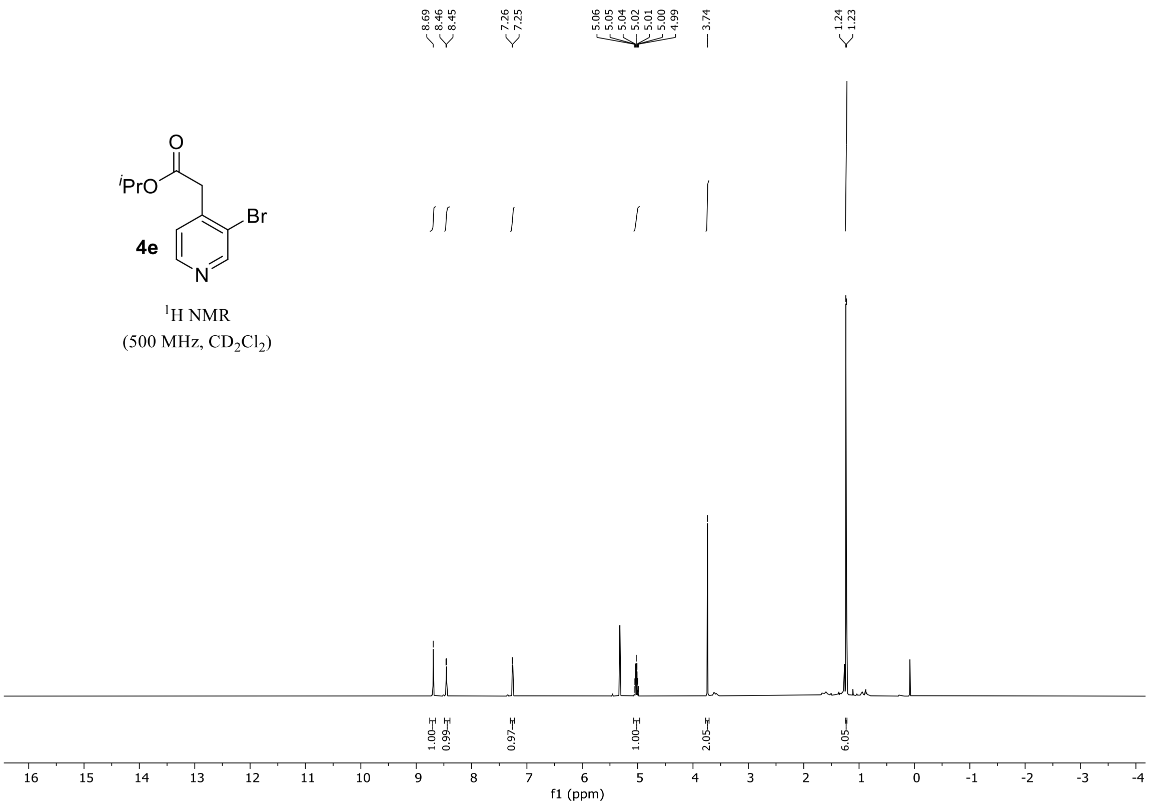

4e

^{1}H NMR
(500 MHz, CD_2Cl_2)

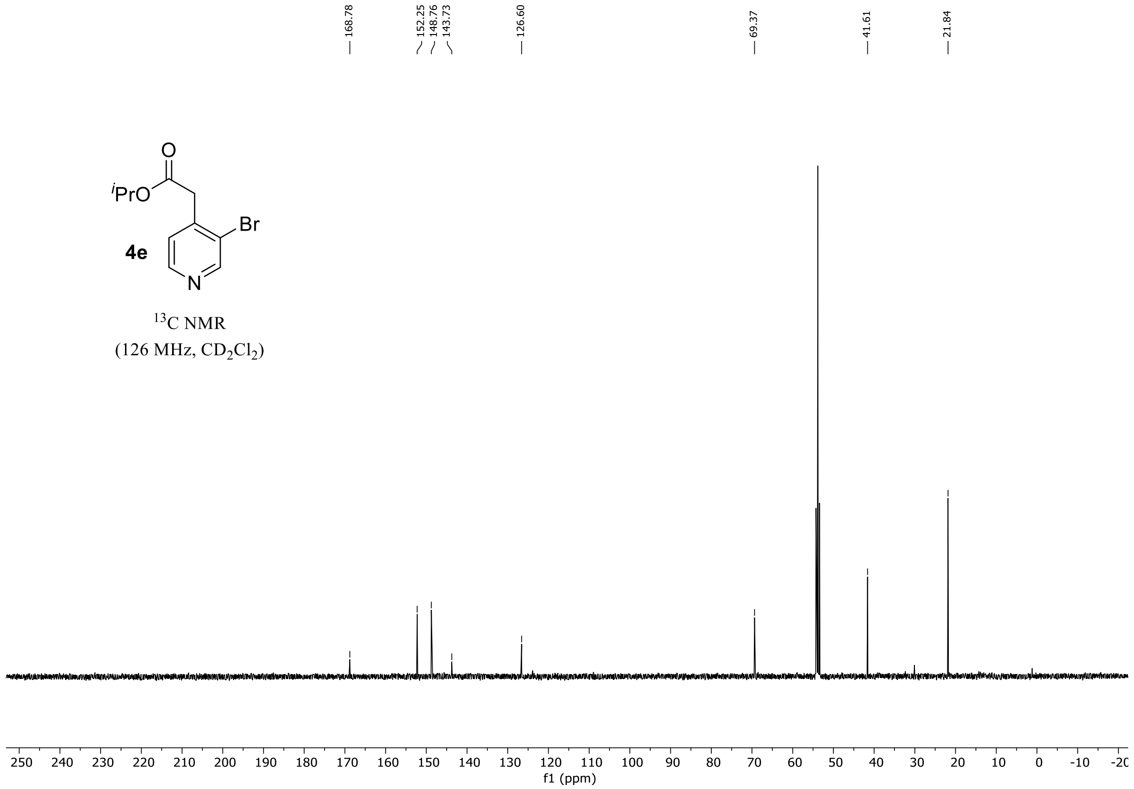

4e

^{13}C NMR
(126 MHz, CD_2Cl_2)

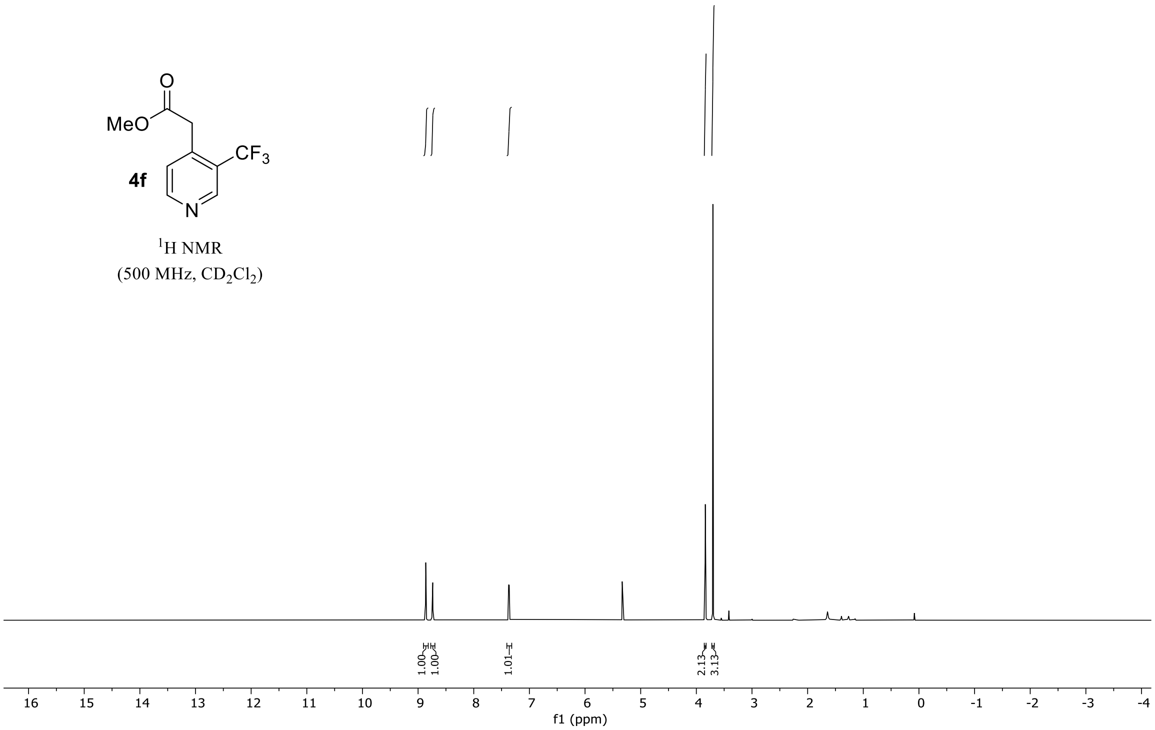

O
MeO
CF3
4f
N
1H NMR
(500 MHz, CD2Cl2)
1.00
1.00
1.01
2.13
3.13
16 15 14 13 12 11 10 9 8 7 6 5 4 3 2 1 0 -1 -2 -3 -4
f1 (ppm)

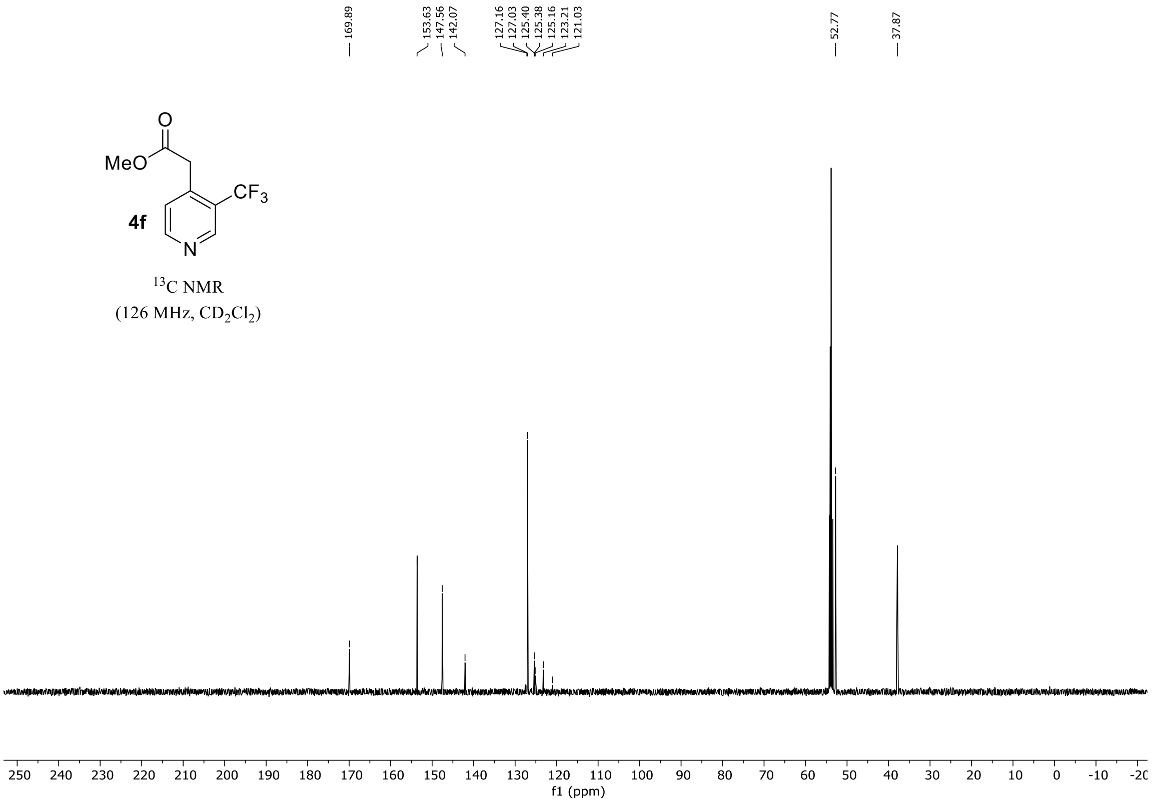

169.89
153.63
147.56
142.07
127.16
127.03
125.40
125.38
125.16
123.21
121.03
52.77
37.87
O
MeO
CF3
4f
N
13C NMR
(126 MHz, CD2Cl2)
250 240 230 220 210 200 190 180 170 160 150 140 130 120 110 100 90 80 70 60 50 40 30 20 10 0 -10 -20
f1 (ppm)

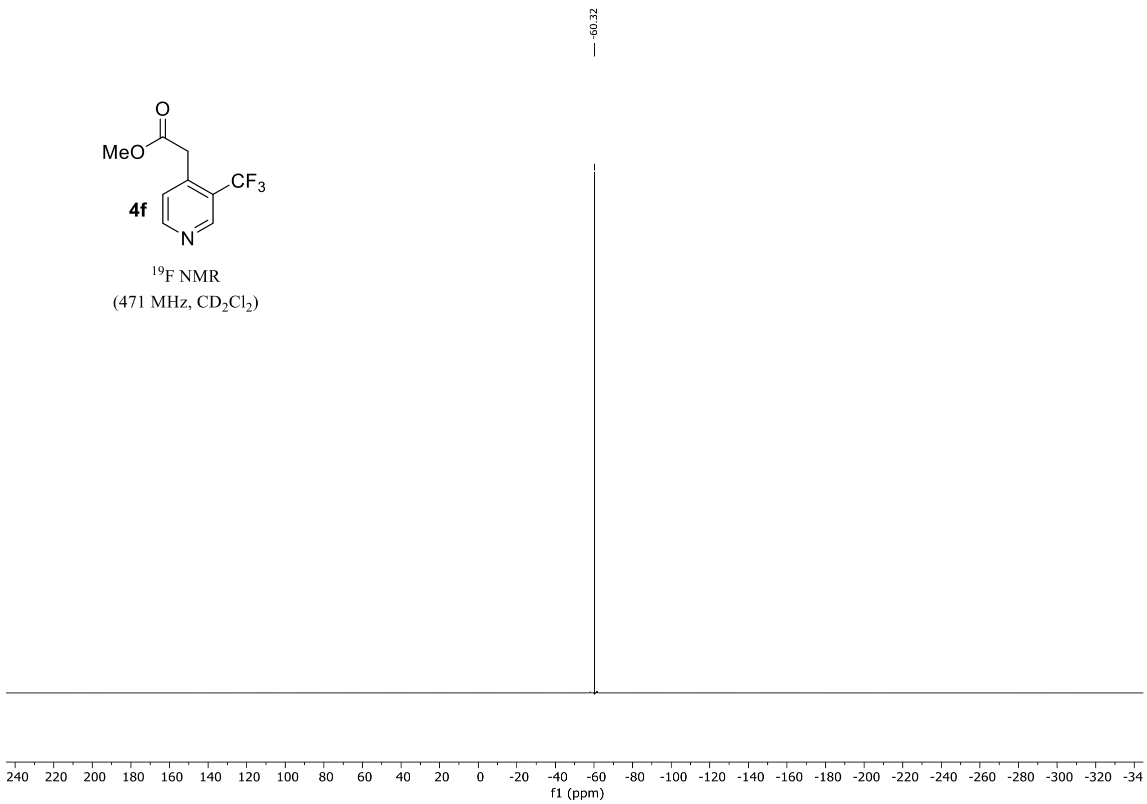

-60.32
MeO
O
CF3
4f
N
19F NMR
(471 MHz, CD2Cl2)
240 220 200 180 160 140 120 100 80 60 40 20 0 -20 -40 -60 -80 -100 -120 -140 -160 -180 -200 -220 -240 -260 -280 -300 -320 -34
f1 (ppm)

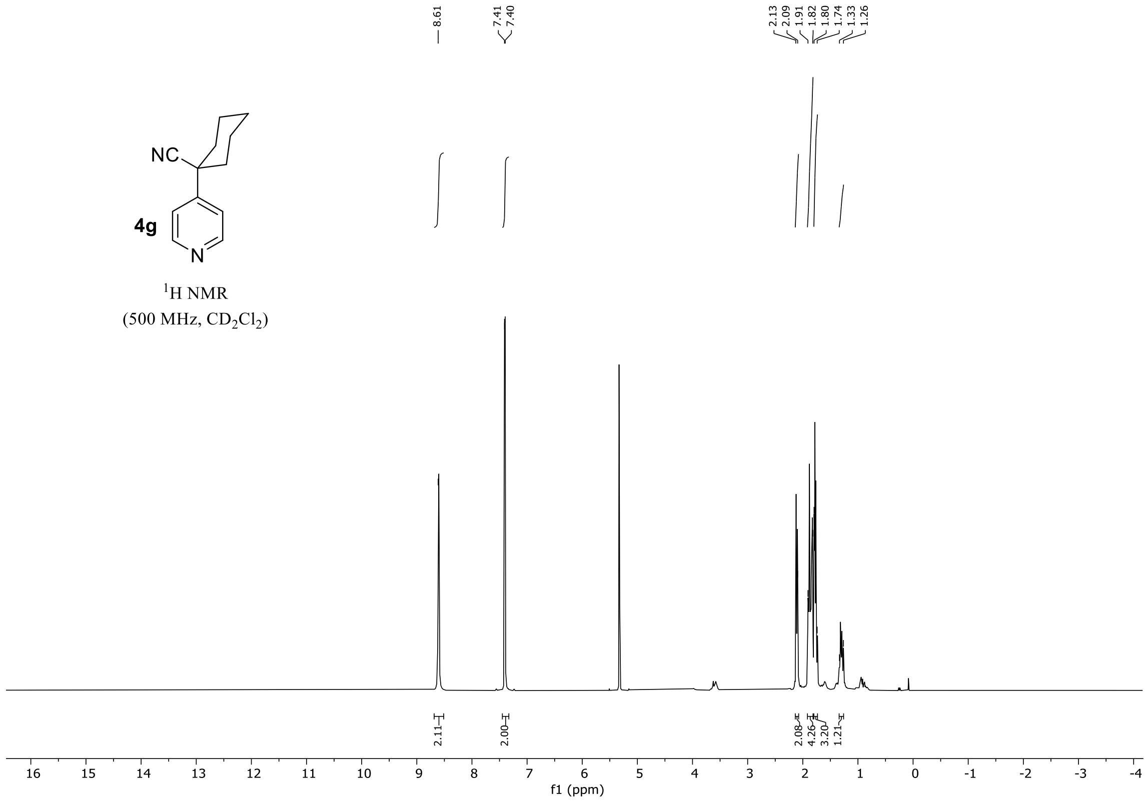

8.61
7.41
7.40
2.13
2.09
1.91
1.82
1.80
1.74
1.33
1.26
NC
4g
N
1H NMR
(500 MHz, CD2Cl2)
2.11
2.00
2.08
4.26
3.20
1.21
16 15 14 13 12 11 10 9 8 7 6 5 4 3 2 1 0 -1 -2 -3 -4
f1 (ppm)

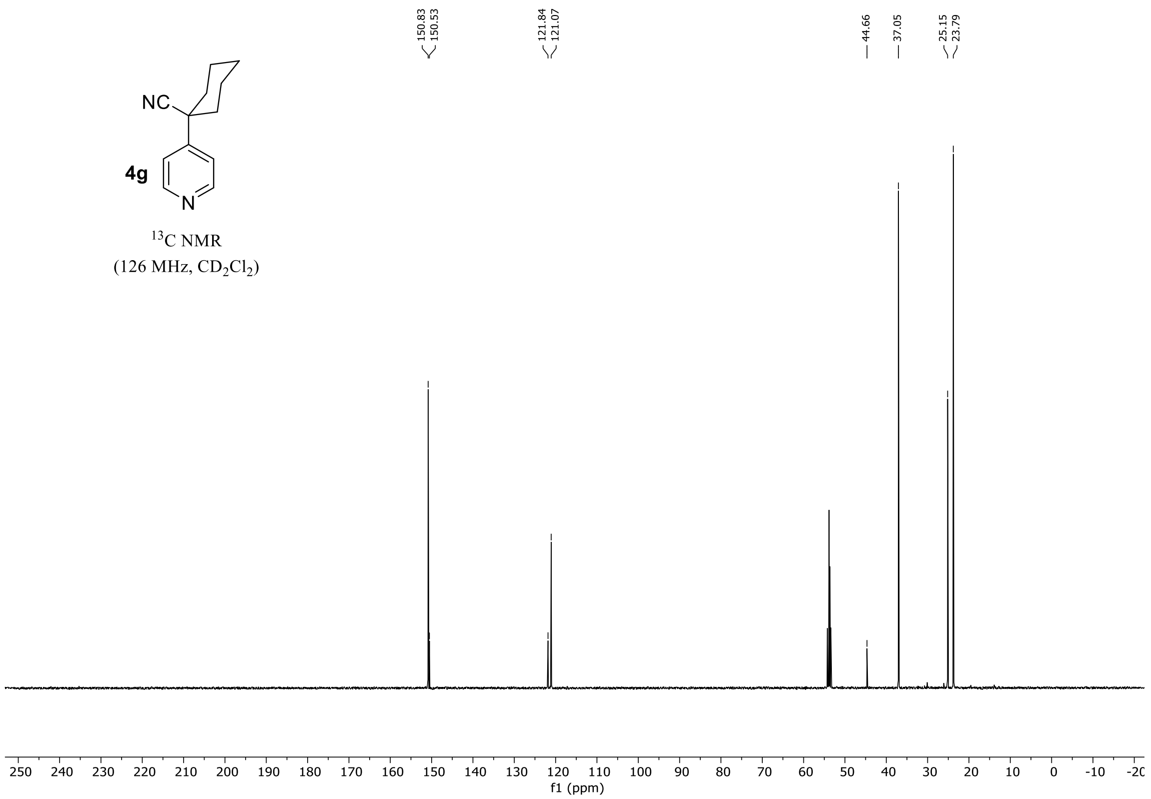

4g

^{13}C NMR
(126 MHz, CD_2Cl_2)

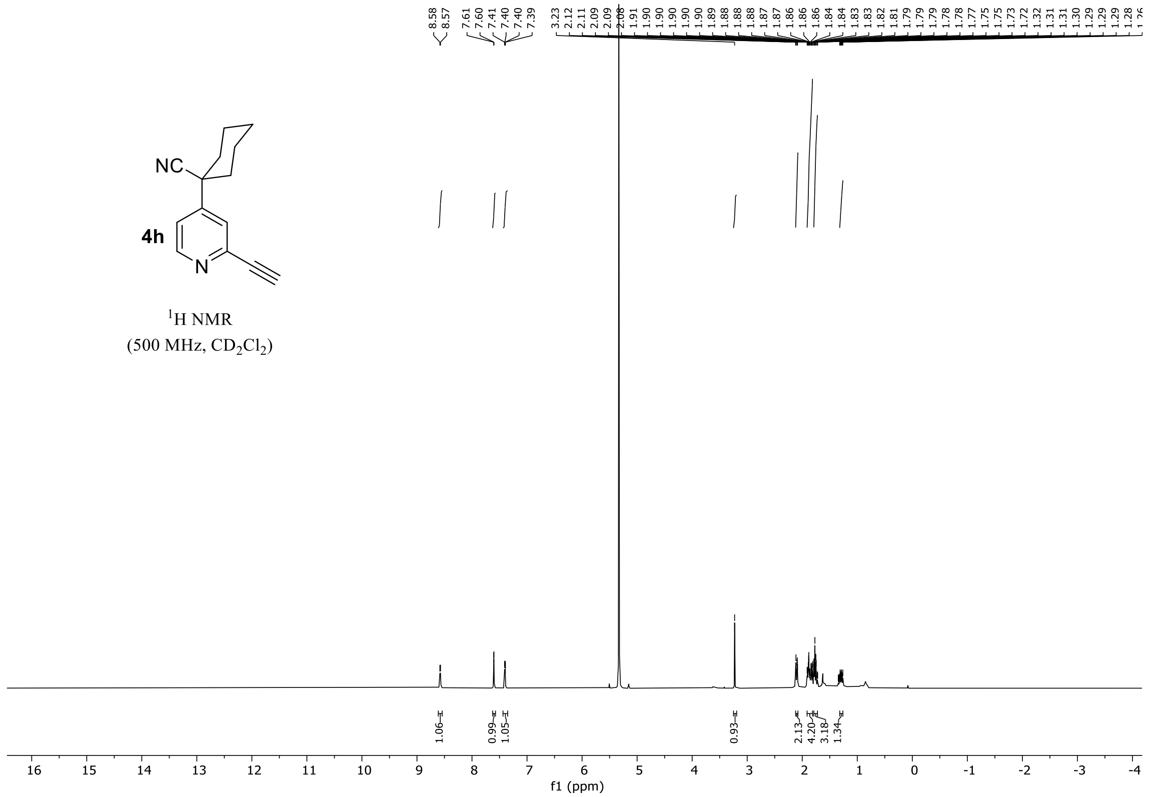

4h

^{1}H NMR
(500 MHz, CD_2Cl_2)

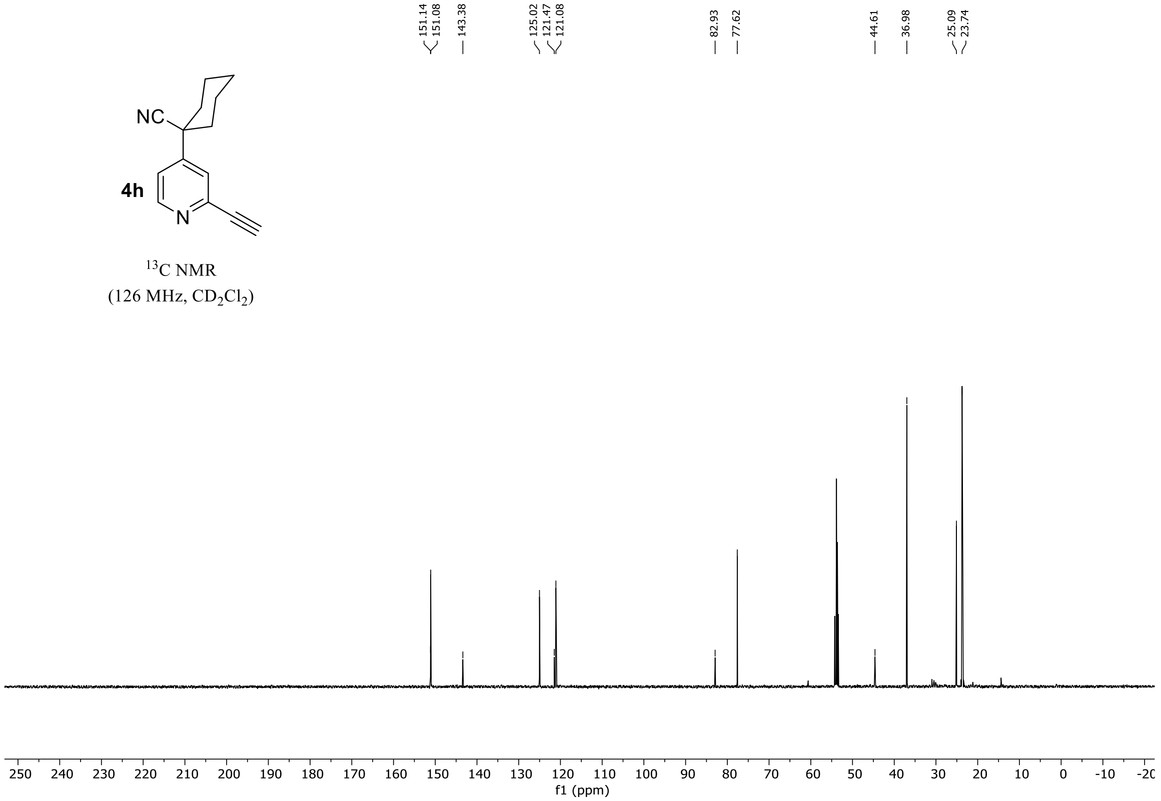

^{13}C NMR
(126 MHz, CD_2Cl_2)

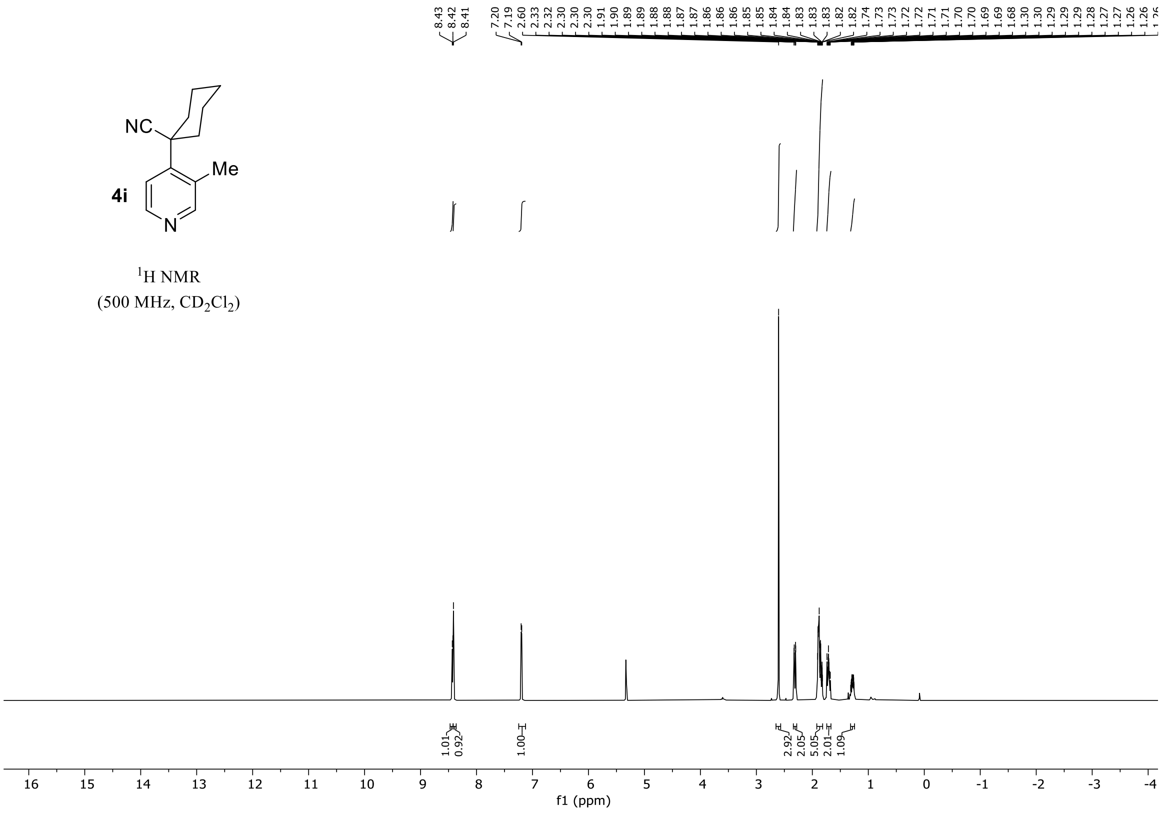

^{1}H NMR
(500 MHz, CD_2Cl_2)

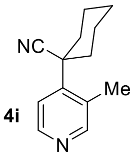

4i

^{13}C NMR
(126 MHz, CD_2Cl_2)

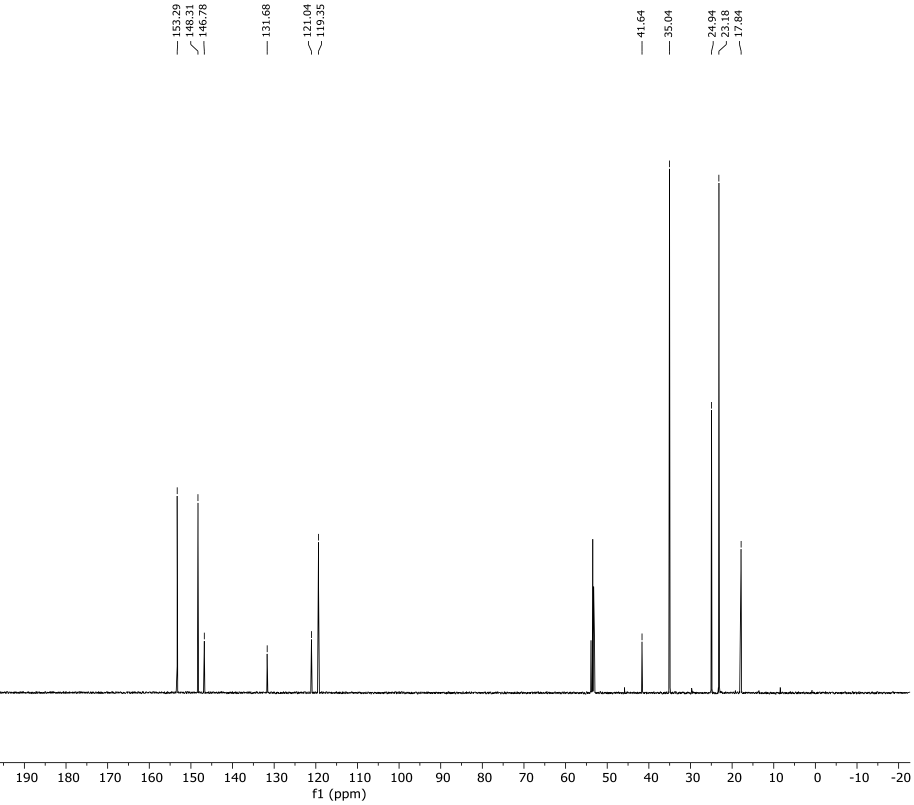

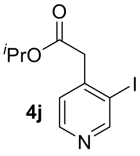

4j

^1H NMR
(500 MHz, CD_2Cl_2)

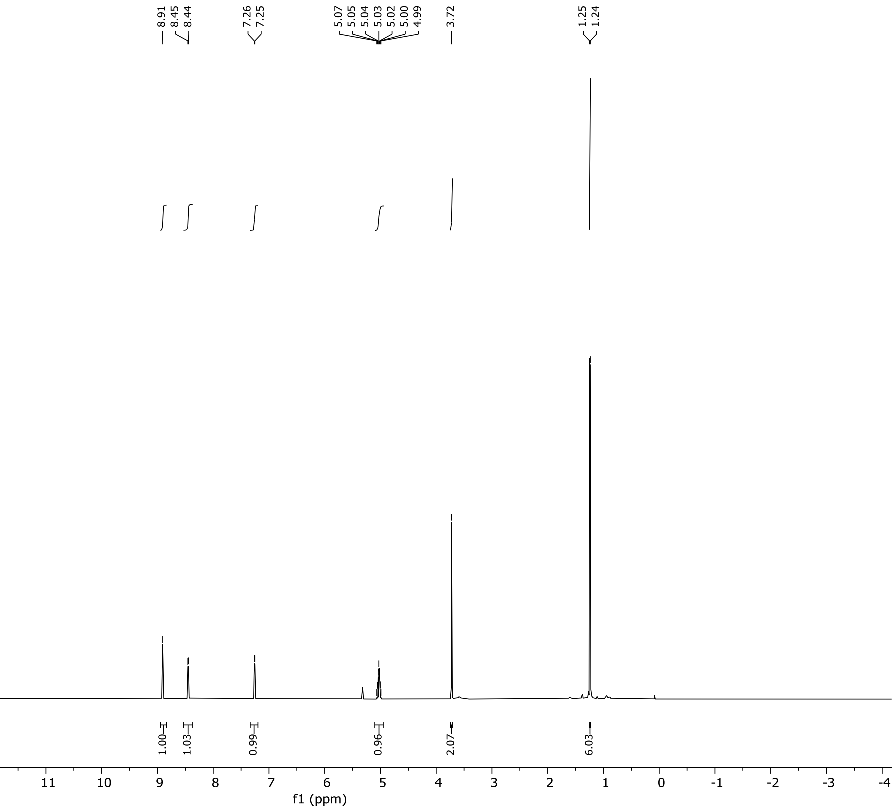

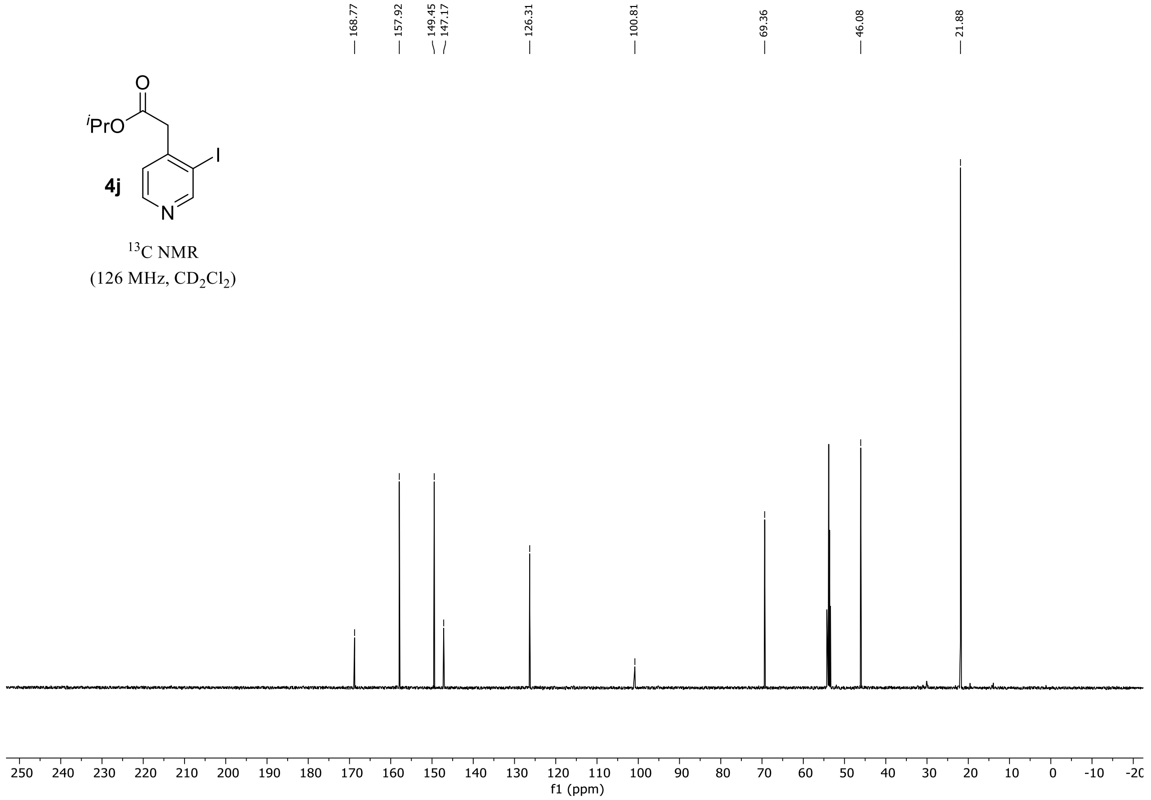
168.77
157.92
149.45
147.17
126.31
100.81
69.36
46.08
21.88
ⁱPrO
O
I
4j
N
^{13}C NMR
(126 MHz, CD_2Cl_2)
f1 (ppm)

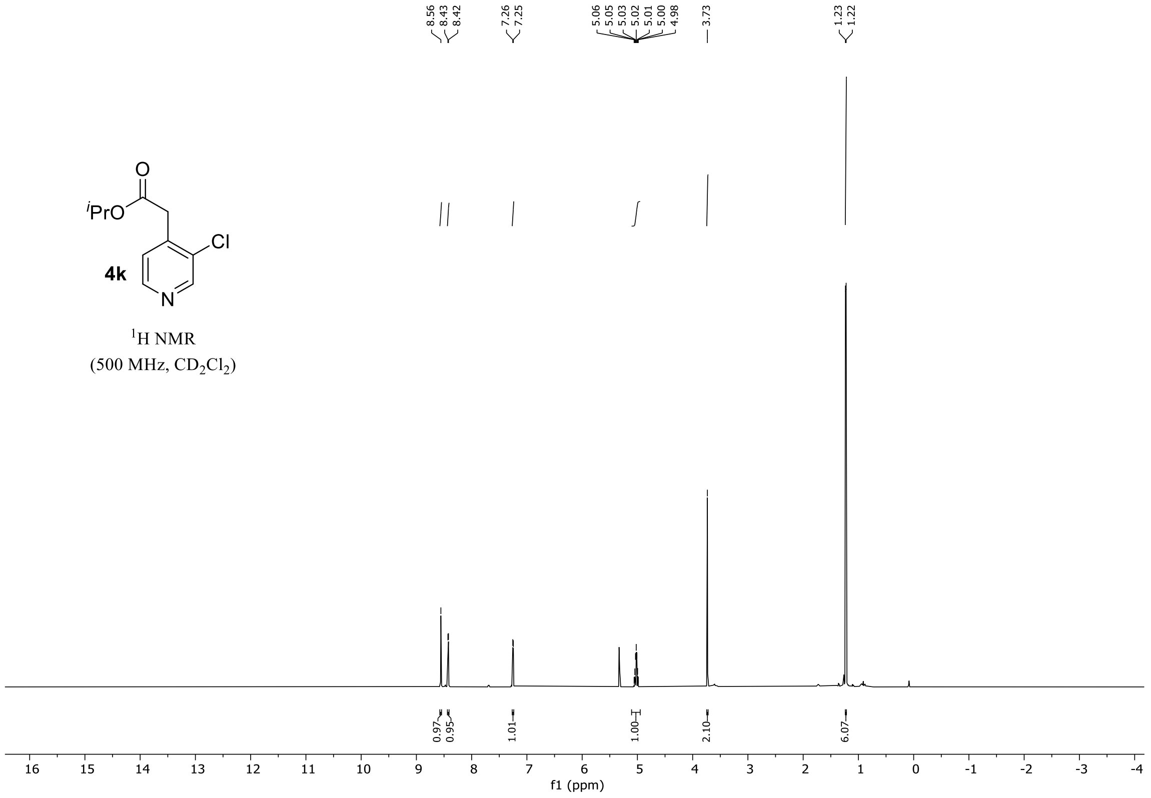
8.56
8.43
8.42
7.26
7.25
5.06
5.05
5.03
5.02
5.01
5.00
4.98
3.73
1.23
1.22
ⁱPrO
O
Cl
4k
N
^{1}H NMR
(500 MHz, CD_2Cl_2)
0.97
0.95
1.01
1.00
2.10
6.07
f1 (ppm)

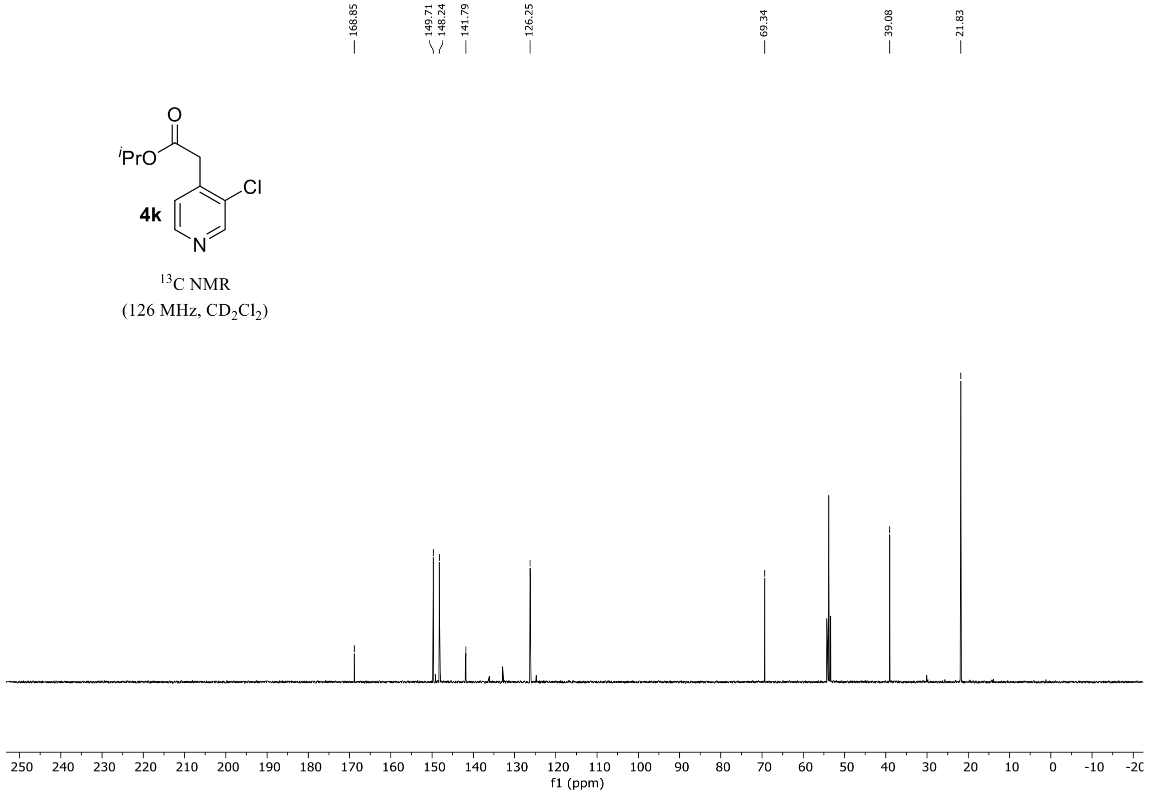

168.85
149.71
148.24
141.79
126.25
69.34
39.08
21.83
O
^{i}PrO
Cl
4k
N
^{13}C NMR
(126 MHz, CD_2Cl_2)
250 240 230 220 210 200 190 180 170 160 150 140 130 120 110 100 90 80 70 60 50 40 30 20 10 0 -10 -20
f1 (ppm)

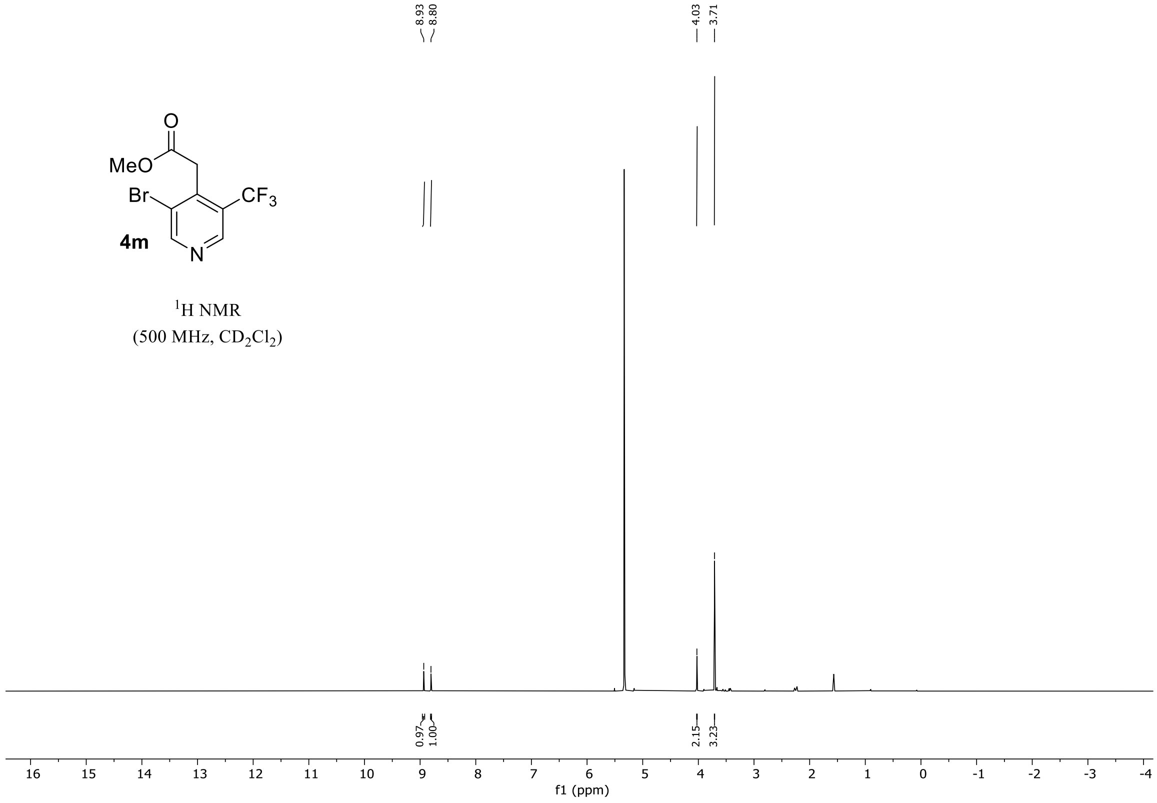

8.93
8.80
4.03
3.71
O
MeO
Br
CF$_3$
4m
N
^{1}H NMR
(500 MHz, CD_2Cl_2)
0.97
1.00
2.15
3.23
16 15 14 13 12 11 10 9 8 7 6 5 4 3 2 1 0 -1 -2 -3 -4
f1 (ppm)

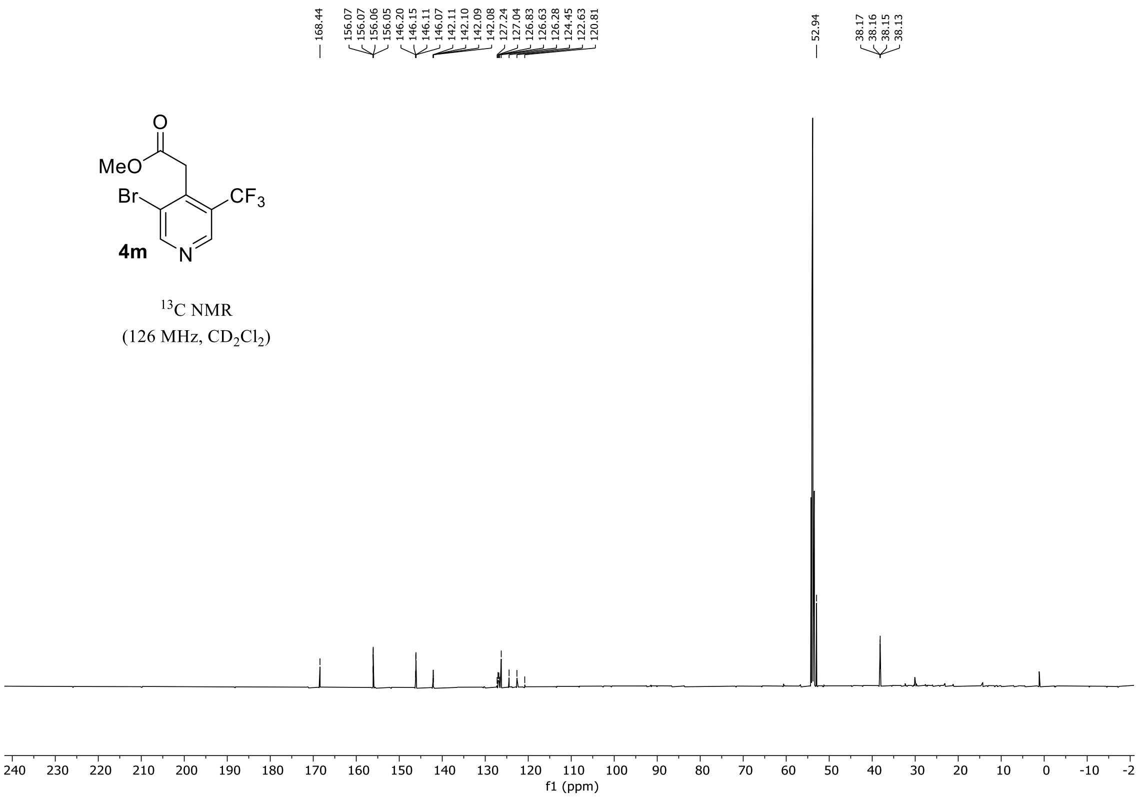

4m

^{13}C NMR
(126 MHz, CD_2Cl_2)

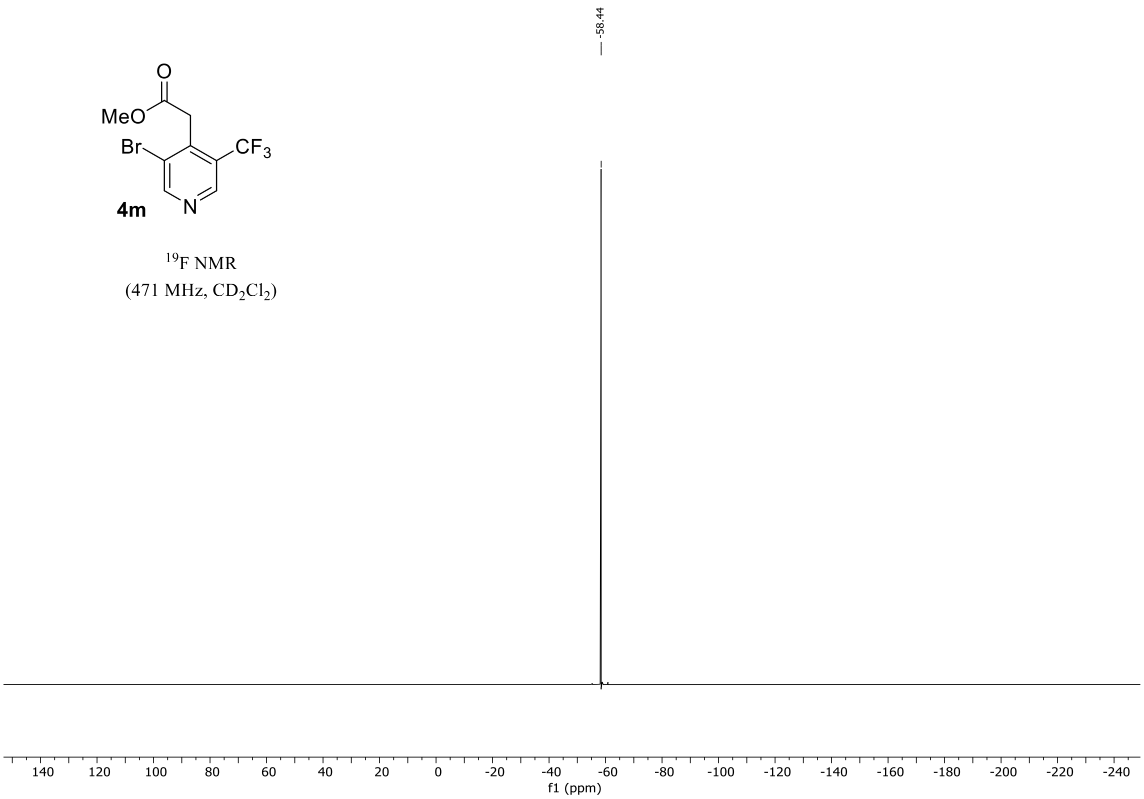

4m

^{19}F NMR
(471 MHz, CD_2Cl_2)

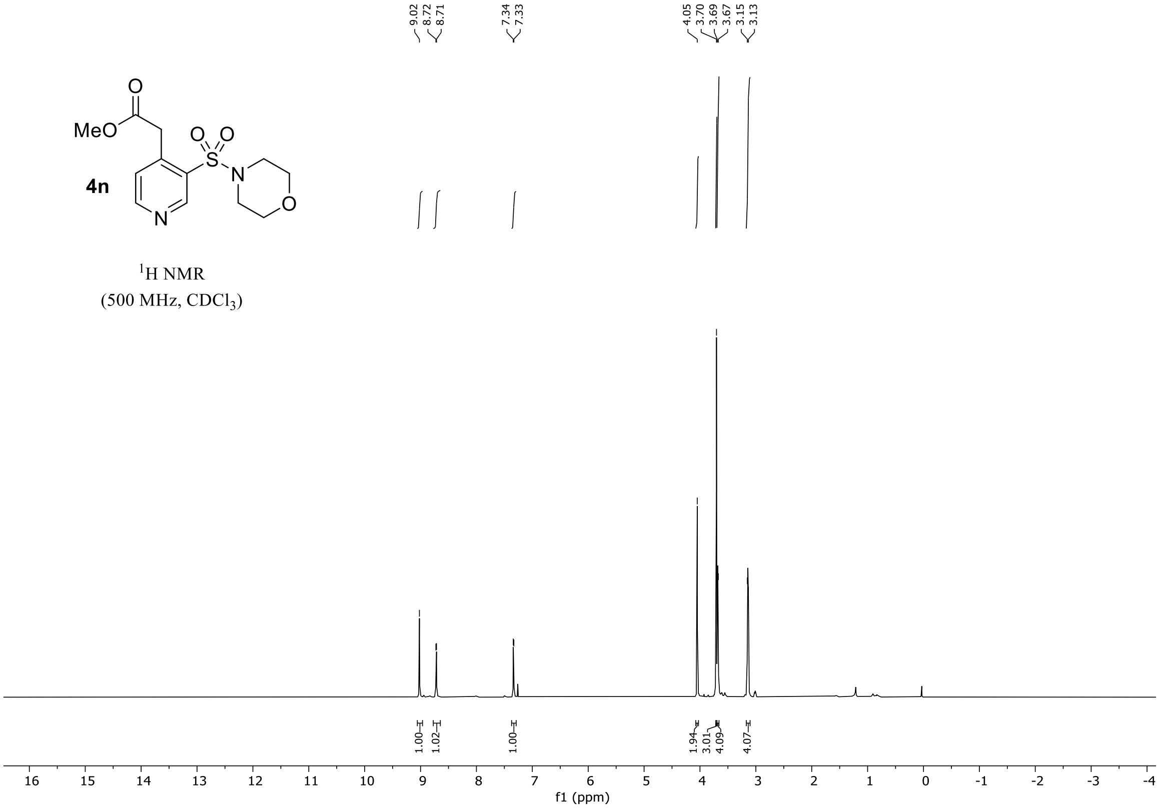

4n
^{1}H NMR
(500 MHz, $CDCl_3$)
9.02
8.72
8.71
7.34
7.33
4.05
3.70
3.69
3.67
3.15
3.13
1.00
1.02
1.00
1.94
3.01
4.09
4.07
f1 (ppm)

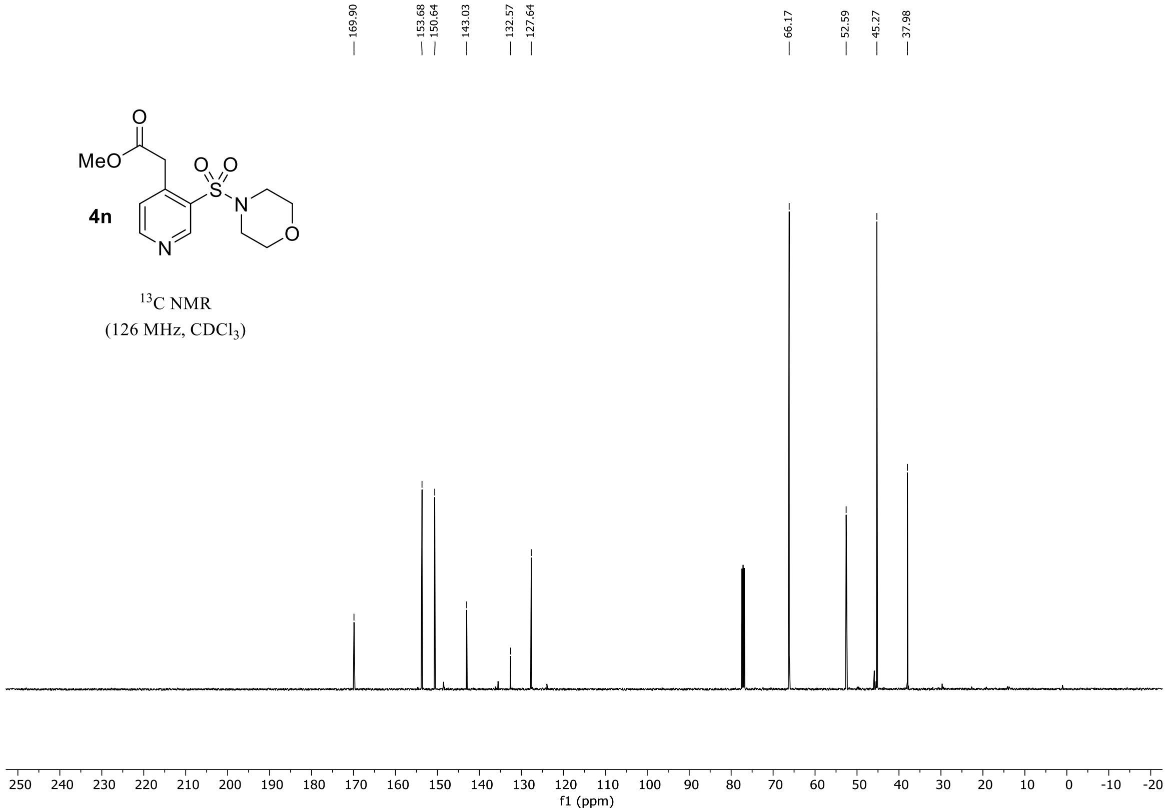

4n
^{13}C NMR
(126 MHz, $CDCl_3$)
169.90
153.68
150.64
143.03
132.57
127.64
66.17
52.59
45.27
37.98
f1 (ppm)

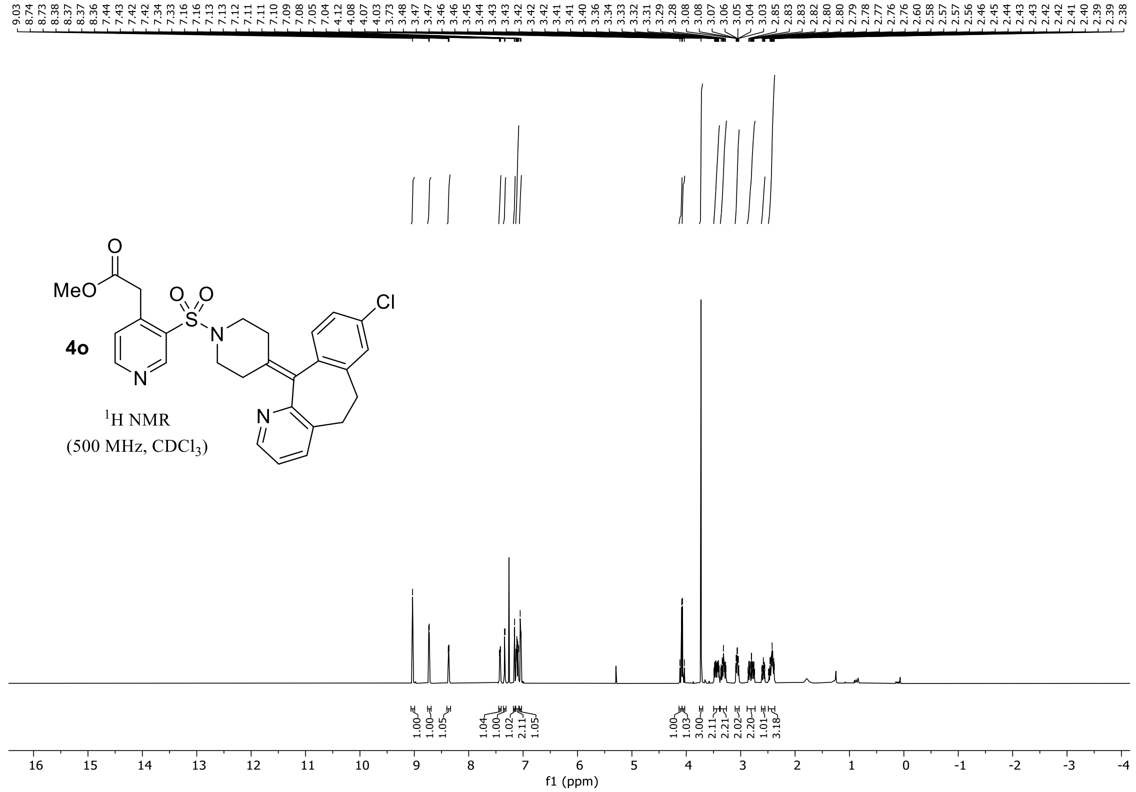
MeO
O
Cl
4o
N
^{1}H NMR
(500 MHz, $CDCl_3$)
f1 (ppm)

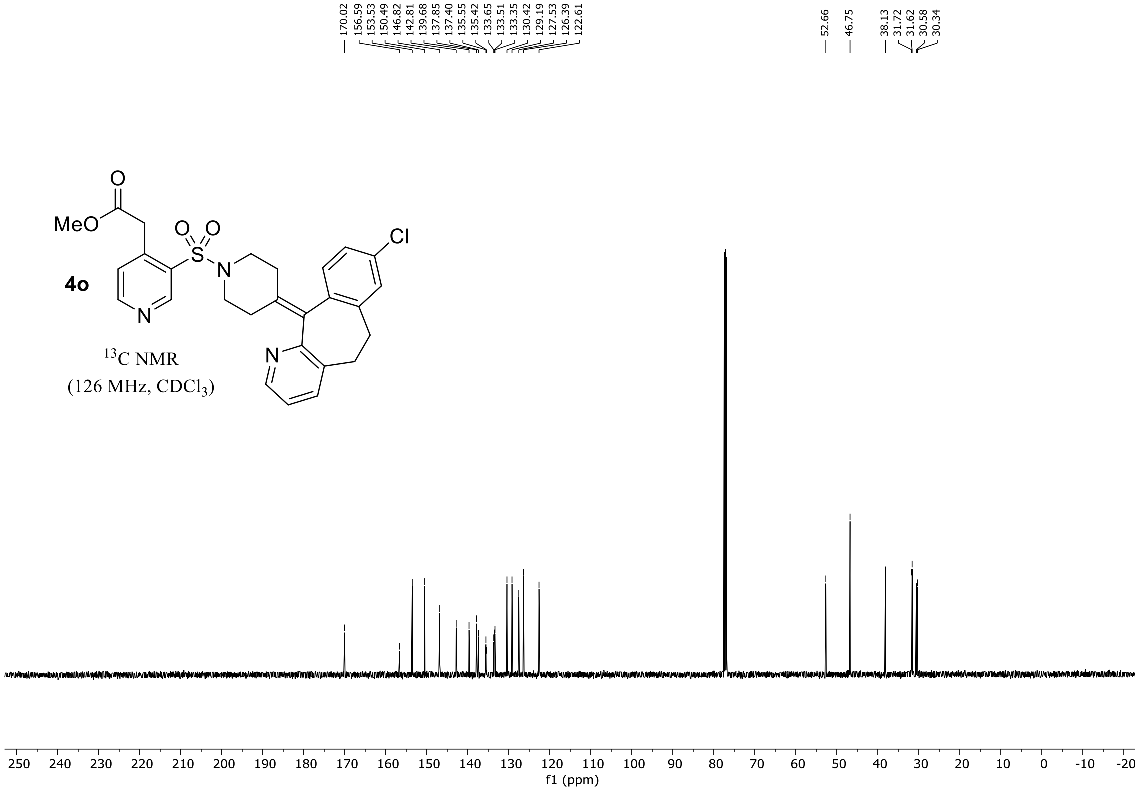
MeO
O
Cl
4o
N
^{13}C NMR
(126 MHz, $CDCl_3$)
f1 (ppm)

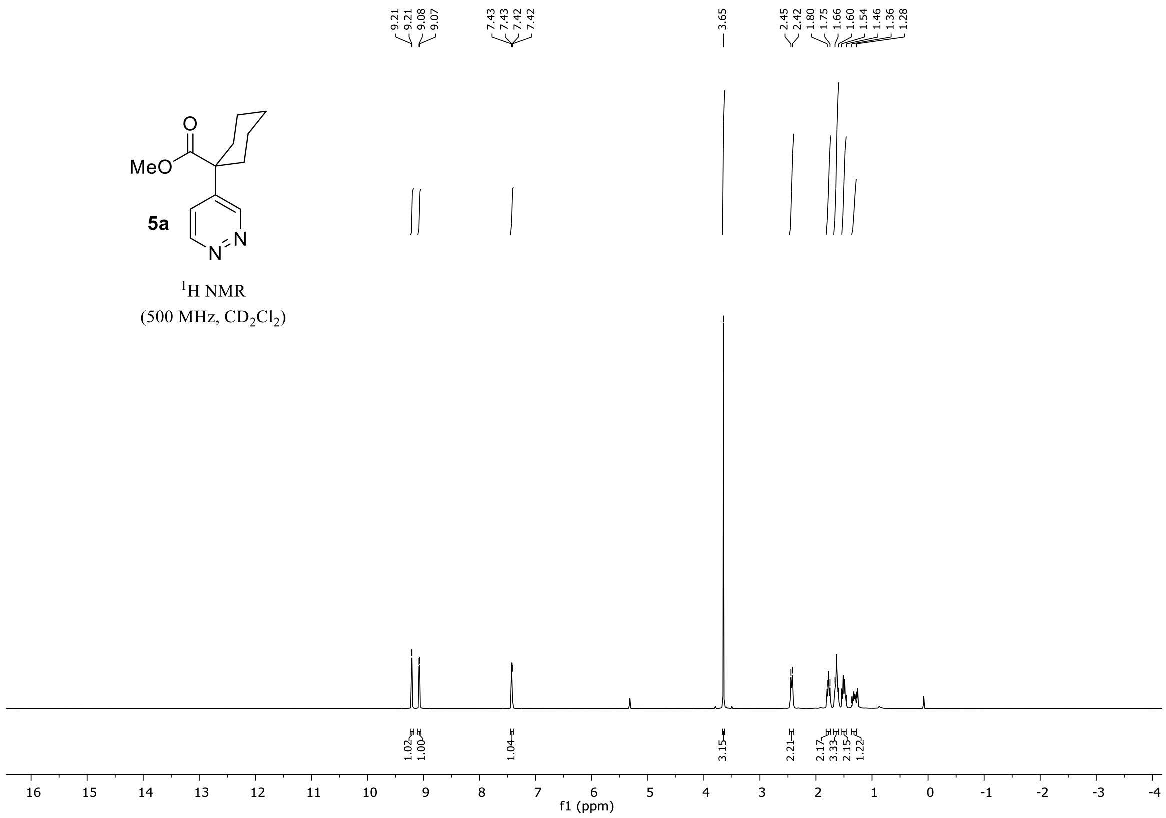
9.21 9.21 9.08 9.07
7.43 7.43 7.42 7.42
3.65
2.45 2.42 1.80 1.75 1.66 1.60 1.54 1.46 1.36 1.28
MeO
O
5a
N
N
1H NMR
(500 MHz, CD2Cl2)
1.02 1.00 1.04 3.15 2.21 2.17 3.33 2.15 1.22
f1 (ppm)

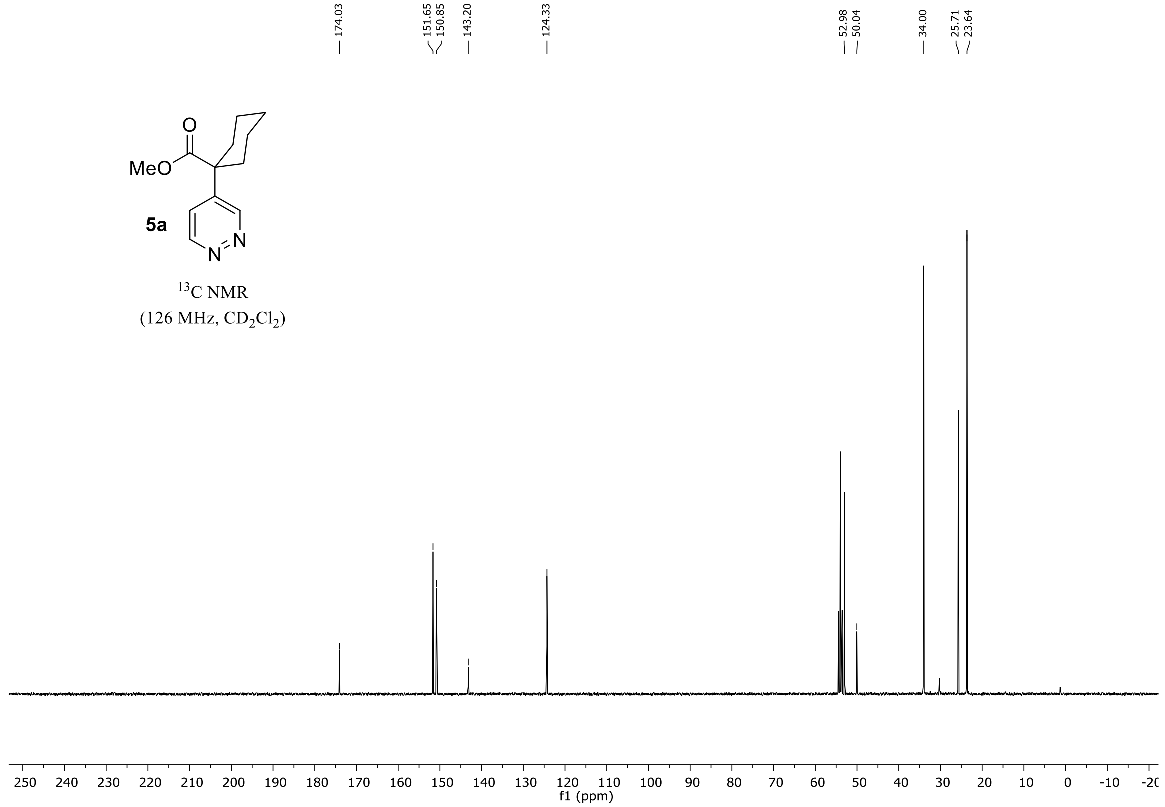
174.03
151.65 150.85
143.20
124.33
52.98 50.04
34.00
25.71 23.64
MeO
O
5a
N
N
13C NMR
(126 MHz, CD2Cl2)
f1 (ppm)

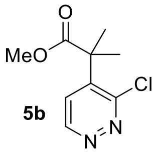

5b

^{1}H NMR
(500 MHz, CD_2Cl_2)

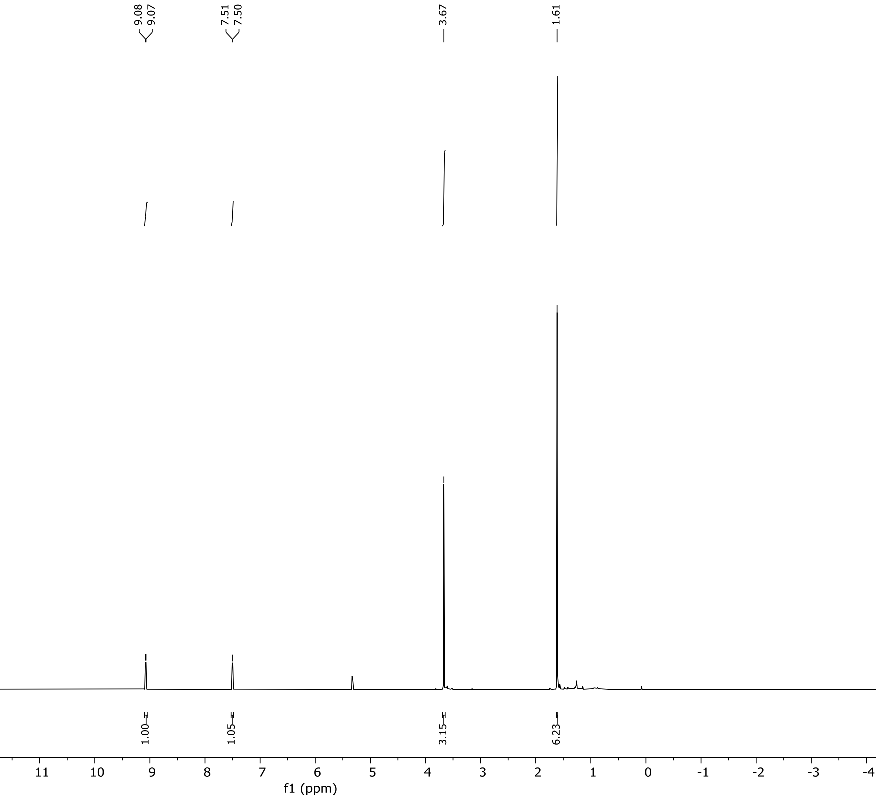

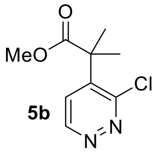

5b

^{13}C NMR
(126 MHz, CD_2Cl_2)

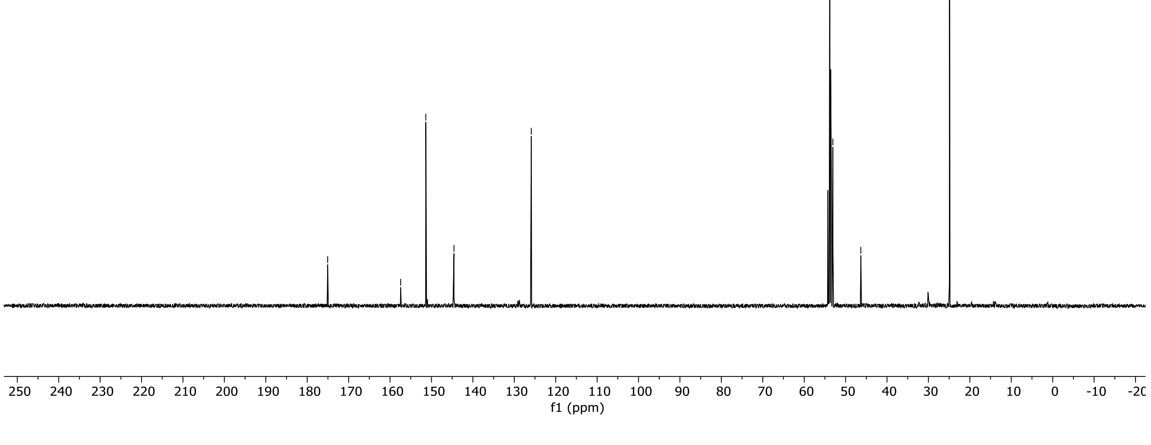

5c

^{1}H NMR
(500 MHz, CD_2Cl_2)

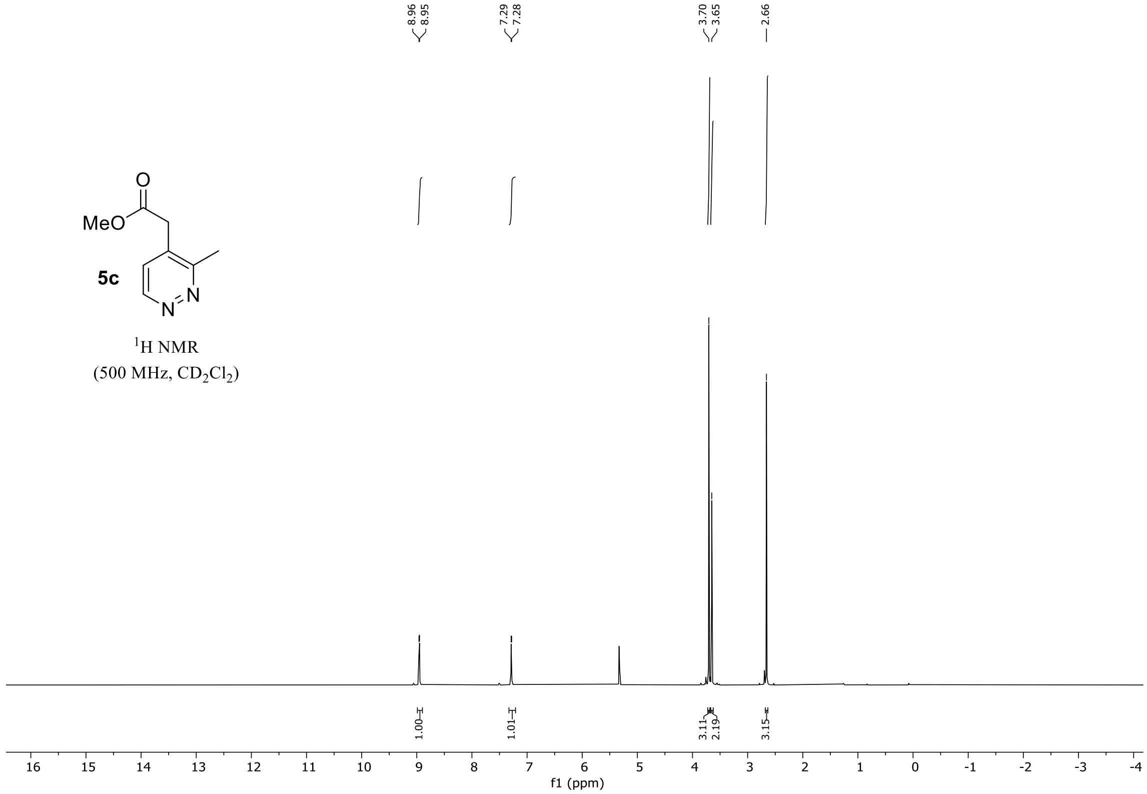

5c

^{13}C NMR
(126 MHz, CD_2Cl_2)

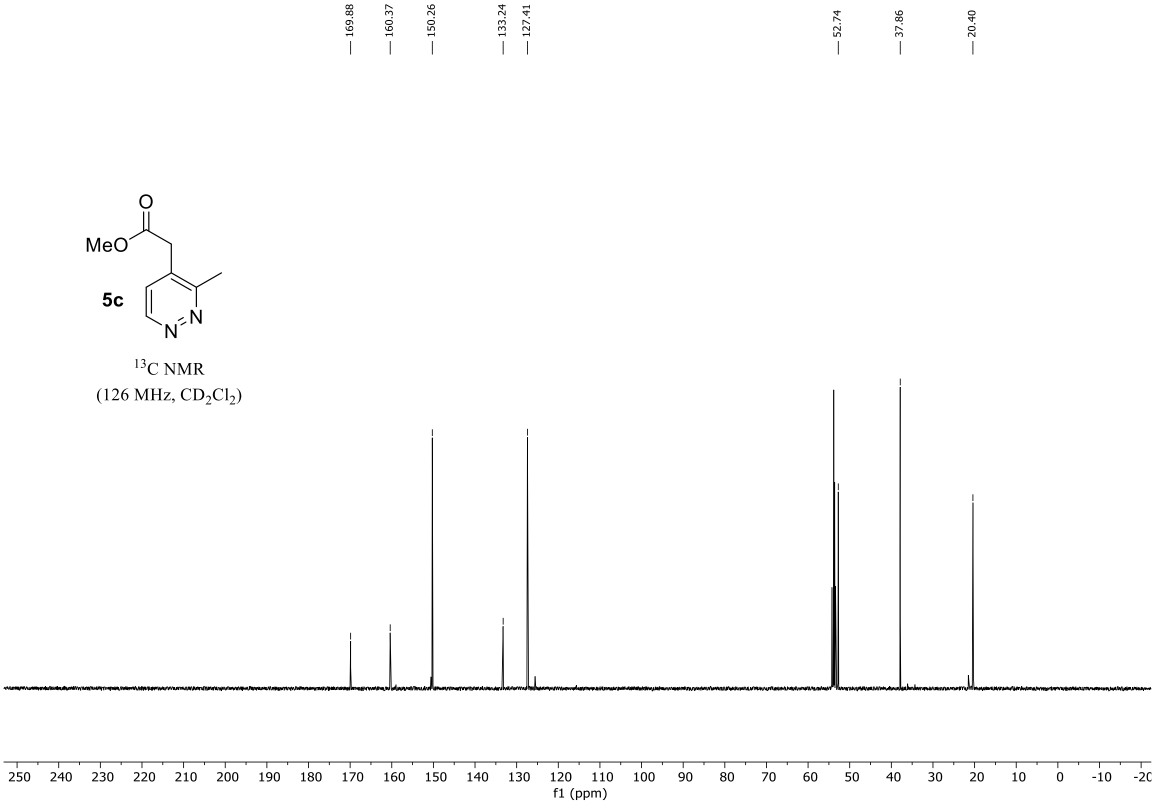

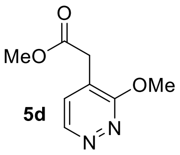

5d

^{1}H NMR
(500 MHz, CD_2Cl_2)

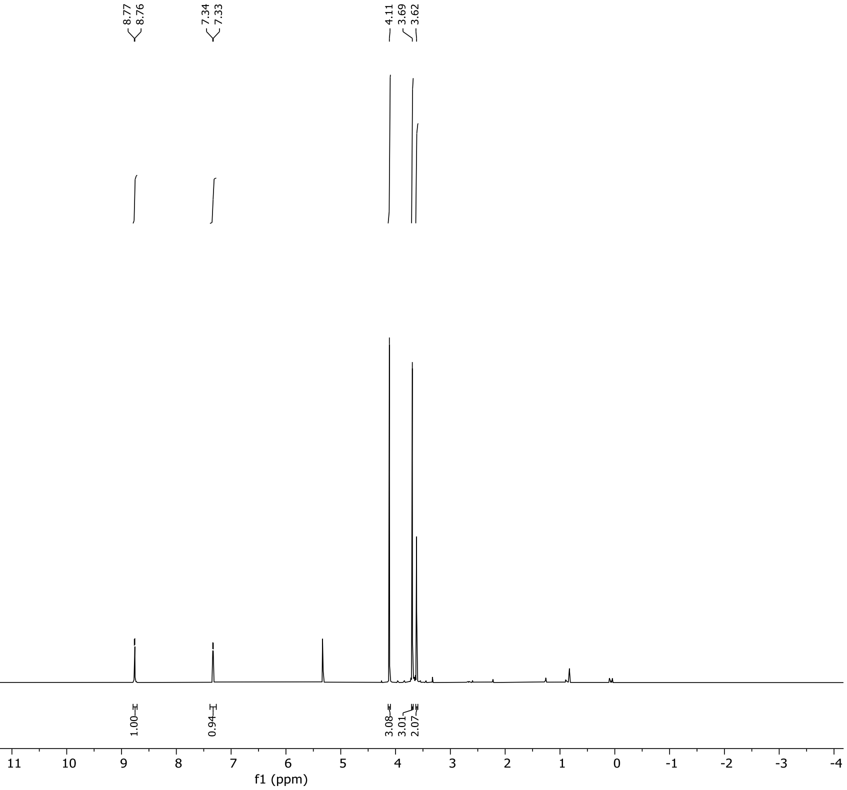

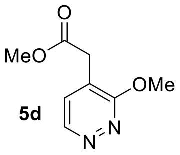

5d

^{13}C NMR
(126 MHz, CD_2Cl_2)

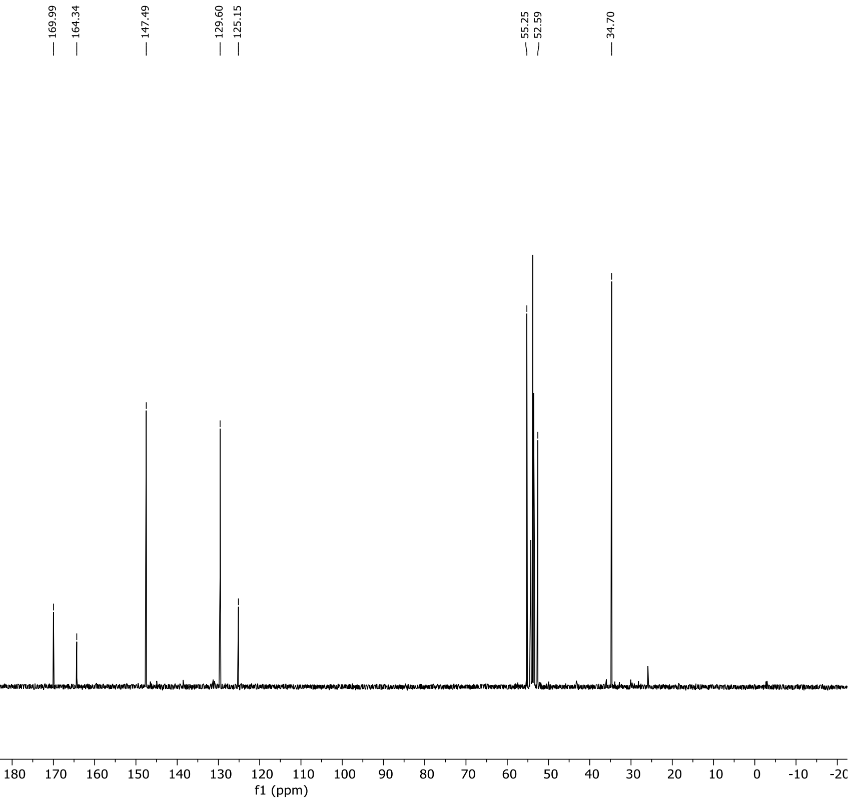

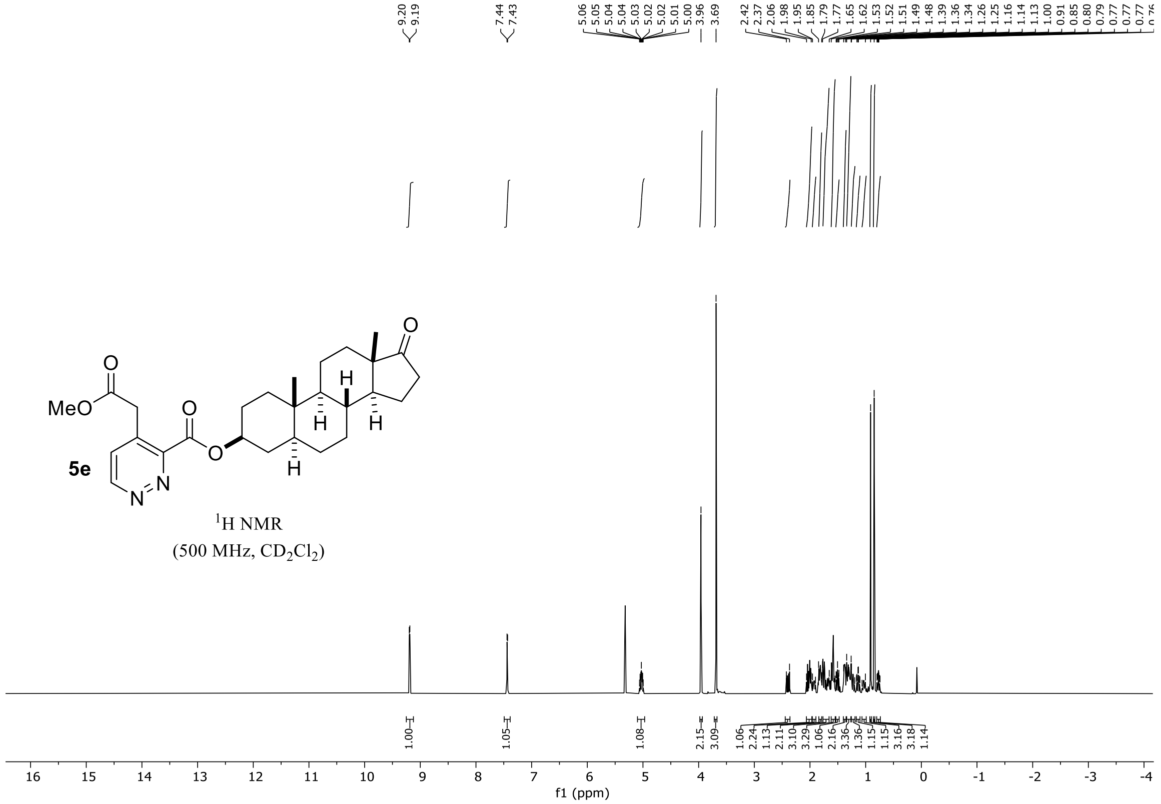

5e
^{1}H NMR
(500 MHz, CD_2Cl_2)
f1 (ppm)

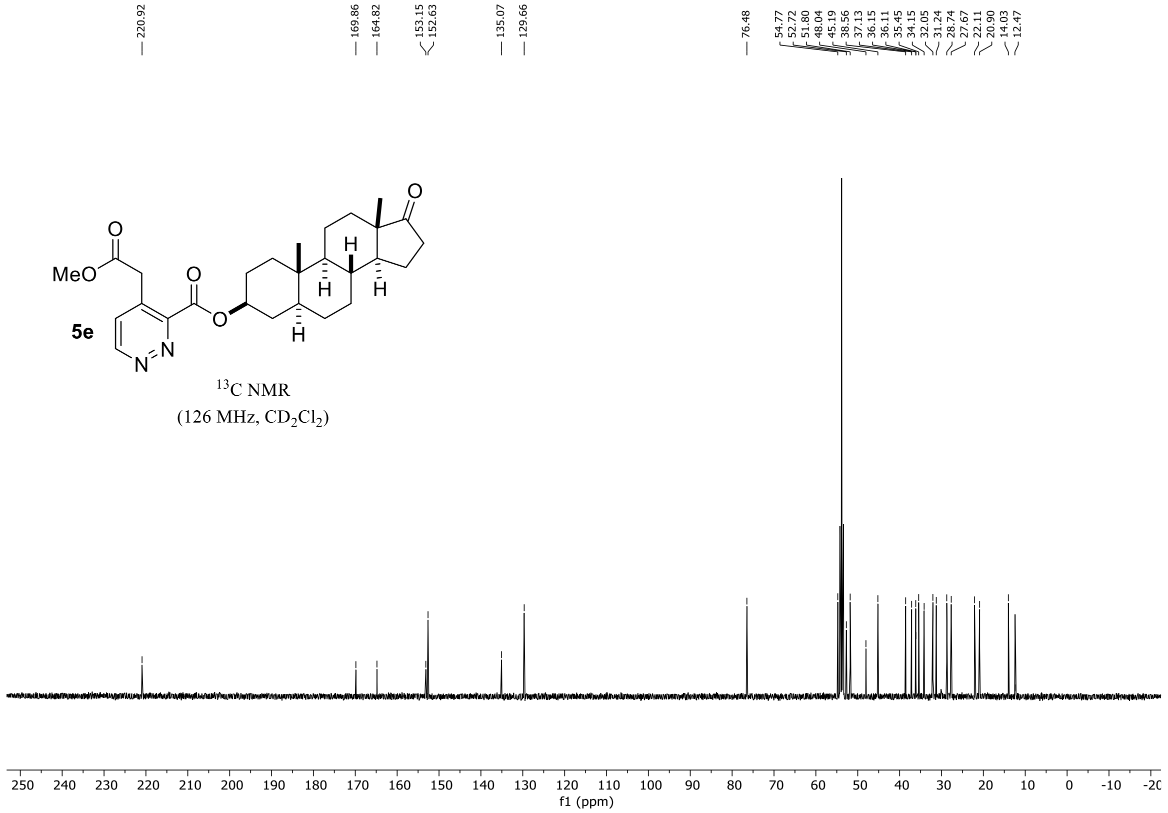

5e
^{13}C NMR
(126 MHz, CD_2Cl_2)
f1 (ppm)

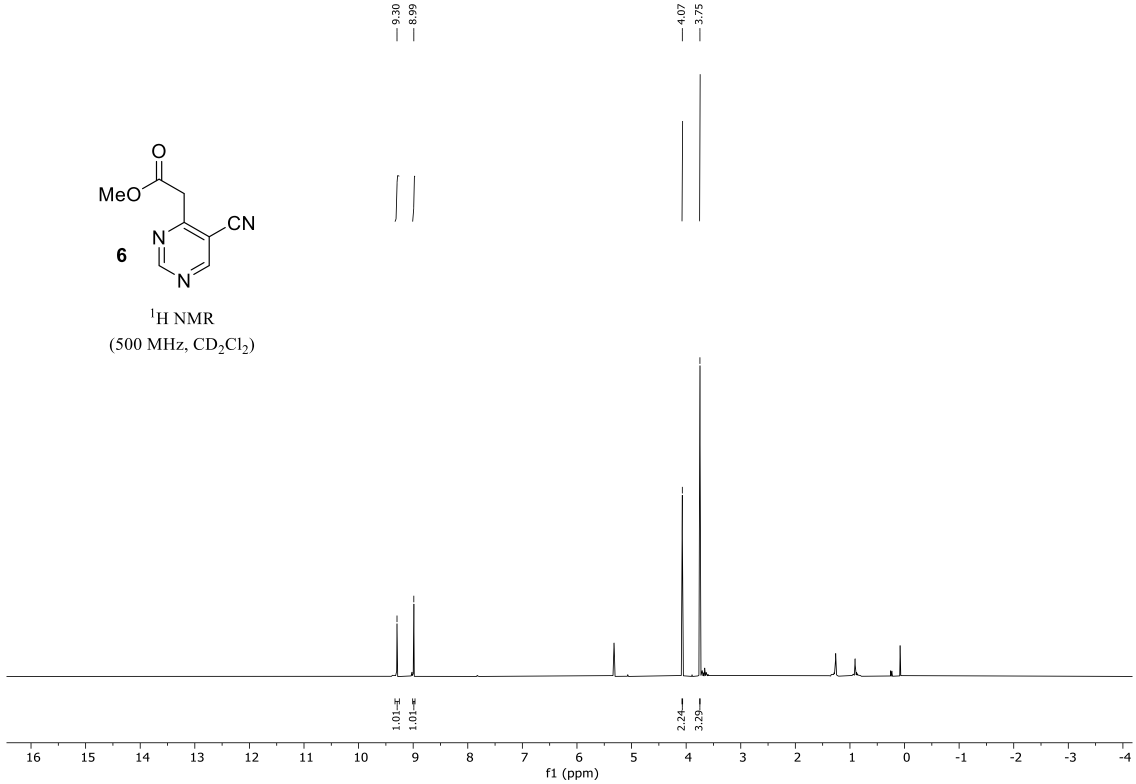
9.30
8.99
4.07
3.75
MeO
O
CN
N
N
6
^{1}H NMR
(500 MHz, CD_2Cl_2)
1.01
1.01
2.24
3.29
f1 (ppm)

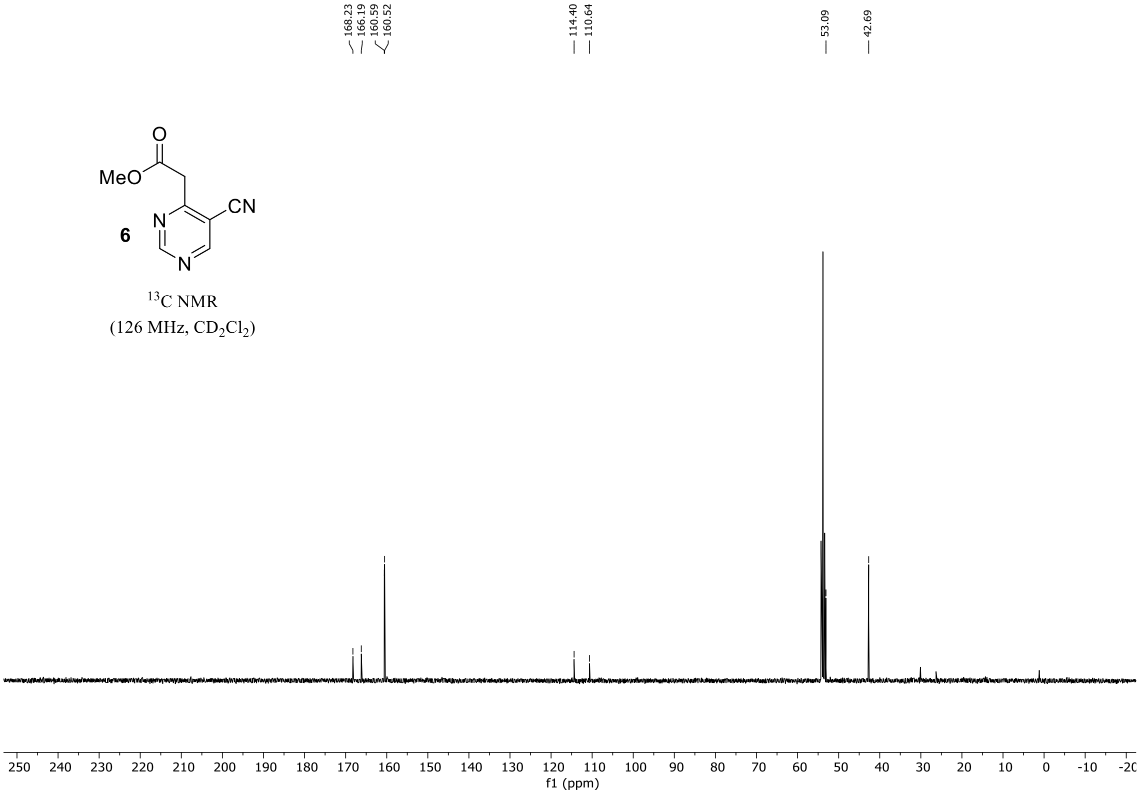
168.23
166.19
160.59
160.52
114.40
110.64
53.09
42.69
MeO
O
CN
N
N
6
^{13}C NMR
(126 MHz, CD_2Cl_2)
f1 (ppm)

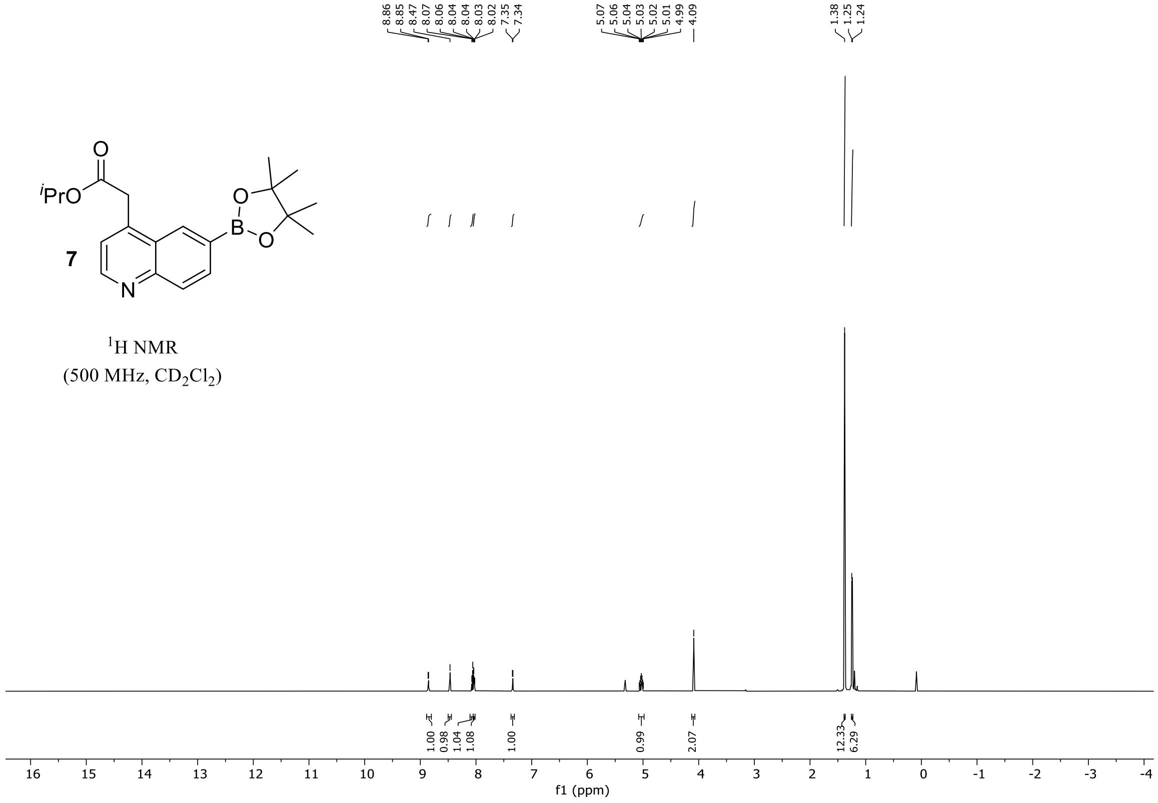

^{1}H NMR
(500 MHz, CD_2Cl_2)

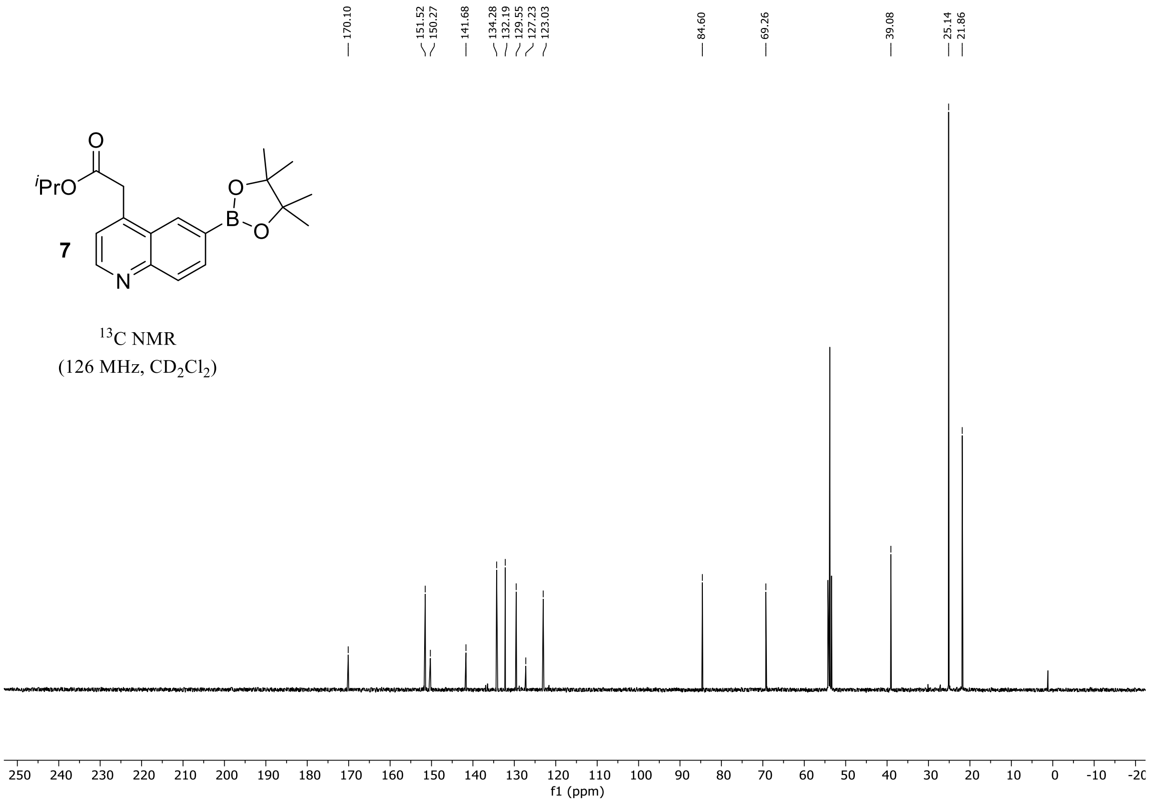

^{13}C NMR
(126 MHz, CD_2Cl_2)

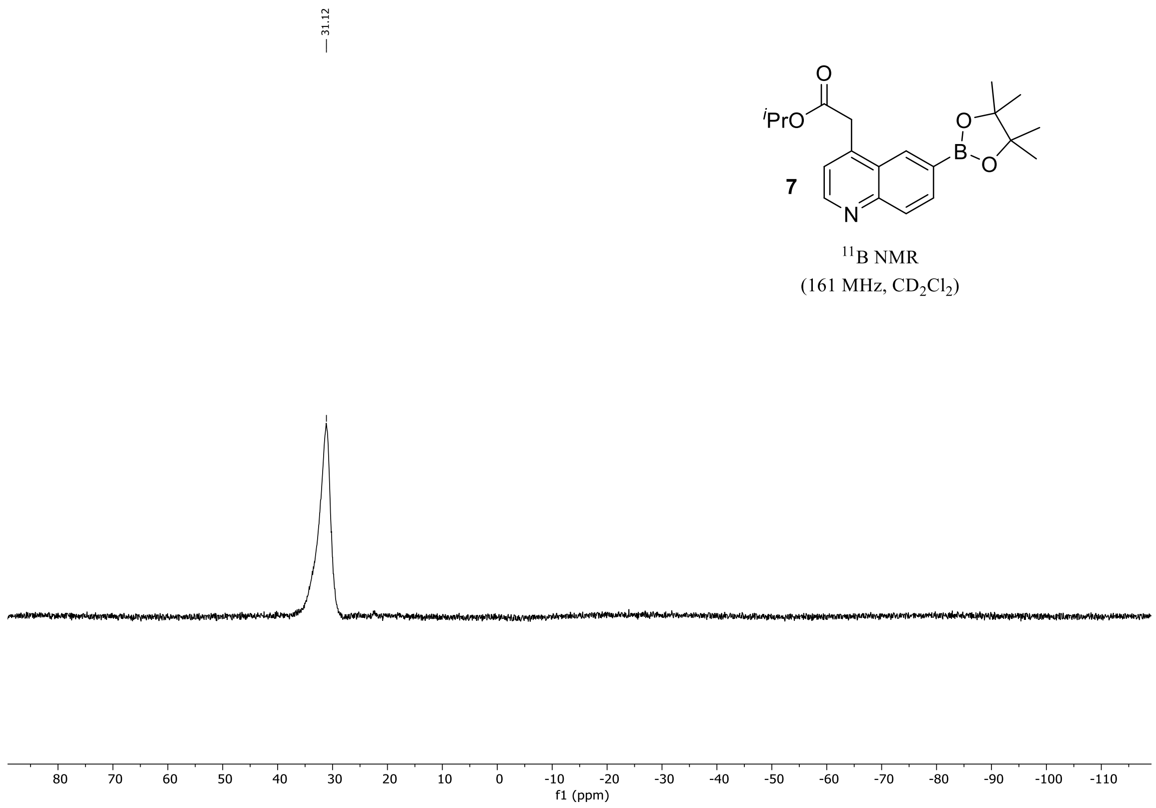
31.12
^{i}PrO
O
7
N
B
O
O
^{11}B NMR
(161 MHz, CD_2Cl_2)
80 70 60 50 40 30 20 10 0 -10 -20 -30 -40 -50 -60 -70 -80 -90 -100 -110
f1 (ppm)

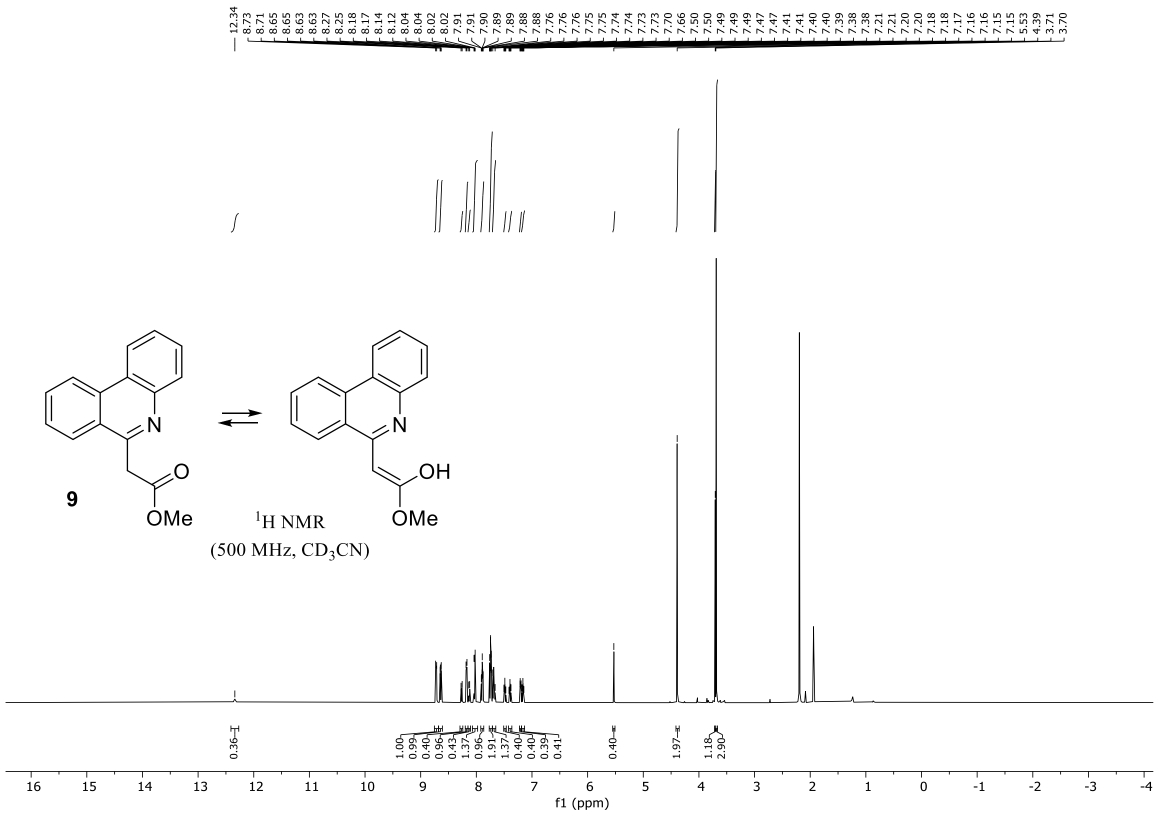
12.34 8.73 8.71 8.65 8.65 8.63 8.63 8.27 8.25 8.18 8.17 8.14 8.12 8.04 8.04 8.02 8.02 7.91 7.91 7.90 7.89 7.89 7.88 7.88 7.76 7.76 7.76 7.75 7.75 7.74 7.74 7.73 7.73 7.70 7.66 7.50 7.50 7.49 7.49 7.49 7.47 7.47 7.41 7.41 7.40 7.40 7.39 7.38 7.38 7.21 7.21 7.20 7.20 7.18 7.18 7.17 7.16 7.16 7.15 7.15 5.53 4.39 3.71 3.70
N
O
9
OMe
N
OH
OMe
^{1}H NMR
(500 MHz, CD_3CN)
0.36 1.00 0.99 0.40 0.96 0.43 1.37 0.96 1.91 1.37 0.40 0.39 0.41 0.40 1.97 1.18 2.90
16 15 14 13 12 11 10 9 8 7 6 5 4 3 2 1 0 -1 -2 -3 -4
f1 (ppm)

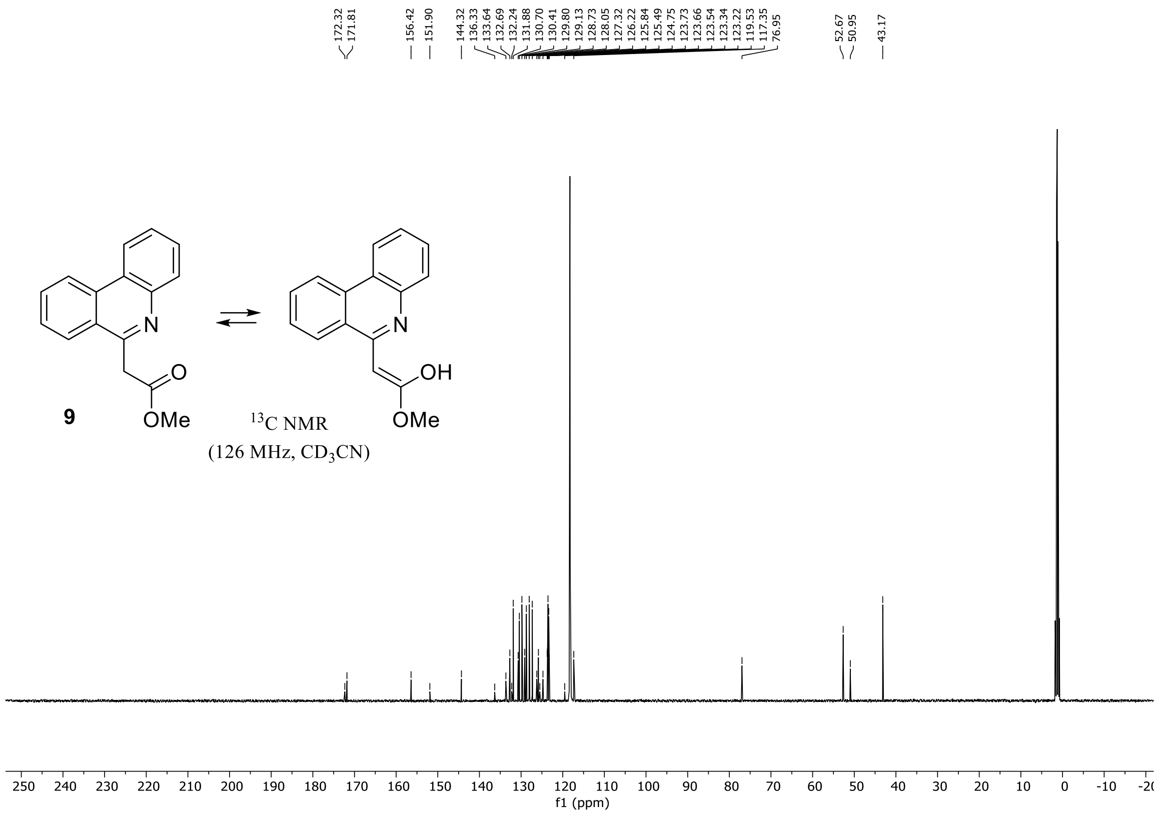
172.32
171.81
156.42
151.90
144.32
136.33
133.64
132.69
132.24
131.88
130.70
130.41
129.80
129.13
128.73
128.05
127.32
126.22
125.84
125.49
124.75
123.73
123.66
123.54
123.34
123.22
119.53
117.35
76.95
52.67
50.95
43.17
N
O
OMe
9
N
OH
OMe
^{13}C NMR
(126 MHz, CD_3CN)
250 240 230 220 210 200 190 180 170 160 150 140 130 120 110 100 90 80 70 60 50 40 30 20 10 0 -10 -20
f1 (ppm)

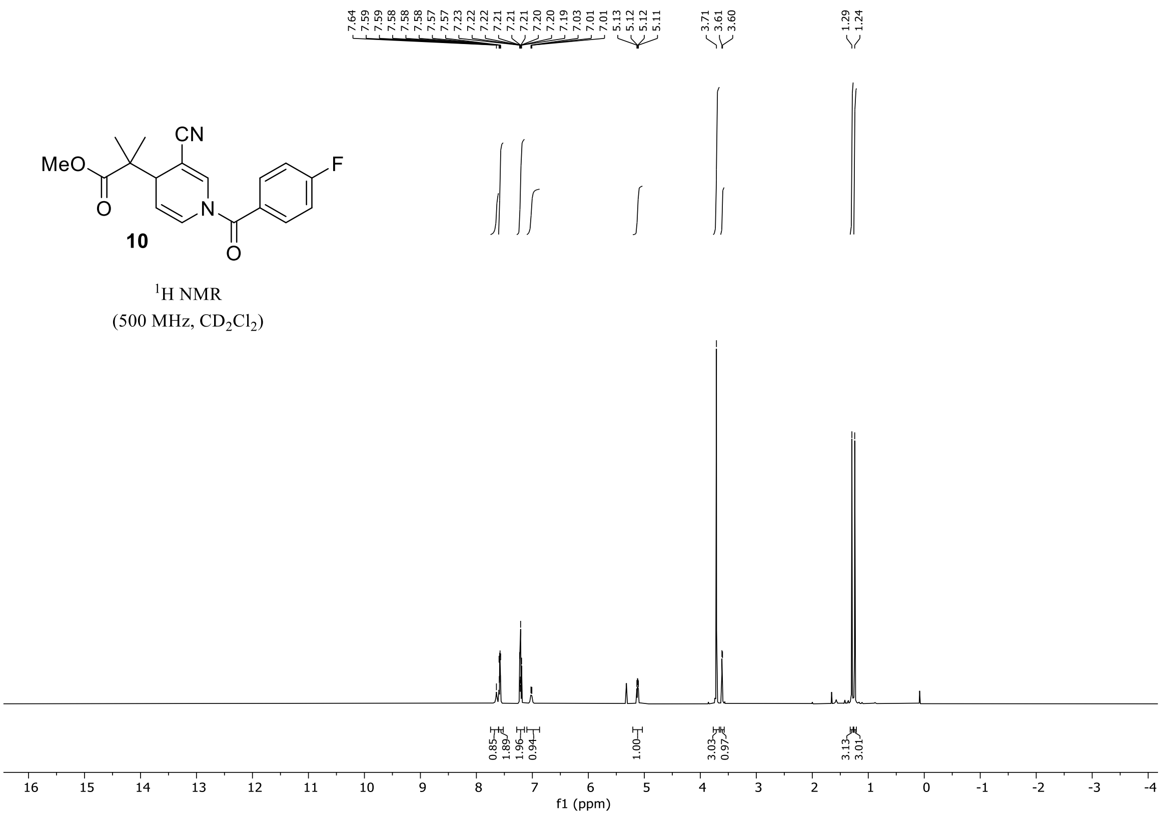
7.64
7.59
7.59
7.58
7.58
7.58
7.57
7.23
7.22
7.22
7.21
7.21
7.21
7.20
7.19
7.03
7.01
7.01
5.13
5.12
5.12
5.11
3.71
3.61
3.60
1.29
1.24
MeO
CN
F
O
N
O
10
^{1}H NMR
(500 MHz, CD_2Cl_2)
0.85
1.89
1.96
0.94
1.00
3.03
0.97
3.13
3.01
16 15 14 13 12 11 10 9 8 7 6 5 4 3 2 1 0 -1 -2 -3 -4
f1 (ppm)

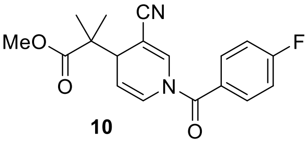

10

^{13}C NMR
(126 MHz, CD_2Cl_2)

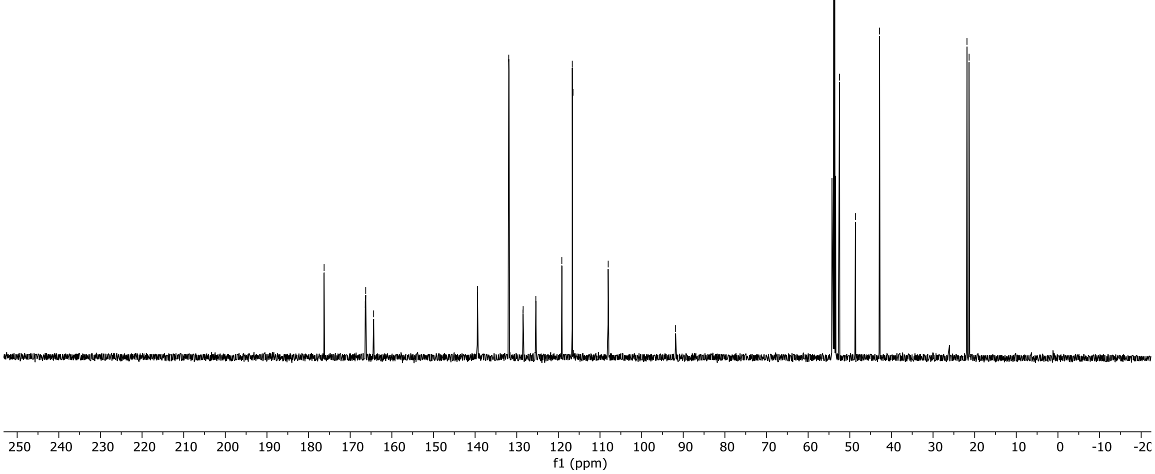

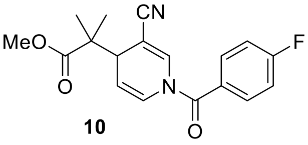

10

^{19}F NMR
(471 MHz, CD_2Cl_2)

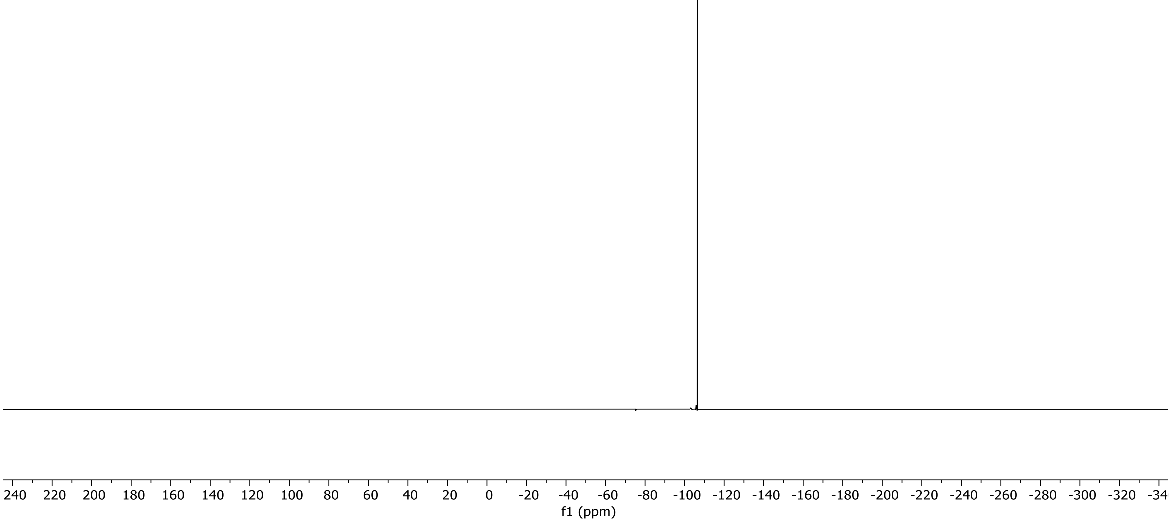

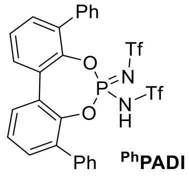

^{1}H NMR
(500 MHz, CD_3OD)

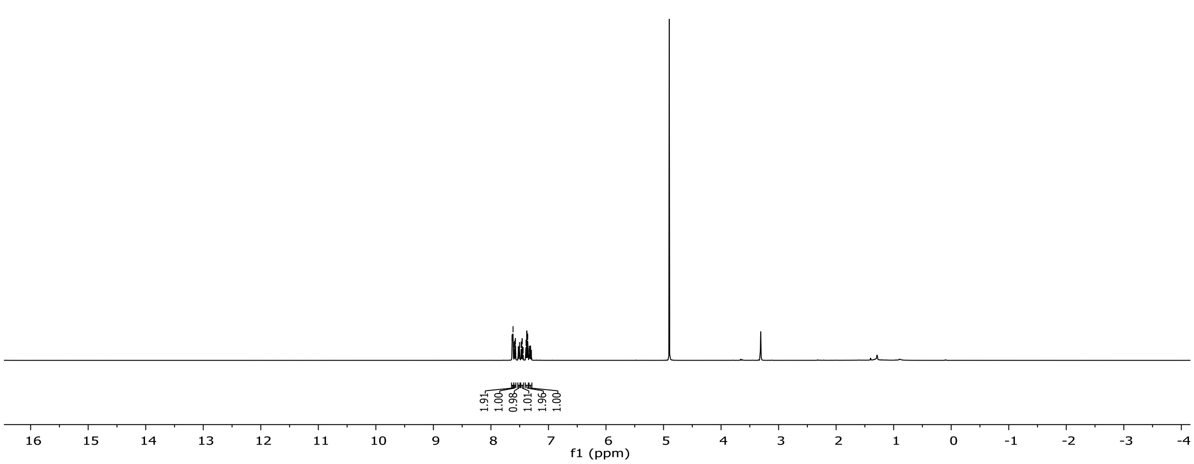

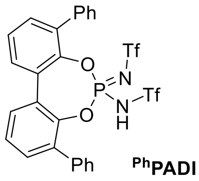

^{13}C NMR
(126 MHz, CD_3OD)

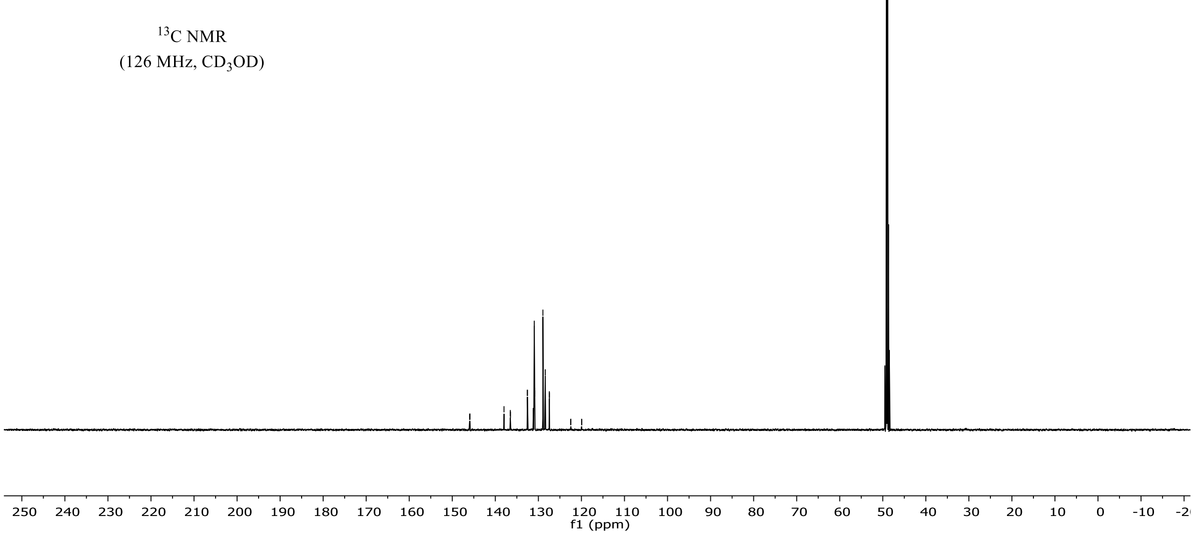

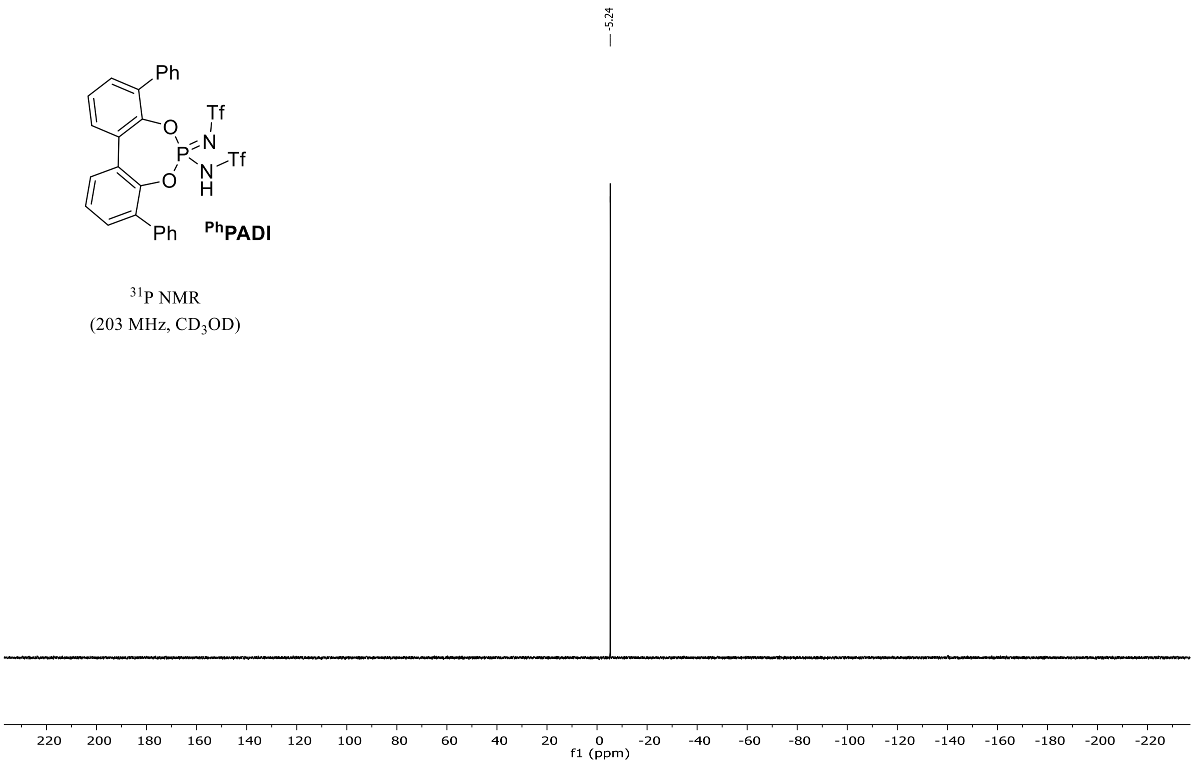
-5.24
Ph
Tf
O
P
N
N
H
Tf
O
Ph
PhPADI
31P NMR
(203 MHz, CD3OD)
220 200 180 160 140 120 100 80 60 40 20 0 -20 -40 -60 -80 -100 -120 -140 -160 -180 -200 -220
f1 (ppm)

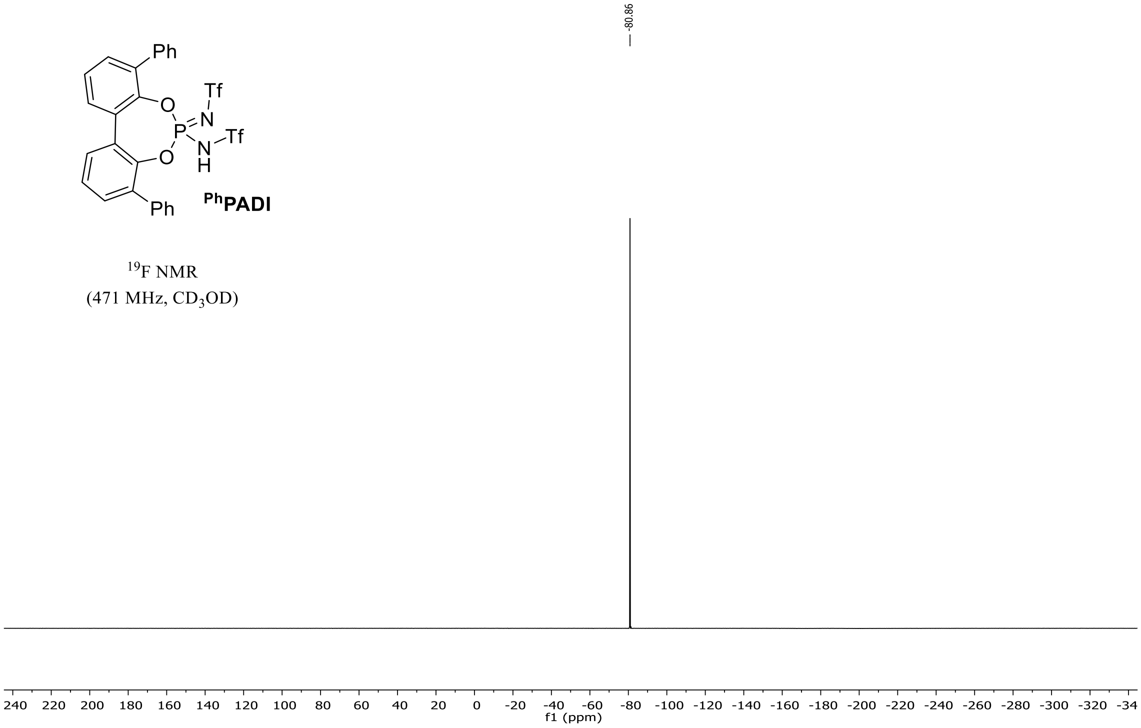
-80.86
Ph
Tf
O
P
N
N
H
Tf
O
Ph
PhPADI
19F NMR
(471 MHz, CD3OD)
240 220 200 180 160 140 120 100 80 60 40 20 0 -20 -40 -60 -80 -100 -120 -140 -160 -180 -200 -220 -240 -260 -280 -300 -320 -34
f1 (ppm)

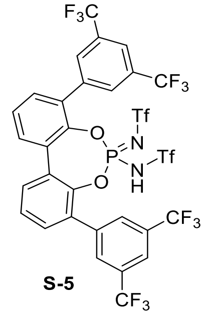

S-5

^{1}H NMR
(500 MHz, CD_3OD)

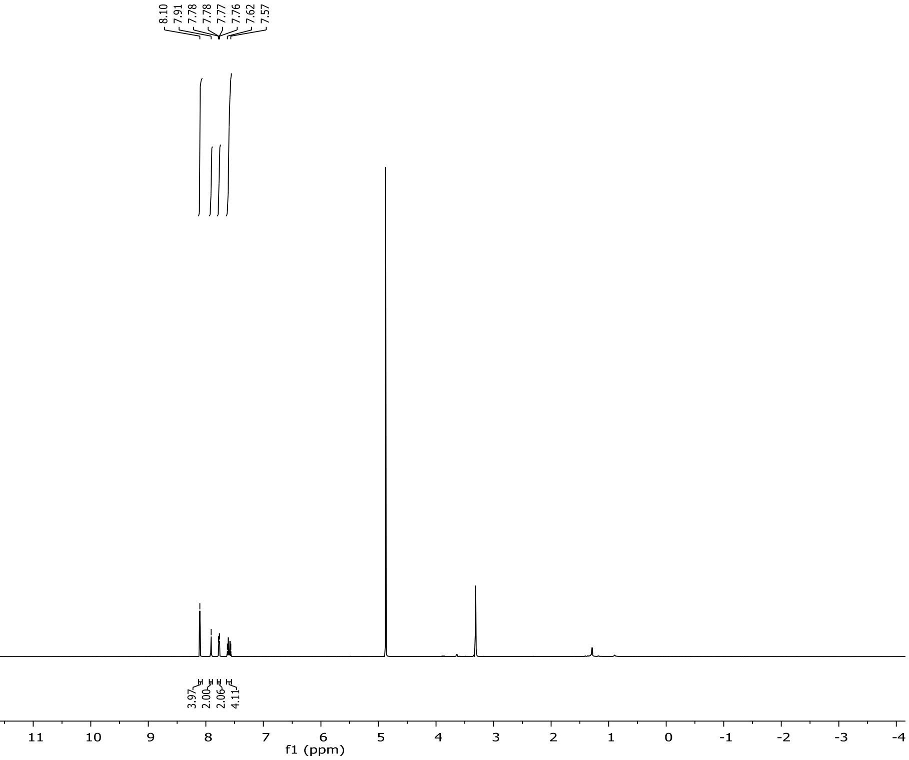

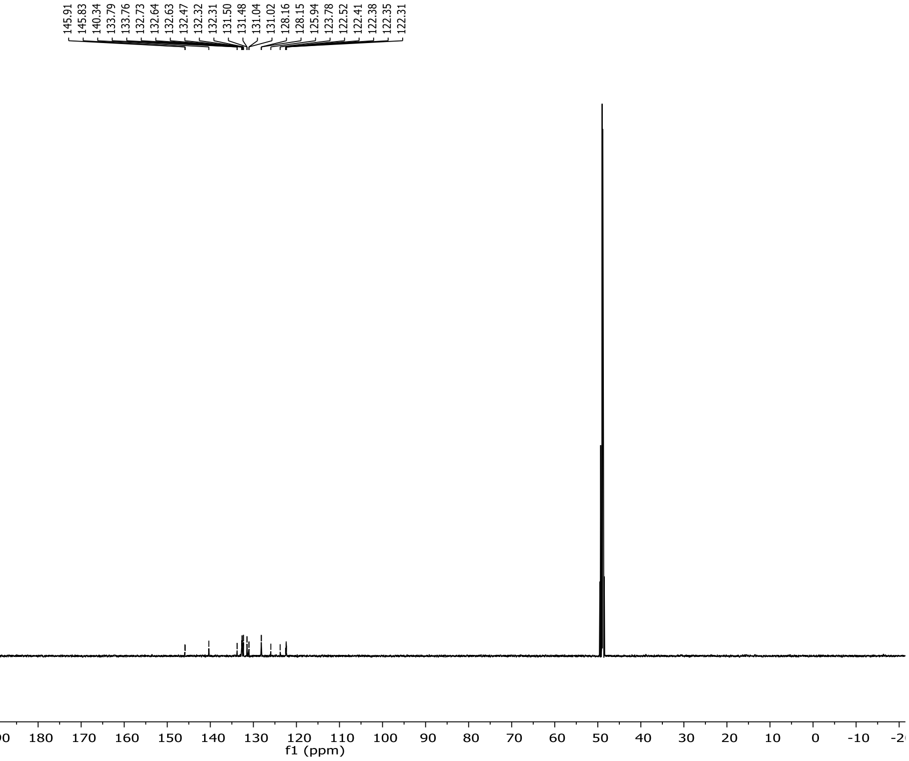

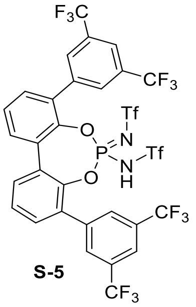

S-5

^{13}C NMR
(126 MHz, CD_3OD)

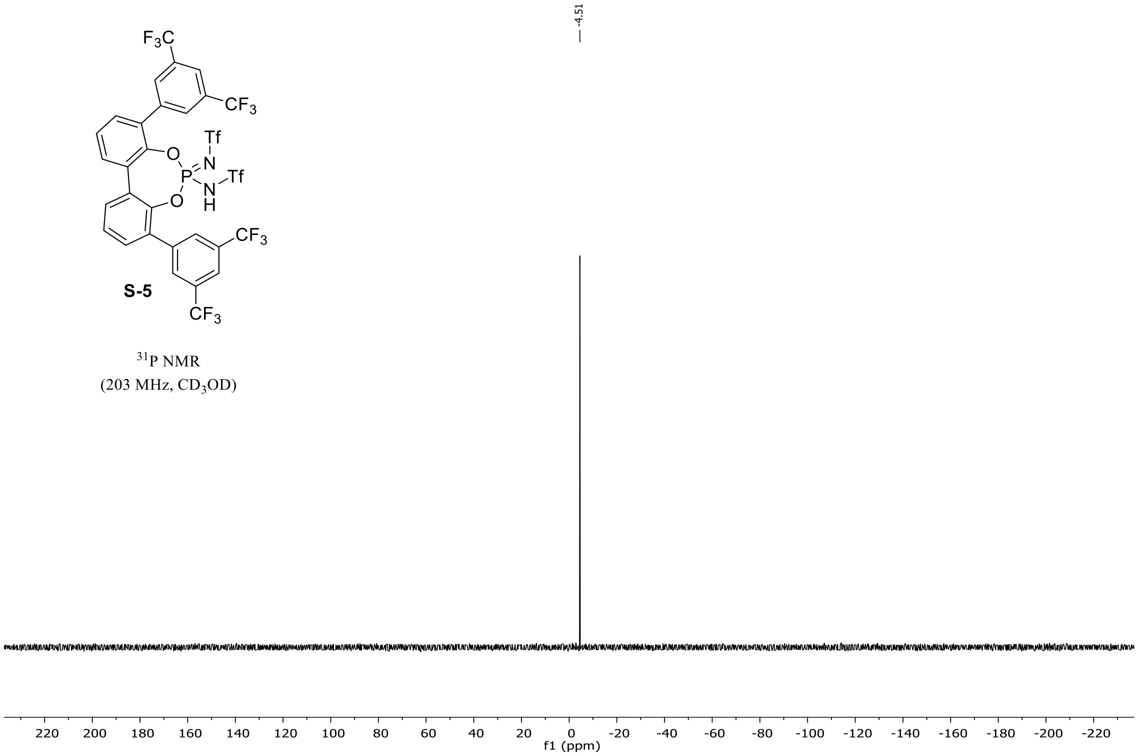

S-5

^{31}P NMR
(203 MHz, CD_3OD)

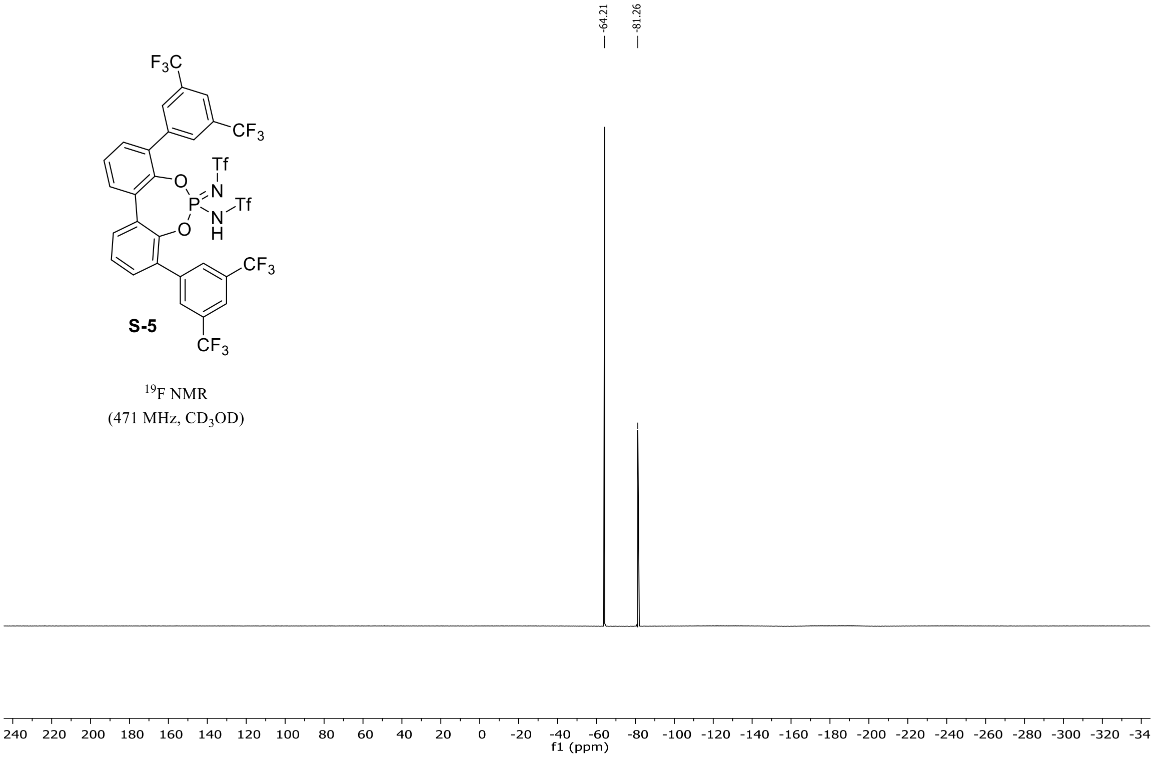

S-5

^{19}F NMR
(471 MHz, CD_3OD)

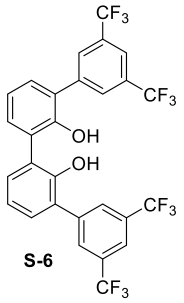

S-6

^{1}H NMR
(500 MHz, $CDCl_3$)

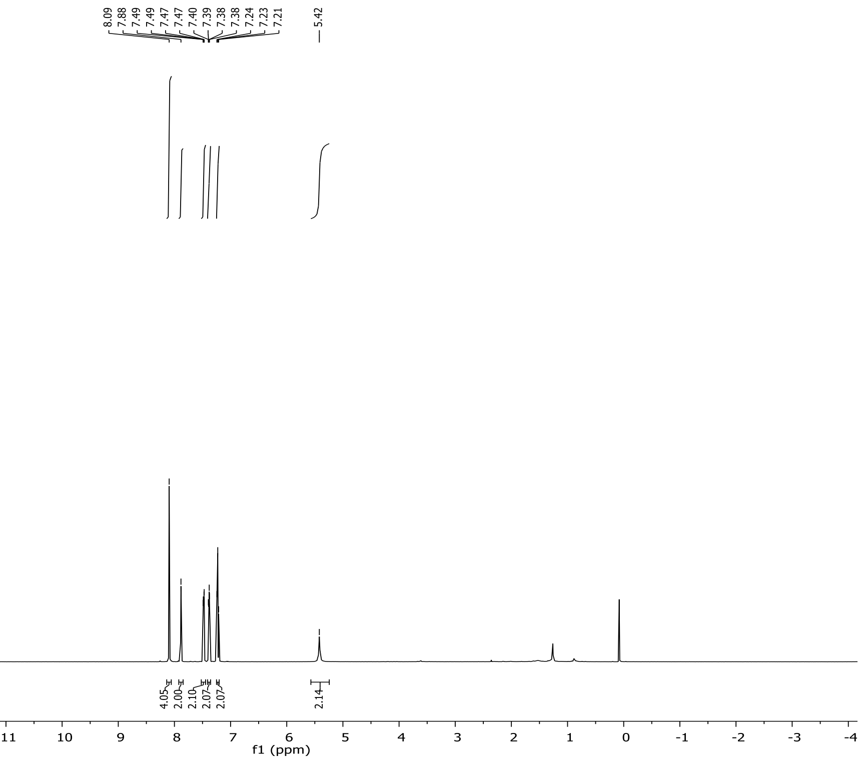

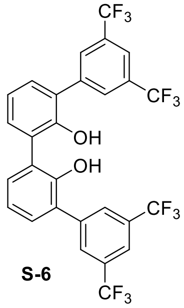

S-6

^{13}C NMR
(126 MHz, $CDCl_3$)

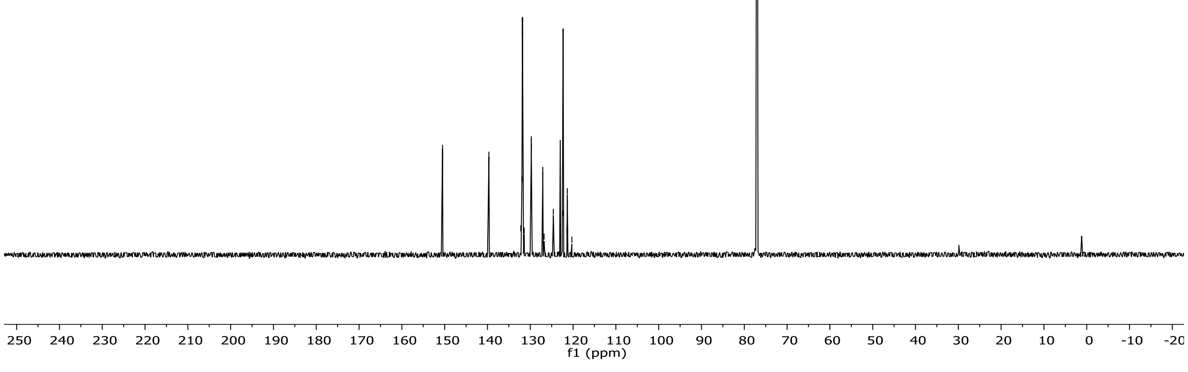

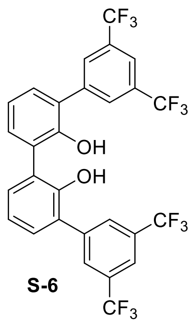

S-6

^{19}F NMR
(471 MHz, CD_2Cl_2)

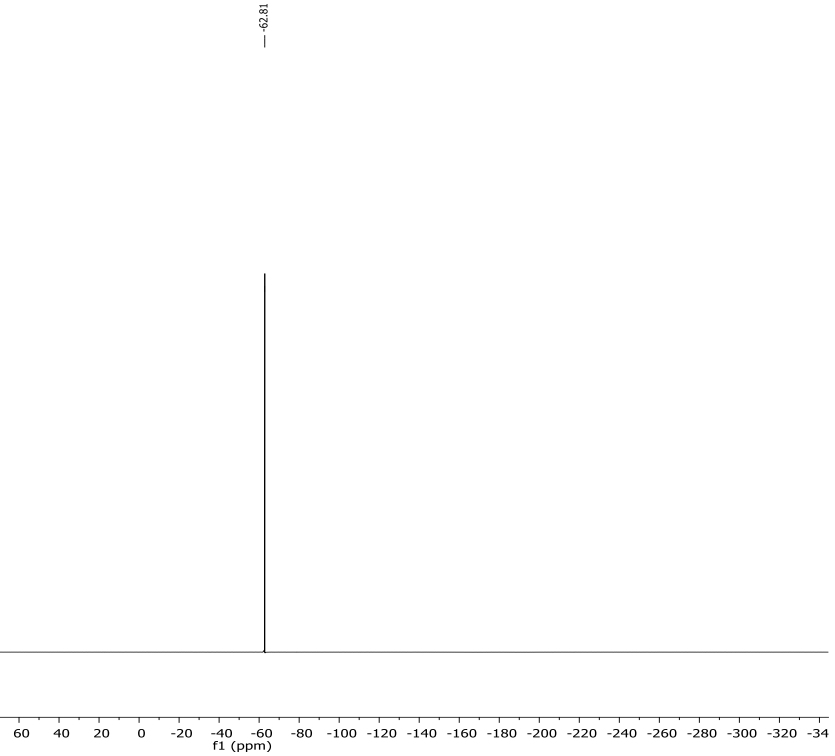

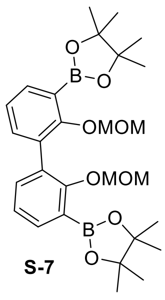

S-7

^{1}H NMR
(500 MHz, CD_2Cl_2)

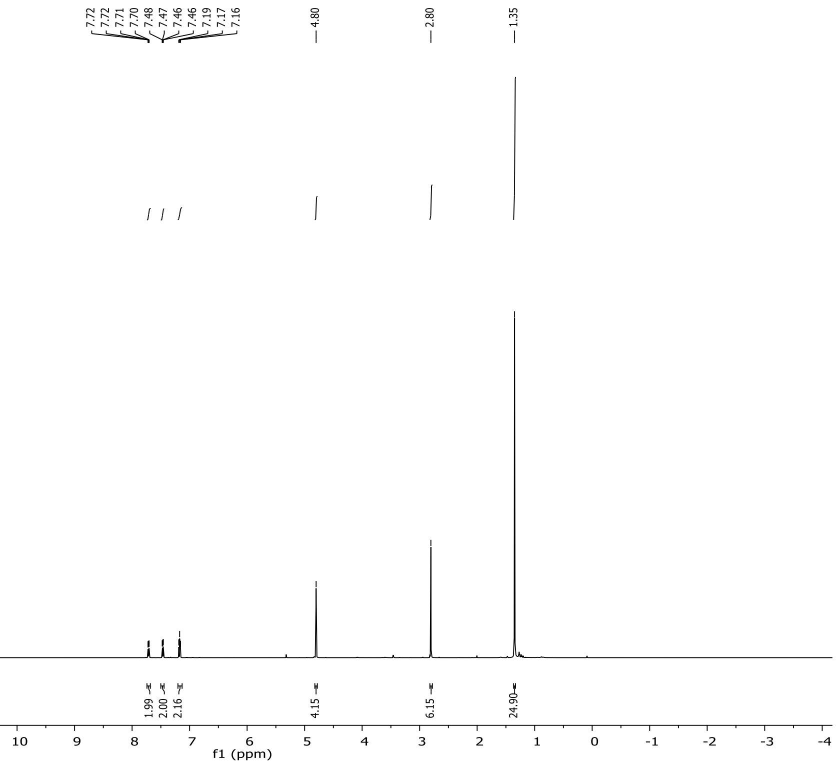

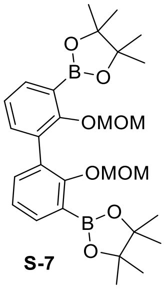

S-7

^{13}C NMR
(126 MHz, CD_2Cl_2)

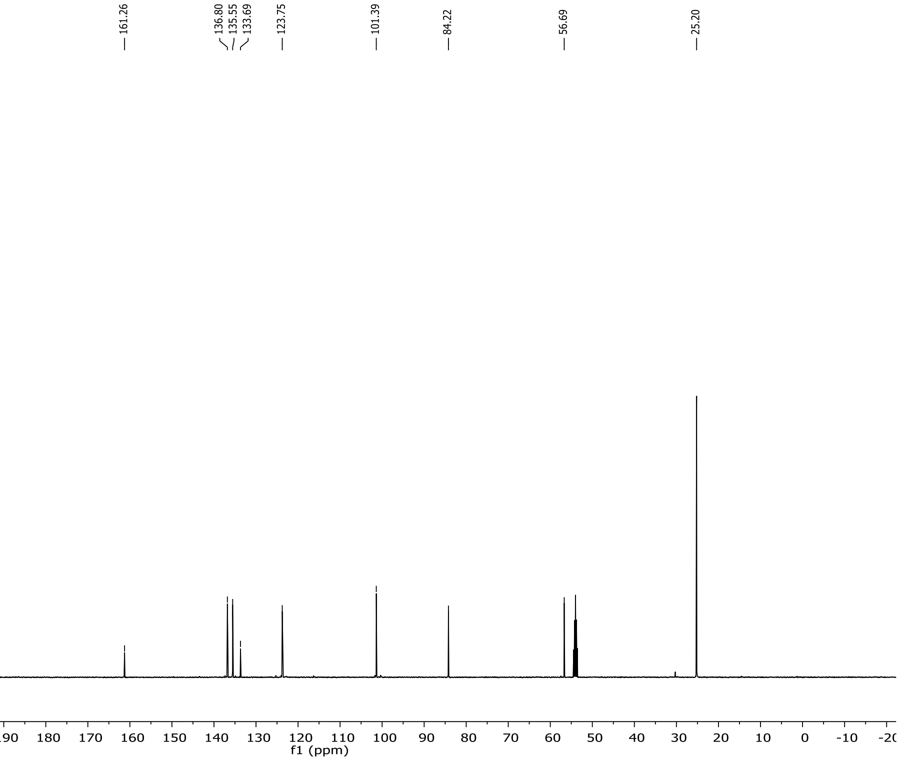

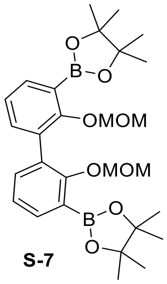

S-7

^{11}B NMR
(161 MHz, CD_2Cl_2)

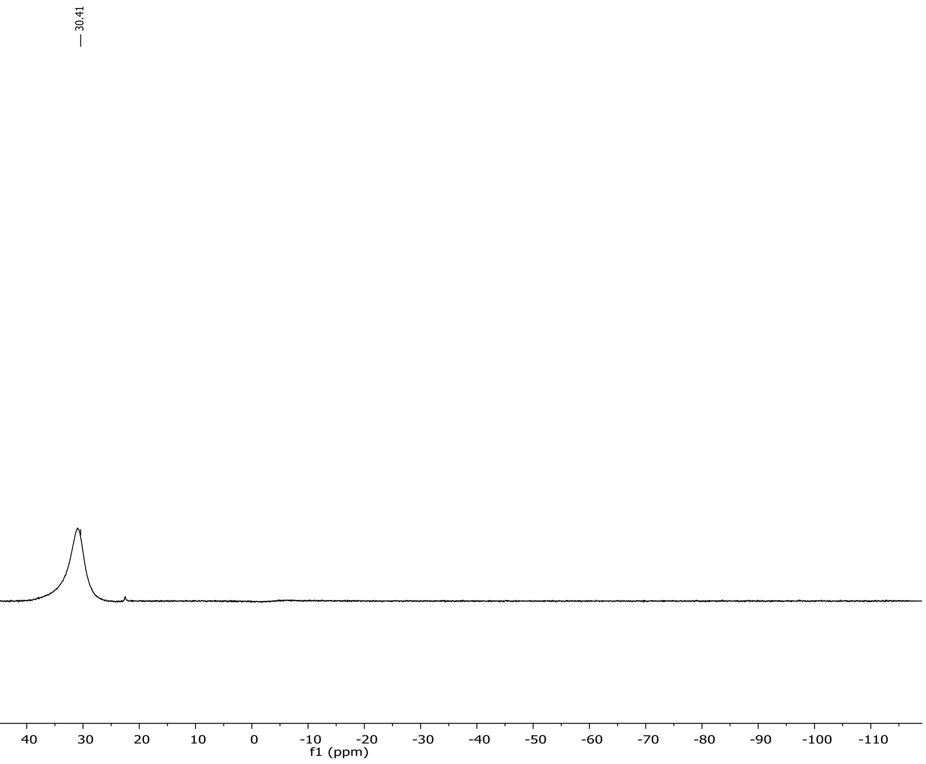

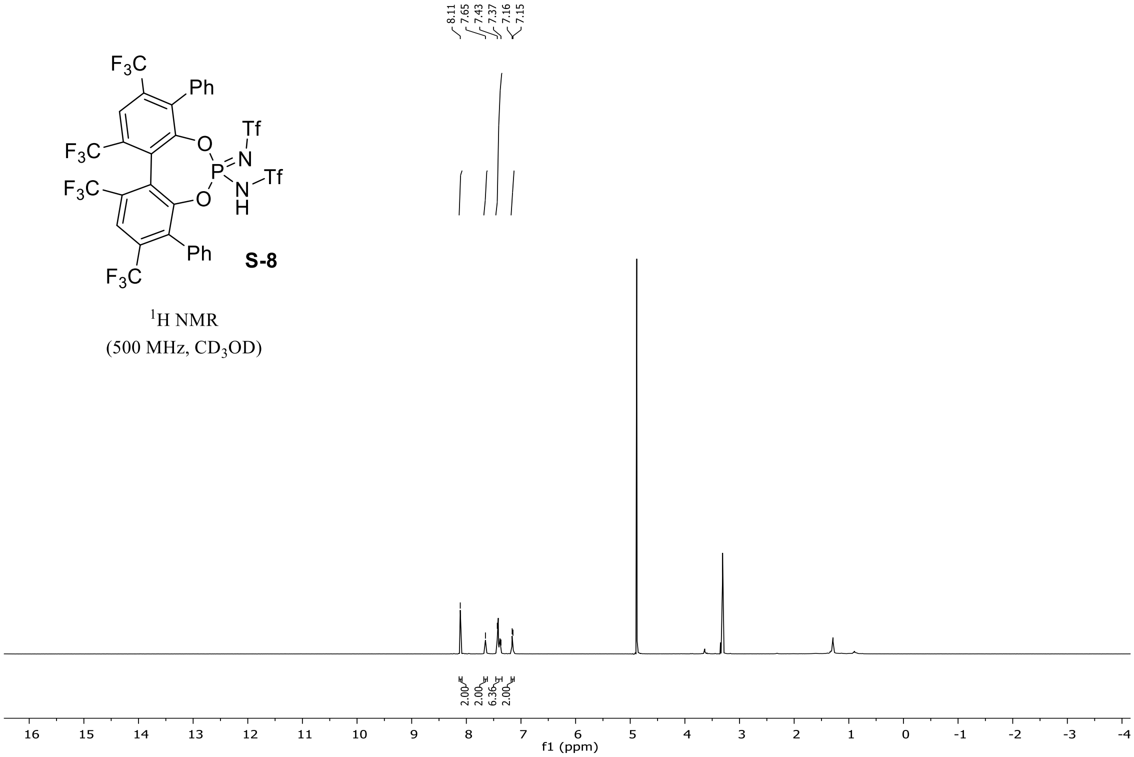

S-8

^{1}H NMR
(500 MHz, CD_3OD)

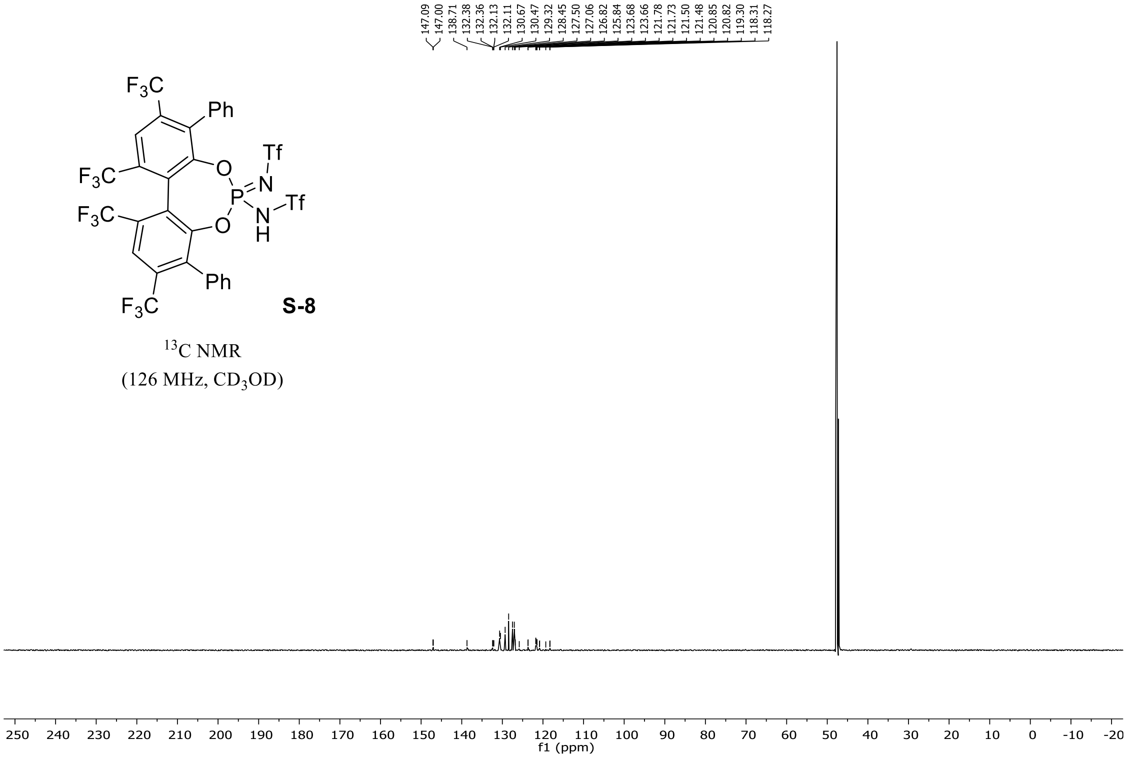

S-8

^{13}C NMR
(126 MHz, CD_3OD)

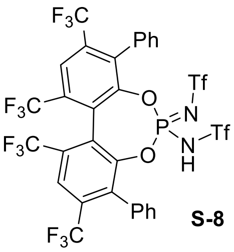

S-8

^{31}P NMR
(203 MHz, CD_3OD)

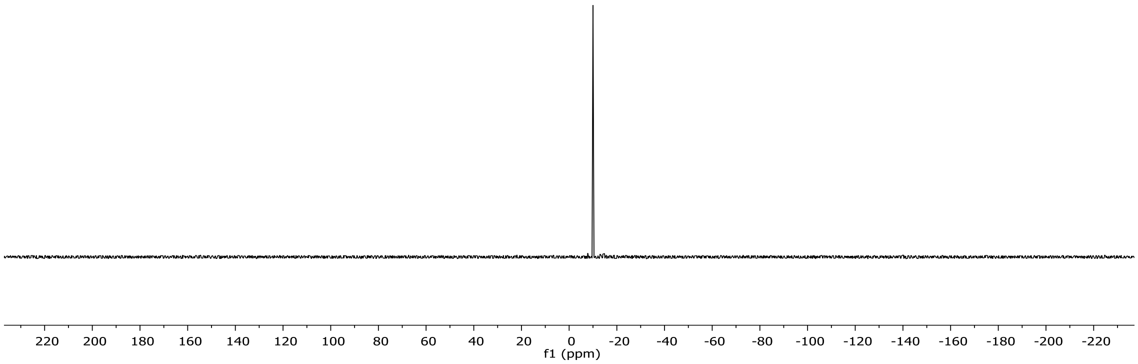

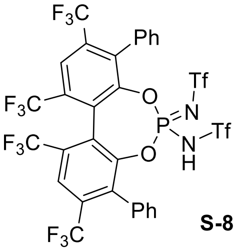

S-8

^{19}F NMR
(471 MHz, CD_3OD)

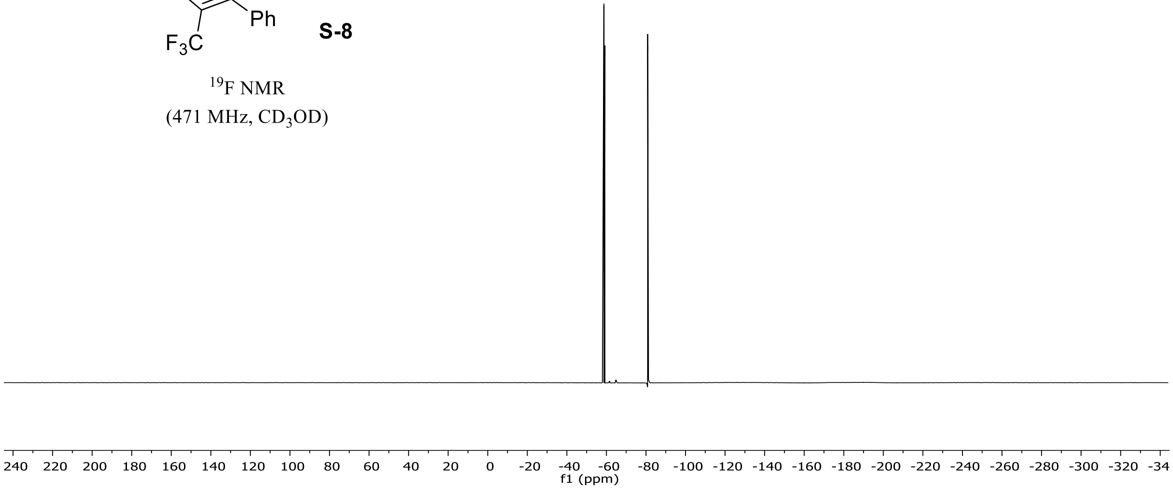

S-9

^{1}H NMR
(500 MHz, $CDCl_3$)

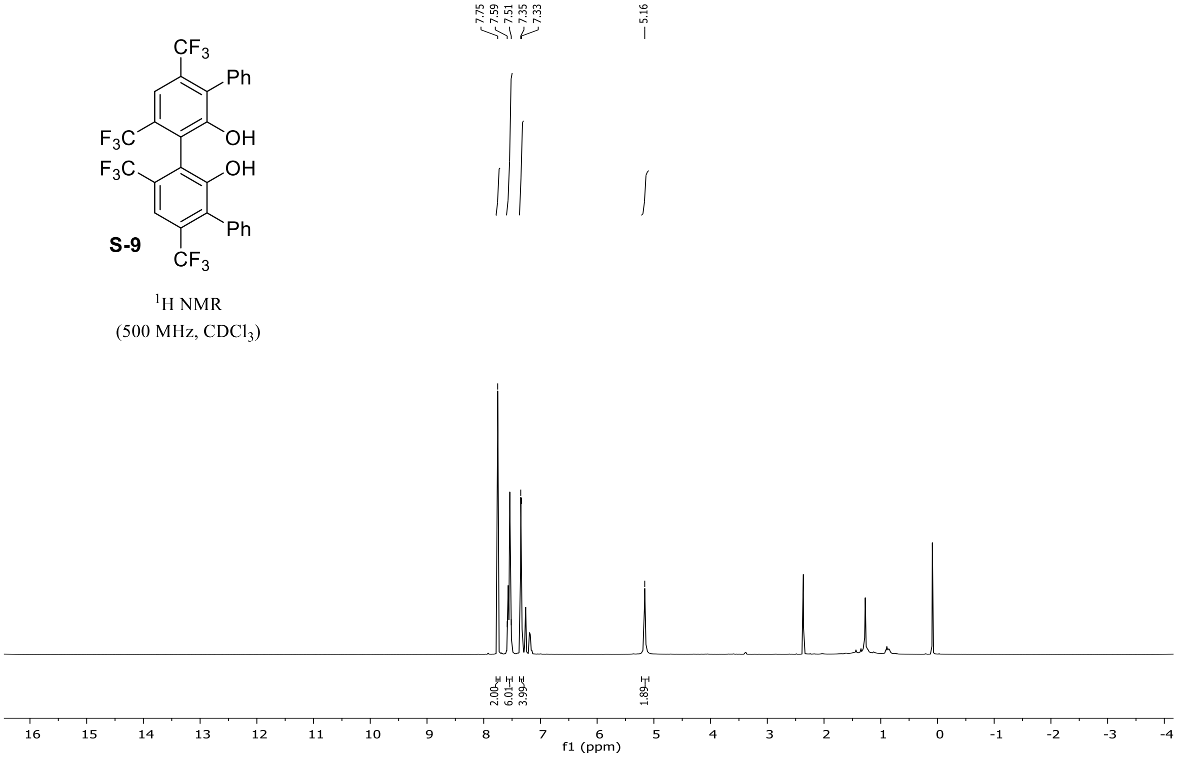

S-9

^{13}C NMR
(126 MHz, $CDCl_3$)

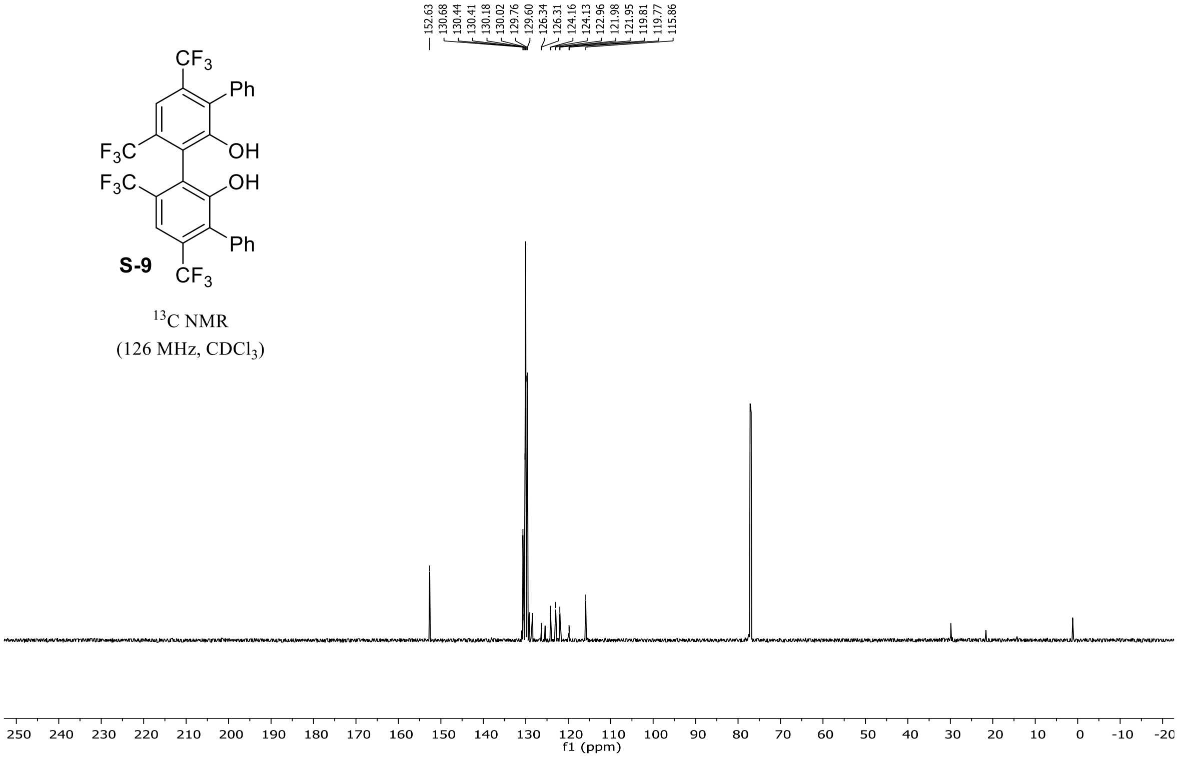

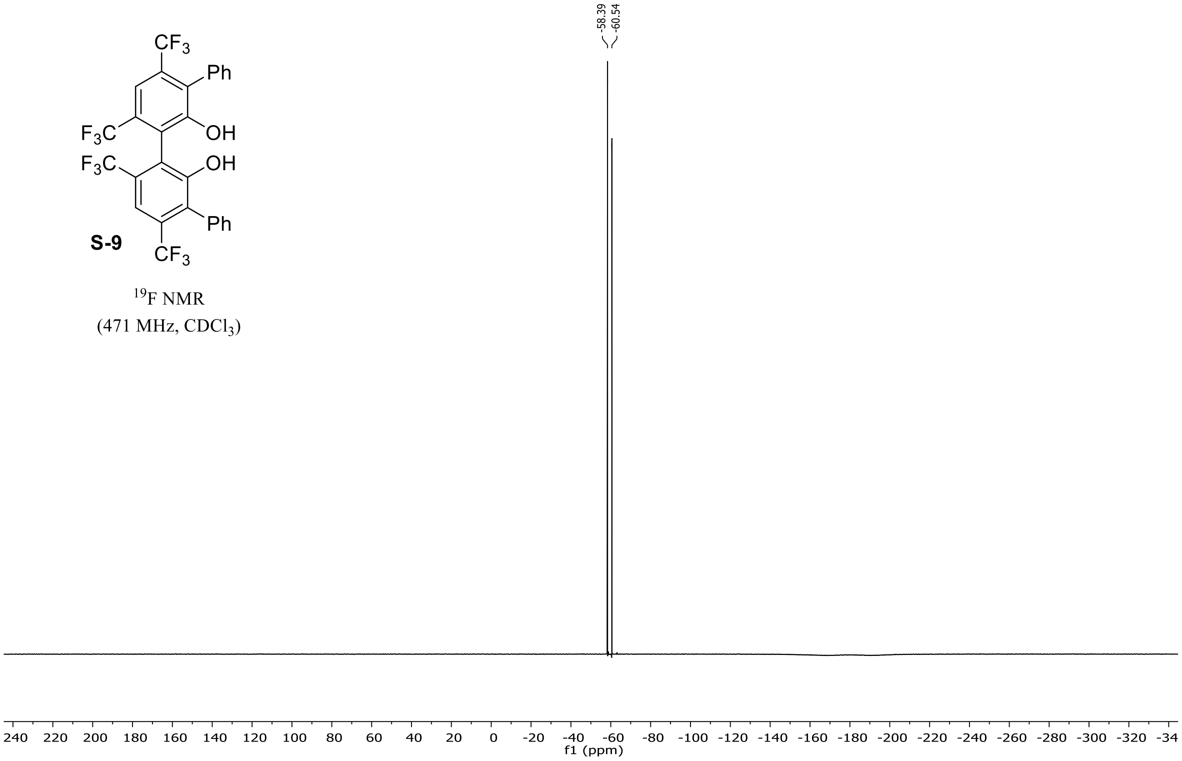

S-9

^{19}F NMR
(471 MHz, $CDCl_3$)

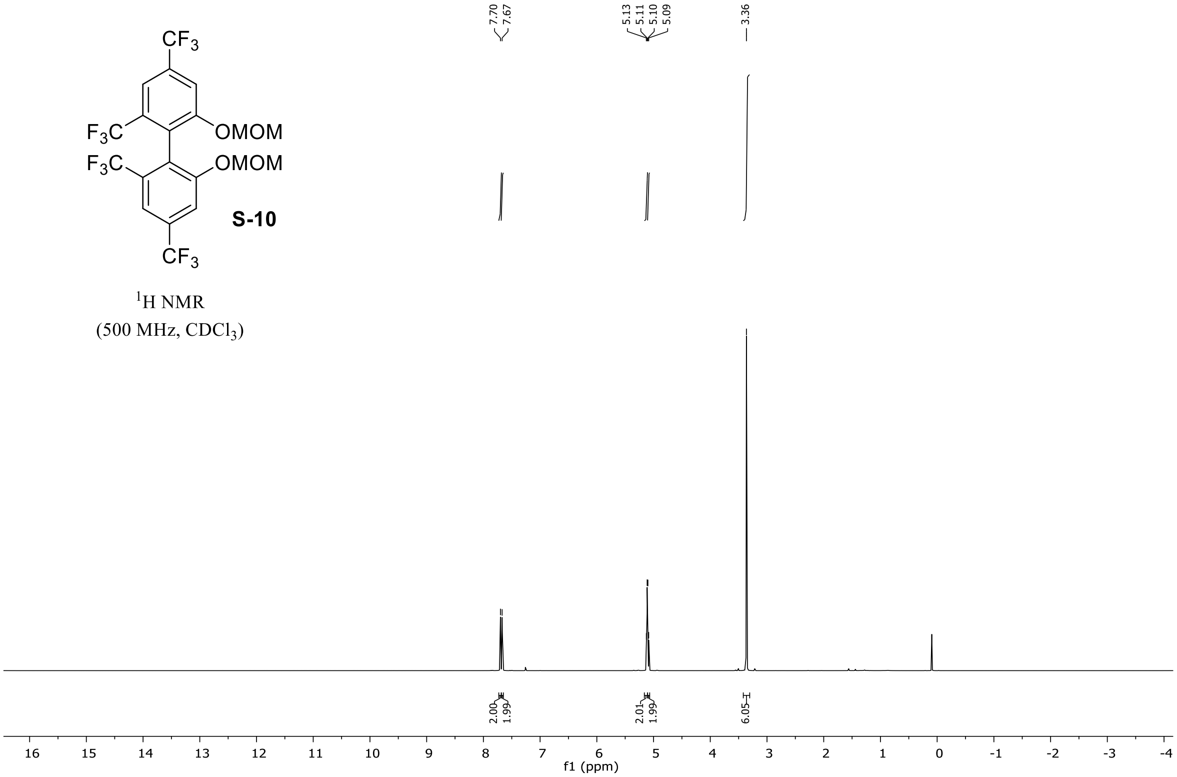

S-10

^{1}H NMR
(500 MHz, $CDCl_3$)

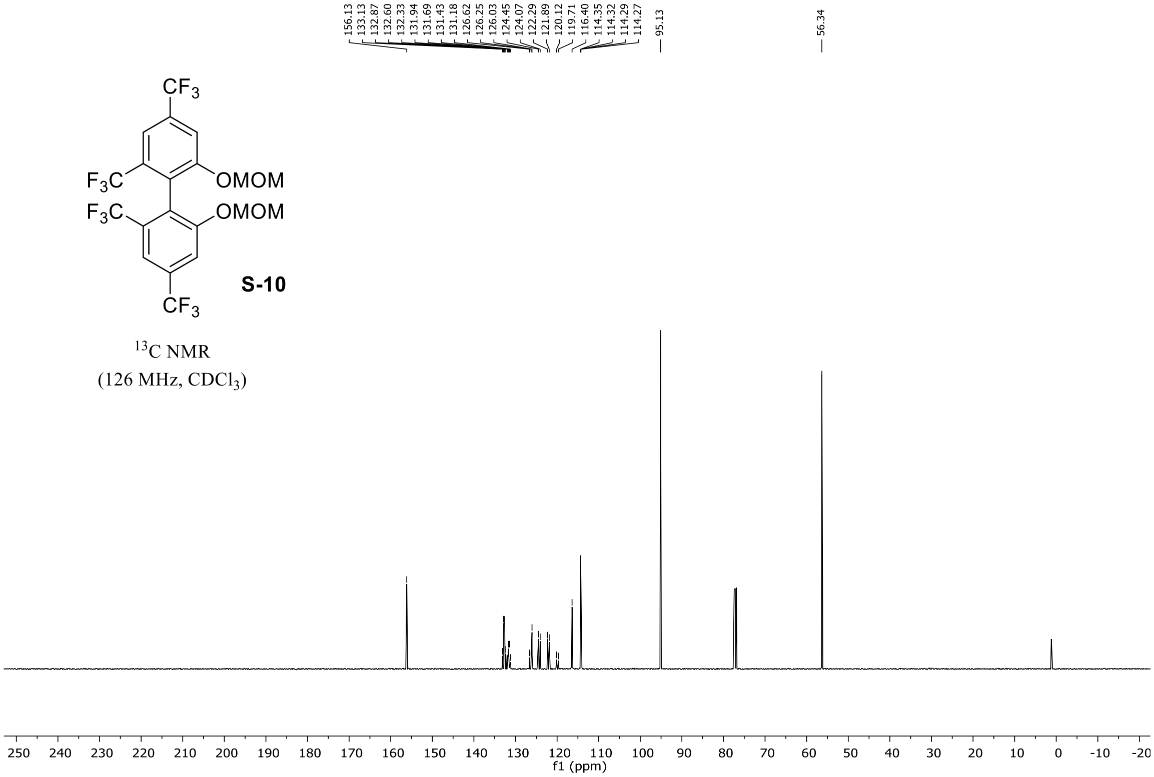

S-10

^{13}C NMR
(126 MHz, $CDCl_3$)

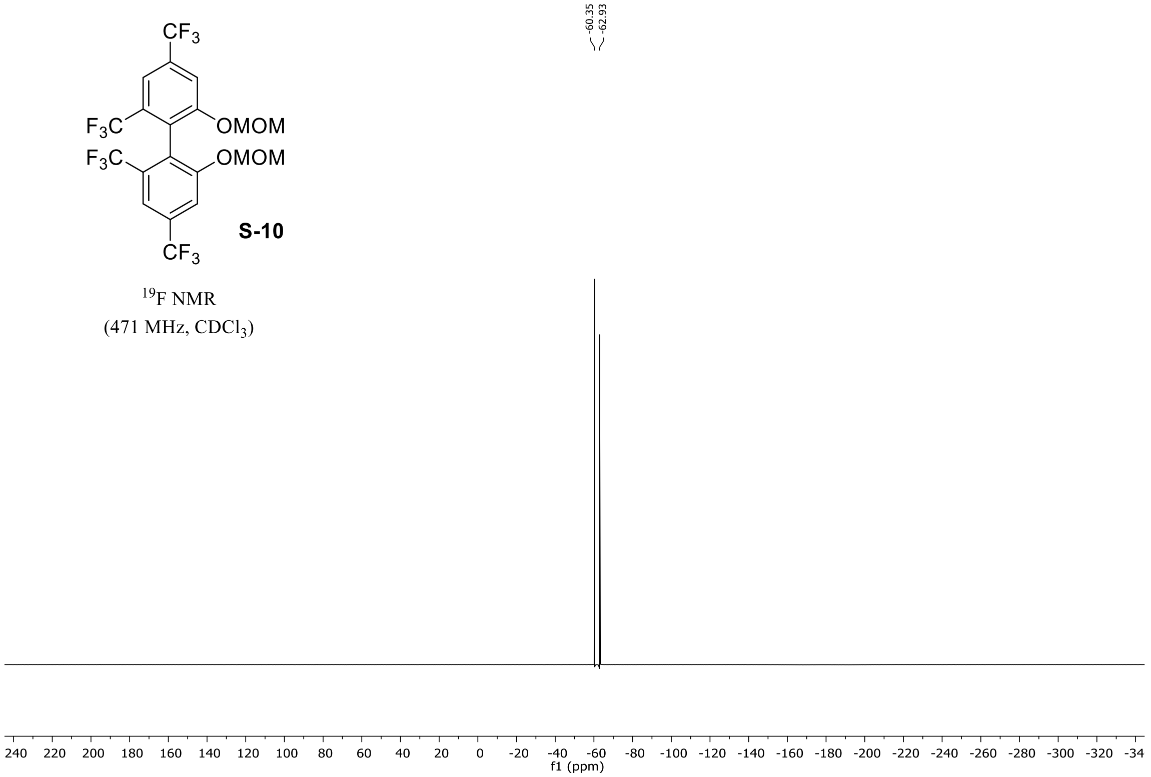

S-10

^{19}F NMR
(471 MHz, $CDCl_3$)

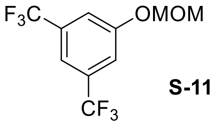

S-11

^{1}H NMR
(500 MHz, $CDCl_3$)

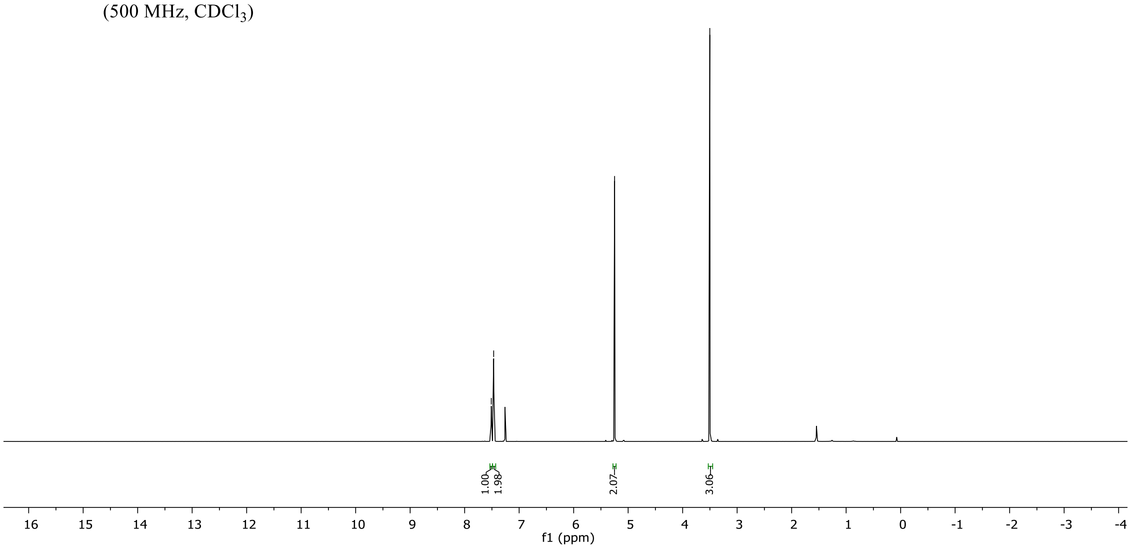

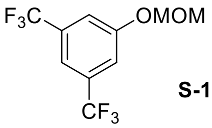

S-11

^{13}C NMR
(126 MHz, $CDCl_3$)

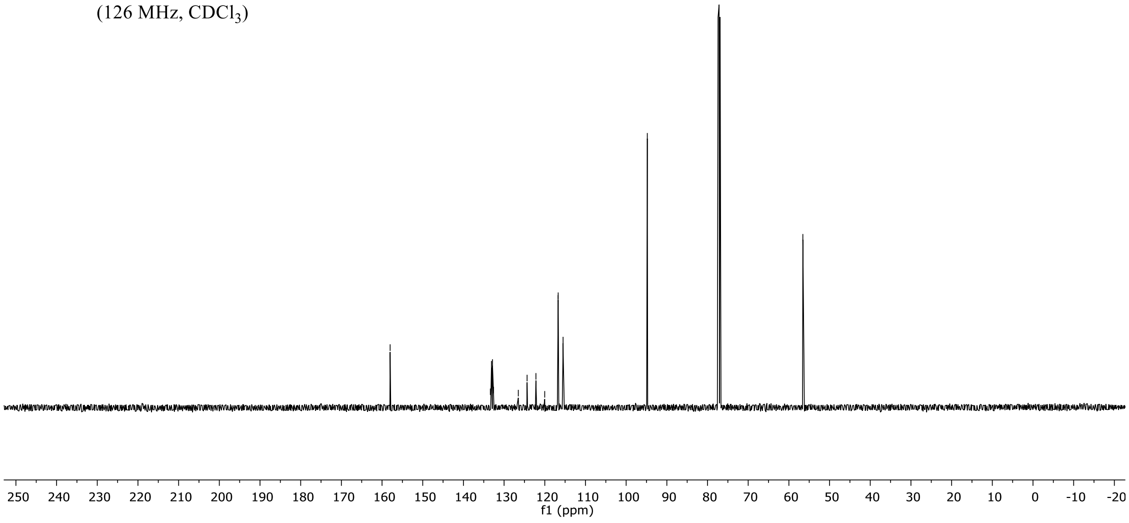

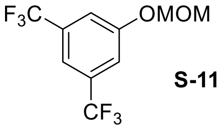

S-11

^{19}F NMR
(471 MHz, $CDCl_3$)

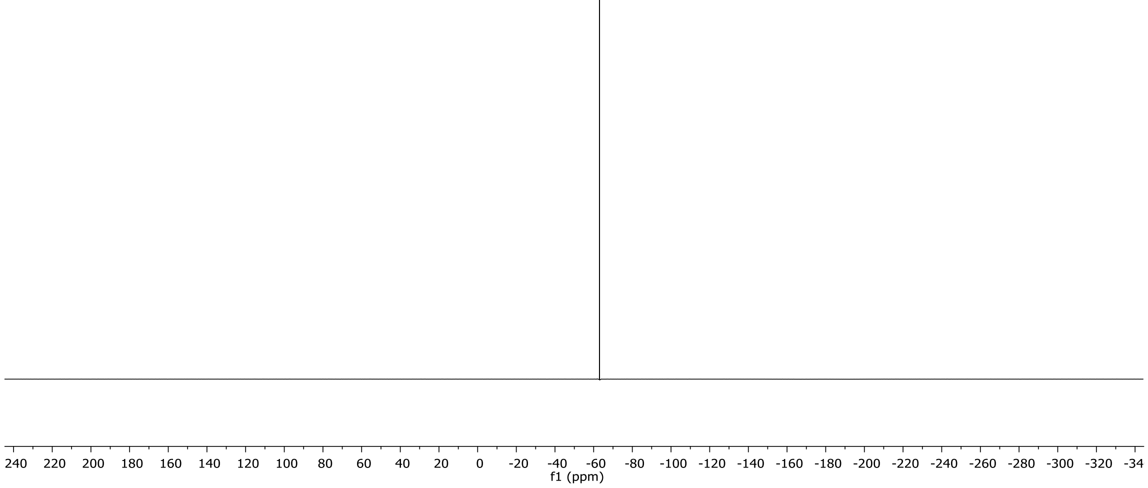

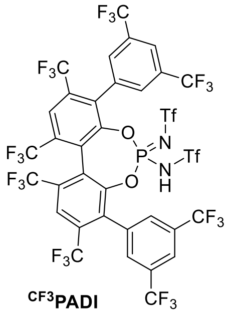

^{CF3}PADI

^{1}H NMR
(500 MHz, CD_3OD)

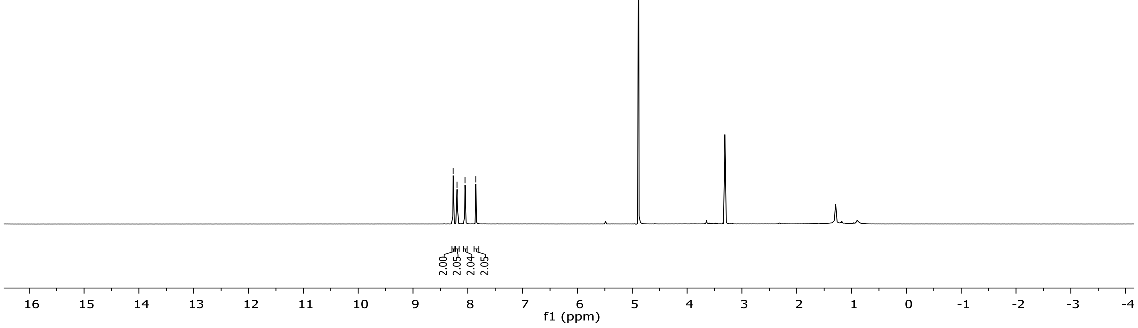

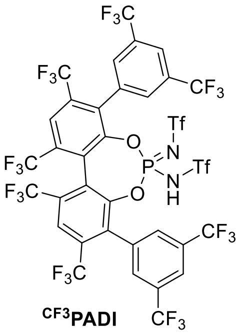

^{CF3}PADI

^{13}C NMR
(126 MHz, CD_3OD)

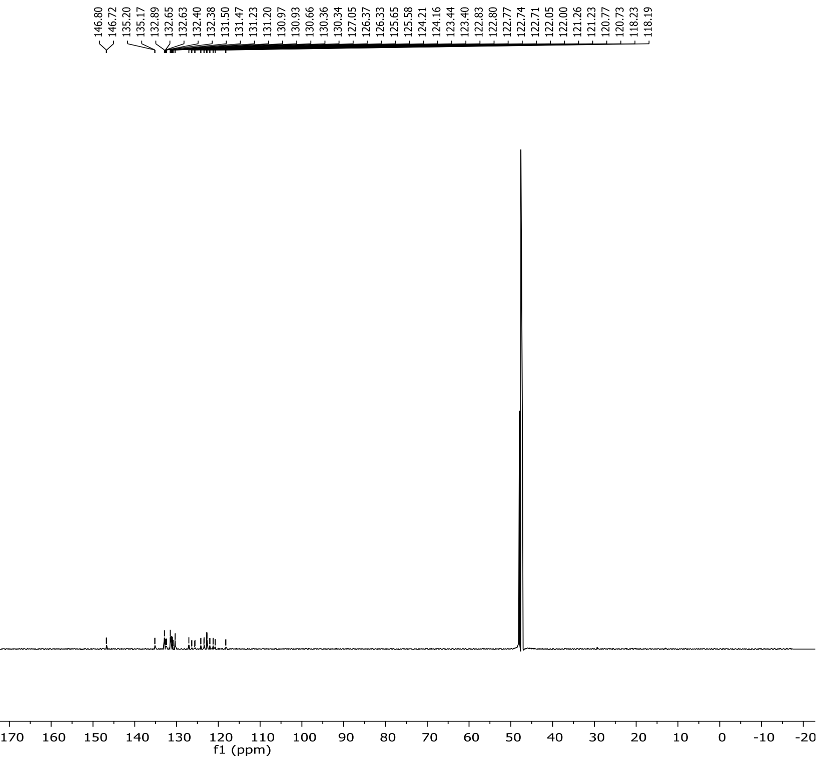

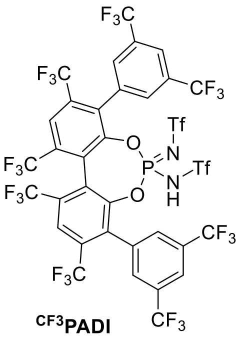

^{CF3}PADI

^{31}P NMR
(203 MHz, CD_3OD)

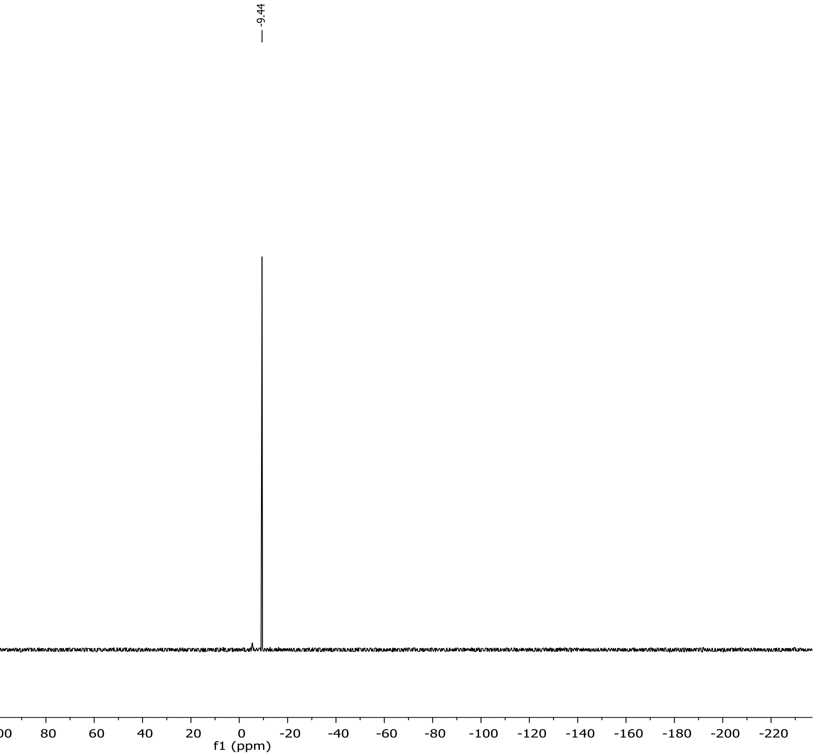

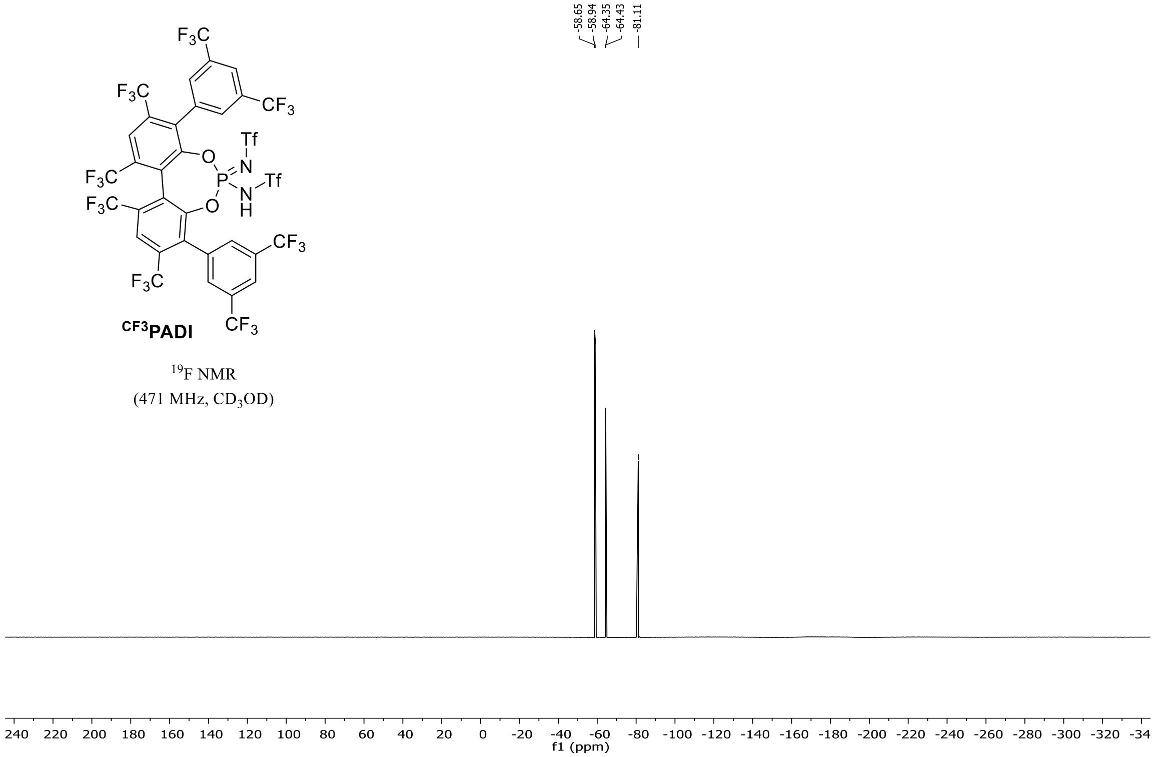
CF3PADI
19F NMR
(471 MHz, CD3OD)
-58.65
-58.94
-64.35
-64.43
-81.11
240 220 200 180 160 140 120 100 80 60 40 20 0 -20 -40 -60 -80 -100 -120 -140 -160 -180 -200 -220 -240 -260 -280 -300 -320 -34
f1 (ppm)

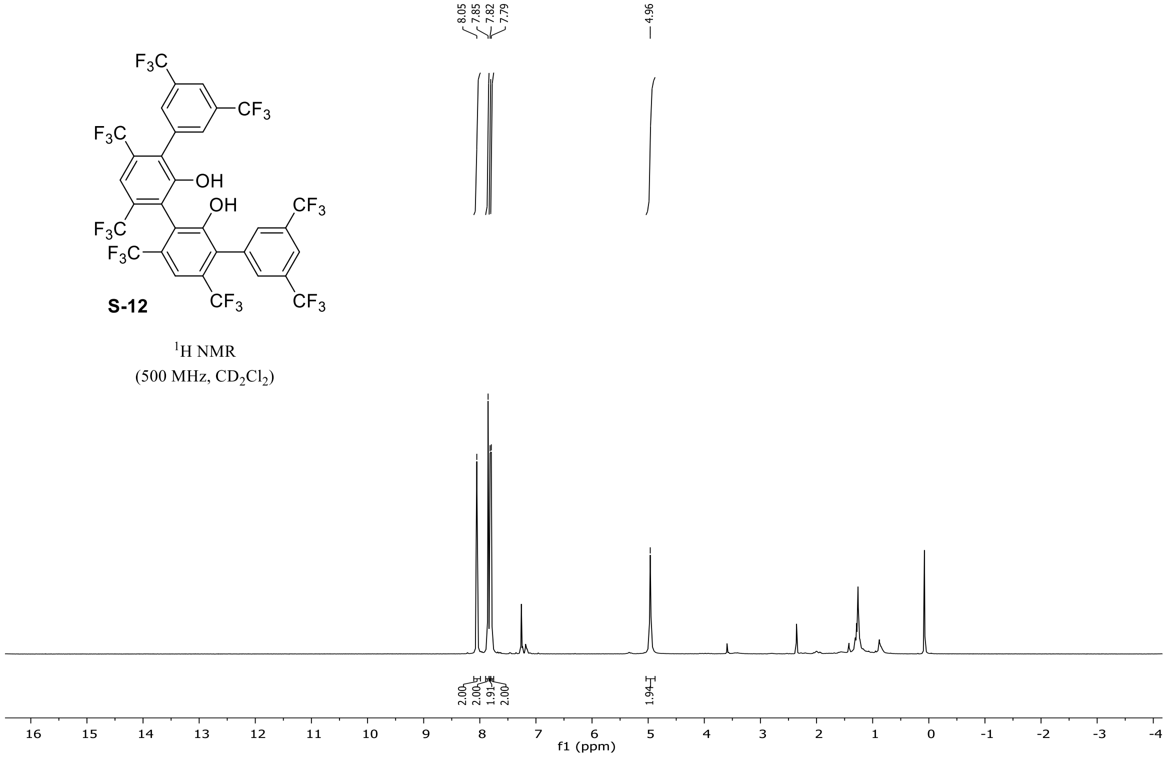
S-12
1H NMR
(500 MHz, CD2Cl2)
8.05
7.85
7.82
7.79
4.96
2.00
2.00
1.91
2.00
1.94
16 15 14 13 12 11 10 9 8 7 6 5 4 3 2 1 0 -1 -2 -3 -4
f1 (ppm)

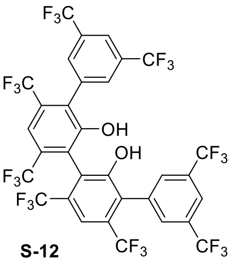

S-12

^{13}C NMR
(126 MHz, CD_2Cl_2)

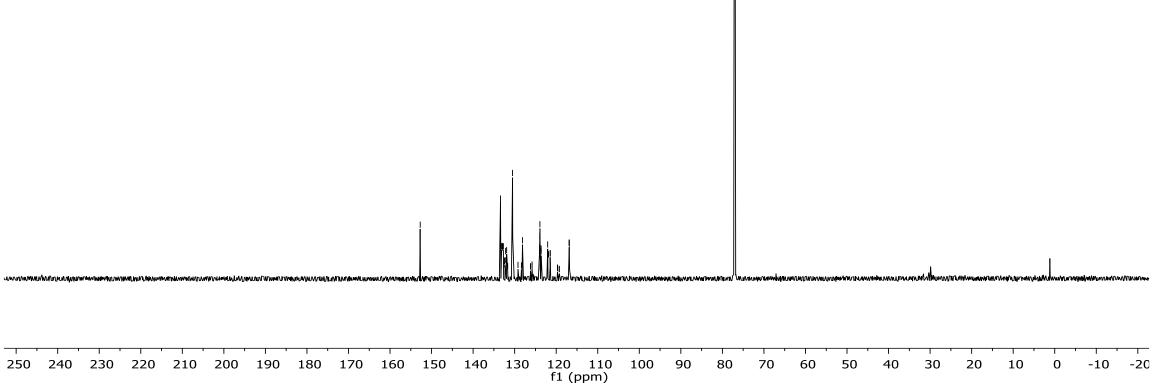

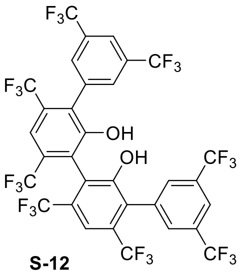

S-12

^{19}F NMR
(471 MHz, CD_2Cl_2)

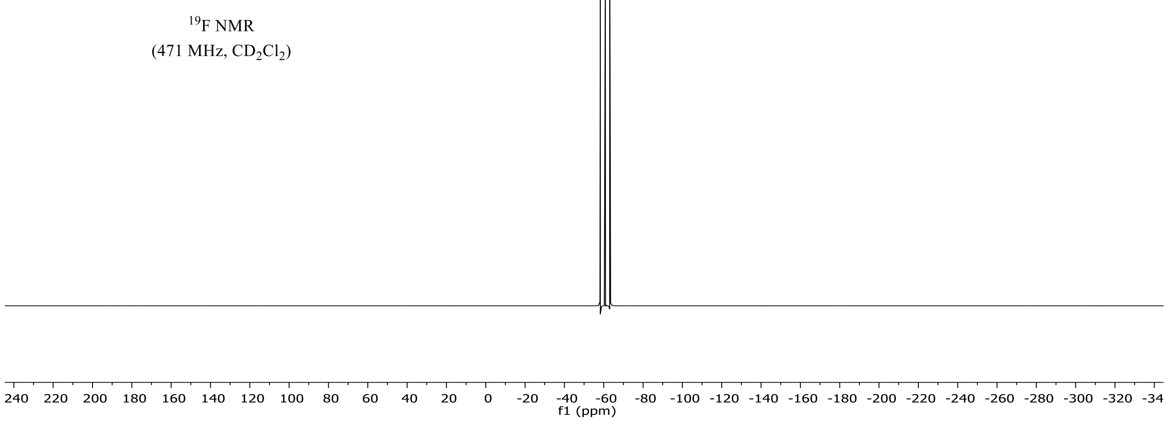

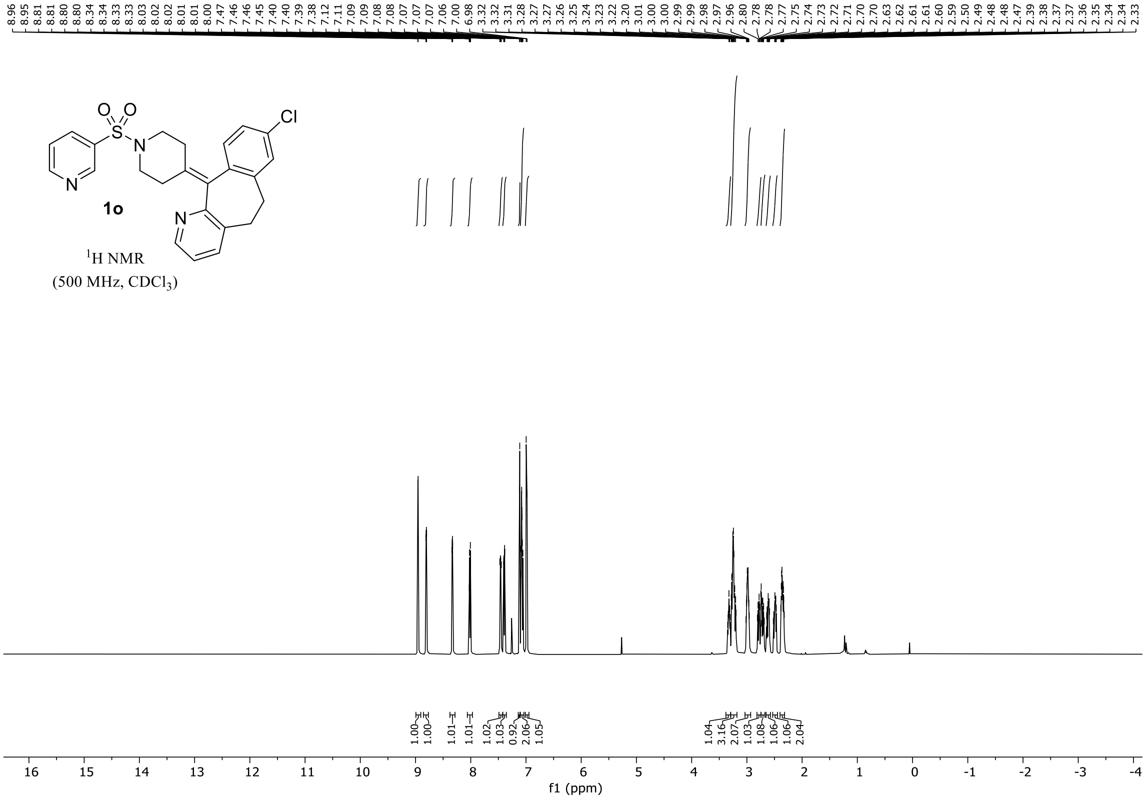
8.96 8.95 8.81 8.80 8.80 8.34 8.34 8.33 8.33 8.03 8.02 8.02 8.01 8.01 8.00 7.47 7.46 7.45 7.40 7.40 7.39 7.38 7.12 7.11 7.09 7.09 7.08 7.08 7.07 7.07 7.06 7.00 6.98 3.32 3.31 3.28 3.27 3.27 3.26 3.25 3.24 3.23 3.22 3.20 3.01 3.00 3.00 2.99 2.99 2.98 2.97 2.96 2.80 2.78 2.78 2.77 2.75 2.74 2.73 2.72 2.71 2.70 2.70 2.63 2.62 2.61 2.61 2.60 2.59 2.50 2.49 2.48 2.47 2.39 2.38 2.37 2.37 2.36 2.35 2.34 2.34 2.33
Cl
N
N
1o
^{1}H NMR
(500 MHz, $CDCl_3$)
1.00 1.00 1.01 1.01 1.02 1.03 0.92 2.06 1.05
1.04 3.16 2.07 1.03 1.08 1.06 1.06 2.04
16 15 14 13 12 11 10 9 8 7 6 5 4 3 2 1 0 -1 -2 -3 -4
f1 (ppm)

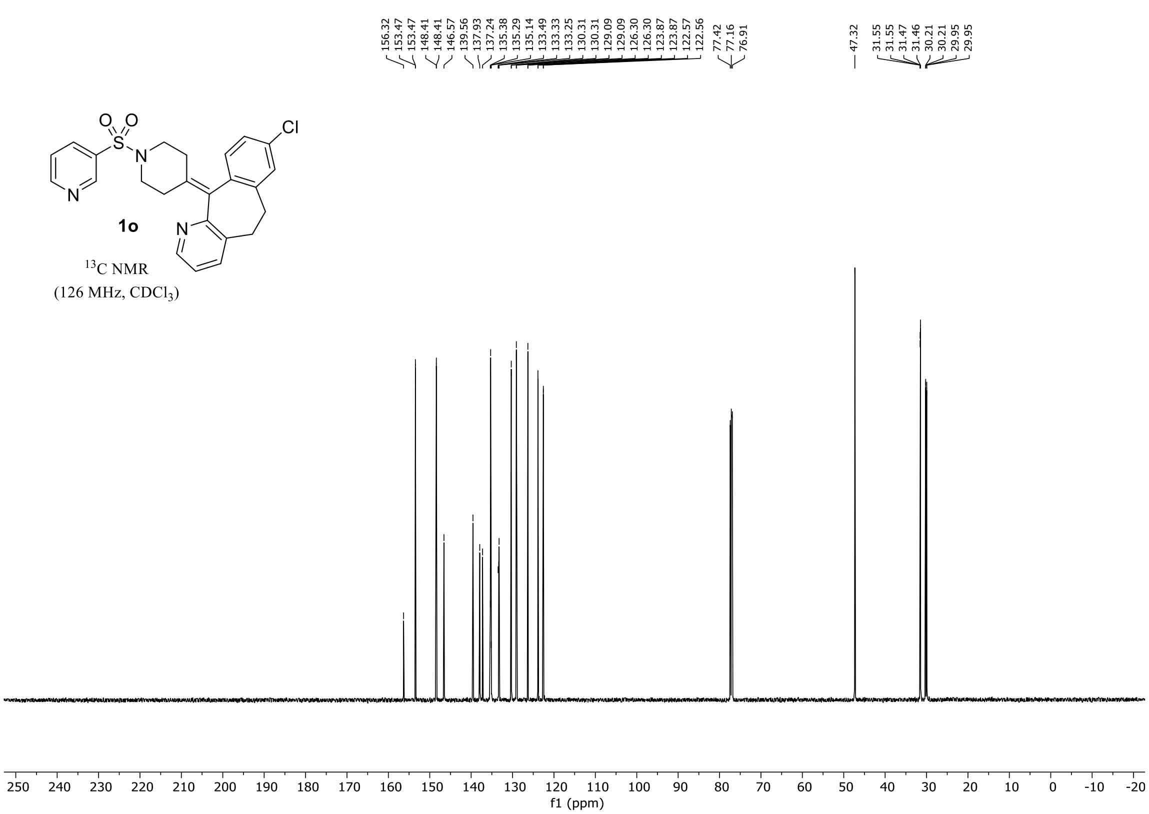
156.32 153.47 153.47 148.41 148.41 146.57 139.56 137.93 137.24 135.38 135.29 135.14 133.49 133.33 133.25 130.31 130.31 129.09 129.09 126.30 126.30 123.87 122.57 122.56 77.42 77.16 76.91
47.32 31.55 31.55 31.47 31.46 30.21 30.21 29.95 29.95
Cl
N
N
1o
^{13}C NMR
(126 MHz, $CDCl_3$)
250 240 230 220 210 200 190 180 170 160 150 140 130 120 110 100 90 80 70 60 50 40 30 20 10 0 -10 -20
f1 (ppm)

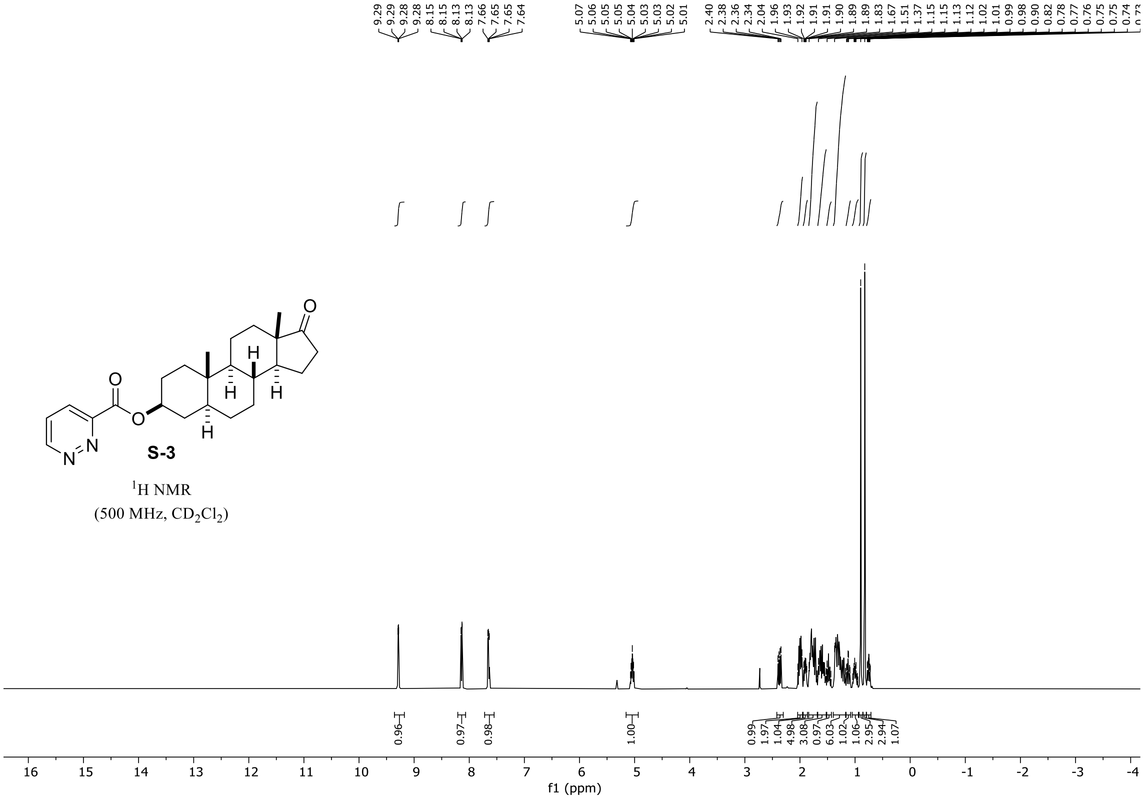

S-3

^{1}H NMR
(500 MHz, CD_2Cl_2)

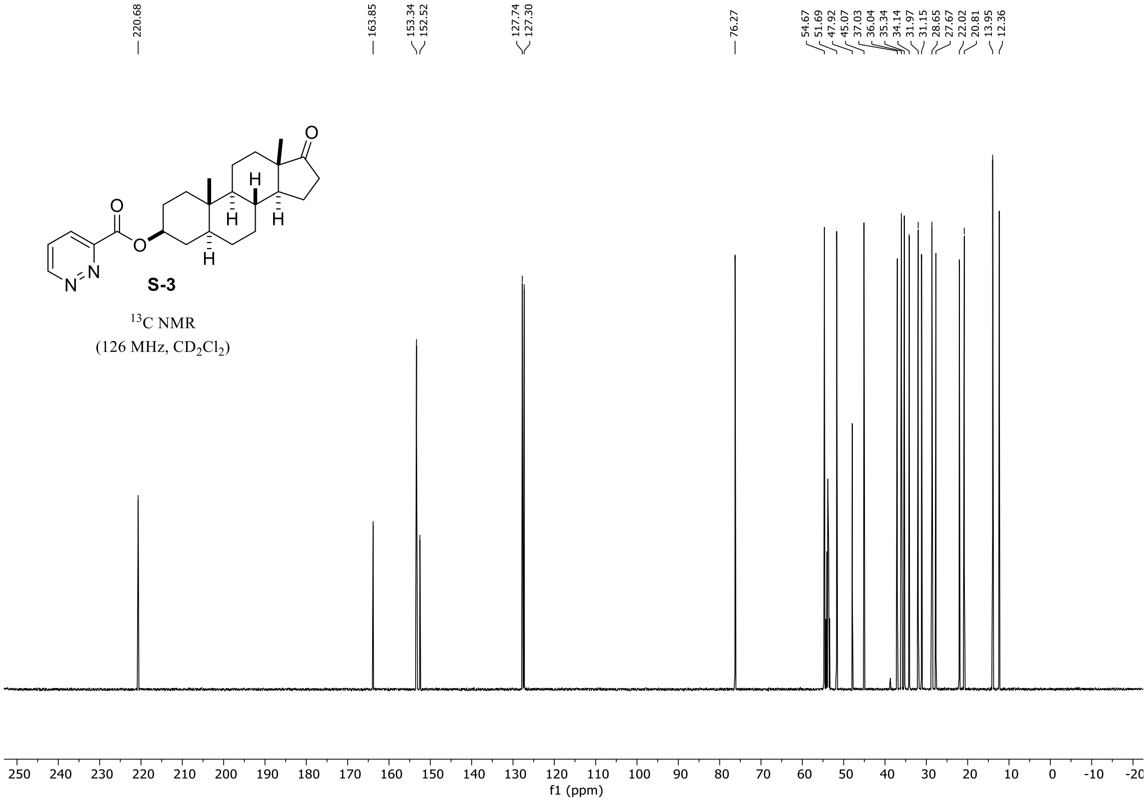

S-3

^{13}C NMR
(126 MHz, CD_2Cl_2)

10. HPLC Data

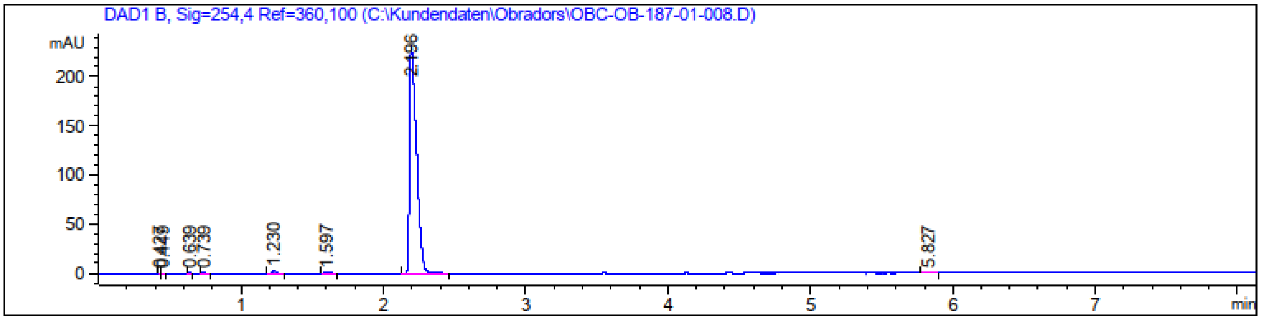

Peak #	RetTime [min]	Type	Width [min]	Area [mAU*s]	Height [mAU]	Area %	
1	0.427	VV	0.0121	1.76520e-1	2.14989e-1	0.0222	
2	0.449	VV	0.0194	2.06760e-1	1.51362e-1	0.0260	
3	0.639	VBA	0.0136	3.77851e-1	4.57332e-1	0.0475	
4	0.739	VV	0.0174	5.67454e-1	4.94580e-1	0.0713	
5	1.230	BB	0.0251	3.98660	2.43821	0.5007	
6	1.597	VB	0.0326	1.59080	6.97796e-1	0.1998	
7	2.196	BB	0.0507	788.39081	231.53247	99.0240	product
8	5.827	BB	0.0356	8.64765e-1	3.52680e-1	0.1086	

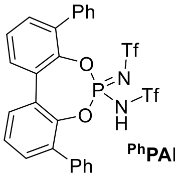

PhPADI

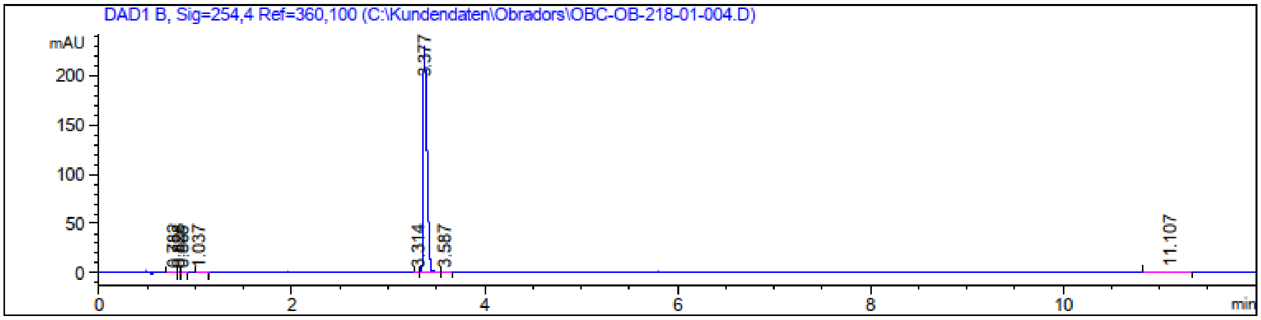

Peak #	RetTime [min]	Type	Width [min]	Area [mAU*s]	Height [mAU]	Area %	
1	0.782	BV	0.0371	9.19163e-1	3.09767e-1	0.1531	
2	0.828	VV	0.0265	8.24246e-1	4.57570e-1	0.1373	
3	0.866	VB	0.0320	3.91418e-1	1.52011e-1	0.0652	
4	1.037	VB	0.0288	5.11308e-1	2.45372e-1	0.0852	
5	3.314	BV	0.0294	5.33895e-1	2.35935e-1	0.0890	
6	3.377	VV	0.0400	593.77930	230.60573	98.9312	product
7	3.587	VB	0.0388	4.82867e-1	1.51189e-1	0.0805	
8	11.107	BB	0.1705	2.75223	1.92232e-1	0.4586	

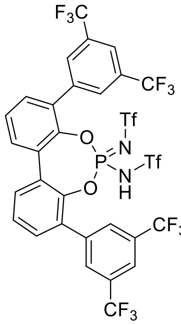

S-5

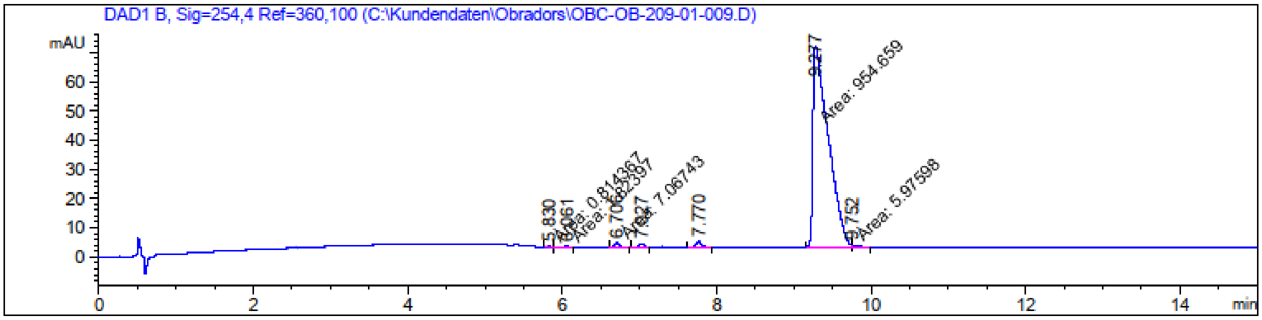

Peak #	RetTime [min]	Type	Width [min]	Area [mAU*s]	Height [mAU]	Area %	
1	5.830	FM	0.0501	8.14367e-1	2.70730e-1	0.0826	
2	6.061	MF	0.0569	1.82397	5.34157e-1	0.1851	
3	6.706	FM	0.0701	7.06743	1.68122	0.7171	
4	7.027	BV	0.0583	5.12985	1.22552	0.5205	
5	7.770	VB	0.0757	10.08289	1.95374	1.0231	
6	9.277	MF	0.2303	954.65894	69.10297	96.8653	product
7	9.752	FM	0.1119	5.97598	8.89902e-1	0.6064	

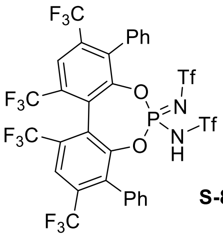

S-8

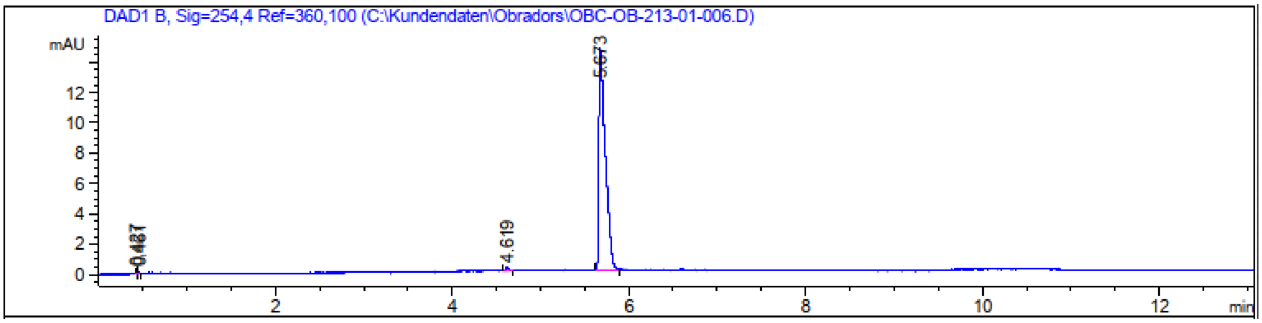

Peak #	RetTime [min]	Type	Width [min]	Area [mAU*s]	Height [mAU]	Area %	
1	0.427	VV	0.0132	1.57757e-1	1.80997e-1	0.2071	
2	0.461	VB	0.0167	1.70912e-1	1.50716e-1	0.2243	
3	4.619	BB	0.0255	4.41592e-1	2.34003e-1	0.5796	
4	5.673	BB	0.0694	75.41988	14.69386	98.9890	product

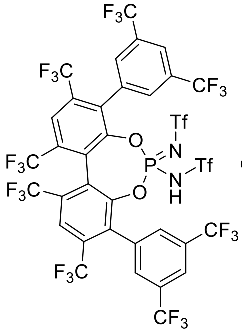

^{CF3}PADI

11. Crystallographic Data

Crystal structure analysis of *N*-silylated dihydropyridine (3a)

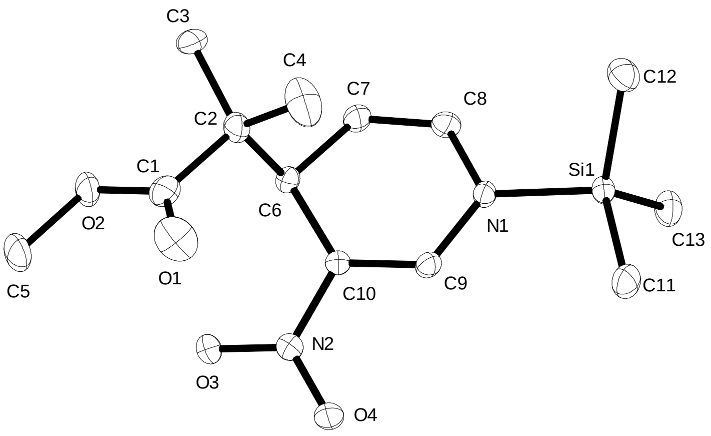

Figure S1a. Structure of **3a** in the solid state, showing the molecule and the atom numbering scheme. H atoms have been removed for clarity.

Table S1a. Crystal data and structure refinement.

Identification code	12545	
Empirical formula	$C_{13} H_{22} N_2 O_4 Si$	
Color	yellow prism	
Formula weight	298.41 g · mol^{-1}	
Temperature	100(2) K	
Wavelength	0.71073 Å	
Crystal system	Monoclinic	
Space group	$P2_1/c$, (no. 14)	
Unit cell dimensions	a = 10.595(6) Å	α = 90°.
	b = 21.520(7) Å	β = 106.08(4)°.
	c = 7.181(2) Å	γ = 90°.
Volume	1573.3(12) Å^3	
Z	4	
Density (calculated)	1.260 Mg · m^{-3}	
Absorption coefficient	0.163 mm^{-1}	
F(000)	640 e	
Crystal size	0.10 x 0.065 x 0.06 mm^3	
θ range for data collection	3.216 to 30.852°.	
Index ranges	$-15 \le h \le 15$, $-30 \le k \le 30$, $-10 \le l \le 10$	
Reflections collected	25819	
Independent reflections	4947 [R_{int} = 0.0807]	

Reflections with I>2σ(I)	3030
Completeness to θ = 25.242°	99.8 %
Absorption correction	Gaussian
Max. and min. transmission	0.99 and 0.99
Refinement method	Full-matrix least-squares on F^2
Data / restraints / parameters	4947 / 0 / 187
Goodness-of-fit on F2	1.110
Final R indices [I>2σ(I)]	R1 = 0.0691 wR^2 = 0.1196
R indices (all data)	R1 = 0.1310 wR^2 = 0.1404
Largest diff. peak and hole	0.4 and -0.4 e · $Å^{-3}$

INTENSITY STATISTICS FOR DATASET #

Resolution	#Data	#Theory	%Complete	Redundancy	Mean I	Mean I/s	Rmerge	Rsigma
Inf - 2.84	77	83	92.8	10.22	112.36	75.62	0.0283	0.0095
2.84 - 1.90	178	178	100.0	10.06	47.21	52.64	0.0305	0.0127
1.90 - 1.50	260	260	100.0	7.80	29.43	34.79	0.0370	0.0195
1.50 - 1.31	259	259	100.0	7.15	16.71	23.86	0.0553	0.0305
1.31 - 1.18	263	263	100.0	6.56	17.78	20.91	0.0611	0.0341
1.18 - 1.10	244	244	100.0	6.08	12.27	16.30	0.0787	0.0501
1.10 - 1.03	278	278	100.0	5.88	10.48	12.83	0.0941	0.0619
1.03 - 0.98	262	262	100.0	5.46	7.05	9.07	0.1342	0.0934
0.98 - 0.94	241	241	100.0	5.17	7.47	9.16	0.1306	0.0959
0.94 - 0.90	264	264	100.0	5.05	5.77	6.93	0.1814	0.1282
0.90 - 0.87	265	265	100.0	4.72	6.44	7.24	0.1655	0.1231
0.87 - 0.84	273	273	100.0	4.49	4.63	5.16	0.2343	0.1780
0.84 - 0.82	221	221	100.0	4.24	4.64	4.91	0.2431	0.1916
0.82 - 0.80	211	211	100.0	4.10	3.41	3.60	0.3114	0.2700
0.80 - 0.78	273	273	100.0	4.05	4.08	4.01	0.2804	0.2343
0.78 - 0.76	289	289	100.0	3.96	3.76	3.57	0.2990	0.2611
0.76 - 0.74	326	326	100.0	3.70	3.56	3.21	0.3168	0.2875
0.74 - 0.73	164	164	100.0	3.67	2.54	2.35	0.4207	0.4113
0.73 - 0.71	378	378	100.0	3.57	2.66	2.32	0.4038	0.4143
0.71 - 0.70	210	210	100.0	3.50	2.36	1.92	0.4527	0.4959
0.70 - 0.69	143	149	96.0	3.19	1.89	1.51	0.5347	0.6673
0.79 - 0.69	1638	1644	99.6	3.66	3.03	2.73	0.3611	0.3539
Inf - 0.69	5079	5091	99.8	5.16	10.84	11.83	0.0744	0.0766

Several low-angle reflections were shadowed by the beamstop and omitted from the data set before the final refinement cycles. The structure was solved by direct methods (SHELXT) and refined by full-matrix least-squares (SHELXL). The H atoms were calculated and refined using a riding model. CSD number: **CCDC-2055772.**

Table S2a. Bond lengths [Å] and angles [°].

Si(1)-N(1)	1.790(2)	Si(1)-C(13)	1.854(3)
Si(1)-C(11)	1.853(2)	Si(1)-C(12)	1.857(3)
O(3)-N(2)	1.247(2)	O(4)-N(2)	1.246(3)
O(2)-C(1)	1.334(3)	O(2)-C(5)	1.448(3)
O(1)-C(1)	1.206(3)	N(1)-C(9)	1.363(3)
N(1)-C(8)	1.415(3)	N(2)-C(10)	1.431(3)
C(10)-C(9)	1.356(3)	C(10)-C(6)	1.501(3)
C(6)-C(7)	1.508(3)	C(6)-C(2)	1.579(3)
C(8)-C(7)	1.334(3)	C(1)-C(2)	1.526(3)
C(2)-C(3)	1.536(4)	C(2)-C(4)	1.531(4)

N(1)-Si(1)-C(13)	108.07(11)	N(1)-Si(1)-C(11)	107.34(10)
N(1)-Si(1)-C(12)	105.72(11)	C(13)-Si(1)-C(12)	112.38(12)
C(11)-Si(1)-C(13)	111.10(13)	C(11)-Si(1)-C(12)	111.88(12)
C(1)-O(2)-C(5)	115.9(2)	C(9)-N(1)-Si(1)	124.73(15)
C(9)-N(1)-C(8)	115.48(18)	C(8)-N(1)-Si(1)	119.44(15)
O(3)-N(2)-C(10)	117.76(19)	O(4)-N(2)-O(3)	121.97(19)
O(4)-N(2)-C(10)	120.24(19)	N(2)-C(10)-C(6)	119.12(19)
C(9)-C(10)-N(2)	117.0(2)	C(9)-C(10)-C(6)	123.9(2)
C(10)-C(9)-N(1)	122.7(2)	C(10)-C(6)-C(7)	106.45(18)
C(10)-C(6)-C(2)	113.95(19)	C(7)-C(6)-C(2)	110.39(19)
C(7)-C(8)-N(1)	123.4(2)	C(8)-C(7)-C(6)	122.8(2)
O(2)-C(1)-C(2)	112.2(2)	O(1)-C(1)-O(2)	123.4(2)
O(1)-C(1)-C(2)	124.4(2)	C(1)-C(2)-C(6)	108.12(19)
C(1)-C(2)-C(3)	110.3(2)	C(1)-C(2)-C(4)	108.4(2)
C(3)-C(2)-C(6)	109.0(2)	C(4)-C(2)-C(6)	110.3(2)
C(4)-C(2)-C(3)	110.7(2)		

Crystal structure analysis of ^{Ph}PADI·Et$_3$N

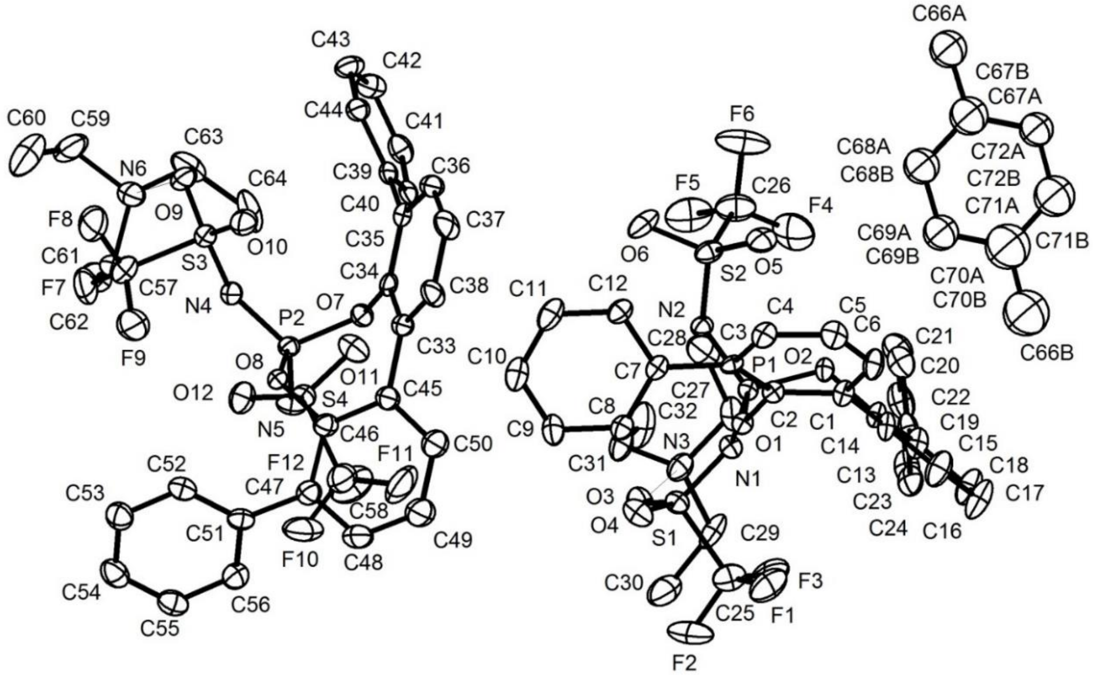

Figure S1b. Structure of ^{Ph}PADI·Et$_3$N in the solid state, showing the two independent molecules and the disordered toluene molecule in the asymmetric unit and the atom numbering scheme. H atoms have been removed for clarity.

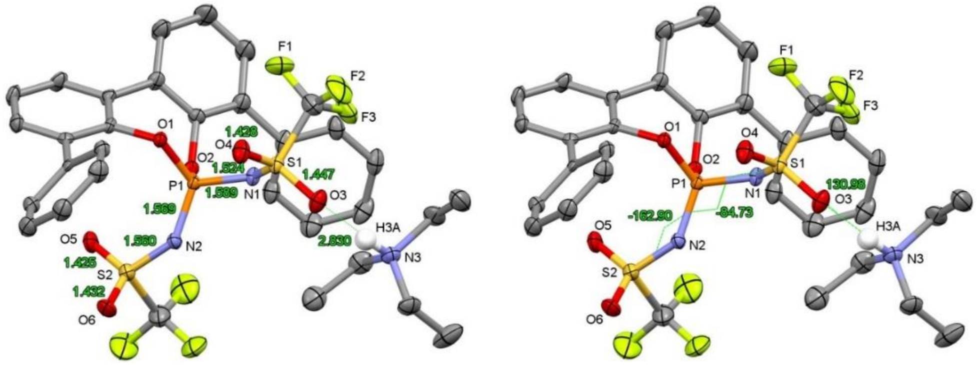

Figure S2b. Selected geometrical parameters of one of the two independent molecules. Distances are shown on the left and torsion angles and the S1-O3----N3 angle are shown on the right.

X-ray Crystal Structure Analysis of ^{Ph}PADI·Et$_3$N: 4(C_{26} H_{16} F_6 N_2 O_6 P S_2),4(C_6 H_{16} N) · C_7 H_8, Mr = 3146.91 g mol^{-1}, colorless prism crystallized from toluene, diethylether and pentane, crystal size 0.023 x 0.057 x 0.061 mm^3, monoclinic, space group $P2_1/c$ [No. 14], a = 17.4051(9) Å, b = 33.4925(15) Å, c = 12.5564(7) Å, β = 90.002(3)°, V = 7319.6(6) Å^3, T = 100(2) K, Z = 2, D_{calc} = 1.428 g cm^{-3}, λ = 0.71073 Å, μ(Mo-Kα) = 0.267 mm^{-1}, face indexed absorption correction, Bruker-AXS Kappa Mach3 APEX-II diffractometer, Helios X-ray optics and Mo rotating anode X-ray source, 1.170 < θ < 26.371°, 194716 measured reflections, 14966 independent reflections, 11112 reflections with $I > 2\sigma(I)$, R_{int} = 0.1252.

INTENSITY STATISTICS FOR DATASET

Resolution	#Data	#Theory	%Complete	Redundancy	Mean I	Mean I/s	Rmerge	Rsigma
Inf - 2.90	346	347	99.7	17.69	23.25	43.80	0.0245	0.0175
2.90 - 1.91	807	807	100.0	18.62	9.08	37.26	0.0503	0.0206
1.91 - 1.51	1159	1159	100.0	18.75	4.74	29.07	0.0815	0.0269
1.51 - 1.32	1135	1135	100.0	18.74	3.26	23.19	0.1137	0.0345
1.32 - 1.19	1234	1234	100.0	18.27	3.67	22.33	0.1168	0.0355
1.19 - 1.11	1082	1082	100.0	17.35	2.93	17.85	0.1465	0.0451
1.11 - 1.04	1229	1229	100.0	13.75	2.00	11.92	0.1978	0.0717
1.04 - 0.99	1098	1098	100.0	11.51	1.46	8.26	0.2653	0.1083
0.99 - 0.95	1067	1067	100.0	10.12	1.10	5.92	0.3348	0.1544
0.95 - 0.91	1251	1251	100.0	9.11	0.96	4.83	0.3724	0.1932
0.91 - 0.88	1089	1089	100.0	8.15	0.89	4.07	0.4029	0.2281
0.88 - 0.85	1245	1245	100.0	7.91	0.71	3.20	0.4686	0.2960
0.85 - 0.82	1454	1454	100.0	7.56	0.64	2.76	0.5267	0.3481
0.82 - 0.80	1067	1067	100.0	7.40	0.61	2.47	0.5593	0.3820
0.80 - 0.78	1182	1182	100.0	7.13	0.58	2.27	0.5747	0.4242
0.78 - 0.76	1348	1348	100.0	6.93	0.45	1.71	0.6754	0.5640
0.76 - 0.75	716	717	99.9	6.76	0.46	1.73	0.6576	0.5712
0.75 - 0.73	1539	1540	99.9	6.63	0.37	1.36	0.7425	0.7216
0.73 - 0.72	843	846	99.6	6.43	0.36	1.27	0.7602	0.7944
0.72 - 0.71	873	873	100.0	6.30	0.33	1.15	0.8076	0.8899
0.71 - 0.70	1121	1412	79.4	4.29	0.27	0.88	0.8384	1.3187
0.80 - 0.70	7622	7918	96.3	6.29	0.40	1.49	0.7065	0.6911
Inf - 0.70	22885	23182	98.7	10.55	1.98	9.38	0.1400	0.1065

Several low-angle reflections were shadowed by the beamstop and removed from the data set before the final refinement cycles. The structure was solved by direct methods (SHELX86) and refined by full-matrix least-squares (SHELXL) against F^2 to $R_1 = 0.0472$ [$I > 2\sigma(I)$], w$R_2 = 0.1219$ [all data], 939 parameters, 16 restraints. The crystal contains a disordered toluene molecule with a half occupancy. Equivalent carbon-carbon distances in the toluene molecule were restrained to be equal with an effective standard deviation of 0.01. The H atoms were calculated and refined using a riding model. The crystal is twinned presumably owing to the beta angle being close to 90 degrees. The twinning law is [-1 0 0 0 -1 0 0 0 1] and the refined batch scale factor is 0.4923(8). The structure was refined using data to 0.8 A resolution where the mean I/sigma(I) is ca. 2.47. Goodness-of-fit on F^2 = 1.022. Maximum residual electron density 0.68 eÅ^{-3} [0.71 Å from C69B] minimum residual electron density -0.36 eÅ^{-3} [0.57 Å from S3]. CSD number: **CCDC-2055774**.

Table S1b. Atomic coordinates and equivalent isotropic displacement parameters (Å^2).

U_{eq} is defined as one third of the trace of the orthogonalized U_{ij} tensor.

	x	y	z	U_{eq}
C(1)	0.4642(2)	0.2908(1)	0.1942(3)	0.019(1)
C(2)	0.4179(2)	0.3103(1)	0.2682(3)	0.016(1)
C(3)	0.3678(2)	0.2904(1)	0.3369(3)	0.017(1)
C(4)	0.3673(2)	0.2485(1)	0.3318(3)	0.022(1)
C(5)	0.4132(2)	0.2283(1)	0.2599(3)	0.025(1)
C(6)	0.4605(2)	0.2491(1)	0.1908(3)	0.025(1)

C(7)	0.3144(2)	0.3112(1)	0.4099(3)	0.016(1)
C(8)	0.2678(2)	0.3428(1)	0.3765(3)	0.021(1)
C(9)	0.2124(2)	0.3580(1)	0.4440(3)	0.029(1)
C(10)	0.2025(2)	0.3433(1)	0.5447(3)	0.031(1)
C(11)	0.2485(2)	0.3119(1)	0.5798(3)	0.031(1)
C(12)	0.3046(2)	0.2965(1)	0.5128(3)	0.022(1)
C(13)	0.5133(2)	0.3126(1)	0.1176(3)	0.021(1)
C(14)	0.5611(2)	0.3438(1)	0.1501(3)	0.020(1)
C(15)	0.6099(2)	0.3640(1)	0.0812(3)	0.027(1)
C(16)	0.6081(3)	0.3524(1)	-0.0266(4)	0.037(1)
C(17)	0.5606(3)	0.3218(1)	-0.0602(4)	0.043(1)
C(18)	0.5146(3)	0.3019(1)	0.0112(3)	0.034(1)
C(19)	0.6632(2)	0.3960(1)	0.1168(3)	0.024(1)
C(20)	0.7102(2)	0.3909(1)	0.2058(3)	0.030(1)
C(21)	0.7632(2)	0.4200(1)	0.2333(4)	0.035(1)
C(22)	0.7705(2)	0.4543(1)	0.1737(4)	0.036(1)
C(23)	0.7232(2)	0.4598(1)	0.0867(4)	0.032(1)
C(24)	0.6699(2)	0.4310(1)	0.0582(3)	0.029(1)
C(25)	0.4222(3)	0.4591(1)	0.1165(4)	0.036(1)
C(26)	0.6251(3)	0.3586(1)	0.5502(4)	0.041(1)
C(27)	0.6366(2)	0.4959(1)	0.3308(3)	0.035(1)
C(28)	0.6278(3)	0.4720(1)	0.4315(4)	0.041(1)
C(29)	0.5865(3)	0.5496(1)	0.2152(3)	0.037(1)
C(30)	0.5216(3)	0.5787(1)	0.1946(4)	0.045(1)
C(31)	0.5796(2)	0.5575(1)	0.4139(3)	0.032(1)
C(32)	0.6539(3)	0.5800(2)	0.4265(4)	0.051(1)
C(33)	0.0373(2)	0.2871(1)	0.7004(3)	0.022(1)
C(34)	0.0887(2)	0.3056(1)	0.7704(3)	0.018(1)
C(35)	0.1377(2)	0.2845(1)	0.8392(3)	0.018(1)
C(36)	0.1340(2)	0.2434(1)	0.8360(3)	0.024(1)
C(37)	0.0833(2)	0.2238(1)	0.7677(3)	0.030(1)
C(38)	0.0363(2)	0.2452(1)	0.7004(3)	0.027(1)
C(39)	0.1932(2)	0.3052(1)	0.9108(3)	0.019(1)
C(40)	0.2430(2)	0.3352(1)	0.8755(3)	0.021(1)
C(41)	0.2987(2)	0.3505(1)	0.9417(3)	0.027(1)
C(42)	0.3051(2)	0.3372(1)	1.0458(3)	0.029(1)

C(43)	0.2555(2)	0.3084(1)	1.0827(3)	0.029(1)
C(44)	0.1998(2)	0.2923(1)	1.0164(3)	0.023(1)
C(45)	-0.0136(2)	0.3099(1)	0.6284(3)	0.025(1)
C(46)	-0.0565(2)	0.3430(1)	0.6626(3)	0.022(1)
C(47)	-0.1080(2)	0.3633(1)	0.5990(3)	0.025(1)
C(48)	-0.1154(3)	0.3503(1)	0.4935(3)	0.037(1)
C(49)	-0.0731(3)	0.3182(1)	0.4558(4)	0.045(1)
C(50)	-0.0230(3)	0.2979(1)	0.5227(3)	0.036(1)
C(51)	-0.1564(2)	0.3968(1)	0.6395(3)	0.023(1)
C(52)	-0.1983(2)	0.3927(1)	0.7326(3)	0.026(1)
C(53)	-0.2465(2)	0.4233(1)	0.7665(3)	0.031(1)
C(54)	-0.2525(2)	0.4581(1)	0.7083(3)	0.032(1)
C(55)	-0.2098(2)	0.4624(1)	0.6170(4)	0.030(1)
C(56)	-0.1618(2)	0.4322(1)	0.5827(3)	0.029(1)
C(57)	-0.0914(3)	0.3686(1)	1.0579(3)	0.040(1)
C(58)	0.1003(3)	0.4663(1)	0.6138(4)	0.037(1)
C(59)	0.0810(3)	0.4447(2)	0.2835(3)	0.050(1)
C(60)	0.0026(3)	0.4291(1)	0.3117(4)	0.058(2)
C(61)	0.0585(2)	0.4729(1)	0.1015(3)	0.029(1)
C(62)	0.0929(2)	0.5139(1)	0.1152(4)	0.035(1)
C(63)	0.1835(3)	0.4387(1)	0.1460(4)	0.044(1)
C(64)	0.2017(3)	0.4337(1)	0.0302(4)	0.047(1)
C(66A)	0.8080(14)	0.2273(7)	0.5117(16)	0.054(7)
C(67A)	0.7816(5)	0.2365(3)	0.4103(8)	0.058(3)
C(68A)	0.7135(5)	0.2571(3)	0.3977(7)	0.050(2)
C(69A)	0.6859(6)	0.2662(3)	0.2979(6)	0.049(2)
C(70A)	0.7325(6)	0.2572(4)	0.2142(9)	0.081(4)
C(71A)	0.8039(6)	0.2392(3)	0.2283(8)	0.066(3)
C(72A)	0.8289(5)	0.2273(3)	0.3263(6)	0.045(2)
C(66B)	0.7044(14)	0.2667(7)	0.1151(17)	0.084(8)
C(67B)	0.7816(5)	0.2365(3)	0.4103(8)	0.058(3)
C(68B)	0.7135(5)	0.2571(3)	0.3977(7)	0.050(2)
C(69B)	0.6859(6)	0.2662(3)	0.2979(6)	0.049(2)
C(70B)	0.7325(6)	0.2572(4)	0.2142(9)	0.081(4)
C(71B)	0.8039(6)	0.2392(3)	0.2283(8)	0.066(3)
C(72B)	0.8289(5)	0.2273(3)	0.3263(6)	0.045(2)

F(1)	0.4068(2)	0.4260(1)	0.0608(2)	0.050(1)
F(2)	0.3693(2)	0.4864(1)	0.0915(2)	0.059(1)
F(3)	0.4901(2)	0.4727(1)	0.0855(2)	0.048(1)
F(4)	0.6696(2)	0.3609(1)	0.4653(3)	0.057(1)
F(5)	0.6221(2)	0.3946(1)	0.5953(2)	0.055(1)
F(6)	0.6559(2)	0.3332(1)	0.6191(3)	0.067(1)
F(7)	-0.0909(2)	0.4070(1)	1.0851(2)	0.052(1)
F(8)	-0.1149(2)	0.3476(1)	1.1405(2)	0.054(1)
F(9)	-0.1412(1)	0.3641(1)	0.9791(2)	0.054(1)
F(10)	0.0308(2)	0.4805(1)	0.5906(2)	0.049(1)
F(11)	0.1180(2)	0.4393(1)	0.5417(2)	0.060(1)
F(12)	0.1506(2)	0.4965(1)	0.6051(2)	0.059(1)
N(1)	0.4874(2)	0.4200(1)	0.2693(2)	0.018(1)
N(2)	0.5012(2)	0.3751(1)	0.4370(2)	0.019(1)
N(3)	0.5793(2)	0.5289(1)	0.3216(2)	0.027(1)
N(4)	0.0261(2)	0.3832(1)	0.9302(2)	0.022(1)
N(5)	0.0377(2)	0.4164(1)	0.7415(3)	0.025(1)
N(6)	0.0979(2)	0.4408(1)	0.1663(3)	0.032(1)
O(1)	0.4191(1)	0.3525(1)	0.2693(2)	0.017(1)
O(2)	0.5626(1)	0.3541(1)	0.2591(2)	0.018(1)
O(3)	0.4381(2)	0.4875(1)	0.3036(2)	0.030(1)
O(4)	0.3447(2)	0.4341(1)	0.2785(2)	0.029(1)
O(5)	0.5379(2)	0.3032(1)	0.4696(2)	0.030(1)
O(6)	0.4872(2)	0.3451(1)	0.6141(2)	0.034(1)
O(7)	0.0944(1)	0.3475(1)	0.7683(2)	0.020(1)
O(8)	-0.0481(1)	0.3549(1)	0.7697(2)	0.018(1)
O(9)	0.0496(2)	0.3621(1)	1.1148(2)	0.034(1)
O(10)	-0.0019(2)	0.3121(1)	0.9887(2)	0.031(1)
O(11)	0.1806(2)	0.4303(1)	0.7563(2)	0.034(1)
O(12)	0.0878(2)	0.4803(1)	0.8142(2)	0.031(1)
P(1)	0.4921(1)	0.3755(1)	0.3127(1)	0.015(1)
P(2)	0.0281(1)	0.3761(1)	0.8058(1)	0.018(1)
S(1)	0.4203(1)	0.4489(1)	0.2583(1)	0.020(1)
S(2)	0.5279(1)	0.3417(1)	0.5154(1)	0.024(1)
S(3)	0.0056(1)	0.3530(1)	1.0195(1)	0.026(1)
S(4)	0.1045(1)	0.4465(1)	0.7481(1)	0.023(1)

Table S2b. Bond lengths [Å] and angles [°].

C(1)-C(2)	1.393(5)	C(1)-C(6)	1.397(5)
C(1)-C(13)	1.479(5)	C(2)-C(3)	1.395(5)
C(2)-O(1)	1.414(4)	C(3)-C(4)	1.404(5)
C(3)-C(7)	1.480(5)	C(4)-C(5)	1.383(5)
C(5)-C(6)	1.384(5)	C(7)-C(12)	1.393(5)
C(7)-C(8)	1.396(5)	C(8)-C(9)	1.382(5)
C(9)-C(10)	1.368(6)	C(10)-C(11)	1.391(6)
C(11)-C(12)	1.389(5)	C(13)-C(18)	1.383(5)
C(13)-C(14)	1.398(5)	C(14)-C(15)	1.389(5)
C(14)-O(2)	1.412(4)	C(15)-C(16)	1.408(6)
C(15)-C(19)	1.485(5)	C(16)-C(17)	1.384(6)
C(17)-C(18)	1.375(6)	C(19)-C(24)	1.389(5)
C(19)-C(20)	1.394(6)	C(20)-C(21)	1.384(6)
C(21)-C(22)	1.377(6)	C(22)-C(23)	1.380(6)
C(23)-C(24)	1.386(6)	C(25)-F(3)	1.326(5)
C(25)-F(1)	1.336(5)	C(25)-F(2)	1.336(5)
C(25)-S(1)	1.813(4)	C(26)-F(4)	1.320(6)
C(26)-F(6)	1.327(5)	C(26)-F(5)	1.332(5)
C(26)-S(2)	1.836(5)	C(27)-N(3)	1.494(5)
C(27)-C(28)	1.503(6)	C(29)-N(3)	1.511(5)
C(29)-C(30)	1.513(6)	C(31)-N(3)	1.504(5)
C(31)-C(32)	1.505(6)	C(33)-C(34)	1.399(5)
C(33)-C(38)	1.403(5)	C(33)-C(45)	1.479(5)
C(34)-C(35)	1.404(5)	C(34)-O(7)	1.409(4)
C(35)-C(36)	1.379(5)	C(35)-C(39)	1.490(5)
C(36)-C(37)	1.395(5)	C(37)-C(38)	1.379(5)
C(39)-C(40)	1.399(5)	C(39)-C(44)	1.400(5)
C(40)-C(41)	1.376(5)	C(41)-C(42)	1.386(6)
C(42)-C(43)	1.374(6)	C(43)-C(44)	1.387(5)
C(45)-C(50)	1.396(5)	C(45)-C(46)	1.403(5)
C(46)-C(47)	1.380(5)	C(46)-O(8)	1.411(4)
C(47)-C(48)	1.400(6)	C(47)-C(51)	1.494(5)
C(48)-C(49)	1.385(6)	C(49)-C(50)	1.389(6)
C(51)-C(52)	1.384(6)	C(51)-C(56)	1.387(5)

C(52)-C(53)	1.391(5)	C(53)-C(54)	1.379(6)
C(54)-C(55)	1.375(6)	C(55)-C(56)	1.379(6)
C(57)-F(8)	1.320(5)	C(57)-F(9)	1.325(5)
C(57)-F(7)	1.329(5)	C(57)-S(3)	1.832(5)
C(58)-F(11)	1.317(5)	C(58)-F(10)	1.332(5)
C(58)-F(12)	1.341(5)	C(58)-S(4)	1.813(5)
C(59)-C(60)	1.504(7)	C(59)-N(6)	1.506(6)
C(61)-C(62)	1.510(5)	C(61)-N(6)	1.513(5)
C(63)-C(64)	1.497(7)	C(63)-N(6)	1.513(6)
C(66A)-C(67A)	1.388(18)	C(67A)-C(72A)	1.373(7)
C(67A)-C(68A)	1.381(7)	C(68A)-C(69A)	1.377(7)
C(69A)-C(70A)	1.361(7)	C(70A)-C(71A)	1.393(7)
C(71A)-C(72A)	1.364(7)	C(66B)-C(70B)	1.375(18)
C(67B)-C(72B)	1.373(7)	C(67B)-C(68B)	1.381(7)
C(68B)-C(69B)	1.377(7)	C(69B)-C(70B)	1.361(7)
C(70B)-C(71B)	1.393(7)	C(71B)-C(72B)	1.364(7)
N(1)-S(1)	1.524(3)	N(1)-P(1)	1.589(3)
N(2)-S(2)	1.560(3)	N(2)-P(1)	1.569(3)
N(4)-S(3)	1.551(3)	N(4)-P(2)	1.581(3)
N(5)-S(4)	1.543(3)	N(5)-P(2)	1.579(3)
O(1)-P(1)	1.581(2)	O(2)-P(1)	1.573(2)
O(3)-S(1)	1.447(3)	O(4)-S(1)	1.428(3)
O(5)-S(2)	1.425(3)	O(6)-S(2)	1.431(3)
O(7)-P(2)	1.573(2)	O(8)-P(2)	1.571(2)
O(9)-S(3)	1.453(3)	O(10)-S(3)	1.429(3)
O(11)-S(4)	1.436(3)	O(12)-S(4)	1.434(3)
C(2)-C(1)-C(6)	117.5(3)	C(2)-C(1)-C(13)	122.5(3)
C(6)-C(1)-C(13)	119.9(3)	C(1)-C(2)-C(3)	123.4(3)
C(1)-C(2)-O(1)	117.8(3)	C(3)-C(2)-O(1)	118.7(3)
C(2)-C(3)-C(4)	116.9(3)	C(2)-C(3)-C(7)	123.4(3)
C(4)-C(3)-C(7)	119.6(3)	C(5)-C(4)-C(3)	121.0(3)
C(4)-C(5)-C(6)	120.5(3)	C(5)-C(6)-C(1)	120.7(3)
C(12)-C(7)-C(8)	118.4(3)	C(12)-C(7)-C(3)	119.0(3)
C(8)-C(7)-C(3)	122.3(3)	C(9)-C(8)-C(7)	120.0(4)
C(10)-C(9)-C(8)	121.4(4)	C(9)-C(10)-C(11)	119.6(4)

C(12)-C(11)-C(10)	119.5(4)	C(11)-C(12)-C(7)	121.1(4)
C(18)-C(13)-C(14)	117.7(3)	C(18)-C(13)-C(1)	120.7(3)
C(14)-C(13)-C(1)	121.5(3)	C(15)-C(14)-C(13)	123.2(3)
C(15)-C(14)-O(2)	118.3(3)	C(13)-C(14)-O(2)	118.4(3)
C(14)-C(15)-C(16)	116.7(3)	C(14)-C(15)-C(19)	123.1(3)
C(16)-C(15)-C(19)	120.2(4)	C(17)-C(16)-C(15)	120.8(4)
C(18)-C(17)-C(16)	120.5(4)	C(17)-C(18)-C(13)	121.0(4)
C(24)-C(19)-C(20)	118.5(4)	C(24)-C(19)-C(15)	120.0(4)
C(20)-C(19)-C(15)	121.4(3)	C(21)-C(20)-C(19)	120.4(4)
C(22)-C(21)-C(20)	120.8(4)	C(21)-C(22)-C(23)	119.2(4)
C(22)-C(23)-C(24)	120.6(4)	C(23)-C(24)-C(19)	120.5(4)
F(3)-C(25)-F(1)	108.1(4)	F(3)-C(25)-F(2)	108.0(3)
F(1)-C(25)-F(2)	107.9(4)	F(3)-C(25)-S(1)	111.7(3)
F(1)-C(25)-S(1)	110.8(3)	F(2)-C(25)-S(1)	110.3(3)
F(4)-C(26)-F(6)	109.1(4)	F(4)-C(26)-F(5)	108.3(4)
F(6)-C(26)-F(5)	108.6(4)	F(4)-C(26)-S(2)	111.5(3)
F(6)-C(26)-S(2)	109.3(3)	F(5)-C(26)-S(2)	110.1(3)
N(3)-C(27)-C(28)	113.0(3)	N(3)-C(29)-C(30)	112.6(3)
N(3)-C(31)-C(32)	113.8(3)	C(34)-C(33)-C(38)	116.8(3)
C(34)-C(33)-C(45)	122.6(3)	C(38)-C(33)-C(45)	120.6(3)
C(33)-C(34)-C(35)	123.6(3)	C(33)-C(34)-O(7)	118.3(3)
C(35)-C(34)-O(7)	118.0(3)	C(36)-C(35)-C(34)	117.1(3)
C(36)-C(35)-C(39)	120.8(3)	C(34)-C(35)-C(39)	122.1(3)
C(35)-C(36)-C(37)	121.2(3)	C(38)-C(37)-C(36)	120.5(3)
C(37)-C(38)-C(33)	120.9(3)	C(40)-C(39)-C(44)	118.2(3)
C(40)-C(39)-C(35)	123.0(3)	C(44)-C(39)-C(35)	118.7(3)
C(41)-C(40)-C(39)	120.7(4)	C(40)-C(41)-C(42)	120.5(4)
C(43)-C(42)-C(41)	119.6(4)	C(42)-C(43)-C(44)	120.6(4)
C(43)-C(44)-C(39)	120.3(4)	C(50)-C(45)-C(46)	117.1(3)
C(50)-C(45)-C(33)	120.2(3)	C(46)-C(45)-C(33)	122.7(3)
C(47)-C(46)-C(45)	123.9(3)	C(47)-C(46)-O(8)	118.6(3)
C(45)-C(46)-O(8)	117.4(3)	C(46)-C(47)-C(48)	117.0(3)
C(46)-C(47)-C(51)	122.7(3)	C(48)-C(47)-C(51)	120.3(3)
C(49)-C(48)-C(47)	121.0(4)	C(48)-C(49)-C(50)	120.5(4)
C(49)-C(50)-C(45)	120.5(4)	C(52)-C(51)-C(56)	118.9(3)
C(52)-C(51)-C(47)	120.6(3)	C(56)-C(51)-C(47)	120.5(3)

C(51)-C(52)-C(53)	120.2(4)	C(54)-C(53)-C(52)	120.4(4)
C(55)-C(54)-C(53)	119.3(4)	C(54)-C(55)-C(56)	120.7(4)
C(55)-C(56)-C(51)	120.5(4)	F(8)-C(57)-F(9)	108.9(4)
F(8)-C(57)-F(7)	108.5(3)	F(9)-C(57)-F(7)	107.9(4)
F(8)-C(57)-S(3)	109.8(3)	F(9)-C(57)-S(3)	111.9(3)
F(7)-C(57)-S(3)	109.8(3)	F(11)-C(58)-F(10)	107.9(4)
F(11)-C(58)-F(12)	108.0(4)	F(10)-C(58)-F(12)	107.9(3)
F(11)-C(58)-S(4)	112.3(3)	F(10)-C(58)-S(4)	111.8(3)
F(12)-C(58)-S(4)	108.9(3)	C(60)-C(59)-N(6)	112.2(4)
C(62)-C(61)-N(6)	113.9(3)	C(64)-C(63)-N(6)	112.2(4)
C(72A)-C(67A)-C(68A)	122.5(9)	C(72A)-C(67A)-C(66A)	117.2(12)
C(68A)-C(67A)-C(66A)	120.0(12)	C(69A)-C(68A)-C(67A)	121.0(9)
C(70A)-C(69A)-C(68A)	116.4(9)	C(69A)-C(70A)-C(71A)	122.1(11)
C(72A)-C(71A)-C(70A)	121.6(10)	C(71A)-C(72A)-C(67A)	116.0(9)
C(72B)-C(67B)-C(68B)	122.5(9)	C(69B)-C(68B)-C(67B)	121.0(9)
C(70B)-C(69B)-C(68B)	116.4(9)	C(69B)-C(70B)-C(66B)	115.8(13)
C(69B)-C(70B)-C(71B)	122.1(11)	C(66B)-C(70B)-C(71B)	122.1(13)
C(72B)-C(71B)-C(70B)	121.6(10)	C(71B)-C(72B)-C(67B)	116.0(9)
S(1)-N(1)-P(1)	131.87(19)	S(2)-N(2)-P(1)	131.53(18)
C(27)-N(3)-C(31)	114.2(3)	C(27)-N(3)-C(29)	110.7(3)
C(31)-N(3)-C(29)	112.9(3)	S(3)-N(4)-P(2)	128.52(19)
S(4)-N(5)-P(2)	127.7(2)	C(59)-N(6)-C(63)	111.2(4)
C(59)-N(6)-C(61)	112.0(3)	C(63)-N(6)-C(61)	112.8(3)
C(2)-O(1)-P(1)	120.1(2)	C(14)-O(2)-P(1)	120.7(2)
C(34)-O(7)-P(2)	123.3(2)	C(46)-O(8)-P(2)	119.4(2)
N(2)-P(1)-O(2)	110.09(15)	N(2)-P(1)-O(1)	114.82(15)
O(2)-P(1)-O(1)	104.95(12)	N(2)-P(1)-N(1)	110.71(15)
O(2)-P(1)-N(1)	108.72(14)	O(1)-P(1)-N(1)	107.25(14)
O(8)-P(2)-O(7)	104.93(12)	O(8)-P(2)-N(5)	109.17(15)
O(7)-P(2)-N(5)	106.76(15)	O(8)-P(2)-N(4)	109.52(15)
O(7)-P(2)-N(4)	113.75(15)	N(5)-P(2)-N(4)	112.37(16)
O(4)-S(1)-O(3)	115.98(16)	O(4)-S(1)-N(1)	117.99(15)
O(3)-S(1)-N(1)	111.65(16)	O(4)-S(1)-C(25)	104.84(19)
O(3)-S(1)-C(25)	102.35(18)	N(1)-S(1)-C(25)	101.17(19)
O(5)-S(2)-O(6)	118.73(15)	O(5)-S(2)-N(2)	115.55(15)
O(6)-S(2)-N(2)	110.05(16)	O(5)-S(2)-C(26)	105.2(2)

O(6)-S(2)-C(26)	103.0(2)	N(2)-S(2)-C(26)	101.78(18)
O(10)-S(3)-O(9)	118.28(16)	O(10)-S(3)-N(4)	116.71(16)
O(9)-S(3)-N(4)	109.63(17)	O(10)-S(3)-C(57)	105.1(2)
O(9)-S(3)-C(57)	102.07(19)	N(4)-S(3)-C(57)	102.44(18)
O(12)-S(4)-O(11)	116.45(17)	O(12)-S(4)-N(5)	113.30(17)
O(11)-S(4)-N(5)	116.79(16)	O(12)-S(4)-C(58)	103.96(18)
O(11)-S(4)-C(58)	104.0(2)	N(5)-S(4)-C(58)	99.16(19)

Table S3b. Anisotropic displacement parameters (Å^2).

The anisotropic displacement factor exponent takes the form:

$-2\pi^2[h^2a^{*2}U_{11} + ... + 2 h k a^* b^* U_{12}]$.

	U_{11}	U_{22}	U_{33}	U_{23}	U_{13}	U_{12}
C(1)	0.018(2)	0.018(2)	0.019(2)	-0.004(2)	0.003(2)	-0.007(2)
C(2)	0.018(2)	0.011(2)	0.018(2)	-0.001(1)	0.000(2)	-0.004(1)
C(3)	0.020(2)	0.018(2)	0.012(2)	-0.001(1)	-0.004(1)	-0.002(2)
C(4)	0.025(2)	0.018(2)	0.023(2)	0.002(2)	0.004(2)	-0.008(2)
C(5)	0.033(2)	0.012(2)	0.032(2)	-0.002(2)	0.002(2)	-0.004(2)
C(6)	0.031(2)	0.020(2)	0.025(2)	-0.009(2)	0.008(2)	-0.004(2)
C(7)	0.019(2)	0.014(2)	0.016(2)	-0.004(1)	0.000(2)	-0.009(1)
C(8)	0.023(2)	0.019(2)	0.020(2)	0.001(2)	0.003(2)	-0.004(2)
C(9)	0.030(2)	0.023(2)	0.032(2)	-0.004(2)	0.009(2)	0.002(2)
C(10)	0.036(2)	0.027(2)	0.030(2)	-0.005(2)	0.013(2)	-0.005(2)
C(11)	0.044(3)	0.028(2)	0.022(2)	-0.002(2)	0.010(2)	-0.010(2)
C(12)	0.028(2)	0.021(2)	0.018(2)	0.001(2)	0.001(2)	-0.005(2)
C(13)	0.022(2)	0.020(2)	0.021(2)	-0.005(2)	0.009(2)	-0.002(2)
C(14)	0.022(2)	0.022(2)	0.016(2)	-0.002(2)	0.006(2)	-0.001(2)
C(15)	0.031(2)	0.024(2)	0.027(2)	-0.004(2)	0.009(2)	-0.004(2)
C(16)	0.049(3)	0.036(2)	0.026(2)	-0.003(2)	0.015(2)	-0.011(2)
C(17)	0.058(3)	0.045(3)	0.024(2)	-0.010(2)	0.017(2)	-0.015(2)
C(18)	0.046(3)	0.030(2)	0.028(2)	-0.007(2)	0.011(2)	-0.013(2)
C(19)	0.018(2)	0.020(2)	0.034(2)	-0.002(2)	0.011(2)	-0.002(2)
C(20)	0.023(2)	0.025(2)	0.042(3)	0.003(2)	0.007(2)	-0.004(2)
C(21)	0.023(2)	0.036(2)	0.046(3)	0.001(2)	0.000(2)	-0.002(2)

C(22)	0.021(2)	0.031(2)	0.056(3)	-0.008(2)	0.008(2)	-0.005(2)
C(23)	0.032(2)	0.021(2)	0.043(3)	0.001(2)	0.014(2)	-0.003(2)
C(24)	0.029(2)	0.027(2)	0.032(2)	0.005(2)	0.011(2)	-0.002(2)
C(25)	0.042(3)	0.035(2)	0.031(2)	0.006(2)	-0.004(2)	-0.003(2)
C(26)	0.044(3)	0.037(3)	0.043(3)	0.008(2)	-0.017(2)	-0.010(2)
C(27)	0.030(2)	0.036(2)	0.037(2)	-0.009(2)	0.008(2)	0.003(2)
C(28)	0.039(3)	0.037(2)	0.047(3)	-0.002(2)	-0.006(2)	0.005(2)
C(29)	0.052(3)	0.041(2)	0.018(2)	-0.004(2)	0.013(2)	-0.011(2)
C(30)	0.074(4)	0.033(2)	0.028(2)	0.003(2)	0.001(3)	0.001(2)
C(31)	0.041(2)	0.031(2)	0.024(2)	-0.011(2)	0.005(2)	-0.002(2)
C(32)	0.044(3)	0.059(3)	0.049(3)	-0.030(3)	0.010(2)	-0.019(2)
C(33)	0.025(2)	0.022(2)	0.019(2)	-0.003(2)	-0.002(2)	0.005(2)
C(34)	0.024(2)	0.017(2)	0.014(2)	-0.005(1)	0.002(2)	0.002(1)
C(35)	0.019(2)	0.021(2)	0.015(2)	-0.002(1)	0.004(2)	0.000(2)
C(36)	0.029(2)	0.023(2)	0.021(2)	-0.003(2)	-0.005(2)	0.007(2)
C(37)	0.036(2)	0.017(2)	0.037(2)	-0.008(2)	-0.002(2)	0.007(2)
C(38)	0.028(2)	0.025(2)	0.030(2)	-0.011(2)	-0.006(2)	0.008(2)
C(39)	0.022(2)	0.017(2)	0.018(2)	-0.005(2)	0.000(2)	0.006(2)
C(40)	0.021(2)	0.017(2)	0.023(2)	-0.004(2)	0.001(2)	0.007(2)
C(41)	0.023(2)	0.021(2)	0.037(2)	-0.005(2)	0.000(2)	0.001(2)
C(42)	0.038(2)	0.022(2)	0.029(2)	-0.007(2)	-0.014(2)	0.004(2)
C(43)	0.043(3)	0.026(2)	0.019(2)	-0.002(2)	-0.010(2)	0.004(2)
C(44)	0.029(2)	0.020(2)	0.021(2)	-0.001(2)	-0.002(2)	0.004(2)
C(45)	0.029(2)	0.024(2)	0.022(2)	-0.004(2)	-0.004(2)	0.006(2)
C(46)	0.025(2)	0.021(2)	0.020(2)	-0.003(2)	-0.005(2)	0.000(2)
C(47)	0.027(2)	0.025(2)	0.022(2)	-0.001(2)	-0.002(2)	0.001(2)
C(48)	0.045(3)	0.041(2)	0.027(2)	0.000(2)	-0.010(2)	0.014(2)
C(49)	0.059(3)	0.045(3)	0.029(3)	-0.013(2)	-0.012(2)	0.019(2)
C(50)	0.045(3)	0.039(2)	0.025(2)	-0.012(2)	-0.008(2)	0.012(2)
C(51)	0.022(2)	0.024(2)	0.023(2)	0.001(2)	-0.007(2)	-0.001(2)
C(52)	0.023(2)	0.026(2)	0.029(2)	0.003(2)	-0.005(2)	0.001(2)
C(53)	0.024(2)	0.036(2)	0.032(2)	-0.002(2)	-0.002(2)	0.001(2)
C(54)	0.025(2)	0.033(2)	0.039(3)	-0.006(2)	-0.007(2)	0.004(2)
C(55)	0.032(2)	0.023(2)	0.036(2)	0.002(2)	-0.006(2)	0.001(2)
C(56)	0.029(2)	0.033(2)	0.025(2)	-0.001(2)	-0.001(2)	0.001(2)
C(57)	0.047(3)	0.051(3)	0.021(2)	0.007(2)	0.009(2)	0.013(2)

C(58)	0.050(3)	0.032(2)	0.030(2)	0.001(2)	0.002(2)	0.005(2)
C(59)	0.074(4)	0.056(3)	0.020(2)	0.000(2)	-0.006(2)	0.010(3)
C(60)	0.099(5)	0.043(3)	0.032(3)	-0.006(2)	0.017(3)	-0.015(3)
C(61)	0.025(2)	0.035(2)	0.026(2)	0.001(2)	-0.005(2)	0.007(2)
C(62)	0.036(3)	0.031(2)	0.039(2)	-0.007(2)	-0.009(2)	0.003(2)
C(63)	0.033(3)	0.041(3)	0.057(3)	-0.002(2)	-0.013(2)	0.012(2)
C(64)	0.027(2)	0.043(3)	0.070(4)	-0.027(2)	0.004(2)	0.009(2)
F(1)	0.067(2)	0.056(2)	0.027(1)	-0.009(1)	-0.009(1)	-0.009(1)
F(2)	0.064(2)	0.055(2)	0.058(2)	0.028(1)	-0.020(2)	0.010(1)
F(3)	0.055(2)	0.057(2)	0.032(1)	0.019(1)	0.006(1)	-0.015(1)
F(4)	0.029(2)	0.070(2)	0.072(2)	0.009(2)	-0.007(2)	-0.008(1)
F(5)	0.064(2)	0.046(2)	0.055(2)	-0.001(1)	-0.028(2)	-0.023(1)
F(6)	0.065(2)	0.061(2)	0.075(2)	0.022(2)	-0.046(2)	-0.004(2)
F(7)	0.064(2)	0.044(1)	0.050(2)	0.000(1)	0.020(1)	0.029(1)
F(8)	0.057(2)	0.062(2)	0.044(2)	0.016(1)	0.024(1)	0.015(1)
F(9)	0.030(1)	0.086(2)	0.045(2)	0.010(2)	0.004(1)	0.014(1)
F(10)	0.064(2)	0.039(1)	0.044(2)	0.014(1)	-0.016(1)	0.009(1)
F(11)	0.093(2)	0.063(2)	0.023(1)	-0.010(1)	0.009(1)	0.008(2)
F(12)	0.077(2)	0.052(2)	0.046(2)	0.020(1)	0.009(2)	-0.021(2)
N(1)	0.018(2)	0.014(1)	0.023(2)	0.002(1)	0.001(1)	-0.002(1)
N(2)	0.026(2)	0.014(1)	0.016(2)	0.001(1)	0.000(1)	0.002(1)
N(3)	0.036(2)	0.024(2)	0.021(2)	-0.004(1)	0.001(2)	-0.005(1)
N(4)	0.025(2)	0.022(2)	0.019(2)	-0.002(1)	0.000(1)	0.004(1)
N(5)	0.028(2)	0.018(1)	0.028(2)	0.008(1)	-0.006(2)	-0.001(1)
N(6)	0.037(2)	0.031(2)	0.029(2)	0.000(2)	-0.005(2)	0.010(2)
O(1)	0.019(1)	0.014(1)	0.017(1)	0.000(1)	0.001(1)	-0.002(1)
O(2)	0.020(1)	0.016(1)	0.018(1)	-0.002(1)	0.004(1)	0.000(1)
O(3)	0.034(2)	0.017(1)	0.041(2)	-0.005(1)	0.002(1)	0.000(1)
O(4)	0.022(1)	0.023(1)	0.042(2)	0.005(1)	-0.002(1)	-0.001(1)
O(5)	0.045(2)	0.016(1)	0.028(2)	0.006(1)	-0.002(1)	0.001(1)
O(6)	0.055(2)	0.032(1)	0.016(1)	0.003(1)	0.003(1)	-0.008(1)
O(7)	0.022(1)	0.016(1)	0.021(1)	0.000(1)	0.000(1)	0.001(1)
O(8)	0.019(1)	0.020(1)	0.014(1)	-0.002(1)	-0.001(1)	0.003(1)
O(9)	0.050(2)	0.036(2)	0.016(1)	-0.003(1)	-0.004(1)	0.014(1)
O(10)	0.041(2)	0.026(1)	0.026(1)	0.004(1)	0.003(1)	0.004(1)
O(11)	0.026(2)	0.032(1)	0.045(2)	0.005(1)	-0.002(1)	0.004(1)

O(12)	0.037(2)	0.026(1)	0.029(2)	-0.007(1)	0.004(1)	-0.005(1)
P(1)	0.018(1)	0.011(1)	0.017(1)	-0.001(1)	0.001(1)	-0.001(1)
P(2)	0.023(1)	0.014(1)	0.016(1)	0.000(1)	-0.001(1)	0.002(1)
S(1)	0.021(1)	0.014(1)	0.023(1)	0.002(1)	0.000(1)	-0.001(1)
S(2)	0.032(1)	0.020(1)	0.020(1)	0.002(1)	-0.004(1)	-0.005(1)
S(3)	0.033(1)	0.026(1)	0.018(1)	0.001(1)	0.002(1)	0.010(1)
S(4)	0.029(1)	0.017(1)	0.023(1)	0.002(1)	0.001(1)	0.001(1)

Table S4b. Hydrogen coordinates and isotropic displacement parameters ($Å^2$).

	x	y	z	U_{eq}
H(4)	0.3349	0.2339	0.3785	0.026
H(5)	0.4123	0.2000	0.2578	0.030
H(6)	0.4908	0.2349	0.1406	0.030
H(8)	0.2742	0.3537	0.3073	0.025
H(9)	0.1805	0.3792	0.4200	0.034
H(10)	0.1646	0.3543	0.5903	0.037
H(11)	0.2415	0.3011	0.6491	0.038
H(12)	0.3369	0.2756	0.5375	0.027
H(16)	0.6398	0.3658	-0.0767	0.044
H(17)	0.5599	0.3144	-0.1333	0.051
H(18)	0.4832	0.2805	-0.0129	0.041
H(20)	0.7059	0.3674	0.2477	0.036
H(21)	0.7949	0.4162	0.2941	0.042
H(22)	0.8076	0.4739	0.1921	0.043
H(23)	0.7271	0.4837	0.0460	0.038
H(24)	0.6377	0.4352	-0.0019	0.035
H(27A)	0.6890	0.5073	0.3284	0.042
H(27B)	0.6309	0.4778	0.2690	0.042
H(28A)	0.5742	0.4634	0.4389	0.062
H(28B)	0.6420	0.4886	0.4927	0.062
H(28C)	0.6613	0.4486	0.4286	0.062
H(29A)	0.5871	0.5293	0.1579	0.045

H(29B)	0.6359	0.5642	0.2126	0.045
H(30A)	0.4723	0.5654	0.2076	0.068
H(30B)	0.5238	0.5878	0.1204	0.068
H(30C)	0.5267	0.6017	0.2423	0.068
H(31A)	0.5694	0.5424	0.4802	0.038
H(31B)	0.5373	0.5769	0.4044	0.038
H(32A)	0.6501	0.5980	0.4879	0.076
H(32B)	0.6638	0.5957	0.3620	0.076
H(32C)	0.6960	0.5611	0.4379	0.076
H(36)	0.1666	0.2282	0.8811	0.029
H(37)	0.0812	0.1954	0.7677	0.036
H(38)	0.0028	0.2314	0.6533	0.033
H(40)	0.2382	0.3452	0.8049	0.025
H(41)	0.3330	0.3703	0.9159	0.033
H(42)	0.3436	0.3478	1.0914	0.035
H(43)	0.2594	0.2995	1.1544	0.035
H(44)	0.1659	0.2724	1.0429	0.028
H(48)	-0.1499	0.3637	0.4470	0.045
H(49)	-0.0785	0.3101	0.3838	0.053
H(50)	0.0051	0.2757	0.4963	0.043
H(52)	-0.1940	0.3690	0.7734	0.031
H(53)	-0.2755	0.4202	0.8300	0.037
H(54)	-0.2859	0.4789	0.7312	0.039
H(55)	-0.2133	0.4865	0.5770	0.036
H(56)	-0.1323	0.4357	0.5198	0.035
H(59A)	0.1203	0.4299	0.3245	0.060
H(59B)	0.0844	0.4732	0.3041	0.060
H(60A)	-0.0367	0.4457	0.2777	0.087
H(60B)	-0.0041	0.4299	0.3892	0.087
H(60C)	-0.0024	0.4015	0.2868	0.087
H(61A)	0.0610	0.4654	0.0252	0.034
H(61B)	0.0036	0.4739	0.1220	0.034
H(62A)	0.0647	0.5331	0.0711	0.053
H(62B)	0.0895	0.5219	0.1901	0.053
H(62C)	0.1469	0.5134	0.0932	0.053
H(63A)	0.2079	0.4635	0.1723	0.053

H(63B)	0.2055	0.4160	0.1863	0.053
H(64A)	0.2576	0.4325	0.0207	0.070
H(64B)	0.1786	0.4089	0.0041	0.070
H(64C)	0.1810	0.4564	-0.0098	0.070
H(66A)	0.7694	0.2352	0.5645	0.064
H(66B)	0.8170	0.1985	0.5168	0.064
H(66C)	0.8561	0.2416	0.5254	0.064
H(68A)	0.6853	0.2651	0.4588	0.060
H(69A)	0.6369	0.2782	0.2879	0.058
H(70A)	0.7158	0.2634	0.1441	0.097
H(71A)	0.8361	0.2351	0.1681	0.079
H(72A)	0.8760	0.2135	0.3358	0.054
H(66D)	0.7420	0.2591	0.0607	0.101
H(66E)	0.6562	0.2523	0.1028	0.101
H(66F)	0.6949	0.2955	0.1111	0.101
H(67B)	0.7964	0.2284	0.4799	0.069
H(68B)	0.6853	0.2651	0.4588	0.060
H(69B)	0.6369	0.2782	0.2879	0.058
H(71B)	0.8361	0.2351	0.1681	0.079
H(72B)	0.8760	0.2135	0.3358	0.054
H(3A)	0.5276	0.5159	0.3219	0.033
H(6A)	0.0759	0.4146	0.1429	0.039

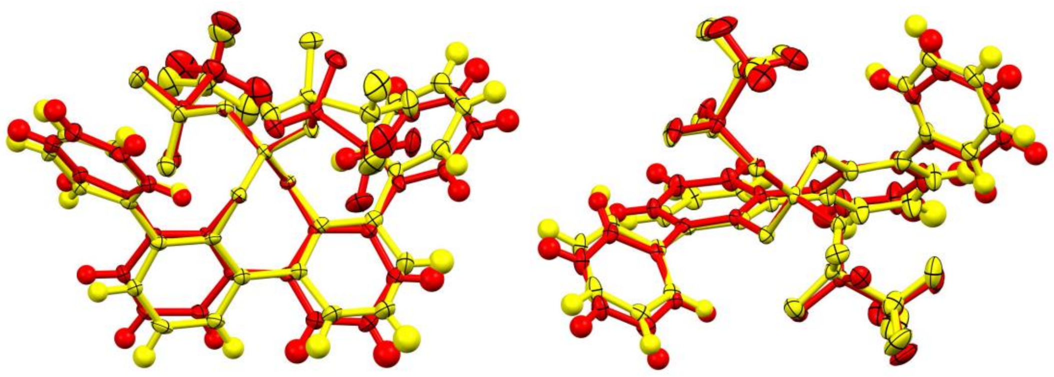

Figure S3b. Superposition of the PO_2N_2 units of the two independent anions, showing the slightly different conformations in the crystal. View from the side and from above.
